# Pan-cancer analyses reveal cancer-type-specific fungal ecologies and bacteriome interactions

**DOI:** 10.1016/j.cell.2022.09.005

**Published:** 2022-09-29

**Authors:** Lian Narunsky-Haziza, Gregory D. Sepich-Poore, Ilana Livyatan, Omer Asraf, Cameron Martino, Deborah Nejman, Nancy Gavert, Jason E. Stajich, Guy Amit, Antonio González, Stephen Wandro, Gili Perry, Ruthie Ariel, Arnon Meltser, Justin P. Shaffer, Qiyun Zhu, Nora Balint-Lahat, Iris Barshack, Maya Dadiani, Einav N. Gal-Yam, Sandip Pravin Patel, Amir Bashan, Austin D. Swafford, Yitzhak Pilpel, Rob Knight, Ravid Straussman

**Affiliations:** 1 Department of Molecular Cell Biology, Weizmann Institute of Science, Rehovot, Israel; 2 Department of Molecular Genetics, Weizmann Institute of Science, Rehovot, Israel; 3 Department of Bioengineering, University of California San Diego, La Jolla, CA, USA; 4 Department of Computer Science and Applied Mathematics, Weizmann Institute of Science, Rehovot, Israel; 5 Bioinformatics and Systems Biology Program, University of California, San Diego, La Jolla, CA, USA; 6 Center for Microbiome Innovation, University of California San Diego, La Jolla, CA, USA; 7 Department of Pediatrics, University of California San Diego School of Medicine, La Jolla, CA, USA; 8 Department of Microbiology and Plant Pathology, Institute for Integrative Genome Biology, University of California Riverside, Riverside, CA, USA; 9 Department of Physics, Bar-Ilan University, Ramat-Gan, Israel; 10 Department of Natural Sciences, The Open University of Israel, Raanana, Israel; 11 Micronoma Inc., San Diego, CA, USA; 12 Cancer Research Center, Sheba Medical Center, Ramat Gan, Israel; 13 School of Life Sciences, Arizona State University, Tempe, AZ, USA; 14 Biodesign Center for Fundamental and Applied Microbiomics, Arizona State University, Tempe, AZ, USA; 15 Sackler Faculty of Medicine, Tel-Aviv University, Tel-Aviv, Israel; 16 Department of Pathology, Sheba Medical Center, Ramat Gan, Israel; 17 Breast Oncology Institute, Sheba Medical Center, Ramat Gan, Israel; 18 Moores Cancer Center, University of California San Diego Health, La Jolla, CA, USA; 19 Department of Computer Science and Engineering, University of California San Diego, La Jolla, CA, USA

**Keywords:** tumor mycobiome, tumor microbiome, cancer, biomarkers, fungi, microbial interactions, liquid biopsy, metagenomics, metatranscriptomics

## Abstract

Cancer-microbe associations have been explored for centuries, but cancer-associated fungi have rarely been examined. Here, we comprehensively characterize the cancer mycobiome within 17,401 patient tissue, blood, and plasma samples across 35 cancer types in four independent cohorts. We report fungal DNA and cells at low abundances across many major human cancers, with differences in community compositions that differ among cancer types, even when accounting for technical background. Fungal histological staining of tissue microarrays supported intratumoral presence and frequent spatial association with cancer cells and macrophages. Comparing intratumoral fungal communities with matched bacteriomes and immunomes revealed co-occurring bi-domain ecologies, often with permissive, rather than competitive, microenvironments and distinct immune responses. Clinically focused assessments suggested prognostic and diagnostic capacities of the tissue and plasma mycobiomes, even in stage I cancers, and synergistic predictive performance with bacteriomes.

## Highlights

• Fungi were detected in 35 cancer types and were often intracellular
• Multiple fungal-bacterial-immune ecologies were detected across tumors
• Intratumoral fungi stratified clinical outcomes, including immunotherapy response
• Cell-free fungal DNA diagnosed healthy and cancer patients in early-stage disease

## Graphical abstract

**Figure:**
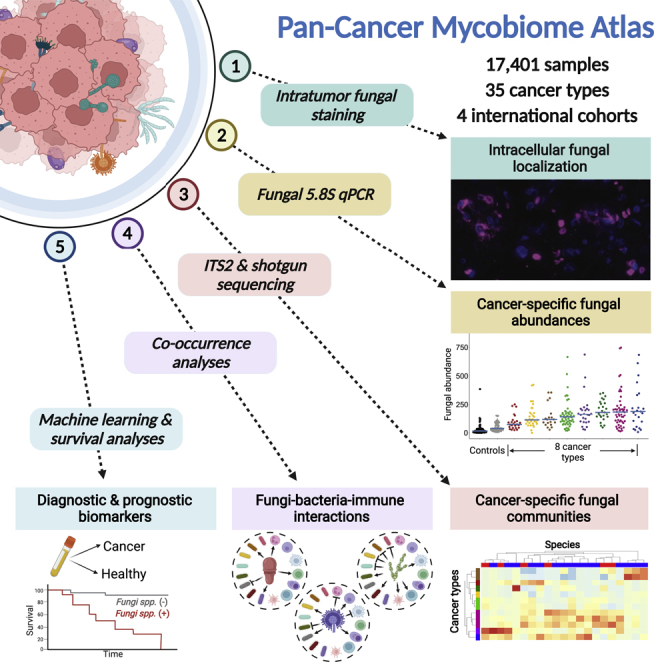

## Teaser

Characterization of fungi across multiple sample types and patient cohorts across 35 cancer types reveals their distribution, association with immune cell types, and potential prognostic value, including synergy with bacteria.

## Introduction

Fungi are understudied but important commensals/opportunistic pathogens that shape host immunity and infect the immunocompromised, including cancer patients (Galloway-Peña and Kontoyiannis, 2020; Iliev et al., 2012; Jain et al., 2021; Köhler et al., 2014; Ost et al., 2021; Underhill and Iliev, 2014). Fungi were found in individual tumor types (Alam et al., 2022; Aykut et al., 2019; Banerjee et al., 2015, 2017, 2018, 2019; Gamal et al., 2022; Luan et al., 2015; Mukherjee et al., 2017; Perera et al., 2017; Zhu et al., 2017) and contribute to carcinogenesis in esophageal and pancreatic cancer (Alam et al., 2022; Aykut et al., 2019; Zhu et al., 2017), but their presence, identity, location, and effects in most cancer types are unknown. Recent studies found metabolically active, immunoreactive, intracellular, and cancer type-specific communities of bacteria and viruses in tumor tissues (Geller et al., 2017; Kalaora et al., 2021; Le Noci et al., 2018; Mima et al., 2015; Nejman et al., 2020; Parhi et al., 2020; Poore et al., 2020; Pushalkar et al., 2018; Riquelme et al., 2019; Sepich-Poore et al., 2021; Shi et al., 2020; Tsay et al., 2021; Wieland et al., 2021; Zapatka et al., 2020), leading to their inclusion in updated cancer ‘hallmarks’ (Hanahan, 2022). Many of these bacteria affect cancer therapies (Geller et al., 2017; Kalaora et al., 2021; Le Noci et al., 2018; Nejman et al., 2020; Pushalkar et al., 2018; Shi et al., 2020; Wieland et al., 2021). Whether fungi act similarly and should be included under the cancer hallmarks’ polymorphic microbiomes is unknown, motivating characterization of the pan-cancer mycobiome. Symbiotic and antagonistic relationships between fungi and bacteria (Frey-Klett et al., 2011; Peleg et al., 2010; Shiao et al., 2021) further motivate studying their interactions in tumors, and recent data suggest that combining their information provides synergistic diagnostic performance for colorectal cancer (Liu et al., 2022). We thus comprehensively characterized cancer mycobiomes in tissues and blood, compared fungal communities with matched bacteriomes and immunomes, and explored fungal utility for prognosis and diagnosis.

## Results

### Fungal nucleic acids exist in many human cancer types

**Figure 1:**
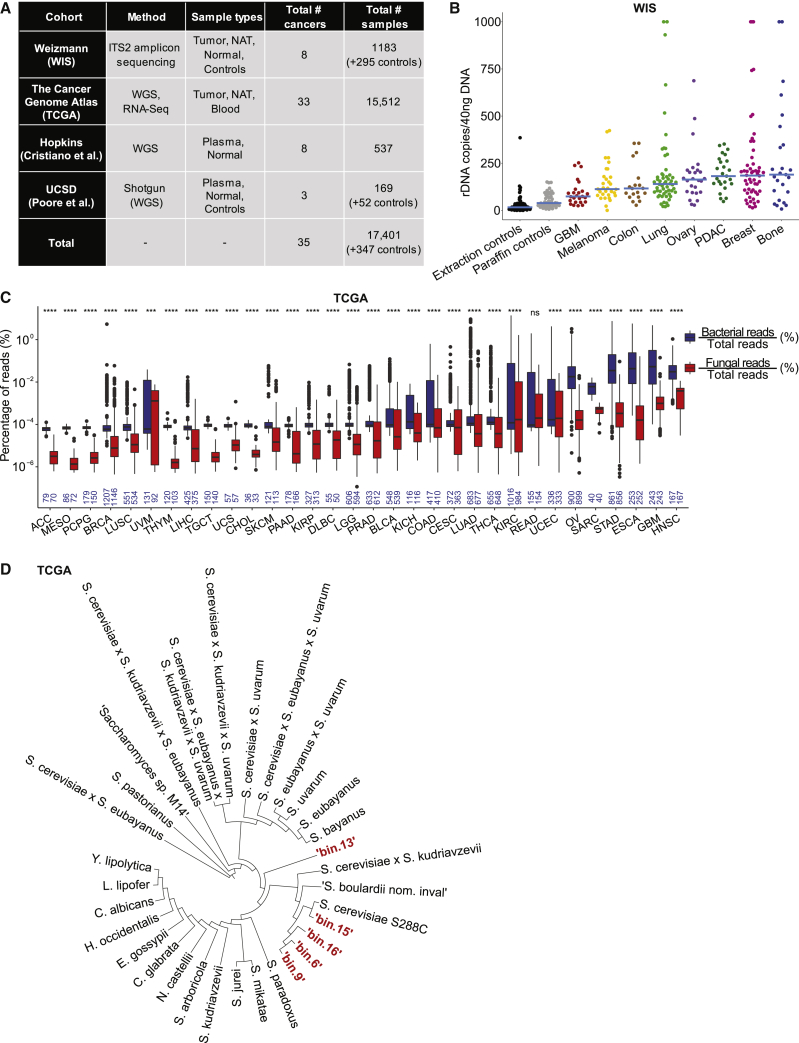
Fungal nucleic acids exist in human cancers (A) Table of all studied samples. (B) Fungal DNA abundance in WIS cohort quantified by 5.8*S* qPCR. Blue bars show medians. Values clipped at 1,000. One-sided t tests between tumor types and extraction controls (n = 89, far left): paraffin controls (n = 48), p = 5.8 × 10^−4^; GBM (n = 25), p = 3.1 × 10^−5^; melanoma (n = 31), p = 2.1 × 10^−7^; colon (n = 19), p = 6.6 × 10^−5^; lung (n = 56), p = 2.1 × 10^−6^; ovary (n = 26), p = 4.2 × 10^−6^; pancreas (n = 25), p = 4.5 × 10^−10^; bone (n = 25), p = 0.014; and breast (n = 54), p = 1.5 × 10^−5^. All p values have an FDR of ≤0.2. (C) Percentage of fungal or bacterial reads in TCGA primary tumors versus total reads. Sample sizes inset in blue, and vary slightly when samples had only bacterial counts. Two-sided Wilcoxon tests for each cancer type; ∗∗∗∗p ≤ 0.0001; ∗∗∗p ≤ 0.001; ns, not significant. Boxplots show median, 25^th^ and 75^th^ percentiles, and 1.5 × interquartile range (IQR). See Data S1.2F for paired analysis. (D) Phylogenomics of TCGA-derived fungal bins >85 kbp using Benchmarking Universal Single-Copy Orthologs (BUSCO) against NCBI fungal genomes.

We profiled fungal DNA in two large cohorts of cancer samples we previously examined for bacteria (Nejman et al., 2020; Poore et al., 2020). The first (Weizmann [WIS]) comprised 1,183 formalin-fixed paraffin-embedded (FFPE) or frozen samples of tumor and normal adjacent tissue (NAT; often paired) from eight tissue types (breast, lung, melanoma, ovary, colon, brain, bone, and pancreas) and non-cancer normal breast tissue. All samples were studied for fungal presence using internal transcribed spacer 2 (ITS2) amplicon sequencing (Figure 1A; Tables S1 and S2). To account for potential contamination by environmental fungi or fungal DNA introduced via sample handling and processing, we included 104 paraffin-only and 191 DNA-extraction negative controls. These controls enabled detection and removal of fungal contaminants and separation of signal from noise in ITS2 data (STAR Methods).

The second cohort encompassed whole genome sequencing (WGS) and transcriptome sequencing (RNA-seq) data from The Cancer Genome Atlas (TCGA) (Figure 1A; Table S1). For quality control, we re-aligned all (∼10^11^) unmapped DNA and RNA reads to a uniform human reference (GRCh38), then removed poor-quality reads. Remaining reads were aligned to the RefSeq release 200 multi-domain database of 11,955 microbial (with 320 fungal) genomes (STAR Methods). 15,512 samples (WGS: 4,736; RNA-seq: 10,776) had non-zero microbial feature counts, of which 15,065 (97%) contained fungi. Of 6.06 × 10^12^ total reads, 7.3% did not map to the human genome: 98.8% of these unmapped reads mapped to no organism in our microbial database. Of the remaining 1.2% of non-human reads that mapped to our microbial database (0.08% of total reads), 80.2% (0.067% of total) were classified as bacterial, and 2.3% (0.002% of total) as fungal, providing 1.172 × 10^8^ fungal reads for downstream analyses with an average read length of 57.4 bp (SD = 15.9; median = 51bp; methods enforced 45-bp minimum). Fungal-containing TCGA samples had an average of 7,780 (95% CI: [7,039, 8,521]) fungal reads/sample. Although TCGA lacked contamination controls, we implemented *in silico* decontamination based on sequencing plate and center (Poore et al., 2020) and cross-referenced all fungal species against the WIS decontaminated amplicon cohort, the Human Microbiome Project (HMP)’s gut mycobiome cohort (Nash et al., 2017), and >100 other publications to obtain a final decontaminated list (Table S3).

We quantified fungal DNA in the WIS cohort using quantitative polymerase chain reaction (qPCR) of the fungal 5.8*S* ribosomal gene in a random subset comprising 261 tumor samples and 137 negative controls (Figure 1B; Data S1.1A). All tumor types tested had higher fungal load than negative controls and fungal load differed among tumor types (Figure 1B). Fungal load was significantly higher in colon and lung tumors than adjacent tissue (Data S1.1B). A similar non-significant trend was found in breast tumors versus NAT and normal (Data S1.1B). Fungal and bacterial load correlated across tumor types (Data S1.1C), with breast and bone cancers highest in fungal (Figure 1B) and bacterial (Nejman et al., 2020) DNA. We then subjected all WIS samples to ITS2 amplification and sequencing to characterize fungi. This analysis also found more fungal reads in all cancer types than in negative controls (Data S1.1D).

In the TCGA cohort, we observed significant, cancer type-specific differences in the percentage of classified fungal, bacterial, and pan-microbial reads of the total or unmapped reads (Data S1.1E-S1.1H and S1.2A-S1.2D). In 31 of 32 cancer types, bacterial read proportions in primary tumors were significantly higher than fungal reads (Figure 1C), and all cancer types had significantly higher bacterial proportions during paired analyses (Data S1.2F) or after normalizing by genome sizes (Data S1.2E). Calculating average relative abundances of bacteria and fungi in TCGA primary tumors revealed 86.7% bacteria and 13.3% fungi without genome size normalization (Data S1.3A) or 96% bacteria and 4% fungi (Data S1.3B) with normalization, suggesting that bacteria predominate over fungi in the tumor microbiome. Fungal and bacterial read proportions had high Spearman correlations (Data S1.3C–S1.3E), including primary tumors (ρ = 0.76, p < 2.2 × 10^−308^), NATs (ρ = 0.84, p < 2.2 × 10^−308^), and blood (ρ = 0.84, p < 2.2 × 10^−308^). These data support a bacterial-dominated but polymicrobial cancer microbiome.

Motivated by the ∼117 million fungal reads in TCGA, we calculated per-sample and aggregate fungal genome coverages across all WGS and RNA-seq samples (Table S4). This revealed 31 fungi with ≥1% aggregate genome coverage, including *Saccharomyces cerevisiae* (99.7% coverage), *Malassezia restricta* (98.6% coverage), *Candida albicans* (84.1% coverage), *Malassezia globosa* (40.5% coverage), and *Blastomyces gilchristii* (35.0% coverage). No one sample explained these top five aggregate coverages (Table S4.2). *M. restricta* and *globosa* had no samples above 26.0% or 4.3% coverage, respectively. *S. cerevisiae*, *C. albicans*, and *B. gilchristi* had no samples above 64.8%, 50.0%, or 30.0% coverage, respectively. Many fungi had equally contributing coverages from different diseases and sequencing centers (Data S1.3F–S1.3H). Moreover, WIS-TCGA overlapping fungi were significantly more likely to have ≥1% aggregate genome coverage than non-WIS-overlapping species (Fisher exact test: p = 1.05 × 10^−8^, odds ratio = 13.1). We constructed *de novo* metagenome co-assemblies per cancer type using non-human primary tumor WGS reads, finding large (>85 kb) fungal metagenome-assembled bins placed within *Saccharomyces* (Figure 1D; Data S1.4A). Smaller bins contained contigs matching the fungal mitochondrially encoded ATP synthase membrane subunit 6 (ATP6) locus of *Malassezia restricta* (Data S1.4B); the fungal mitochondrially encoded cyclooxygenase-2 (COX2) locus of *Blastomyces dermatitidis* (Data S1.4C); and two partial sequences of *Candida albicans*’s chromosome I and chromosome “R” (data not shown). Thus, metagenomic analyses support fungal presence and verify several well-covered taxa from our bioinformatic pipeline.

The WIS and TCGA cohorts each have distinct advantages and drawbacks. Advantages of the WIS cohort include aseptic sample processing, mechanical shearing to optimize microbial DNA extraction, hundreds of experimental contamination controls, complementary tissue imaging, and fungal-specific qPCR, which together improve confidence in the true presence/absence of intratumoral fungi. However, ITS2 amplicon sequencing precludes genome-wide coverage analyses and has limited taxonomic resolution (Data S1.5A). Conversely, the TCGA cohort’s shotgun metagenomic approach with large sample sizes enables reconstruction of near-complete fungal genomes, comparison with host information, inference across most human cancer types, and represents a scalable approach compatible with historical data; however, lacking experimental contamination controls, its *in silico* decontamination yields less confident presence/absence calls. Differences in sample preparation, sequencing, bioinformatic pipelines, and reference databases (STAR Methods)—which affect bacteriome analyses (Sinha et al., 2017)—exist between these cohorts. Despite these differences, we identified, within the intersection of the WIS cohort and TCGA fungal reference database, 87.2% of WIS species and 93.4% of fungal genera in matched TCGA cancer types (Data S1.5B and S1.5C). To be conservative, we included versions of TCGA mycobiome data subset to WIS-intersecting fungi, with similar conclusions irrespective of the cohort.

### Fungi are detected by multiple staining methods in human tumors

**Figure 2:**
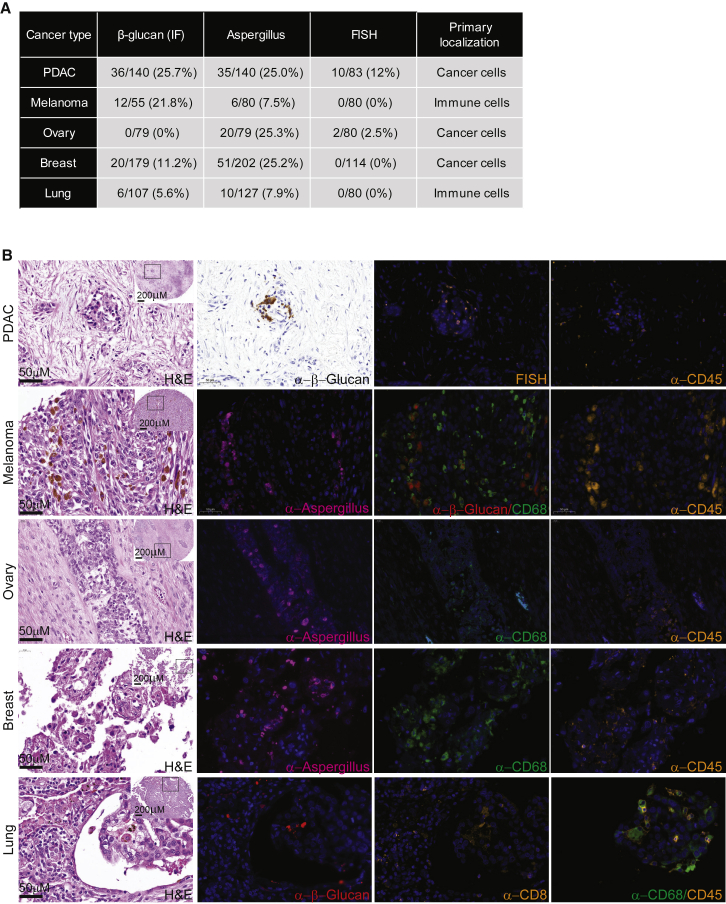
Visualization of fungi in human cancer tissue (A) Table summarizing percent of tumor microarray cores from five cancer types with positive fungal staining of α-β glucan, α-*Aspergillus*, and 28*S* rRNA FISH probes, and their localization. (B) Representative stained tumor microarrays from five cancer types using hematoxylin and eosin (H&E), antibodies against β-glucan, *Aspergillus*, CD45, CD68, CD8, and by FISH probes against fungal 28*S* rRNA sequences. Negative controls for all these cores are in Data S2.3. Scale bars shown. Squares in H&E images demarcate areas presented at higher magnification. PDAC, pancreatic adenocarcinoma; FISH, fluorescence *in situ* hybridization.

We visualized fungi in human tumors by staining melanoma, pancreas, breast, lung, and ovarian cancer tissue microarrays (Figure 2; Data S2.1A). Because no staining method can detect all fungi in tissues, we integrated four staining methods with varying levels of sensitivity and specificity: (1) a fungal cell wall-specific anti-β-glucan antibody with a high false-negative rate (Data S2.1), (2) an anti-*Aspergillus* antibody that also binds several additional fungal species (Data S2.1), (3) fluorescence *in situ* hybridization (FISH) against three conserved fungal 28*S* rRNA sequences with selective sensitivity for yeast over hyphal morphologies due to lower hyphae probe penetration (Data S2.1B and S2.2A), and (4) fungal cell wall-specific Gomori methenamine silver (GMS) stain with high false-positive background staining in tissues. Numerous negative controls mitigated false positives (Data S2.3 and S2.4). Overall, we found 0%–25% of the tumors per cancer type to be positive for either β-glucan or *Aspergillus* staining (Figure 2; Data S2.3, and S2.4). Fungal 28*S* rRNA FISH staining was less prevalent, showing positive stain in 12% of pancreatic cancer samples. GMS staining was difficult to interpret due to high background but was useful for rare cases where canonical fungal cells were identified (Data S2.2B).

Interestingly, images showed cancer type-specific localization patterns. Although fungal staining was mainly evident within cancer cells in pancreatic, breast, and ovarian cancer, it mostly localized to macrophages in melanoma and lung cancers (Figure 2; Data S2.3 and S2.4). In rare cases where canonical fungal cells were identified, they were extracellular (Data S2.2).

### Different cancer types exhibit cancer-type-specific mycobiomes

**Figure 3:**
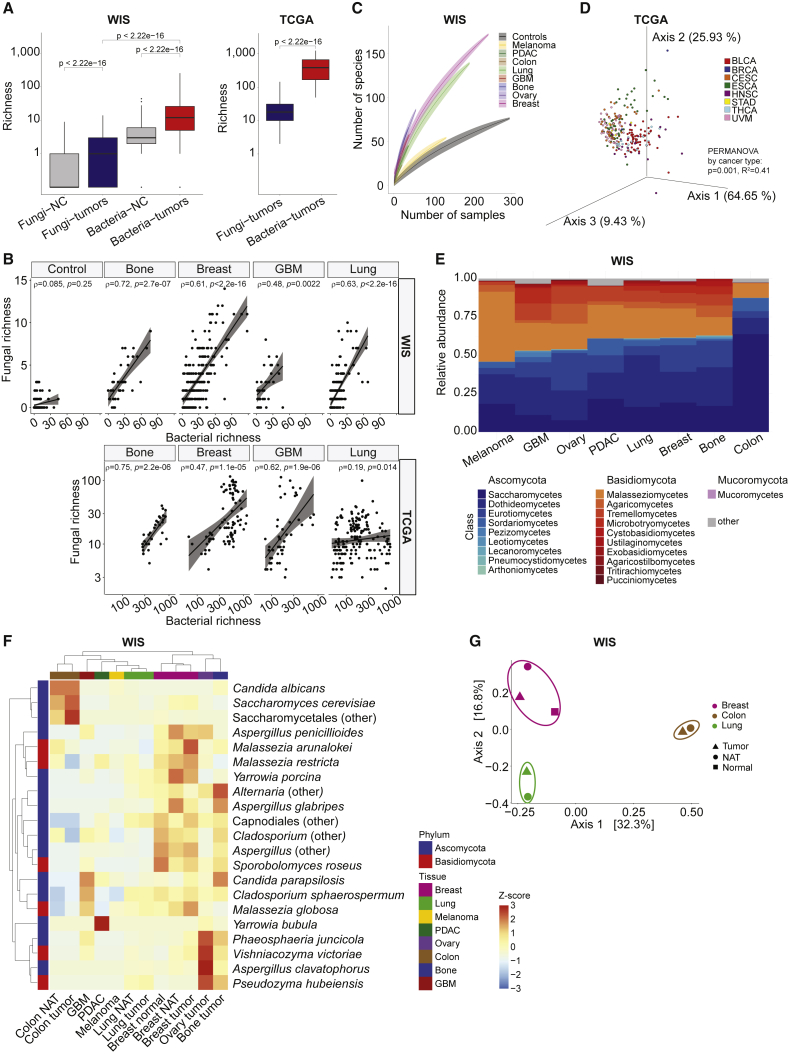
Different cancer types exhibit distinct mycobiomes (A) Fungal and bacterial species richness for WIS and TCGA cohorts. NC, negative controls. Boxplots show median, 25^th^ and 75^th^ percentiles, and 1.5 × IQR. t test p values inset on plots. (B) Scatter plot demonstrating significant Spearman correlations (ρ) and p values between fungal and bacterial richness in four tumor types shared between WIS and TCGA cohorts and no correlation in negative extraction controls. Linear regression lines and 95% confidence intervals shown. (C) Rarefaction plot of the number of species detected in the WIS cohort per tumor type with 100 random subsamples per number of samples. Mean and SD shown. Extraction and paraffin controls were grouped together. (D) Fungal beta diversity analyses using robust Aitchison PCA (Martino et al., 2019) on decontaminated mycobiome data from TCGA MD Anderson primary tumor (WGS) samples (n = 259, 8 cancer types). Permutational multivariate analysis of variance (PERMANOVA) statistics (999 permutations) shown on plot. (E) Mean relative abundance bar plots at class-level phylotypes across WIS tumor types. Colors correspond to fungal class. (F) Unsupervised hierarchical clustering of fungal prevalence in the WIS cohort using species that appear in ≥10% of samples in ≥1 tumor/NAT/normal tissue types. Values represent *Z* scores per row. Amplicon sequence variants (ASVs) without species level classification were aggregated by the lowest classification they received. (G) Principal coordinate analysis (PCoA) of Jaccard dissimilarities between composite fungal species profiles across tissues.

Tumor bacterial richness was significantly higher than fungal richness (Figure 3A), similar to the gut microbiome (Nash et al., 2017). Mycobiome richness varied significantly across cancer types in WIS and TCGA cohorts (Data S3.1). However, WIS (amplicon) cohort richness was lower than the TCGA (shotgun metagenomic) cohort, likely due to (1) numerous negative controls that were used to decontaminate the WIS cohort, (2) flooring the WIS data to counteract index hopping sequencing noise (Reitmeier et al., 2021), and (3) potential read splitting during shotgun metagenomic alignments in the TCGA cohort (STAR Methods). Interestingly, four of seven cancer types shared by both cohorts showed significant positive correlations between intratumoral fungal and bacterial richness (Figure 3B). No such correlation was observed in WIS extraction negative controls (Figure 3B).

WIS intratumoral mycobiome alpha diversity was low, but beta diversity was high between tumor samples (Data S3.2A), preventing rarefaction plot saturation (Figure 3C). We found that clustering by mycobiome composition grouped samples by cancer type (F = 1.65, R^2^ = 0.02, p = 0.029 by permutational multivariate analysis of variance [PERMANOVA]; Data S3.2B). Beta diversity analyses within TCGA sequencing centers similarly revealed cancer-type-specific mycobiome compositions (Figure 3D; Data S3.2C–S3.2G and S3.3).

Across WIS cancer types, Ascomycota and Basidiomycota phyla dominated the intratumor mycobiome (Figure 3E). The Ascomycota to Basidiomycota ratio (A/B ratio) was highest in colon cancer (A/B = 8.8), due to abundant Saccharomycetes, and lowest in melanoma (A/B = 0.86), due to abundant Malasseziomycetes. These differences correspond to the known fungal taxa that inhabit the gut (Nash et al., 2017) and skin (Findley et al., 2013), suggesting a possible seeding of the tumors from tissue-specific ecologies. Indeed, unsupervised clustering of tumors alongside normal and NAT samples showed tissue-specific clustering by the most prevalent fungi in these tissues (Figure 3F; Data S3.2H and S3.2I). Moreover, both WIS and TCGA cohorts demonstrated co-clustering of tumor and NAT samples when comparing beta-diversity scores, supporting similar tumor and NAT compositions (Figure 3G; Data S3.2J). PERMANOVA analyses within each TCGA disease type for Aitchison and Bray-Curtis distances also failed to show significant differences between tumor and NAT (Table S5). Co-clustering of tumor and NAT profiles in the WIS cohort still occurred after discarding from the analysis pairs of tumor-NAT samples from the same patients (Data S3.2K), which had a higher similarity than unmatched samples from the same tumor type (Data S3.2A). Collectively, these analyses portray ubiquitous, low-abundance, cancer-type-specific mycobiomes that have community assemblies similar to those in adjacent normal tissues.

### Intratumoral mycobiome-bacteriome-immunome interactions

Fungi interact with bacteria by physical and biochemical mechanisms (Peleg et al., 2010), motivating exploration of inter-domain connections between mycobiome and bacteriome data in WIS and TCGA cohorts (Nejman et al., 2020; Poore et al., 2020). For the WIS cohort, we compared presence/absence data at different taxonomic levels with shuffled counterparts to calculate the normalized mutual information between domains (Neeson and Mandelik, 2014). Most significant inter-domain co-occurrences presented in breast cancer, which had the most samples, potentially reflecting less power in other cancer types (Data S4.1A; Table S6). 96.5% (82 of 85) of significant fungi-bacteria co-occurrences in breast cancer were positive, with *Aspergillus* and *Malassezia* serving as hubs for inter-domain co-occurrences (Data S4.1A).

Since fungi and bacteria elicit unique host immune responses (Aykut et al., 2019; Iliev et al., 2012; Jain et al., 2021; Ost et al., 2021; Sepich-Poore et al., 2021; Shiao et al., 2021; Underhill and Iliev, 2014; Wolf and Underhill, 2018), we hypothesized that intratumoral fungal-bacterial-immune clusters exist. Because bacteriomes (Nejman et al., 2020; Poore et al., 2020), immunomes (Thorsson et al., 2018), and mycobiomes each demonstrate cancer type specificity, we also reasoned that multi-domain clusters likely vary across cancer types. We thus compared WIS-overlapping fungal and bacterial genera in TCGA with TCGA immune cell compositions derived from CIBERSORT (Newman et al., 2015; Thorsson et al., 2018), using a neural network method previously developed to estimate microbiome-metabolite co-occurrences (Morton et al., 2019a).

**Figure 4:**
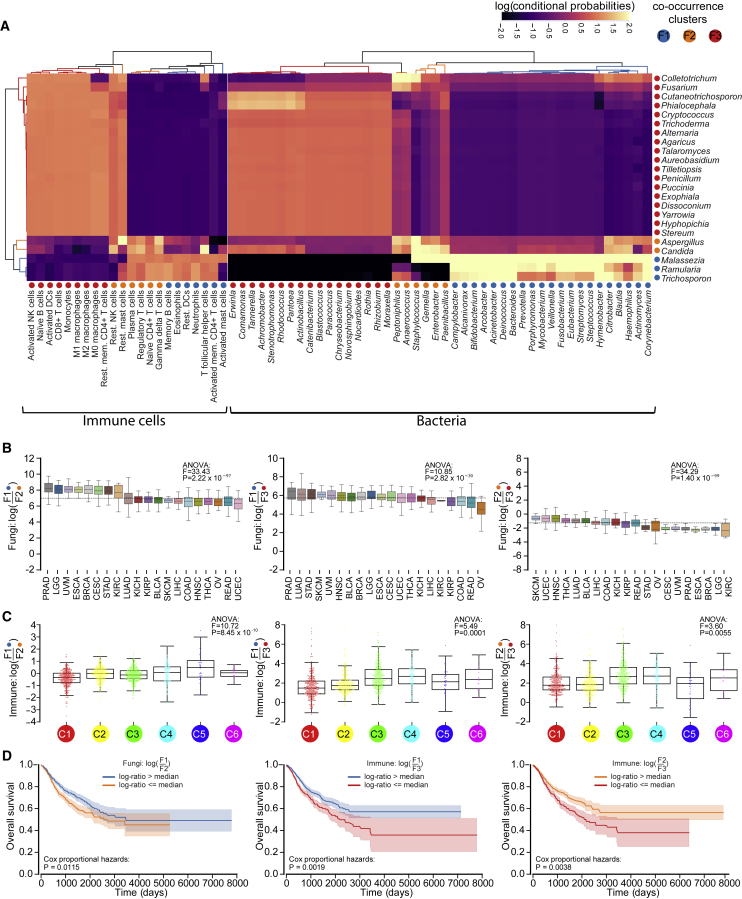
Establishing pan-cancer mycotypes through mycobiome-bacteriome-immunome interactions (A) Co-occurrence analyses of WIS-overlapping TCGA fungal and bacteria genera (Table S7.5), and TCGA immune cell compositions (Thorsson et al., 2018) using MMvec (Morton et al., 2019a). Hierarchical clustering linkage information identified three distinct clusters (“mycotypes”) associated with groups of fungal genera: F1, F2, and F3. (B) Log-ratios of fungal mycotype abundances across TCGA cancer types, revealing significantly differing values (one-way ANOVAs). (C) Varying mycotype immune log-ratios across pan-cancer immune subtypes (Thorsson et al., 2018). C1, wound healing; C2, IFN-γ dominant; C3, inflammatory; C4, lymphocyte depleted (but with second most macrophages); C5, immunologically quiet (but with most macrophages); C6, TGF-β dominant. Table S7.3 shows pairwise log-ratio comparisons across all immune subtypes. (D) Significant associations with overall survival in 20 cancer types based on the F1/F2 fungal mycotype log-ratio (left) or mycotype immune log-ratios (F1/F3, middle; F2/F3, right). Table S7.6 shows the sample sizes above and below the medians. Note: the C3 immune subtype has the best prognosis. (B and C) Boxplots show median, 25^th^, and 75^th^ percentiles and 1.5 × IQR.

Unsupervised analyses revealed three distinct fungi-bacteria-immune clusters driven by fungal co-occurrences, herein called “mycotypes,” named F1 (*Malassezia*-*Ramularia*-*Trichosporon*), F2 (*Aspergillus*-*Candida*), and F3 (multi-genera including *Yarrowia*) (Figure 4A). F1 and F2 mycotypes comprised fewer but more prevalent fungal genera (Data S4.1B). Raw counts were then aggregated within each domain (e.g., bacteria) and mycotype (e.g., F1) to form log-ratio comparisons (Morton et al., 2019b). Log-ratio denominators provide ‘reference frames’ for stable inferences between groups (Morton et al., 2019b), such that fungal F1/F2 denotes how *Malassezia*, *Ramularia*, and *Trichosporon* compositions change relative to *Aspergillus* and *Candida*. Mycotype log-ratios varied across TCGA and WIS cancer types (Figure 4B; Data S4.1C–S4.1F; Table S7.1). Six of nine TCGA log-ratios between domains significantly correlated (e.g., fungal F1/F2 versus bacterial F1/F2; Table S7.2), suggesting similar shifts within multi-domain ecologies among diverse human cancers and validating inferred co-occurrences.

We then tested whether mycotypes were associated with immune responses previously identified in TCGA patients (categorized into six immune subtypes, C1–C6 (Thorsson et al., 2018)) and/or patient survival. Log-ratios of immune cells co-occurring with F1, F2, or F3-clustered fungi significantly separated immune response subtypes (Figure 4C; Table S7.3), suggesting that different intratumoral mycobiomes may elicit distinct host responses. Two of the three significant comparisons were associated with higher inflammatory (C3) and lymphocyte-depleted, strong macrophage (C4) responses (Figure 4C, middle and right), whereas the third comparison (Figure 4C, left) was enriched in the two immune subtypes with the strongest macrophage responses (C4, C5) (Thorsson et al., 2018). Inflammatory responses (C3) have the best survival prognosis (Thorsson et al., 2018), and we found that a greater abundance of C3-linked immune mycotypes was associated with better overall survival (OS) across 20 cancer types (Figure 4D, middle and right) without sequencing center associations (Table S7.4). Although most fungal or bacterial comparisons did not stratify OS (Data S4.2A) or separate immune subtypes (Data S4.2B), the log-ratio of just five fungal genera (*Malassezia*-*Ramularia*-*Trichosporon* to *Aspergillus*-*Candida*) significantly prognosed pan-cancer survival (Figure 4D, left). Cox proportional hazards analyses confirmed that categorization using log-ratio medians significantly stratified OS in many individual cancer types—excluding separation solely by differential cancer ranking—and cancer type and stage (Data S4.3), warranting further investigation.

### Statistical and machine learning analyses demonstrate cancer-type-specific mycobiomes

**Figure 5:**
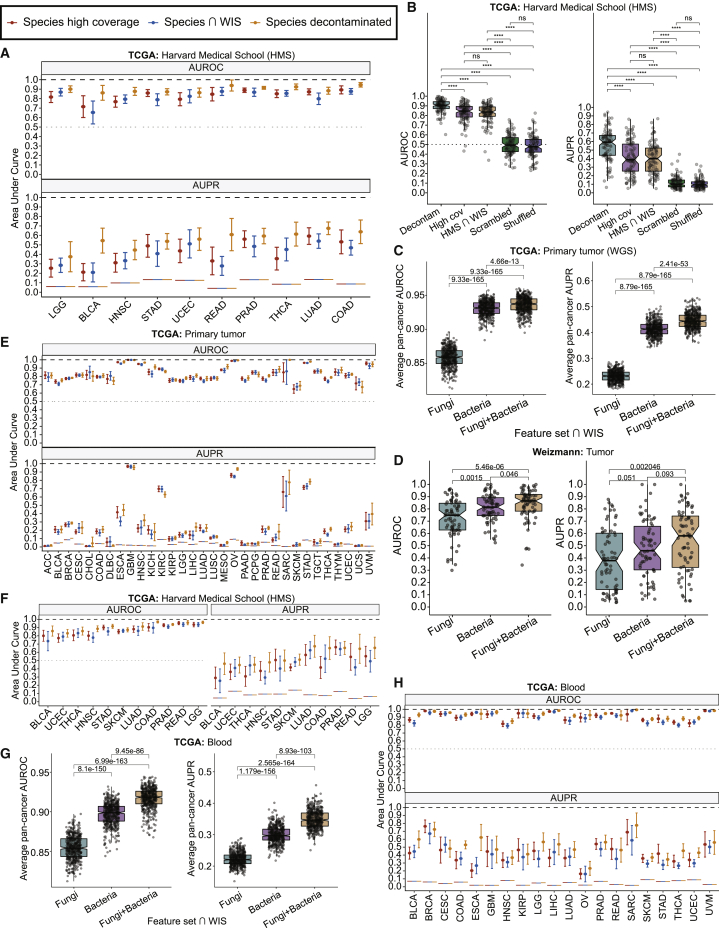
Machine learning (ML) analyses reveal cancer-type-specific tumor and blood mycobiomes (A) One-cancer-type-versus-all-others predictions on Harvard Medical School tumors (HMS, n = 876). (B) Negative control analyses for (A) using scrambled metadata or shuffled samples. All one-cancer-type-versus-all-others performances are aggregated. ^∗∗∗∗^ q < 0.001; ns, not significant. (C) Multi-class pan-cancer discrimination among TCGA WGS tumor samples using WIS-overlapping features across 500 independent folds (50 iterations of 10-fold CV). (D) Aggregated one-cancer-type-versus-all-others ML performance in WIS cohort tumors. (E) One-cancer-type-versus-all-others predictions using batch-corrected, TCGA primary tumor data (n = 10,998). (F) One-cancer-type-versus-all-others predictions using HMS blood samples (n = 835). (G) Multi-class pan-cancer discrimination among TCGA WGS blood samples using WIS-overlapping features across 500 independent folds (50 iterations of 10-fold CV). (H) One-cancer-type-versus-all-others predictions using batch-corrected, TCGA blood data (n = 1,771). (A, E, F, and H) Area under ROC curve (AUROC) and area under precision-recall curve (AUPR) measured on independent holdout folds (10-fold cross-validation [CV]) to estimate averages (dots) and 95% confidence intervals (brackets). “High coverage,” 31 fungal species with ≥1% aggregate genome coverage; “∩ Weizmann,” 34 WIS-overlapping fungal species; “decontaminated,” 224 decontaminated fungal species. Horizontal lines denote null AUROC or AUPR. (B, C, D, and G) Two-sided Wilcoxon tests with Benjamini-Hochberg correction. Boxplots show median, 25^th^, and 75^th^ percentiles and 1.5 × IQR.

We next tested whether machine learning (ML) on mycobiomes discriminates between and within cancer types. We first evaluated ML models on raw, decontaminated TCGA fungal count data (n = 14,495 non-zero decontaminated samples) with extensive positive and negative control analyses, revealing pan-cancer discrimination, and found synergistic performance when adding bacterial information in TCGA and WIS tumors (Figures 5A–5D; Data S5 Note and S5.1–S5.5). Toward building a pan-cancer classifier, we combined all decontaminated TCGA mycobiome data using supervised batch correction, as previously done with TCGA bacteriomes and viromes (Poore et al., 2020) (Data S5.6A). Evaluating one-cancer-type-versus-all-others models on batch-corrected mycobiome species revealed strong discrimination across 32 cancer types (Figure 5E; area under receiver operating characteristic [ROC] curve [AUROC] 95% CI: [83.27, 85.39]%). Negative controls showed null performances (Data S5.6B). We then cross-tested models built on two independent raw or batch-corrected TCGA halves, finding significantly correlated performance among primary tumor comparisons (Data S5.3G, S5.3H, S5.6C, and S5.6D). Subsetting the batch-corrected data to fungi identified by EukDetect (Table S8.1) (Lind and Pollard, 2021), a eukaryotic-specific, marker-based taxonomy assignment algorithm, gave strong performance similar to our high-coverage fungi (Data S5.1K–S5.1P). Notably, our 31 high coverage fungi were significantly more likely to be detected by EukDetect (Fisher exact test: p = 5.67 × 10^−11^, odds ratio = 28.0), suggesting that marker-based methods may be limited in low biomass settings.

We next applied differential abundance (DA) testing (Lin and Peddada, 2020) and ML between stage I and stage IV tumor mycobiomes. DA testing revealed stage-specific fungi for stomach, rectal, and renal cancers among RNA-seq samples (Data S5.7), and ML supported stomach and renal cancer stage differentiation (Data S5.8A), agreeing with previous results on stage-specific bacteriomes excluding colon cancer (Poore et al., 2020). Mycobiomes may not correlate with cancer stages, as defined by host tissue, for all cancer types.

Tumor and NAT mycobiome samples are similar in composition (Figures 3G; Data S3.2J); hence, discriminating them may be hard. Tumor versus NAT ML performed poorly on most TCGA raw data subsets and WIS data (Data S5.8B–S5.8G). Stomach and kidney cancers may comprise exceptions (Data S5.8B, S5.8C, S5.8E, and S5.8F) but were absent in the WIS cohort. Nonetheless, the small tumor-NAT effect size seemed surmountable when re-examining the full, batch-corrected dataset (Data S5.8H). Analogously, comparing breast tumors with true normal tissue in the WIS cohort revealed differential fungal prevalence and better ML performance (Data S5.8I and S5.8J). These analyses suggest tissue mycobiomes may distinguish tumor and NAT in sufficiently powered studies.

Previous bacteriome-centric analyses revealed cancer-type-specific, blood-derived microbial DNA (Poore et al., 2020), prompting us to examine fungal DNA in TCGA WGS blood samples. DA testing and ML on raw, decontaminated fungal data with extensive controls showed strong discrimination between cancer types and synergy with bacterial features (Figures 5F and 5G; Data S5 Note and S5.9–S5.12). ML on batch-corrected fungal species also showed pan-cancer discrimination (AUROC 95% CI: [92.42, 94.02]%; Figure 5H) with null performance on negative controls (Data S5.13A). Subsetting the analysis to stage Ia–IIc cancers in raw and batch-corrected datasets suggested stage-invariant performance (Data S5.13B and S5.13C).

We then repeated all raw and batch-corrected tumor, blood, and NAT analyses using differing ML model types and sampling strategies, finding similar results (Data S5.14 and S5.15), suggesting generalizable performance. Statistical and ML analyses support cancer-type-specific tissue and blood mycobiomes, with potential clinical utility. To encourage hypothesis generation, we summarized our results on an interactive website (http://cancermycobiome.ucsd.edu/).

### Clinical utility of cancer mycobiomes

We next explored the cancer mycobiome’s diagnostic and prognostic capacities, as previously established for cancer bacteriomes (Nejman et al., 2020; Poore et al., 2020; Sepich-Poore et al., 2021, 2022). Using the WIS cohort, we first tested fungal associations with disease phenotypes, patient survival, and treatment response.

**Figure 6:**
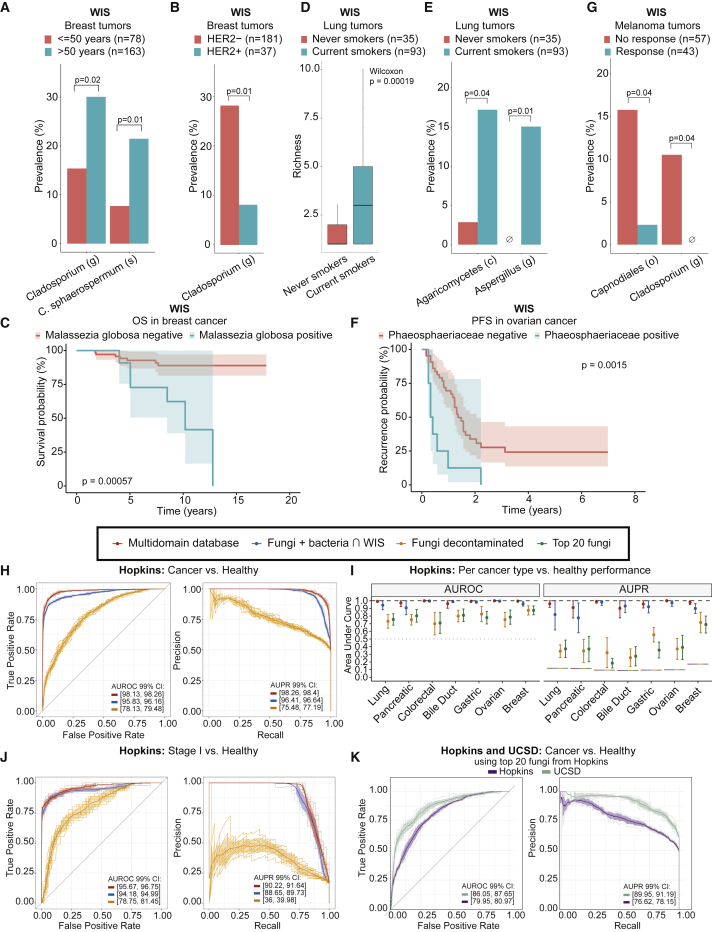
Clinical utility of cancer mycobiomes (A and B) Differential prevalence of fungal taxa in WIS breast tumors by (A) age or (B) human epidermal growth factor receptor 2 (HER2) status. (C) Kaplan-Meier survival probability of WIS breast cancer patients positive (n = 11) or negative (n = 69) for *Malassezia globosa*. p value from log-rank test. (D) Fungal richness in WIS lung tumors by smoking status. Boxplots: median, 25^th^ and 75^th^ percentiles, and 1.5 × IQR. (E) Differential prevalence of fungi in WIS lung tumors by smoking status. (F) Kaplan-Meier plot demonstrating progression free survival (PFS) probability in WIS ovarian patients positive (n = 9) or negative (n = 45) for Phaeosphaeriaceae family. p value from log-rank test. (G) Differential prevalence of fungi in WIS melanoma tumors by immune checkpoint inhibitor response. (H) Treatment-naive pan-cancer versus healthy discrimination in the Hopkins plasma cohort across all database hits (red, 7,418 features), WIS-overlapping fungi and bacteria (blue, 287 species), or decontaminated fungi (orange, 209 species). (I) Per cancer type versus healthy discrimination in the Hopkins cohort with 10-fold cross-validation. The “top 20 fungi” (green) are derived from pan-cancer versus healthy machine learning model. Dots and brackets represent average performance and 95% confidence intervals, respectively. Horizontal lines (gray or colored) denote null AUROCs and AUPRs. (J) Stage I pan-cancer versus healthy discrimination in the Hopkins cohort with equivalent feature sets and colors as (H). (K) Pan-cancer versus healthy controls discrimination in the Hopkins (purple) and UCSD (teal) plasma cohorts using the “top 20 fungi” features. (A, B, E, and G) p values calculated by Fisher’s exact test. (H, J, and K) 10-fold cross-validation repeated ten times. Mean performance with 99% confidence intervals (colored ribbons) and gray or lightly colored lines each denoting single repeats.

In breast cancer, we found *Cladosporium sphaerospermum* and the *Cladosporium* genus, previously reported in breast cancer (Banerjee et al., 2021), enriched in tumors of patients ≥50 years old. *Cladosporium* was also enriched in human epidermal growth factor receptor 2 (HER2) negative tumors (Figures 6A and 6B), although known age-HER2-status associations complicate causality (Howlader et al., 2014). We also found significantly shorter OS in patients with intratumoral *Malassezia globosa* (Figure 6C), a common fungus on human skin (Findley et al., 2013), in breast milk (Boix-Amorós et al., 2017), and in pancreatic tumors, in which it has oncogenic effects (Alam et al., 2022; Aykut et al., 2019). *Malassezia restricta*, another abundant skin fungus present in breast cancer, was not correlated with OS (data not shown). In lung cancer, we found higher intratumoral fungal richness and enrichment of *Aspergillus* and Agaricomycetes in current smokers compared with never smokers (Figures 6D and 6E). In ovarian cancer, patients with intratumoral Phaeosphaeriaceae, or related *Phaeosphaeria* genus, had significantly shorter progression free survival (PFS), shortening median PFS from 498 to 135 days (Figure 6F; Data S6.1A). We also examined fungal associations with immunotherapy response in metastatic melanoma. Fungal richness did not significantly vary (p=0.88, two-sided Wilcoxon test), but Capnodiales, and its genus, *Cladosporium*, were significantly enriched in non-responders (Figure 6G).

Blood-derived, stage-invariant, cancer-type-specific fungal compositions in TCGA suggest their utility as minimally invasive diagnostics, analogous to bacterial counterparts (Poore et al., 2020). We validated these findings in two independent, published cohorts (Hopkins, UCSD) comprising 330 healthy and 376 cancer-bearing subjects (Table S1) that underwent shallow whole genome plasma sequencing (Cristiano et al., 2019; Poore et al., 2020). The Hopkins cohort focused on treatment-naive, early-stage cancers, whereas the UCSD cohort focused on treated, late-stage cancers, collectively addressing most clinical scenarios across 10 cancer types. Additionally, the Hopkins cohort benchmarked well established (Lo et al., 2021), state-of-the-art fragmentomic diagnostics (Cristiano et al., 2019), providing direct performance comparisons to microbial-centric methods.

The Hopkins cohort underwent the same stringent human-read removal, microbial classification, and fungal decontamination as TCGA (n = 537; 8 cancer types). Examining treatment-naive, earliest-time point samples (n = 491), we estimated pan-cancer-versus-healthy diagnostic performance of raw microbial abundances using a published ML framework and hyperparameters (Cristiano et al., 2019). Decontaminated fungal species (n = 209) provided moderate discriminatory performance, and performance with multi-domain feature sets exceeded state-of-the-art, fragmentomic approaches (average AUROCs: 96%–98%), including a subset of 287 WIS tumor-overlapping fungi and bacteria (Figure 6H). Running ML models with WIS-overlapping fungi, bacteria, or both also revealed significant, synergistic performances (Data S6.1B). Per cancer type ML versus controls performed similarly (Figure 6I), with best fungal performance in breast cancer (AUROC 95% CI: [81.40, 93.53]%). Fungal discriminatory performance mostly plateaued at the taxonomic class level until species (Data S6.1C). Negative controls had null performances (Data S6.1D). All log-ratios of fungi from treatment-naive TCGA tumor mycotypes (Figure 4B) significantly varied among treatment-naive Hopkins cancer types in plasma (Data S6.1E–S6.1G), and the F1/F3 fungal log-ratio was significantly higher in cancer than controls (Data S6.1H). Testing ML models between cancer types also revealed moderate discrimination for decontaminated fungi and best performance with multi-domain features (Data S6.2A). Collectively, these analyses suggest clinical utility of plasma-derived, multi-domain microbial nucleic acids in treatment-naive patients.

We then focused our ML analyses on Hopkins’s 45 stage I, treatment-naive samples across eight cancer types versus healthy controls (Figure 6J). Decontaminated fungal species provided notable performance, and multi-domain features matched or exceeded published fragmentomic approaches (average AUROCs: 94%–96%; Figure 6J). ML across individual stages continued this pattern (Data S6.2B), with AUROCs not significantly varying across stages for any feature set (Data S6.2C) or area under precision-recall curves (AUPRs) for multi-domain feature sets (Data S6.2D). These data suggest stage-invariant performance of microbial-augmented liquid biopsies.

Hopkins pan-cancer versus healthy ML analyses revealed that the top 20 ranked, decontaminated fungal species (9.6% of total) performed at least as well as all 209 decontaminated fungi (Data S6.2E; Table S8.2). This reduced signature performed similarly to all decontaminated fungi in the Hopkins cohort when examining individual cancer types (Figure 6I), stages (Data S6.2B), and negative controls (Data S6.1D). These 20 fungi also strongly discriminated among batch-corrected, pan-cancer TCGA blood samples (AUROC 95% CI: [87.76, 89.79]%; Data S6.2F), collectively affirming a pan-cancer plasma mycobiome signature.

We then reprocessed all 169 plasma samples from the UCSD cohort, which tested different experimental methods (fragmented versus unfragmented DNA), patient types (treated versus treatment-naive), and cancer types than the Hopkins cohort (1 of 8 Hopkins cancer types overlapped with UCSD). Although these differences limited direct comparisons, we tested whether the Hopkins 20-fungi signature provided similar healthy-versus-cancer performance, which it did (average AUROCs: 80%–86%; Figure 6K). The Hopkins 20-fungi signature performed similarly to the full set of UCSD decontaminated fungi in pan-cancer versus healthy (Data S6.2G) or per-cancer-type versus healthy comparisons (Data S6.2H), demonstrating its generalizability. Comparing performances with this signature or all decontaminated fungi in the UCSD cohort to negative controls revealed expected results (Data S6.3A). Log-ratios of TCGA-derived mycotype fungi did not significantly vary among UCSD cancer types (data not shown), potentially due to treatment status, but ML between cancer types showed detectable differences (Data S6.3B). Like the Hopkins cohort, the F1/F3 fungal log-ratio was significantly higher in cancer versus healthy samples (Data S6.3C), highlighting their potential clinical utility. Exploratory analyses of immunotherapy response information on UCSD cohort patients also revealed that WIS-overlapping fungi moderately discriminated responders from non-responders in melanoma (Data S6.3D) but not lung cancer (data not shown), although this remains to be validated in other cohorts. Overall, analyses across two independent cohorts and 10 cancer types show the utility of multi-domain cancer diagnostics and the plasma mycobiome, with a 20-fungi signature potentially able to distinguish pan-cancer versus healthy individuals.

## Discussion

We characterized the mycobiomes of 17,401 tissue and blood samples in four independent cohorts across 35 cancer types with complementary strategies. The study revealed cancer-type-specific fungal ecologies with lower diversities and abundances than matched bacteriomes; however, although fungi were detected in all examined cancer types, not all individual tumors were found positive for fungal signal. Imaging showed most fungi to be intracellular within cancer and immune cells, analogous to intratumoral bacteria (Nejman et al., 2020). We also found significant correlations between specific fungi and age, tumor subtypes, smoking status, response to immunotherapy, and survival measures. Whether these fungi are correlated or causally associated is yet to be determined. Interestingly, we found *Malassezia globosa*, which promotes pancreatic oncogenesis (Alam et al., 2022; Aykut et al., 2019), correlated to shorter OS in breast cancer. Although fungal pancreatic oncogenesis occurs via complement cascade activation and IL-33 secretion, little is known about fungal functional repertoires in other cancers. Functional characterization remains difficult due to low fungal abundances and a paucity of published fungal genomes that limit gene content inference from amplicon data, as possible for bacteria (Douglas et al., 2020).

To infer potential effects of fungi in tumors, we examined mycobiome-bacteriome-immunome interactions. We identified fungal-driven, pan-cancer “mycotypes” with distinct immune responses that stratified patient survival. Although our data do not establish causal relationships behind these clusters, they suggest that fungi are sparse but immunologically potent, analogous to PD1^+^ cells in immunotherapy (Kather et al., 2018). The associations of fungi with clinical parameters including OS in breast cancer, PFS in ovarian cancer, immunotherapy response in melanoma tumors, and detection of early-stage cancers support their clinical utility as potential biomarkers and therapeutic targets.

We observed strong positive correlations between fungal and bacterial diversities, abundances, and co-occurrences across several cancer types, suggesting tumor microenvironments (TMEs) may be non-competitive spaces for multi-domain microbial colonization, which we term a “permissive” phenotype. This differs from the gut, especially under anti-cancer or antibiotic therapies, where fungal and bacterial populations alternate and compete over shared resources—an “antagonistic” phenotype (Seelbinder et al., 2020; Shiao et al., 2021). It remains unclear whether a permissive phenotype is passively allowed by immunosuppressed, nutrient-rich TMEs (Hinshaw and Shevde, 2019) or denotes active synergy for greater ecosystem multifunctionality (Wagg et al., 2019) or a selection advantage for tumors (Aykut et al., 2019; Geller et al., 2017; Pushalkar et al., 2018). Mechanism(s) notwithstanding, the presence of spatially heterogeneous, intracellular, and polymicrobial communities in tumors motivates exploring cancer clonal evolution as a multi-species process with joint (e.g., TME nutrient limitations) and disjoint (e.g., antibiotic) selection pressures affecting fungal-bacterial-cancer-immune cell compositions (Sepich-Poore et al., 2022).

We provide the first analysis of plasma mycobiomes in treatment-naive, early-stage cancers, with stage-invariant diagnostic performance from multi-domain biomarkers that exceeds host-centric fragmentomics (Cristiano et al., 2019). Although sources of cell-free plasma-derived fungal or bacterial DNA remain unknown (Sepich-Poore et al., 2021), tumor-derived WIS species provided similar performance to a multi-domain database 26-fold larger, suggesting significant tumor origins.

### Limitations of the study

Our study has several caveats. Despite the relatively large number of samples per cohort, the strength of cancer type separation by mycobial data varied somewhat by cohort. We also note that low alpha diversity within samples and high beta diversity between samples of the same cancer type (Figures 3A, 3C, and 3D) yielded a high variation component in the data that required thousands of samples to obtain robust signals of cancer-specific mycobiomes. Thus, we have often displayed primary results as averages across samples within cancer types (Figures 3F and 3G) and positioned per-sample distributions in the supplement (Data S3.2B–S3.2F and S3.2I).

In TCGA data analysis, data aggregation across samples was needed to achieve high fungal genome coverages. The low per-sample genome coverages of fungi indicated fungal undersampling in the available TCGA data. Intersecting datasets increased confidence of fungal calls, but low coverage species could be impacted by false assignments to mobile genetic elements. More work is needed on the technical impact of mobile elements in characterizing microbiome communities using WGS technologies. The assembly of fungal genomes per cancer type identified fungal genomic bins that were phylogenetically concordant to our taxonomy data, but the size and complexity of fungal genomes resulted in relatively incomplete and possibly mixed fungal bins (Table S8.3).

The low fungal biomass in our samples presented many challenges. Although we have utilized multiple methods to control for possible contaminations, including extraction and paraffin controls and bioinformatic decontamination, we cannot rule out all false-positive results. The low biomass and bioinformatic challenges further precluded functional characterization of the tumor mycobiome. Our work also did not include truly normal tissues, excluding breast, limiting conclusions around tumor fungal origins, including whether tumor fungi derive from such surrounding tissues or vice versa, or the broad characterization of normal tissue mycobiomes—an effort complicated by ethical and acquisition difficulties of normal human tissues. Ideally, future studies will validate these findings in geographically diverse cohorts with matched, truly normal tissues in addition to tumor and NAT samples.

Additionally, although four different staining methods revealed intratumoral fungal presence and tumor-specific localization patterns, they proved challenging, with differing sensitivities and specificities across cancer types. Indeed, as all of these staining methods can only detect a subset of the fungal kingdom, we expect them to have a relatively high false-negative rate. Moreover, although negative controls for each of these methods were used, some false-positive results are inevitable.

Finally, although our study broadens the cancer microbiome landscape, our findings do not establish causality. Nonetheless, this first pan-cancer mycobiome atlas informs future study directions while characterizing a new layer of information for cancer diagnostics and therapeutics for the benefit of patients worldwide.

## STAR★Methods

### Key resources table

**Table:** 

REAGENT or RESOURCE	SOURCE	IDENTIFIER
**Antibodies**		

Mouse monoclonal anti-1-3 β-glucan	abcam	Cat#ab233743; RRID: AB_2923478
Rabbit polyclonal anti-Aspergillus	abcam	Cat#ab20419; RRID: AB_445571
Mouse monoclonal anti-CD45	Thermo Fisher Scientific	Cat# 14-0459-82; RRID: AB_467274
Mouse monoclonal anti-CD68	Thermo Fisher Scientific	Cat# MA5-12407; RRID: AB_10979558

**Biological samples**		

Ovarian cancer TMA	US Biomax	Cat#OV8010a
Melanoma TMA	US Biomax	Cat#ME804a
Lung cancer TMA	US Biomax	Cat#LC819a
Lung cancer TMA	US Biomax	Cat#LC813b
Breast cancer TMA	US Biomax	Cat#BR1191
Breast cancer TMA	US Biomax	Cat#BC08118a
Pancreatic cancer TMA	US Biomax	Cat#PA961f
Pancreatic cancer TMA	US Biomax	Cat#PA804b
Fungus 4-Tissue Artificial TMA Aspergillus, Candida, Histoplasma and Negative Control	BioSB	Cat#BSB-0335-CS
The resource of the WIS samples cohort was constructed at the Weizmann Institute of Science. All details are in Table S3.	Nejman et al., 2020	N/A

**Critical commercial assays**		

Phusion Hot Start II DNA Polymerase	Thermo Fisher Scientific	Cat# F549L
Qiaquick PCR purification kit	Qiagen	Cat#28104
Agencourt AMPure XP beads	Beckman Coulter	Cat#A63881
MiSeq Reagent Kit v3 (600-cycle)	Illumina	Cat#MS-102-3003
KAPA SYBR FAST qPCR Master Mix (2X) ABI Prism	Kapa Biosystems	Cat#KK4605
Gomori Methenamine-Silver (GMS) Nitrate Stain Kit	abcam	Cat#ab150671
Bond polymer refine detection kit	Leica Biosystems	Cat#DS9800
BOND epitope retrieval solution 1	Leica Biosystems	Cat#AR9961

**Deposited data**		

Raw data from WIS cohort	This paper	PRJNA786764

**Datasets**		

TCGA CIBERSORT immune cell abundances and linked survival data	Thorsson et al., 2018	Table S1
TCGA WGS and RNA-Seq raw data and metadata (processed and/or downloaded from Cancer Genomics Cloud)	Lau et al., 2017	Cancer Genomics Cloud
Hopkins plasma WGS raw data	Cristiano et al., 2019	EGAD00001005339
UCSD plasma WGS raw data	Poore et al., 2020	ENA: ERP119598 (HIV-negative controls); ERP119596 (prostate cancer); ERP119597 (lung cancer and melanoma)

**Experimental models: Cell lines**		

HS-5 human fibroblast cell line	ATCC	Cat# CRL-11882

**Oligonucleotides**		

Forward primer for 1^st^ ITS2 PCR: ITS86F 5’-GTGAATCATCGAATCTTTGAA-3’	Turenne et al., 1999	N/A
Reverse primer for 1^st^ ITS2 PCR: ITS4+rd2 Illumina adaptor 5’-AGACGTGTGCTCTTCCGATCT - TCCTCCGCTTATTGATATGC-3’	ITS4 from White et al., (1990)	N/A
Forward primer for 2^nd^ ITS2 PCR: P5-rd1-ITS86F 5’ - AATGATACGGCGACCACCGAGATCT - ACACTCTTTCCCTACACGACGCTCTTCCGATCT - GTGAATCATCGAATCTTTGAA-3’	ITS86F from Turenne et al., (1999)	N/A
Reverse primer for 2^nd^ ITS2 PCR: 5’- CAAGCAGAAGACGGCATACGAGAT - NNNNNNNN - GTGACTGGAGTTCAGAC GTGTGCTCTTCCGATCT-3’	Nejman et al., 2020	N/A
Forward primer for 5.8S qPCR: ITS3 - 5’-GCATCGATGAAGAACGCAGC-3’	White et al., 1990	N/A
Reverse primer for 5.8S qPCR: ITS86R - 5’- TTCAAAGATTCGATGATTCAC-3’	Turenne et al., 1999	N/A
Probe for 28S fungal FISH: D-205: 5’- ATTCCCAAACAACTCGAC-3’	Inácio et al., 2003	N/A
Probe for 28S fungal FISH: D-223: 5’-CCACCCACTTAGAGCTGC-3’	Inácio et al., 2003	N/A
Probe for 28S fungal FISH: D-260: 5’-TCGGTCTCTCGCCAATATT-3’	Inácio et al., 2003	N/A

**Software and algorithms**		

Python version 3.6	Python Software Foundation	https://www.python.org/
Qiita cloud-enabled microbiome analyses	Gonzalez et al., 2018	https://qiita.ucsd.edu/
Woltka alignment-based taxonomy classification (used by Qiita)	Zhu et al., 2022	https://github.com/qiyunzhu/woltka
ITS2 classification pipeline	This paper	https://github.com/microbiofunc/ITS2-pipeline
Dockerized host depletion pipeline	This paper	https://github.com/knightlab-analyses/mycobiome/tree/master/Docker_host_depletion_pipeline
Per-sample and aggregate genome coverage	Hakim et al., 2022	https://github.com/ucsd-cmi/zebra_filter
MMvec co-occurrence analyses	Morton et al., 2019a	https://github.com/biocore/mmvec
Robust Aitchison beta diversity	Martino et al., 2019	https://github.com/biocore/DEICODE
EMPeror PCoA visualizer	Vázquez-Baeza et al., 2013	https://github.com/biocore/emperor
EukDetect	Lind and Pollard, 2021	https://github.com/allind/EukDetect
metaSPAdes 3.13.1	Nurk et al., 2017	https://github.com/ablab/spades
MaxBin2 2.2.4	Wu et al., 2016	https://sourceforge.net/projects/maxbin2/
MetaBAT2 2.12.1	Kang et al., 2019	https://bitbucket.org/berkeleylab/metabat
Concoct 1.0.0	Alneberg et al., 2014	https://github.com/BinPro/CONCOCT
BowTie2 2.2.3	Langmead and Salzberg, 2012	https://github.com/BenLangmead/bowtie2
SAMtools 0.1.19	Li et al., 2009	https://github.com/samtools/samtools
metaWRAP 1.1.2)	Uritskiy et al., 2018	https://github.com/bxlab/metaWRAP
CheckM (v. 1.0.13)	Parks et al., 2015	https://github.com/Ecogenomics/CheckM
EukCC	Saary et al., 2020	https://github.com/Finn-Lab/EukCC
BUSCO v5.1.2	Simão et al., 2015	https://busco.ezlab.org/
Mfannot	N/A	https://github.com/BFL-lab/Mfannot
PEAR version 0.9.10	Zhang et al., 2014	https://cme.h-its.org/exelixis/web/software/pear/
cutadapt version 1.17	Martin, 2011	https://cutadapt.readthedocs.io/en/v1.17/
QIIME 2 version 2018.8	Bolyen et al., 2019	https://qiime2.org/
ITSx version 1.1b1	(Bengtsson-Palme and Ryberg, 2013	https://microbiology.se/software/itsx/
R version 4.03	R CRAN	www.r-project.org
phyloseq 1.34.0	McMurdie and Holmes, 2013	https://bioconductor.org/packages/release/bioc/html/phyloseq.html
caret 6.0-90	Kuhn, 2008	https://topepo.github.io/caret/
PRROC 1.3.1	Grau et al., 2015	https://cran.r-project.org/web/packages/PRROC/index.html
gbm 2.1.8	Ridgeway, 2007	https://cran.r-project.org/web/packages/gbm/gbm.pdf
xgboost 1.5.0.1	Chen et al., 2015	https://xgboost.readthedocs.io/en/stable/R-package/xgboostPresentation.html
randomForest 4.6-14	Liaw and Wiener, 2002	https://cran.r-project.org/web/packages/randomForest/randomForest.pdf
ANCOM-BC 1.4.0	Lin and Peddada, 2020	https://github.com/FrederickHuangLin/ANCOMBC
decontam 1.14.0	Davis et al., 2018	https://github.com/benjjneb/decontam
limma-voom 3.50.0	Law et al., 2014	https://bioconductor.org/packages/release/bioc/html/limma.html
snm 1.42.0	Mecham et al., 2010	https://www.bioconductor.org/packages/release/bioc/html/snm.html
MATLAB version 2019b with the Statistics and Machine Learning Toolbox	MathWorks	https://www.mathworks.com
Cytoscape 3.8.1	Shannon et al., 2003	https://cytoscape.org/
UNITE database (version 8, dynamic, sh_taxonomy_qiime_ver8_dynamic_04.02.2020.txt)	Nilsson et al., 2019	https://unite.ut.ee/repository.php
scikit-bio 0.5.6	N/A	https://github.com/biocore/scikit-bio

**Other**		

Pannoramic SCAN II automated slide scanner	3D HISTECH	N/A

### Resource availability

#### Lead contact

Further information and requests for resources and reagents should be directed to and will be fulfilled by the lead contact, Ravid Straussman (Ravidst@weizmann.ac.il).

#### Materials availability

This study did not generate new unique reagents.

### Method details

#### WIS cohort: Sample collection

The samples of the WIS cohort were collected from nine medical centers, and we previously reported their DNA extraction as well bacterial characterization (Nejman et al., 2020). For ITS2 profiling, 1183 samples of this original cohort were used (Figure 1A; Table S1). Samples include tumor, normal adjacent tissue (NAT) and normal tissue from eight tumor types for a total of 12 conditions (condition is defined by the tissue type and the tumor/NAT/normal status) (Table S1). Samples included both formalin fixed paraffin embedded (FFPE) and snap frozen samples. To account for potential contamination by fungi or fungal DNA during sample handling and processing, our cohort also included two types of negative controls: 104 paraffin-only controls which were made by sampling paraffin only (without tissue) from the study FFPE blocks and 191 DNA-extraction negative controls in which only sterile DDW was introduced at the beginning of the DNA-extraction pipeline. These controls enabled detection of potential fungal contaminants and delineation of signal versus noise allowing for appropriate processing of the data prior to analysis (see below). Note that matching bacterial data of the same samples that were used in this study, was generated by us and published in Nejman *et al*. (Nejman et al., 2020). Also note that all handling of the samples (including DNA extraction and PCR set up) was done in a clean dedicated pre-PCR room to avoid potential contamination of amplified PCR products from the main lab.

#### TCGA cohort: Data accession

All TCGA sequence data were accessed via the Cancer Genomics Cloud (CGC) as sponsored by SevenBridges (https://cgc.sbgenomics.com) (Lau et al., 2017) after obtaining data access from the TCGA Data Access Committee through dbGaP (https://dbgap.ncbi.nlm.nih.gov/aa/wga.cgi?page=login). Details of how TCGA samples were acquired and processed are comprehensively described elsewhere (Hoadley et al., 2018), and SOPs for TCGA sample processing are available in the NCI Biospecimen Research Database (https://brd.nci.nih.gov/brd/sop-compendium/show/701). Metadata for these patients were previously published and originally compiled using SevenBridges’s metadata ontology (Poore et al., 2020).

#### Hopkins and UCSD cohorts: Data accessions

Raw BAM files for the Hopkins plasma cohort were accessed through the European Genome-Phenome Archive (EGA) under Study ID EGAS00001003611 with prior data access approval. These files were previously analyzed for host-centric, fragmentomic cancer diagnostics by Cristiano *et al*. (Cristiano et al., 2019). Raw BAM files for the UCSD cohort were internally available after Poore and Kopylova *et al*. (Poore et al., 2020) previously published them using bacterial-centric analyses, and host-depleted versions of the files are publicly available on European Nucleotide Archive (ENA) with the following accession IDs: ERP119598 (UCSD HIV-negative controls), ERP119596 (UCSD prostate cancer), and ERP119597 (UCSD lung cancer and melanoma).

#### WIS cohort: ITS2 amplification and sequencing

ITS2 sequencing was used for fungal identification in all samples. In order to avoid batch effects as much as possible, samples from different cancer types and medical centers were randomized between different batches of DNA extraction, PCR amplification, and sequencing runs (Table S2.3). PCR was performed on 100ng of DNA per sample (or the maximum available). For extraction controls a volume of 5μl per sample was used, and for empty paraffin controls a volume equal to the volume taken for the matching sample was used. Forward primer ITS86F 5’-GTGAATCATCGAATCTTTGAA-3’ (Turenne et al., 1999) and reverse primer ITS4 (White et al., 1990) with rd2 Illumina adaptor 5’-AGACGTGTGCTCTTCCGATCT - TCCTCCGCTTATTGATATGC-3’ were used for the first PCR amplification. PCR mix per sample contained 5ul sample DNA, 0.2μM per primer (primers purchased from Sigma), 0.02 unit/μl of Phusion Hot Start II DNA Polymerase (Thermo Scientific F549), 10μl of X5 Phusion HS HF buffer, 0.2mM dNTPs (Larova GmbH), 31.5ul ultra pure water, for a total reaction volume of 50μl. PCR conditions used were 98°C 2min, (98°C 10 sec, 55°C 15 sec, 72°C 35 sec) X 35, 72°C 5 min. A second PCR was performed to attach Illumina adaptors and barcode per sample for 6 additional cycles. Samples from 1^st^ PCR were diluted 10 fold and added to the PCR mix as described above. Primers of second PCR included: forward primer P5-rd1-ITS86F 5’ - AATGATACGGCGACCACCGAGATCT - ACACTCTTTCCCTACACGACGCTCTTCCGATCT - GTGAATCATCGAATCTTTGAA-3’, and reverse primer 5’- CAAGCAGAAGACGGCATACGAGAT - NNNNNNNN - GTGACTGGAGTTCAGACGTGTGCTCTTCCGATCT-3’ (Nejman et al., 2020). Every 96 samples were combined for a single mix by adding 14μl from each. Before mixing, an aliquot from each of the samples was run on an agarose gel. In cases where the amplified bands were very strong, samples were diluted between 5 and 20-fold before they were added to the mix (Table S2.3). Each sample mix was cleaned with QIAquick PCR purification kit (QIAGEN, catalog # 28104). Four cleaned sample mixes were then combined into a single mix of 384 samples, and size selection was performed with Agencourt AMPure XP beads (Beckman Coulter #A63881) to remove any excess primers. Beads to sample ratio was 0.85 to 1. Samples were then run on the Miseq v3 600 cycles paired-end with 30% PhiX. Overall, six runs of Miseq were performed for this study.

#### TCGA, Hopkins, and UCSD cohorts: Library preparation and sequencing

Library preparation and sequencing methods of TCGA were described in detail by Hoadley *et al*. (Hoadley et al., 2018), and primarily employed QIAGEN products for multi-analyte (DNA, RNA) extraction and Illumina platform sequencing. Sample processing and sequencing of the Hopkins cohort was described in detail by Cristiano *et al*. (Cristiano et al., 2019), and, briefly, performed cell-free plasma DNA extraction using the Qiagen Circulating Nucleic Acids Kit, non-fragmented library preparation using a modified protocol of the NEBNext DNA Library Prep Kit for Illumina, and sequencing with 100-bp paired-end runs on the Illumina HiSeq machines (Cristiano et al., 2019). Sample processing and sequencing of the UCSD cohort was described in detail by Poore and Kopylova *et al*. (Poore et al., 2020), and, briefly, performed cell-free DNA extraction using the Qiagen Circulating Nucleic Acids Kit, library preparation using the KAPA HyperPlus Kit (Kapa Biosystems), and paired-end 2×150-bp sequencing on an Illumina NovaSeq 6000 instrument (S4 flow cell).

#### WIS cohort: ITS2 sequencing analysis

##### ITS2 read classification pipeline

The ITS2 classification pipeline was built with Python 3.6. For each sequencing library, paired end reads were joined using PEAR (version 0.9.10) followed by filtering of merged reads by minimum length of 80bp and trimming of primers from both ends with cutadapt (version 1.17). Note, that after flooring, the minimum length of the remaining amplicons was 143bp. Within the QIIME 2 environment (version 2018.8), Dada2 was used to create amplicon sequence variants (ASVs), then ITSx (version 1.1b1) was used to delineate ASVs to ITS2 regions (removing preceding 5.8S and trailing 28S sequences). A taxonomic naive bayesian classifier in QIIME 2 (Bolyen et al., 2019) was trained on the UNITE database (version 8, dynamic, sh_taxonomy_qiime_ver8_dynamic_04.02.2020.txt) and used to classify the processed ASVs. ASVs were filtered by the ITSx and UNITE classifications to include fungal reads only. Any ASVs that were classified by ITSx as fungi were included in the downstream analysis. Any ASVs that were classified by ITSx as non-fungal, were included in the downstream analysis only if their classification as fungi reached a class or lower phylogenetic level by UNITE and were validated by NCBI BLAST to be fungal. Seventy percent of ASV reads that were included in the downstream analysis were classified to species level (Data S1.5A). The other 30% of ASV reads were classified to higher taxonomic levels. To make sure that no human reads were misaligned as fungal reads, we tried to align all ASVs to the human genome or to the human rDNA sequences, but none of the ASVs aligned to those human sequences. Specifically, all 1191 ASVs in Table S2.2, with and without flanking primer regions, were aligned to the human genome (hg19) and human ribosomal region (RNA45SN1, NR_145819.1) using Bowtie2 with –end-to-end and –sensitive parameters to increase local alignment sensitivity. In addition, all ASVs found in the mock human cell line samples were also mapped to the human genome. Neither of these produced successful alignments. Any ASV that was classified as fungus by the naive bayesian classifier against the UNITE database, but wasn’t classified down to species level, we also BLASTed against the entire nr/nt database with default parameters, which only resulted in significant alignments to fungal sequences. 76 ASVs which were classified as fungal by the UNITE classifier, but by ITSx as ‘T’ for Tracheophyta, were also BLASTed and found to be aligned to fungal sequences.

##### WIS cohort negative controls

The ITS2 dataset includes two types of negative controls (Table S1): (1) 191 DNA extraction controls performed on empty tubes (with DDW only) in parallel to sample DNA extraction, and (2) 104 paraffin controls which were made by sampling paraffin only (without tissue) from a subset of the study FFPE blocks. The 295 negative controls allowed for a better understanding of the fungal signal in the tissues versus technical background noise (i.e., index hopping) as can be detected in the negative control samples. The histogram of the number of reads per ASV per sample as well as the number of reads per sample (Data S1.6A and S1.6B) both presented a bimodal distribution with the peaks found on either side of 1000 reads/ASV or 1000 reads/sample. We found that the chance of an ASV to have more than 1000 reads was 3 times higher in samples vs controls (21.6% vs 7.1%). We therefore floored the data in a sample-specific manner, such that if an ASV was assigned less than 1000 reads in a specific sample, its assigned reads were converted to zero.

##### ITS2 data normalization

Next, we introduced two types of data normalization: (1) Library normalization, where samples were normalized to account for the difference in the average number of reads/sample per library. A factor was assigned to each of the six sequencing libraries to reflect the fold change of the mean number of reads/sample in the library as compared to the mean number of reads/sample in all samples across all six libraries. Then the number of reads for each ASV in each sample was corrected by this factor. (2) Dilution normalization: as a subset of the samples were diluted before sequencing based on the amplification levels as detected on agarose gel (see above), their ASV reads were multiplied by the dilution factor per sample to reflect their true original load. Table S2.2 presents the number of reads per ASV per sample after both data flooring and normalization.

Next, ASVs were aggregated based on UNITE classification, to species level when possible. ASVs that could not be classified to species level, were grouped together by the lowest known phylogenetic level and labeled “Other”. Lastly, data were aggregated by summing all reads in each taxonomic level by the associated taxa in the level above it (Table S2.3).

##### WIS cohort decontamination

The negative control samples were then used to flag potential contaminant species (Data S1.6C). Out of 456 species detected in the data (after flooring and normalization), 13 species unique to the negative control samples were removed from the dataset (Data S1.6D). For an additional 63 species that were detected in both negative control samples and true samples, statistical testing was applied: (1) Fisher’s exact test (on the floored normalized data converted to present/absent) was applied to check if a species was more prevalent in a specific condition (tissue+tumor/NAT/normal) versus the 191 extraction control samples. (2) Wilcoxon test (on the number of reads, without flooring, with library and dilution factor normalization) was applied to check if a species was more abundant in a specific condition (tissue+tumor/NAT/normal) versus the 191 extraction control samples. A species that had a p-value≤0.05 and FDR≤0.2 in at least one of these tests passed this filtering step for the condition. Next, the same tests were performed against the 104 paraffin control samples. Forty-two species (out of the 63 that were tested) passed both filtering steps in at least one condition. All of these 42 species, as well as the 380 species that did not appear in any of the 295 controls were considered part of the ‘fungal world’ that was used for all downstream analysis. The same filtering steps were also performed for each of the taxonomic levels (Table S2.4). In addition, we applied the same filtering strategy to the original 1191 ASVs (after flooring and normalization), yet this approach was more permissive, letting more reads through the filters: 4,138,346 reads (2.67%) removed at ASV level versus 4,295,957 reads (2.77%) removed after decontaminating at species level. This difference was largely due to the removal at the species level being more stringent in removing all reads from all ASVs classified to a contaminating species, which stems from the natural variation of ITS2 sequences across multiple copies of the rDNA segment within the same fungal cell/species. Therefore, this ASV-level decontamination approach was not further used. Note that only fungi and bacteria that passed the filtering steps in at least one of the tumor types were included in most of the analysis in this work (exceptions are described in figure legends).

##### WIS cohort clinical utility of cancer mycobiomes

In Figures 6A, 6B, 6E, and 6G, p-values were calculated by Fisher’s exact test. Only fungi that appeared in ≥5% and at least twice in one of the groups were included in the analysis. All fungi in these plots had FDR corrected values of ≤0.2. Data used was after flooring and normalization as described above. Only fungi that passed the filtering step in at least one of the tumor types were included in the analysis.

#### WIS cohort: ITS2 Pipeline validation and testing

A mock community of 17 fungal species was generated to validate the ITS2 experimental procedure and assess the success of detecting different fungi (Data S1.6E). DNA from all fungi was extracted using MasterPure Yeast DNA Purification Kit (Epicentre, MPY 80200). Equal amounts of DNA from each of the fungal species were mixed together and then 0.00032ng DNA of the mix was spiked into 100ng of human DNA (extracted from the HS-5 human fibroblast cell line (ATCC# CRL-11882)). ITS2 amplification and sequencing was done as described above. Fourteen out of the 17 species were detected (Data S1.6E). One of the species that was not detected (*Flavodon flavus*) was wrongly classified to a different family in the same order (Polyporales). Overall, 99.89% of the reads belonged to the species included in the mock. We repeated the ITS2 amplification and sequencing two more times and reached almost identical results, detecting the same fungal species (data not shown).

To assess the reproducibility of our technical and computational pipeline we repeated the ITS2 amplification and sequencing three times, for 88 human tumor or NAT samples. For 82 samples that passed our quality control, we compared the Bray-curtis dissimilarity scores between all pairs that belong to the same original sample versus all pairs that belong to different samples within the same tissue type. We found that the dissimilarity was significantly lower between repeats relative to between samples from the same tissue (p-value<2.22×10^-16^) (Data S1.6F). Only one sequencing result from each triplicate (the one with the highest amount of reads) was used for all other analyses that were subsequently done.

#### TCGA, Hopkins, and UCSD cohorts: Bioinformatic processing

##### Determining read counts in TCGA

Total and mapped read counts were calculated using SAMtools’s idxstats function (v. 1.11) (Li et al., 2009), which was wrapped in a Docker container and applied to all available TCGA BAM files on the CGC as an “app.” The app was then run in parallel across files using Amazon Web Services (AWS) as a backend using 8 cores per file. Total read counts were extracted from the resultant idxstats output files using *awk '{s+=$3+$4} END {print s}'* and mapped read counts were extracted using *awk '{s+=$3} END {print s}'.* Unmapped read counts were determined via the subtraction of mapped from total. Microbial read counts were derived by summing all genome-level microbial hits against the “rep200” database (see below for more details). Similarly, fungal-specific or bacterial-specific counts were determined by summing all genome-level microbial hits against the rep200 database within those domains.

##### Host depletion of WGS and RNA-Seq data

Previous efforts to mine host genomic or transcriptomic information for microbial nucleic acids relied on extracting unaligned, “non-human” reads from pre-aligned BAM files, followed by mapping those reads against a database of microbial genomes (Poore et al., 2020) Since TCGA samples were collected during a decade (2006-2016), the human genome references used for BAM file generation changed over time, and uniform realignments were not performed until very recently (ICGC/TCGA Pan-Cancer Analysis of Whole Genomes Consortium, 2020). Although this was not detected to be a problem by Poore and Kopylova *et al*. (Poore et al., 2020) for bacterial-centric analyses, we wanted to uniformly host deplete and further quality control all TCGA files prior to multi-domain mapping and metagenome assembly. Thus we designed, optimized (for speed), and Dockerized a uniform host depletion pipeline using a combination of SAMtools (v. 1.11) (Li et al., 2009), Minimap2 (v. 2.17-r941) (Li, 2018), and fastp (Chen et al., 2018) capable of being run on any high performance compute system. The scripts necessary for creating this host-depletion Docker container and running it on new samples are on the TCGA-associated mycobiome GitHub link (https://github.com/knightlab-analyses/mycobiome#docker-host-depletion-pipeline-docker_host_depletion_pipeline). The exact one-line piped command being run within the Docker container is also explicitly shown below.

Read pairs were subsequently discarded if either mate mapped to the GRCh38.p7 human genome (https://www.ncbi.nlm.nih.gov/assembly/GCF_000001405.33/) or the Phi X 174 viral genome. Reads were also discarded if less than 45 base pairs in length or if they exhibited poor base quality (using fastp default parameters). Specifically, the following command was run within the Docker container to host deplete, where $cpus and $db denote the number of compute cores and a precomputed Minimap2 reference database (as a.mmi file), respectively:

*samtools view -f 4 -O BAM $in_dir/$filename |*

*samtools bam2fq - |*

*fastp -l 45 --stdin -w $cpus --stdout --interleaved_in |*

*minimap2 -ax sr -t $cpus $db - |*

*samtools fastq -@ $cpus -f 12 -F 256 - -1 $out_dir/$base_name.R1.trimmed.fastq.gz -2 $out_dir/$base_name.R2.trimmed.fastq.gz*.

The final line outputs host-depleted forward (“R1”) and reverse (“R2”) fastq files. Sometimes, due to cloud computing constraints, the first line of the command (samtools -f 4) was done separately from the remaining lines, which were consistently run together. Typical compute time per file for the host depletion and read extraction ranged from several minutes to a few hours, depending on the file size, using 8-16 cores and ∼100 GB of RAM.

We note that this additional, uniform host depletion reduced the number of total files available for the TCGA mycobiome analysis when files resulted in 0 non-human reads. Specifically, 77 WGS files and 2530 RNA-Seq files had 0 non-human reads after additional host depletion and could not be used for shotgun metagenomic or metatranscriptomic microbial assignments. This dropout of ∼15% of the total TCGA cohort indicates that there is no “baseline” of bacterial, fungal, or other microbial read percentages, as these 2,607 samples have 0 such reads, and the plots showing microbial read percentages (e.g., Figure 1C) only reflect those samples with non-zero read counts. Another 16 files repeatedly failed the host depletion pipeline and could not be used. Overall, this reduced the number of files available for the TCGA mycobiome analysis compared to our previous bacteriome-centric analysis (Poore et al., 2020).

##### Shotgun metagenomic and metatranscriptomic microbial assignments

Host depleted and quality controlled output fastq files were then uploaded to Qiita web server (Gonzalez et al., 2018) for per-sample metagenomic or metatranscriptomic microbial classification. Qiita offers a graphical user interface that facilitates shotgun metatranscriptomic and/or metagenomic analyses using direct genome alignments based on Woltka v0.1.1 (https://github.com/qiyunzhu/woltka) (Zhu et al., 2022) against Qiita’s concomitant “rep200” multi-domain database. “Rep200” corresponds to RefSeq release 200 (built as of May 14, 2020), which comprises 11,955 genomes with the following taxa numbers and domains: 419 archaea; 11,080 bacteria; 320 fungi; 88 protozoa; 48 viruses (https://qiita.ucsd.edu/static/doc/html/processingdata/processing-recommendations.html#reference-databases). We note that the only other database used for Qiita metagenomics or metatranscriptomics (Web of Life, WoL) does not include fungi and was not used for this work. Direct genome alignments against rep200 were run using Bowtie2 v2.4.1 (Langmead and Salzberg, 2012) as the backend. This process is equivalent to a Bowtie2 run with the following parameters:

*--very-sensitive -k 16 --np 1 --mp “1,1” --rdg “0,1” --rfg “0,1” --score-min “L,0,-0.05”*

The sequence alignment is treated as a mapping from queries (sequencing data) to subjects (microbial reference genomes). Reads mapped to a microbial reference genome are counted as hits, such that the resultant feature table comprises samples (rows) by microbial genome IDs (columns) and concomitant abundances. These microbial genome IDs (named “operational genomic units” or OGUs) provide a shotgun metagenomic equivalent to ASVs in 16S rRNA gene amplicon sequencing data (Zhu et al., 2022). Of note, in the case that one sequence is mapped to multiple genomes by Bowtie2 (up to 16), each genome is counted 1 / k times, where k is the number of genomes to which this sequence is mapped. The frequencies of individual genomes were then summed after the entire alignment was processed, and rounded to the nearest even integer, thereby making the sum of OGU frequencies per sample nearly equal (considering rounding) to the number of aligned sequences in the dataset. The resultant count matrix was saved as a biom file for downstream analyses. This process was repeated for the TCGA, Hopkins, and UCSD cohorts, with separate Qiita projects under the following study IDs: 13722 (TCGA WGS), 13767 (TCGA RNA-Seq), 13984 (Hopkins), 12667 (UCSD HIV-free controls), 12691 (UCSD prostate cancer), 12692 (UCSD lung cancer and melanoma).

##### Running EukDetect on TCGA samples

To verify fungal taxonomic matches, we used EukDetect version 1.0.1 (Lind and Pollard, 2021) against their database built on 1/23/2021 (https://figshare.com/articles/dataset/Eukdetect_database/12670856/6) and followed their standard processing pipeline (https://github.com/allind/EukDetect). In summary, host-depleted TCGA fastq files in Qiita were additionally filtered via fastp to discard sequences shorter than 75bp, since EukDetect requires 75bp minimum to operate and the host depletion pipeline only removed reads shorter than 45bp (see “Host depletion of WGS and RNA-Seq data” section above). The resulting per-sample fastq files were then grouped by Qiita preparation, and the average length of the first 2500 sequences of all the files in each preparation was used as the readlen parameter. EukDetect was then run using default settings, and the default filtered tables were extracted for downstream analyses. We note that EukDetect currently cannot provide accurate relative abundances (see https://github.com/allind/EukDetect/issues/11) because the calculated abundances are marker dependent (i.e., the more markers there are for a given fungus, the higher abundance it can have; also, differences in marker size and genome size exist). Thus, we only used the filtered fungal species outputted by EukDetect to intersect with and subset the TCGA fungal species as features for machine learning. We also noticed that high coverage fungi (≥1% aggregate genome coverage in TCGA) were much more likely to be detected by EukDetect (Fisher exact test: p=5.67×10^−^^11^, odds ratio=28.0) than low coverage fungi (<1% aggregate genome coverage in TCGA), suggesting; see below), suggesting that marker-based taxonomy classification methods may be limited in very low-biomass settings.

#### TCGA and WIS cohorts: Alpha and beta diversity calculations

##### A general note on employed beta diversity distance metrics

Three beta diversity distance metrics were evaluated in this study—Jaccard, Bray-Curtis, and RPCA—all of which showed significant cancer type variation via PERMANOVA. However, metagenomic and metatranscriptomic alignment read-splitting in TCGA can artificially influence Jaccard presence/absence measurements, prompting a focus on using Jaccard for the WIS cohort, which had decontamination informed by experimental negative controls, and Bray-Curtis for TCGA. Secondly, both Jaccard and Bray-Curtis require rarefaction and discarding of information to provide accurate comparisons between samples of varying library sizes, and fungal abundances in these cohorts were already low, motivating usage of a distance metric that did not require rarefaction. RPCA is an ideal distance metric for accomplishing this task without rarefaction, so we included it for both WIS and TCGA cohorts. Again, to be clear, statistical significance did not depend on which method was used, but the above logic guided why these metrics were each included.

##### TCGA alpha diversity calculations

Raw decontaminated fungal count data from primary tumors was subset to each TCGA WGS sequencing center and processed using QIIME 2 (version qiime2-2020.2) (Bolyen et al., 2019) to calculate richness and shannon alpha diversity per center. Rarefaction amounts were determined by the fungal read count distribution per TCGA sequencing center, and a common value of 5000 reads/sample was identified among 4 of the 5 WGS sequencing centers as being approximately the first quartile of reads/sample—Broad Institute WGS samples excepted, with 2000 reads/sample approximately representing its first quartile, which was used for rarefaction.

##### TCGA alpha diversity fungi-bacteria correlations

Multi-domain TCGA alpha diversity was calculated using the following procedure: (1) Subset to WGS primary tumor samples; (2) rarefy the entire WGS rep200 table to 115,000 reads/sample (approximately the first quartile of the read/sample distribution) using phyloseq (v. 1.38.0) (McMurdie and Holmes, 2013); (3) separate fungal and bacterial features into two separate tables; (4) calculate richness among both fungal and bacterial rarefied tables; (5) correlate, using Spearman correlations, the paired fungal and bacterial richnesses (Figure 3B). We also attempted this procedure with the modification that fungal and bacterial feature tables were independently rarefied, but we found that this version caused microbial richness to weakly but still significantly, positively correlate with the sample library sizes (data not shown), so it was discarded in favor of the ‘global’ table rarefaction.

##### TCGA beta diversity calculations

Given the limited fungal reads/sample in TCGA, we desired to perform beta diversity without rarefying using a method we previously published named robust Aitchison PCA (RPCA, also called DEICODE) (Martino et al., 2019). DEICODE has a QIIME 2 plugin (https://library.qiime2.org/plugins/deicode/19/) that was used on the raw decontaminated fungal count data in primary tumors subset to each TCGA WGS sequencing center with the following parameters: {--p-min-feature-count 10, --p-min-sample-count 500}. The resultant biplots were visualized using EMPeror (Vázquez-Baeza et al., 2013) and the QIIME 2 plugin for ADONIS (i.e., PERMANOVA) was used to estimate the significance and explained R^2^ of cancer type with the DEICODE Aitchison distance matrix.

Additionally, Bray-Curtis beta diversity distances, calculated through QIIME2 (Bolyen et al., 2019) on the batch corrected data proportions, were compared for WGS primary tumor samples, if having ≥10 samples per cancer type, within a TCGA cancer type to the distances between that cancer type and all others through a Mann-Whitney U test via Scipy (Virtanen et al., 2020) with an FDR multiple test correction across cancer types through statsmodels (Seabold and Perktold, 2010), as shown in Data S3.2G.

To compare tumor vs. NAT samples in TCGA, we performed the following analyses:

Analysis #1: (1) Rescale Voom-SNM batch corrected, decontaminated, species-level pan-cancer data into counts using a scalar of 10^4^; (2) calculate relative abundances using the batch corrected counts; (3) average fungal relative abundances across disease type-sample type groups (e.g., “Breast Invasive Carcinoma NAT”); (4) calculate Bray-Curtis dissimilarity on the averaged relative abundances; (5) plot using a principal coordinates analysis using cancer types also found in the Weizmann cohort and with at least 10 tumors and NATs available in TCGA (Data S3.2J).

Analysis #2: We performed a PERMANOVA analysis within each TCGA disease type for sample-type for both Aitchison and Bray-Curtis distances on the full sample set of relative abundances and found that no disease type significantly differs between tumor and NAT after accounting for multiple testing correction (Table S5).

#### TCGA, Hopkins, and UCSD cohorts: Decontamination

##### TCGA decontamination

Although TCGA protocols did not include contamination controls during the processing of their samples, we showed that *in silico* methods could be used to decontaminate the TCGA bacteriome (Poore et al., 2020). The fundamental principle of these methods is that consistent negative correlations exist for external (e.g., reagent, environmental) contaminating taxa between their read fractions and analyte (DNA or RNA) concentrations (Davis et al., 2018). A published tool named *decontam* (https://github.com/benjjneb/decontam) (version 1.14.0) (Davis et al., 2018) wraps the method into an R package and function based on two underlying mathematical assumptions: (i) the contaminants are added in uniform amounts across samples; and (ii) the amount of contaminant DNA or RNA is small relative to the true sample DNA or RNA (microbial or host). Since per-sample DNA and RNA concentrations are available in TCGA metadata, they can be used to indicate putative contaminating taxa. Importantly, though, our past analyses (Poore et al., 2020) demonstrated that too stringent of an *in silico* decontamination threshold actually removes flora known to be associated with a given body site (e.g., too stringent decontamination of NAT colon tissues in TCGA dissociates it from normally-associated fecal material). Additionally, there are difficulties of strict filtering with taxa that are known commensals and/or pathogens but also can be contaminants in certain contexts, even at the species level (e.g., *Malassezia restricta*, a skin fungus). Thus, in our mycobiome analyses, we sought a balance between strict filtering, allowance of known commensals/pathogens, inclusion of WIS-identified (this study) or HMP-identified (Nash et al., 2017) fungi, and inclusion of fungi of unknown significance that may be related to cancer biology.

Decontamination was thus broken into two steps: (i) Statistical decontamination via *decontam* using per-sample DNA or RNA concentrations and read fractions across plate-center batches (see below); and (ii) manual curation, comparison against WIS-identified and HMP-identified fungi, and literature review prior to making final determinations.

Step #1: TCGA sample identifiers (e.g., “TCGA-02-0001-01C-01D-0182-01”) denoted the sequencing center and plate within that center upon which the sample was run (for details, see https://docs.gdc.cancer.gov/Encyclopedia/pages/ TCGA_Barcode/). These barcodes were used to extract all sequencing plate-center combinations using the last two sets of integers (e.g., “0182-01” is plate 182 from center 1). We previously found the plate-center method to work well on TCGA bacterial data, as it removed many likely contaminants while retaining several known commensals and pathogens (Poore et al., 2020). Since *decontam* effectively performs a regression analysis to determine if a taxon is a contaminant, we required ≥10 samples per plate-center batch, retaining 329 total plate-center batches among samples positive for fungi. *Decontam* was then run in “frequency” mode, identifying putative contaminants using TCGA sample aliquot concentrations, a default P^∗^ stringency threshold of 0.1, and the default batch.combine=“minimum” parameter, such that a taxon was removed if identified in any one of the 329 plate-center batches as a contaminant. This analysis identified 57 putative contaminants out of 319 total fungi with ≥1 reads identified during direct genome alignments. Table S3 summarizes the *decontam* output and contaminant predictions.
Step #2: All 319 fungal taxa found in TCGA were cross-referenced against species identified in the WIS tumor mycobiome cohort (this study), the HMP gut mycobiome cohort (Nash et al., 2017), and 131 other papers in the literature (Table S3). This comprehensive literature survey informed the final decontamination decisions. Specifically, the following decision making process was applied: (i) Any fungal specie identified in the WIS tumor mycobiome cohort or HMP gut mycobiome cohort was retained; (ii) any fungal species known in the literature to have caused a clinically pathogenic infection or be a human commensal was retained; (iii) any fungal species with evidence of no known human association was discarded; (iv) any species that had little evidence for or against human associations (i.e., “unknown” human associations) had their fate decided by the plate-center *decontam* predictions. This process ultimately discarded 95 species (29.8% of total) as contaminants, comprising 2.1% of total reads, and retained 224 species as non-contaminants (Table S3).

##### Hopkins cohort decontamination

The Hopkins plasma cohort was originally collected to examine host-centric fragmentomic diagnostics (Cristiano et al., 2019) and did not employ contamination control samples. Since the TCGA contamination analysis thoroughly covered 319 out of 320 total fungi in the rep200 database, the contamination decisions from TCGA based on the WIS cohort, HMP gut mycobiome cohort (Nash et al., 2017), and 131 other papers were applied to the Hopkins cohort. The Hopkins cohort began with 296 identified fungal species and after decontamination retained 209 fungal species (29.4% removed).

##### UCSD cohort decontamination

The UCSD plasma cohort was designed to include positive and negative contamination control samples (Poore et al., 2020). Positive controls included 26 samples of serially diluted *Aliivibrio fischeri* (bacteria), which were previously analyzed (Poore et al., 2020), while negative controls included 15 blank DNA extraction samples and 11 blank library preparation samples. All control and biological samples were run on a single sequencing plate at a single time, as described previously (Poore et al., 2020). Decontamination was performed using *decontam* in (i) “prevalence” mode with P^∗^=0.5 among blanks and biological samples, and in (ii) “frequency” mode using the default P^∗^=0.1 (also used in TCGA) with DNA concentrations. Importantly, for “prevalence” mode, P^∗^=0.5 will flag taxa as contaminants if they are more prevalent in negative controls than in biological samples. These were run separately because several of the blanks had zero or otherwise undetectable DNA concentrations, which are compatible with “prevalence” filtering but not “frequency” filtering. “Prevalence” filtering flagged 30 out of 227 (13.2%) identified fungi while “frequency” filtering identified 4 out of 227 identified fungi (1.8%), or 32 unique total fungi (14.1%). These putative contaminants were then compared against the comprehensive TCGA decontamination analysis and guided the decision of any “unknown” human associated fungi. As with TCGA and the Hopkins cohorts, fungi matching the WIS cohort, HMP gut mycobiome cohort (Nash et al., 2017), or with known pathogenic/commensal associations were retained whereas those with evidence against human associations were removed. This ultimately left 215 decontaminated fungi for analysis in the UCSD cohort.

#### TCGA cohort: Coverage, genome assembly, binning, and phylogenetic placement

##### Aggregate fungal genome coverage calculations

Tarred output sam alignment files from Qiita were unzipped and searched using grep for all alignments belonging to fungal genome IDs in rep200. All fungal alignments were then inputted into custom python scripts (https://github.com/ucsd-cmi/zebra_filter) to calculate the aggregate fungal genomic coverage across fungal genomes in rep200 (Hakim et al., 2022). The outputs of this analysis were saved in Table S4.1, and a cutoff of ≥1% aggregate genome coverage (31 fungal species) was used in machine learning analyses. Additionally, using the extracted fungal alignments, we applied the same custom python scripts on each TCGA sample separately, which were run in parallel on a Slurm compute cluster, to calculate per-sample fungal genome coverage (Table S4.2). We also used the extracted fungal alignments in aggregate to calculate the aligned fungal read length distribution (mean=57.38bp; SD=15.89bp; median=51bp; note that host depletion enforced 45bp minimum).

##### Fungal metagenome assembly and binning

Due to the aggregate number and high prevalence of several species of bacteria and fungi in the TCGA metagenomic dataset, we aimed to better describe them through metagenomic assembly. First, preprocessed (i.e., trimmed, quality controlled, and human read filtered) WGS samples were coassembled by cancer type. Coassembly by cancer type was motivated by past findings that microbes are distributed differently across cancer types and that most individual samples had insufficient read depths to assemble microbial genomes (Nejman et al., 2020; Poore et al., 2020). Coassemblies were performed through metaSPAdes (v. 3.13.1) (Nurk et al., 2017) with an allocated memory of 1 TB RAM limit, across ten threads, and k-mer sizes 21, 33, 55, 77, 99, and 127. The resulting contigs from each co-assembly were filtered for a length of greater than 1500 and separated as being either prokaryotic or eukaryotic in origin with EukRep (v. 0.6.6) (West et al., 2018). Each set of prokaryotic or eukaryotic contigs were binned using MaxBin2 (v. 2.2.4) (Wu et al., 2016), MetaBAT2 (v. 2.12.1) (Kang et al., 2019), and Concoct (v. 1.0.0) (Alneberg et al., 2014) on default parameters and with contig abundance profiles estimated independently per sample. Abundance profiles were estimated by mapping reads against contigs using BowTie2 (v. 2.2.3) (Langmead and Salzberg, 2012) and SAMtools (v. 0.1.19) (Li et al., 2009). The three resulting sets of bins were refined into a single set with metaWRAP (v. 1.1.2) (Uritskiy et al., 2018). Quality metrics for the resulting prokaryotic refined bin sets were calculated using CheckM (v. 1.0.13) (Parks et al., 2015) For eukaryotic bins, we did not anticipate generating completed bins given their very low biomass but rather aimed to have phylogenetically placeable contigs or small bins; nonetheless, we quantified the estimated eukaryotic bin quality with EukCC (Saary et al., 2020) for the two bins with sufficient marker genes (Table S8.3).

##### Fungal phylogenomics

Fungal refined bin completeness were assessed with BUSCO v5.1.2 (Simão et al., 2015) using fungi_odb10 and ascomycota_odb10 lineage files. To phylogenetically classify the bins, BUSCO with fungi was also run on a set of 2,623 fungal genomes from NCBI relying on scripts for downloading and processing assemblies and gene sets (https://github.com/1KFG/NCBI_fungi) (Stajich and Ettinger, 2021). Assemblies were processed with BUSCO and ascomycota_odb10 to identify conserved marker genes, followed by extraction of the predicted proteins. The proteins were matched against HMMs for the markers using hmmsearch (Eddy, 2011), aligned with hmmalign, trimmed with clipkit (Steenwyk et al., 2020), and concatenated into super alignment with the PHYling pipeline (https://github.com/stajichlab/PHYling_unified). A phylogenetic tree to estimate phylogenetic placements of the bins was generated using individual gene trees computed with FastTree (Price et al., 2010) and combined with coalescent ASTRAL (Zhang et al., 2020). The small bins with insufficient recovery of BUSCO markers with assemblies of less than ∼85 kb were further processed for identification of conserved genes by aligning with Minimap2 (Li, 2018) using parameters “-x asm20” against the collection of NCBI fungal genomes. Further analysis by translated BLASTX searches against NCBI nr database also identified likely closest taxa. The bins identified to be mitochondrial sequence based on top BLASTX hits were further annotated for their protein coding and ncRNA genes using MFannot (https://github.com/BFL-lab/Mfannot). The translated peptides of the predicted genes were used for further phylogenetic analyses for species assignment.

#### TCGA cohort: Co-occurrence analyses with MMvec

In order to explore the fungal genera identified in the controlled amplicon based sequencing at a large scale, the TCGA metagenomic dataset count table was group summed to the genus level and matched to genera in the WIS amplicon data. This process was then repeated for the bacterial data, so that both tables were operating at the same taxonomic level and only contained WIS-overlapping features (Table S7.5). TCGA immune compositions were obtained from Thorsson *et al*. (Thorsson et al., 2018), who derived them using CIBERSORT (Newman et al., 2015) on TCGA RNA-Seq samples. Note that TCGA performed combined RNA-Seq and WGS on many samples, enabling usage of the WGS data to inform microbial composition and paired RNA-Seq data to inform immune cell composition. RNA-Seq microbial data was not used for co-occurrence analyses due to (i) much lower read microbial depths and (ii) bias in the bacterial data due to polyA selection as noted in TCGA SOPs. In this case, TCGA patient identifiers published by Thorsson *et al*. (Thorsson et al., 2018) were used to match immune cell compositions to microbial data.

MMvec (v. 1.0.6) (Morton et al., 2019a) was optimized between each data modality (i.e., bacteria, fungi, and immune cell composition) within each submission center (Harvard Medical School, Baylor College of Medicine, and MD Anderson) to (i) mitigate center effects and (ii) produce a minimized cross-validation (CV) error, log-loss, and a maximized Q-squared (1 − model coefficient of variation [CV] / null model CV) values. Note that a Q-squared value > 0 ensures a good model fit. Training and test labels were produced across all tables stratified by cancer type. Each model had the following optimized parameters: 2e3 to 5e3 iterations, batch size of one fourth the training tables numbed of features, number of epochs as (# of iterations ^∗^ batch size) / total reads in the training table, latent dimension of 3, and all other parameters were set to default. The null model operated on the exact same training/test set and parameters with the exception of the latent dimension set to zero. All models produced between all data modalities and submission centers had Q-squared values greater than zero, except for fungi and immune co-occurrences predicted from M D Anderson - Institute for Applied Cancer Science. To further mitigate any deleterious effect of center effects, only those co-occurrences that showed consistent trends between centers were retained.

To explore co-occurrence clusters between all data modalities, MMvec conditional probabilities were z-score transformed along the first axis (i.e., across columns of the MMvec output, as done elsewhere (Allaband et al., 2021)). In order to minimize the effect of the TCGA submission center, we explored only those features with consistent co-occurrences across TCGA submission centers—defined as features whose median co-occurrence values were less than the standard mean error (SEM) of their co-occurrence values across centers. Next, the median of these filtered features were taken across all submission centers. To explore the co-occurrence clustering and define subtypes across modalities, hierarchical clustering was performed through Scipy’s (v. 1.3.0) hierarchy linkage function (Virtanen et al., 2020) via Seaborn’s (v. 0.11.2) (Hunter, 2007) clustermap plotting function. Three fungi-driven “mycotypes,” or subtypes, were identified across the highest partition of linkages on the immune co-occurrences. These subtypes were defined as follows: F1 (*Malassezia*, *Ramularia*, and *Trichosporon*), F2 (*Candida*, *Aspergillus*), and F3 (*Tilletiopsis*, *Penicillium*, *Cryptococcus*, *Puccinia*, *Agaricus*, *Alternaria*, *Phialocephala*, *Fusarium*, *Hyphopichia*, *Exophiala*, *Stereum*, *Colletotrichum*, *Dissoconium*, *Aureobasidium*, *Talaromyces*, *Cutaneotrichosporon*, *Yarrowia*, and *Trichoderma*). The immune cells and bacterial genera associated with each mycotype were then defined by their within-linkage-cluster maximum co-occurrences.

To explore these fungal-bacterial-immune mycotypes further, pairwise log-ratios with a pseudocount of one (to reduce sample dropout) were taken across mycotypes in each data modality and explored across cancer type, submission center, and previously defined immune subtypes in Thorsson *et al*. (Thorsson et al., 2018) visually and through type-one ANOVA via statsmodels (Seabold and Perktold, 2010). Note that Thorsson *et al*. (Thorsson et al., 2018) defined their immune subtypes as follows: C1 = wound healing, C2 = IFN-γ dominant, C3 = inflammatory, C4 = lymphocyte depleted (with second highest macrophage activity), C5 = immunologically quiet (with highest macrophage activity), C6 = TGF-β dominant. Comparison of mycotype log-ratios across fungi, bacteria, and immune compositions were performed using Spearman correlation across samples, to evaluate similarity in log-ratio shifts across samples via Scipy. Overall survival (OS) data were obtained using data published by Thorsson *et al.* (Thorsson et al., 2018). Kaplan-Meier survival analysis for OS across partitions greater or less than the median value for each mycotype log ratio was performed using the survival package (v. 3.2) in R (Therneau, 2022) and statistically evaluated through Cox Proportional Hazards (CoxPH) in R modeling. All p-values across all statistics were corrected for False Discovery Rate (FDR) correction using the Benjamini-Hochberg method. For the survival analyses, the sample sizes of groups that were categorized above or below the median log-ratio values are shown in Table S7.6.

#### WIS cohort: Construction and analysis of the multi-domain interaction networks

The following analysis was performed with MATLAB version 2019b with the Statistics and Machine Learning Toolbox. To construct the network, we first chose a taxonomic level for the fungi and bacteria. We then constructed three different networks of interaction for each tumor type, fungus-to-fungus (FF) network, bacteria-to-bacteria (BB) network, and fungus-to-bacteria (FB), independently.

The relationship between each pair of taxa was calculated based on the presence/absence data, using the normalized mutual information (NMI) measure, which has been shown to perform as good or better than other ecological indicators of co-occurrence (Neeson and Mandelik, 2014)

Given two vectors, X and Y, each with M discrete elements (corresponding to M samples), $x_{i}$ and $y_{i}$ (i=1…M) which can be equal to either 0 or 1, the NMI between them is defined as

$$NMI\left(X,\;Y\right)=\frac{I\left(X,\;Y\right)}{\sqrt{H\left(X\right)H\left(Y\right)}}\;,$$

where I(X,Y)=H(X)+H(Y)−H(X,Y) is the mutual information between X and Y, H(X) and H(Y) are the Shannon entropies of X and Y respectively, and H(X,Y) is the joint entropy of X and Y, i.e.,

H(X)=−P(x=0)logP(x=0)−P(x=1)logP(x=1),

H(Y)=−P(y=0)logP(y=0)−P(y=1)logP(y=1),

and

$$H\left(X,\;Y\right)=\;-\sum_{x_{i}\in \left(1,0\right)\;}\sum_{y_{i}\in \left(1,\;0\right)}P\left(x,\;y\right)\;logP\left(x,\;y\right)\;.$$

The NMI is bounded between 0 and 1, where 0 indicates no relationship between the presence/absence of taxon X and Y and 1 indicates maximal relationship (can mean that both always appear together or never appear together, i.e., it does not distinguish the sign of the relationship).

The p-value is calculated as the fraction of times a random reshuffling process of the taxon had outputted greater or equal NMI value to the original samples:

$$p=\frac{\#NMI\left(X_{shuffle},\;Y_{shuffle}\right)\geq NMI\left(X,\;Y\right)}{\#Shuffle\;realizations}.$$

To fairly compare the NMI values of random realizations, the shuffling is done in a weighted manner which preserves the total number of observed taxa in each sample. The weight $w_{i}$ of each sample i is defined as

$$w_{i}=\;\frac{\#Observed\;taxa\;in\;sample\;i}{\#Total\;observed\;taxa\;in\;all\;the\;samples}$$

Then, the presence/absence of species X is randomly shuffled between the samples, with probability corresponding to the weight of each sample (i.e., the total number of fungi and the total number of bacteria present in each tumor was always kept as in the original data). The process is repeated 1000 times to calculate the p-value. We then perform BH FDR multiple comparison analysis on the p-values list of each tissue type and interaction type (FF, BB, and FB). Finally, a positive or negative sign of interaction was given to each pair of taxa according to Pearson correlation. Only pairs with FDR≤0.2 were used in the figures (Data S4.1A).

#### WIS cohort: 5.8S real-time quantitative PCR (RT-qPCR)

RT-qPCR was performed on the 5.8*S* region of the fungal rDNA. The following primers were used: Forward primer (ITS3) - 5’-GCATCGATGAAGAACGCAGC-3’ (White et al., 1990) and reverse primer (ITS86R) - 5’- TTCAAAGATTCGATGATTCAC-3’ (Turenne et al., 1999). qPCR was performed on 40ng of DNA per sample (or the maximum available in 5ul). For extraction controls a volume of 5ul per sample was used. For empty paraffin controls a volume equal to the volume taken for the matching sample from the same block was used. The PCR mix included 0.2uM of each primer, 5ul Kapa SYBR FAST qPCR Master Mix (2X) (Kapa Biosystems, #KK4605) and ultra pure water to a total volume of 10ul. PCR conditions used were 95°C 3min, (95°C 3sec, 58°C 20sec, 72°C 30sec) X40 cycles and included a dissociation curve at the end. ViiA 7 Real-Time PCR System (Applied Biosystems) was used for the qPCR. qPCR was performed in triplicates per sample and results were averaged across repeats. Fungal load was estimated by comparison to a standard curve created with *Saccharomyces cerevisiae* DNA that was spiked into human DNA.

#### WIS cohort: Staining methods

Human tumor tissue microarrays (TMAs) were purchased from US Biomax and included over 600 cores representing the following tumor types: breast (Cat#BR1191, Cat#BC08118a), lung (Cat#LC819a, Cat#LC813b), melanoma (Cat#ME804a), ovary (Cat#OV8010a) and PDAC (Cat#PA961f, Cat#PA804b). All TMAs were stained by H&E using standard protocol and serial sections from the same TMAs were used for the different stains (Figure 2; Data S2.3 and S2.4). All fungal antibodies were tested and their protocols calibrated on TMAs with known fungi in them that served as positive controls (Bio SB #BSB-0335-CS) (Data S2.1A).

##### Modified Gomori Methenamine-Silver (GMS) Nitrate Stain

GMS (abcam #ab150671) was used for staining. Slides were deparaffinized and rehydrated as described above. Next they were washed in distilled water twice and incubated in chromic acid solution for 20 minutes. Slides were rinsed in tap water, and then washed in distilled water twice. Slides were then incubated in sodium bisulfite solution for 1 minute and then rinsed as before (1 tap water, 2 distilled water). Next slides were incubated in a pre-warmed GMS solution for 7 minutes at 60°C after which they were rinsed 4 times in distilled water and incubated in gold chloride solution for 30 seconds. Four additional distilled water rinses were performed followed by incubation in sodium thiosulfate solution for 2 minutes. Slides were next rinsed in tap water and 2 changes of distilled water. Next slides were stained with light-green solution for 2 minutes. Finally, slides were rinsed in absolute alcohol 3 times, left to dry and mounted with synthetic resin. For GMS protocol all tools used were plastic or glass (no metal-containing tools were used).

##### 28S fungal fluorescence in-situ hybridization (FISH)

28*S* fungal FISH was performed with a mix of three fungal probes. ‘D-205’ probe: 5’- ATTCCCAAACAACTCGAC-3’; ‘D-223’ probe: 5’-CCACCCACTTAGAGCTGC-3’; and ‘D-260’ probe: 5’-TCGGTCTCTCGCCAATATT-3’ (Inácio et al., 2003), all conjugated to cy5 at the 5′ end (IDT). Non-specific complement probes for each of the three probes as well as a mix of all probes together were tested on positive control tissues that were known to contain fungi in them, and found to have no background fluorescence (Data S2.2A). For staining: slides were deparaffinized and rehydrated (Xylene for 10 minutes, Xylene for 5 minutes, 100% ethanol for 10 minutes X2, 96% ethanol for 10 minutes, 70% ethanol for 2-12 hours at 4°C). Slides were next rinsed in RNAse-free 2X SSC (Ambion #AM9765) for 10 minutes and proteinase K solution (10μg/ml in 2X SSC, Ambion #AM2546), pre-heated to 50°C was added to the slides. Slides were incubated for 10 minutes at 42°C. Slides were then rinsed twice with 2X SSC for 5 minutes each, followed by 2 rinses in wash buffer (2X SSC, 15% formamide (Ambion #AM9342)) for 5 minutes each. Next, slides were incubated overnight at 30°C with a probe mix of 1μM per probe in hybridization buffer (10% Dextran sulfate (Sigma #D8906), 15% formamide, 1mg/ml EcolitRNA (Sigma #R4251), 2X SSC, 0.02% BSA (Ambion #AM2616), 2mM vanadyl ribonucleoside (New England Biolabs #S1402S)). Slides were rinsed in wash buffer for 30 minutes at 30°C followed by incubation in wash buffer with DAPI with a final concentration of 1μg/ml. Finally, slides were washed in 2X SSC, 10mM TRIS pH 8 and 0.4% glucose and mounted with ProLong Gold Antifade Mountant (Life technologies #P36930).

##### Immunofluorescent staining

Slides were deparaffinized and rehydrated using the following protocol: Xylene for 10 minutes X2, 100% ethanol for 5 minutes, 96% ethanol for 5 minutes, 70% ethanol for 5 minutes and 3 washes in PBS for 2 minutes each. Next endogenous peroxidase quenching was performed (1% H2O2, 0.185% HCl) for 30 min, followed by antigen retrieval using citric acid buffer (pH 6) for 10 minutes at 95°C. Slides were left to cool at room temperature and then washed 3 times in PBS. Blocking was done with 1% BSA and 0.2% Triton in PBS for 60 minutes at R/T. Slides were incubated with primary antibodies that were diluted using a staining buffer (2% horse serum, 0.2% Triton in PBS) overnight at 4°C. The following antibodies were used: anti-1-3 β-glucan (abcam #ab233743; 1:50), anti-CD45 (eBioscience #14-0459-82; 1:100), anti-CD68 (Invitrogen #MA5-12407; 1:50), anti-Aspergillus (Abcam ab20419; 1:100), anti-CD8 (Abcam ab17147, 1:50). Slides were washed in PBS for 2 minutes and secondary antibodies and DAPI (1μg/ml) diluted in staining buffer were added for 30 minutes at room temperature. The following secondary antibodies were used: Goat anti-Mouse IgG2b Cross-Adsorbed Secondary Antibody with Alexa Fluor 555 (Invitrogen #A21147; 1:200), Goat anti-Mouse IgG1 Cross-Adsorbed Secondary Antibody with Alexa Fluor 647 (Invitrogen #A21240; 1:200), Goat anti-Mouse IgG3 Cross-Adsorbed Secondary Antibody with Alexa Fluor 488 (Invitrogen #A21151, 1:200) and Donkey anti-Rabbit IgG (H+L) Cross-Adsorbed Secondary Antibody with DyLight 755 (Invitrogen #SA5-10043, 1:100). Slides were washed twice in PBS and mounted with ProLong Gold Antifade Mountant (Life technologies #P36930).

##### Immunohistochemistry

Slides were stained by anti-fungal 1-3 beta-glucan (abcam #ab233743; 1:100) or no primary antibody (negative control) with the automated slide stainer BOND RXm (Leica Biosystems) using the Bond polymer refine detection kit (Leica Biosystems #DS9800), according to the manufacturer's instructions. Acidic antigen retrieval was done by a 20 min heating step with the epitope retrieval solution 1 (Leica Biosystems #AR9961).

##### Imaging

Slides stained in all staining methods (IHC/IF/FISH/GMS/CFW) were scanned with the Pannoramic SCAN II automated slide scanner (3D HISTECH) at 40X.

#### TCGA and UCSD cohorts: Batch correction

TCGA data was collected across a decade at multiple sequencing centers, sequencing platforms, and experimental strategies (WGS vs. RNA-Seq) among other technical variables. Fortunately, strict SOPs limited other forms of variation between centers. Our previous analyses on the TCGA bacteriome suggested that the largest technical factors were (in order from most to least) experimental strategy, sequencing center, and sequencing platform. Collectively, these factors explained 95.9% of the variability in bacterial data (Poore et al., 2020) using principal variance components analysis (PVCA) and necessitated batch correction prior to pan-cancer analyses. We found a similar effect within the fungal data, which motivated subsetting all samples to Illumina HiSeq platform, comprising 97% of samples (see Data S5 Note), and performing batch correction on the experimental strategy and sequencing center, which explained 49% and 30% of variance, respectively, using PVCA (Data S5.6A). Batch correction was applied using the combination of Voom and SNM, as done previously (Law et al., 2014; Mecham et al., 2010; Poore et al., 2020). Briefly, Voom converts discrete counts to pseudo-normally distributed (“microarray-like”) data (Law et al., 2014), which is then used by SNM to iteratively remove batch effects in a supervised manner (Mecham et al., 2010), such that biological signal is not removed while technical variance is removed. PVCA was used before and after batch correction (Scherer, 2009), as recommended by the National Institute of Environmental Health Sciences (NIEHS) (https://www.niehs.nih.gov/research/resources/software/biostatistics/pvca/index.cfm). We set the single tunable parameter for PVCA (the percentage of variance explained to obtain a number of PCA components) to 80%, based on NIEHS’s recommendation of 60–90% and our past analyses (Poore et al., 2020).

For Voom and SNM, the biological variable was sample type (e.g., tumor, NAT, blood) for TCGA and disease type for the UCSD cohort, both as done previously (Poore et al., 2020). During exploratory analyses of immunotherapy response in the UCSD cohort (Data S6.3D), the patient treatment response status was included as another biological variable in addition to disease type. We briefly but importantly note that SNM was designed for all possible biological variables to be included, including those that would later be examined using differential expression/abundance testing (Mecham et al., 2010). For technical factors, the TCGA cohort used experimental strategy and sequencing center, whereas age and sex were used for the UCSD cohort, both also done previously on the same cohorts (Poore et al., 2020). As with bacterial-centric data, PVCA on TCGA before and after batch correction on mycobiome data showed remarkable reduction in technical variable variance up to 15.5-fold while retaining or increasing (i.e., improving the signal-to-noise ratio) biological variable variance up to 7.9-fold (Data S5.6A).

When subsetting feature sets to those with (i) 34 WIS-overlapping fungal species, (ii) 31 fungal species with ≥1% aggregate genome coverage, (iii) the top 20 Hopkins-associated fungi, or (iv) overlapping WIS fungi and bacteria (approximately 300 species depending on the intersected dataset), the raw count data were first subset followed by Voom-SNM. This means that batch correction occurred independently on each smaller feature set prior to downstream machine learning. Performing PVCA on each of these feature sets before and after batch correction frequently showed similar reductions in technical variable variance and maintenance or increases in biological variable variance (data not shown). For the 22 fungal species detected by EukDetect (Lind and Pollard, 2021), the post-batch-corrected, decontaminated, species-level data in TCGA were subset prior to running downstream machine learning, which resulted in highly correlated performance to machine learning done on the 31 fungal species with ≥1% aggregate genome coverage that separately underwent batch correction (Data S5.1K–S5.1P).

#### All cohorts: Machine learning methods

##### Note of caution when interpreting AUROC and AUPR values

It is common to estimate ML performance using area under ROC (AUROC) and PR (AUPR) curves; however, there are important differences between them, as they measure different aspects of discrimination and have different null values. Specifically AUROC on a model that performs as good as random coin flipping would be approximately 50%, and this calculation takes into account both true positives and true negatives. However, the AUPR of a model that has null performance would actually have differing null areas depending on the underlying prevalence of the positive class, and the calculation does not take into account true negatives. For example, TCGA contains many more tumor samples than NAT samples, and we model tumor samples as the positive class since it represents an active diagnosis of cancerous tissue. A model that performs randomly on tumor vs. NAT discrimination would have an AUROC of ∼50% but a much higher AUPR (e.g., in a hypothetical case, if we had 90 tumors and 10 NAT samples, the null AUPR would be 90%). Furthermore, the calculations of precision and recall on the resultant predictions would not take into account how many samples were true negatives (i.e., those predicted to be NAT and indeed being NAT). Both of these can make interpretation of AUPR difficult, especially when compared to one-cancer-type-versus-all-others ML models, where the prevalence of the positive class (cancer type of interest), and thus null AUPR, is often in the range of 1-10%. Nonetheless, it is common to advocate for measuring AUPR in addition to AUROC when classes are imbalanced, since large class imbalances in certain circumstances can artificially raise AUROCs. Thus, for these analyses, we have consistently calculated both and indicated the null AUROCs and null AUPRs on most ML performance plots, and we continue to caution that for analyses where true negatives are important AUROCs may be more appropriate to examine.

##### ML of individual cancer types versus each other or controls

We previously published ML on the TCGA bacteriome using stratified 70% training, 30% holdout testing splits (Poore et al., 2020) across all cancer types. While suitable for the large number of ML models being built and tested within and between cancer types in TCGA, this strategy did not provide information of performance error ranges. We thus decided to modify the strategy for the mycobiome analyses in such a way to provide both the performance estimate and a confidence interval for that performance across each cancer type without largely increasing compute times for each model. Specifically, for each model, we performed 10-fold cross validation using gradient boosting models (GBMs) with ten independent, stratified 10% holdouts (i.e., the prediction class proportions are similar in train/test, such that if the entire dataset was 10 positive class and 90 negative class, then each independent k^th^ holdout would have 1 positive class sample and 9 negative class samples). ROC and PR curves and areas were calculated for each independent 10% holdout test set, such that ten sets of two-class discriminatory performance—effectively ten sets of 90% training-10% testing—were obtained for each model. To be clear, the sampling algorithm used in the R caret package for machine learning (https://topepo.github.io/caret/model-training-and-tuning.html) treated each 10% cross-validation fold set as an independent test set when training a model, and that since the hyperparameters were fixed (see below), the test outputs were representative of ten train/test iterations on different splits in the data. These performance estimates on the ten folds were then aggregated for each model to calculate the 95% confidence intervals of performance. One other key difference between this and our previous approach (Poore et al., 2020) is that the hyperparameter grid search was removed in favor of a fixed GBM grid with the following parameters: {n.trees=150, interaction.depth=3, shrinkage=0.1, n.minobsinnode=1}. We note that these parameters were possible in our past TCGA analysis (Poore et al., 2020) and were equal to those used in the host-centric fragmentomic analyses of the Hopkins cohort (Cristiano et al., 2019) (https://github.com/cancer-genomics/delfi_scripts/blob/master/06-gbm_full.r). Equal to our last approach (Poore et al., 2020), we also up-sampled the minority class in cases of class imbalance while requiring ≥20 samples in the minority class to help the model generalize. We also centered and scaled the data prior to ML model building when using Voom-SNM batch corrected data; however, when using raw count data, we only removed zero variance features prior to the ML model building. The one exception to this comprised models run in the “***Validation of ML approach in TCGA using different models and sampling strategies***” section (below), which did not apply any preprocessing on the data. ML was rapidly iterated on TCGA, WIS, Hopkins, and UCSD cohorts, collectively representing hundreds of models and thousands of independently held out folds. We also note that in the case of WIS data, all filtered fungal or bacterial hits were used regardless of taxonomic rank (i.e., “free rank” data), based on empirical performance benefits, whereas ML in TCGA, UCSD, or Hopkins was performed with data summarized to a single taxonomic level (e.g., species, genus).

##### Validation of ML approach in TCGA using different models and sampling strategies

To ensure that the GBM models were not providing artificially higher performance or overfitting the data, we also re-ran all TCGA primary tumor, blood, and tumor vs. NAT machine learning models, using batch-corrected or raw decontaminated data at every taxa level, using random forests (RF), which are less prone to overfit, with fixed hyperparameters (n_trees=500, mtry=4). Specifically, we ran the random forest models with the same k^th^-fold splits, so that the GBM and RF performances were directly paired. We then regressed the paired RF and GBM k^th^-fold performances, as shown in Data S5.14. The very strong, significant performance correlations, which held for all taxa levels, suggested that the strong ML performance was generalizable to other model types that were less prone to overfitting. For brevity, although models built on raw decontaminated data at every taxa level were calculated separately within each TCGA sequencing center, they were plotted in Data S5.14 in aggregate.

Furthermore, to ensure that the ten-fold cross-validation sampling strategy was not providing artificially higher performance, we re-ran all TCGA primary tumor, blood, and tumor vs. NAT machine learning models, using batch-corrected or raw decontaminated data at every taxa level, with an explicit holdout test set. Specifically, the following steps were done: (1) Perform stratified random sampling to allocate 90% of the data to a training set and 10% to a holdout test set, (2) train a gradient boosting machine learning model on the 90% training dataset using 4-fold cross-validation, (3) apply the trained model onto the 10% holdout test set, (4) repeat steps #1-3 for a total of 10-times per comparison using different train-test splits, and (5) calculate the 95% confidence interval of performance based on the AUROCs and AUPRs measured on the ten independent holdout test sets. The 95% confidence intervals of these performances for all cancer types were then regressed against the 95% confidence intervals generated using the 10-fold CV method, as shown in Data S5.15. The comparison revealed strong, significant correlations between the two methods, which held for all taxa levels (Data S5.15). For brevity, although models built on raw decontaminated data at every taxa level were calculated separately within each TCGA sequencing center, they were plotted in Data S5.15 in aggregate.

##### Multi-class ML in TCGA using raw data

During validation analyses on raw TCGA count data (see Data S5 Note), we noticed that independently training ML models on two stratified TCGA halves and subsequently testing on the other half provided highly concordant performance (Data S5.3G and S5.3H). (Note that stratified samples were based on sequencing center, sample type, and disease type and that experimental strategy was covered since 7 of 8 sequencing centers only performed WGS or RNA-Seq, and the one that did both [Broad] processed 83% of samples with WGS only.) This motivated testing whether multi-class machine learning was possible. Since experimental strategy had the largest batch effect (see “TCGA cohort: Batch correction” section above), we conservatively used WGS-only samples to ensure that multi-class ML performance would not be affected by WGS vs. RNA-Seq variability. We then ran multi-class ML using the same GBM modeling approach described above with 10-fold cross-validation. This type of multi-class ML came up in two circumstances: (1) Comparing the pan-cancer ML performance in TCGA of WIS-overlapping fungal species vs. equal sized feature sets of non-WIS-overlapping features in tumor tissue and blood, and (2) comparing the relative pan-cancer performance in TCGA of WIS-overlapping fungi, bacteria, or both. Details of these are provided below, and as above, we note that up-sampling the minority class and removing zero variance samples were continued here.

Case #1: A total of 34 fungal species overlapped between TCGA and WIS cohorts. To test whether these features were more informative when discriminating between cancer types versus similarly sized feature sets, we did the following: (1) Randomly sample 34 non-WIS-overlapping fungal species; (2) perform pan-cancer ML using multi-class classification with 10-fold cross-validation using WIS-overlapping fungi; (3) perform pan-cancer ML using multi-class classification with 10-fold cross-validation using non-WIS fungi; (4) calculate average performance (AUROC, AUPR) across all one-cancer-type-versus-all-others comparisons based on the iterative, independent holdout folds for models built in steps #2-3; (5) repeat steps #1-5 for a total of 50 times, thereby calculating ML performance on 500 aggregate folds; (6) repeat for both primary tumor and blood derived normal samples. The resultant performance indeed suggested that WIS-overlapping fungi provided better pan-cancer discriminatory performance (Data S5.5A, S5.12F, and S5.12G).
Case #2: To test whether adding fungal to bacterial information would improve pan-cancer discrimination, we did the same procedure as Case #1 except that three feature sets were used, consisting of WIS-overlapping fungi, WIS-overlapping bacteria, and both WIS-overlapping fungi and bacteria. We note that WIS-overlapping features were used for these analyses because they represented the most confident species calls identified in two international cohorts. The resultant performance indeed suggested that combining fungal and bacterial information synergistically provided better pan-cancer discriminatory performance (Figures 5C and 5G).

##### Hopkins and UCSD pan-cancer analyses

Cristiano *et al*. (Cristiano et al., 2019) originally benchmarked the performance of host-centric, fragmentomic, pan-cancer diagnostics using GBM ML models based on 10-fold cross-validation repeated 10 times using the following model hyperparameters (https://github.com/cancer-genomics/delfi_scripts/blob/master/06-gbm_full.r): {n.trees=150, interaction.depth=3, shrinkage=0.1, n.minobsinnode=1}. Notably, the only major ML difference between their method and ours (described above) was that we did not repeat the 10-fold cross validation ten-times. Thus, to directly compare our pan-cancer performance on the Hopkins cohort with their previously published results, we implemented an approach to repeat the 10-fold cross-validation ten-times, such that the ten iterations of performance measurement were done on the aggregated predictions. In other words, the first iteration of this method created 10 sets of predictions of equal dimensions to the input data that were aggregated into a single prediction vector prior to AUROC/AUPR performance measurement, rather than having 10 separate predictions per iteration each with AUROC and AUPR measurements. Collectively, this procedure left 10 AUROC and 10 AUPR values, one for each repeat of the 10-fold cross-validation. These ten values were used to estimate the 99% confidence intervals of performance and were overlaid on plots with the average performance and confidence interval ribbons (Figures 6H, 6J, and 6K).

Regarding plotting, we adapted an approach from the scikit-learn python package (https://scikit-learn.org/stable/auto_examples/model_selection/plot_roc_crossval.html) in R to estimate the average AUROC and AUPR curves among their 10 repeated iterations. This can be a challenging task because the specificity breaks of the ten model iterations are not always equivalent to each other, requiring interpolation. Specifically, to obtain the average performance lines, we performed linear interpolation using the *approx()* base R function of each ROC and PR curve across 1000 equally spaced points between 0 and 1, also ensuring that each average curve begins and ends at the corners of the plots. The 1000 interpolated y-values between x=0 and x=1 were then used to calculate the average ROC and PR curve and its concomitant 99% confidence interval at each point. Overlaying these average performance lines with 99% confidence interval ribbons showed good concordance (Figures 6H, 6J, and 6K).

##### Immunotherapy response predictions

A small number of patients with melanoma and lung cancer in the UCSD cohort had clinical immunotherapy response information available. Due to small sample sizes, machine learning on these patients was done using nested leave-one-out cross-validation, such that each *k*^th^ patient was iteratively left out and a model was built on the *k-1* patients (tuned using internal four-fold cross-validation) to make a prediction about the immunotherapy response of the *k*^th^ patient. In this case, a hyperparameter grid search was employed to select optimal parameters based on the internal four-fold cross validation for each iteration, and this grid included the following options: {interaction.depth = seq(1,3), n.trees = floor((1:3) ^∗^ 50), shrinkage = 0.1, n.minobsinnode = 1}. After iterating through all *k* patients, the list of predicted responses and known responses were compared to calculate ROC and PR curves and their respective areas. Using WIS-overlapping fungi, moderate discrimination between responders and non-responders was observed in patients with melanoma (Data S6.3D) but not in lung cancer (data not shown).

##### Scrambled and shuffled control analyses

In addition to comparing ML model performances to null AUROC and AUPR values, we wanted to implement additional negative control analyses. These were done in two independent ways just prior to ML model building: (1) scrambling metadata of prediction labels and (2) shuffling the sample IDs in the count data. We note that the scrambling and shuffling can occur globally (i.e., once before all ML models are built and tested) or dynamically (i.e., just prior to ML model building but after data subsetting and labeling). For example, when discriminating one cancer type versus all others, global scrambling would randomly sample all disease type labels among all sample types, whereas dynamic scrambling would happen only after subsetting to primary tumors and relabeling the disease types to two classes (i.e., the cancer type of interest and “Others”). We tested both of these approaches and found that both generally worked; however, the dynamic scrambling and shuffling yielded more consistent results (less variance) and showed greater agreement with known null values (i.e., 50% AUROC and positive class prevalence for AUPR). Hence, we used dynamic scrambling and shuffling as negative controls when comparing performance to actual samples.

##### Taxonomic generalizability

To test taxonomic generalizability, we aggregated raw read counts based on the decontaminated fungal data up the taxonomic levels (species through phylum) prior to ML using the phyloseq R package (v. 1.38.0) (McMurdie and Holmes, 2013). Aggregated counts were then inputted into the same ten-fold cross-validation models (repeated once) described above to estimate performance and concomitant 95% confidence intervals (Data S5.2 and S5.11).

##### Stratified halves validation analyses

As another control, we split raw TCGA count data into two stratified halves using sequencing center, sample type, and disease type metadata information. We again note that experimental strategy was covered in this stratification since 7 of 8 sequencing centers only performed WGS or RNA-Seq, and the one that did both [Broad] processed 83% of samples with WGS only. We then used both of these stratified halves to iteratively train ML models employing ten-fold cross-validation (repeated once) predicting one cancer type versus all others; each trained model was then immediately applied to the data of the other stratified half to discriminate that particular cancer type. The ML performance from testing each model on the corresponding half was then compared, revealing highly concordant values (Data S5.3G and S5.3H). This process was repeated using Voom-SNM normalization as well with the same procedure except that Voom-SNM normalization occurred independently on each half after stratification but prior to ML model building/testing. This additional analysis showed concordant performance among TCGA primary tumors and blood samples (Data S5.6C and S5.6D).

#### TCGA cohort: Differential abundance testing

As a positive control, yet orthogonal analysis of the cancer type ML, we implemented differential abundance testing using ANCOM-BC, which is currently the only statistically valid microbiome test that provides appropriate taxon-specific p-values and differential abundance confidence intervals for each taxon while controlling the false discovery rate (Lin and Peddada, 2020). We thus implemented ANCOM-BC on raw TCGA count data subsetted to each sequencing center to (i) discriminate between cancer types using primary tumors or blood, or (ii) discriminate between stage I and IV tumors. For (i), ANCOM-BC was applied with the following parameters: {p_adj_method = "BH", zero_cut = 0.999, lib_cut=1000, tol = 1e-5, max_iter = 100, conserve = FALSE, alpha = 0.05, global = FALSE}. Statistical discrimination was done per cancer type versus all others within each subset. We then used the calculated beta values, p-values, and BH adjusted q-values to make volcano plots (Data S5.4, S5.7, and S5.9). For (ii), the ANCOM-BC parameters were the same except for lib_cut, which was 100. For both (i) and (ii), we required a minimum of 10 samples in each class before computing differentially abundant taxa.

### Quantification and statistical analysis

#### WIS cohort: Statistical analyses

Most of the downstream analysis and plots were performed with R version 4.03. Packages used in analysis include phyloseq 1.34.0, ggplot2 3.3.4, ggbeeswarm 0.6.0, ggrepel 0.9.1. VennDiagram 1.6.20, pheatmap 1.0.12, ggforce 0.3.3, ggpubr 0.4.0, RColorBrewer 1.1-2, proxy 0.4-26, reshape2 1.4.4, stringr 1.4.0, dplyr 1.0.7, purrr 0.3.4, readr 1.4.0, tidyr 1.1.3, tidyverse 1.3.1. We note that the R programming language has two numerical limits when it comes to calculating small numbers, including p-values: (1) double eps, or smallest positive floating-point number x such that 1 + x!= 1, which is 2.220446×10^−16^; (ii) double x_min_, or the smallest non-zero normalized floating-point number, which is 2.225074×10^−308^ (although this limit may be even lower depending on the computing environment). Some R packages, notably ggpubr, do not report p-values less than double eps, so they are denoted in our data as p<2.2×10^−16^; conversely, other R packages, notably rstatix (listed below), report p-values as low as double x_min_, and p-values that were less than double x_min_ in our data are reported as p<2.2×10^−308^. They are not a range of p-values.

#### TCGA, Hopkins, and UCSD cohorts: Statistical analyses

Downstream analyses and plots were generated with either R version 4.03 or 4.1.1. Common R packages used include phyloseq (v. 1.38.0), vegan (v.2.5-7), microbiome (v. 1.16.0), doMC (1.3.7), dplyr (v. 1.0.7), reshape2 (v. 1.4.4), ggpubr (0.4.0), ggsci (v. 2.9), rstatix (v. 0.7.0), ggrepel (v. 0.9.1), tibble (v. 3.1.6), caret (v. 6.0-90), gbm (v. 2.1.8), xgboost (v. 1.5.0.1), randomForest (v. 4.6-14), MLmetrics (v. 1.1.1), PRROC (v. 1.3.1), e1071 (v. 1.7-9), gmodels (v. 2.18.1), ANCOM-BC (v. 1.4.0), decontam (v. 1.14.0), limma (v. 3.50.0), edgeR (v. 3.36.0), snm (v. 1.42.0), biomformat (v. 1.22.0), and Rhdf5lib (v. 1.16.0). The rstatix package corrected for multiple hypothesis testing where applicable. Sample sizes were not estimated in advance and power calculations were not performed. The gbm package was used for two-class gradient boosting ML, the xgboost package was used for multi-class gradient boosting ML, and the randomForest package was used for two-class random forest ML. AUROC and AUPR were calculated using the PRROC package.

### Additional resources

#### Website

A website summarizing the TCGA analyses was created using RShiny (https://shiny.rstudio.com/). There are several clickable tabs on the left-hand side that reveal (1) interactive ANCOM-BC differential abundance volcano plots; (2) hundreds of ML models on raw and batch-corrected data with associated performance plots, confusion matrices, and ranked feature lists; and (3) pan-cancer normalized abundance plots at species and genus-levels for viewing normalized fungal abundances in TCGA. The website can be accessed at http://cancermycobiome.ucsd.edu/.

## Data Availability

TCGA, Hopkins, and UCSD cohorts: Raw data BAM files comprise sensitive patient data and require data access approval. To access raw TCGA BAM files, researchers will need to apply to the TCGA Data Access Committee (DAC) through dbGaP (https://dbgap.ncbi.nlm.nih.gov/aa/wga.cgi?page=login). To access raw Hopkins BAM files, researchers will need to apply through the European Genome-Phenome Archive (EGA): EGAS00001003611. Host-depleted UCSD BAM files are available on the European Nucleotide Archive (ENA): ERP119598 (UCSD HIV-negative controls); ERP119596 (UCSD prostate cancer); ERP119597 (UCSD lung cancer and melanoma). Additionally, all host-depleted fastq files and processed biom tables for TCGA, Hopkins, and UCSD cohorts are available on Qiita (https://qiita.ucsd.edu/): 13722 (TCGA WGS), 13767 (TCGA RNA-Seq), 13984 (Hopkins), 12667 (UCSD HIV-free controls); 12691 (UCSD prostate cancer); 12692 (UCSD lung cancer and melanoma). Physical sample accession for TCGA, Hopkins, and UCSD cohorts is not available. WIS cohort: Breast and colon samples from Sheba; melanoma samples from the Netherlands Cancer Institute–Antoni van Leeuwenhoekziekenhuis (NKI-AVL); and lung, breast, ovary, and GBM samples from the Israeli Biorepository Network for Research (MIDGAM) are available from R.S. under material transfer agreements with the Weizmann Institute. All processed data of the WIS cohort are available in the manuscript or the supplementary materials. WIS ITS2 amplicon data are available from the National Center for Biotechnology Information (NCBI) BioProject: PRJNA786764. The ITS2 pipeline scripts are available on GitHub: https://github.com/microbiofunc/ITS2-pipeline. All files and code associated with phylogenomics of the fungal bins are available on Zenodo (https://doi.org/10.5281/zenodo.6419964) and GitHub (https://github.com/stajichlab/Tumor_mycobiome_2022). All code associated with TCGA, Hopkins, and UCSD analyses in this manuscript, including processed data, is available at the following GitHub repository link: https://github.com/knightlab-analyses/mycobiome. The website associated with this paper is http://cancermycobiome.ucsd.edu/.

## Supplemental information

Table S1. Detailed breakdown of sample cohorts, related to Figure 1

Table S2. WIS cohort ITS2 sequencing related data, related to Figures 3 and 6

Table S3. In-depth contamination analysis for TCGA fungal calls, related to Figures 3, 5, and 6 and STAR Methods

Table S4. Pan-TCGA fungal genome coverages, related to Figure 5, Data S1, and STAR Methods

Table S5. PERMANOVA analysis within each disease type for sample-type for both Aitchison and Bray-Curtis distances to compare tumor versus NAT, related to Figure 3 and Data S3

Table S6. WIS fungi-bacteria network analysis results with FDR less than 0.2, related to Data S4

Table S7. Mycobiome-bacteriome-immunome analysis, related to Figure 4 and Data S4

Table S8. EukDetect-identified fungi, top plasma fungi in Hopkins, and TCGA fungal bin completeness, related to Figures 1 and 6, Data S1, S5, and S6, and STAR Methods

Data S1. Distribution of fungal and bacterial nucleic acids across cancer types, related to Figure 1 and STAR Methods

Data S2. Fungal imaging in human tumors, related to Figure 2 and STAR Methods

Data S3. Cancer type-specific mycobiome characteristics and NAT comparisons, related to Figure 3

Data S4. Fungal-bacterial-immune analyses, related to Figure 4

Data S5. Mycobiome machine learning and control analyses, related to Figure 5 and STAR Methods

Data S6. Clinical associations and utility of fungal DNA in tumors or plasma, related to Figure 6
