# Supplementary material for: Pan-cancer analyses reveal cancer-type-specific fungal ecologies and bacteriome interactions
Source: Cell. 2022 Sep 29;185(20):3789–3806.e17. doi: 10.1016/j.cell.2022.09.005 (PMC9567272; doi:10.1016/j.cell.2022.09.005)
Supplement: Data S1. Distribution of fungal and bacterial nucleic acids across cancer types, related to Figure 1 and STAR Methods [file mmc9.pdf]

Pan-cancer analyses reveal cancer type-specific fungal ecologies and bacteriome interactions

DATA S1

Distribution of fungal and bacterial nucleic acids across cancer types, related to **Figure 1** and **STAR Methods**.

Table of Contents

Data S1.1. Distribution of fungal nucleic acids across cancer types and their correlation with bacterial nucleic acids 3

Data S1.2. Distribution of pan-microbial and bacterial nucleic acids across TCGA cancer types and the comparison of genome-normalized fungal versus bacterial proportions 6

Data S1.3. Comparison of pan-cancer fungal and bacterial read proportions in TCGA 9

Data S1.4. Phylogenomics of several metagenome-assembled fungal bins from TCGA non-human data 11

Data S1.5. ITS2 classification and taxa overlap between WIS and TCGA cohorts 13

Data S1.6. ITS2 sequencing pipeline development 14

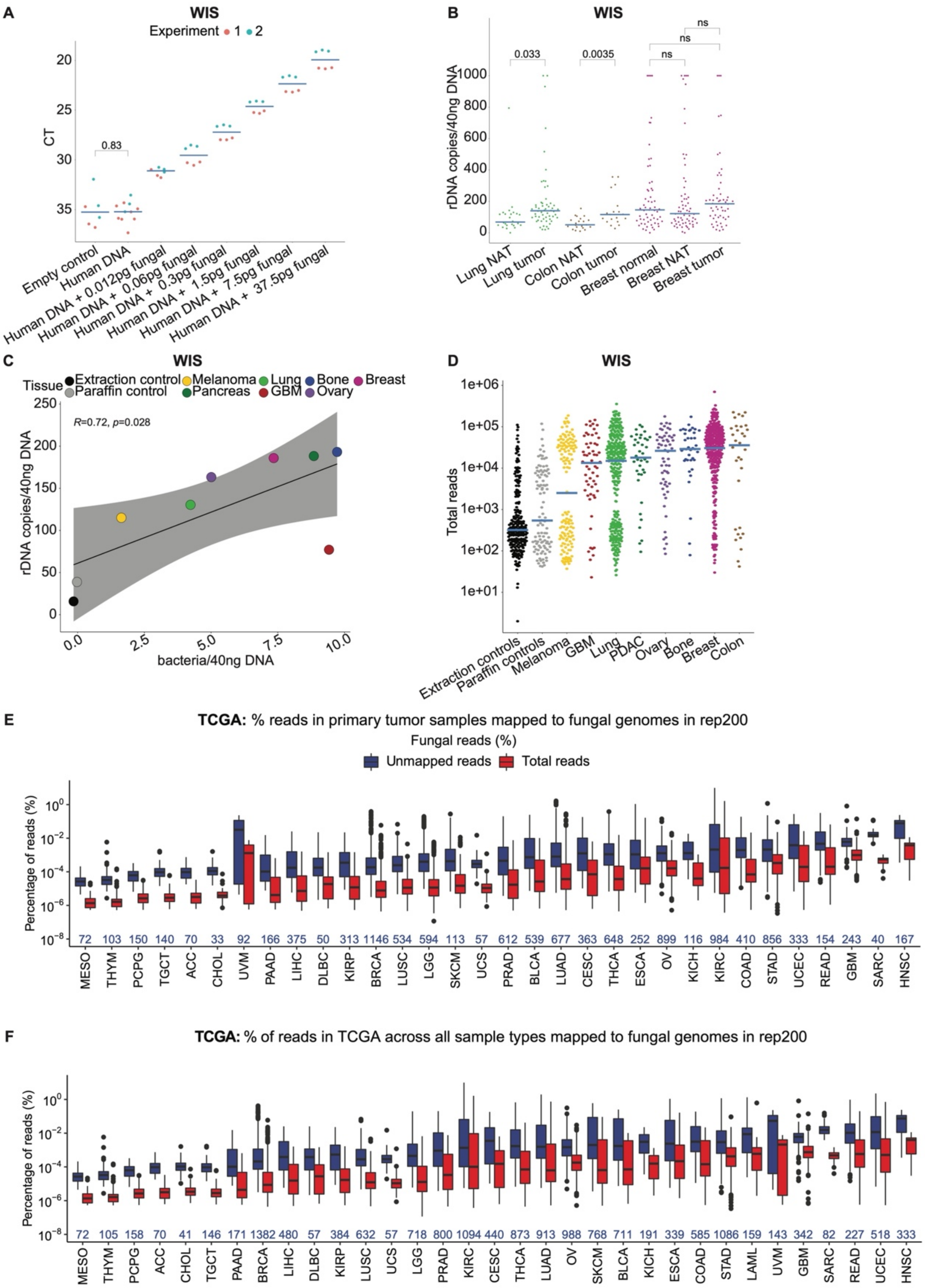
A
WIS
Experiment 1 2
CT
0.83
Empty control
Human DNA
Human DNA + 0.012pg fungal
Human DNA + 0.06pg fungal
Human DNA + 0.3pg fungal
Human DNA + 1.5pg fungal
Human DNA + 7.5pg fungal
Human DNA + 37.5pg fungal
B
WIS
rDNA copies/40ng DNA
0.033
0.0035
ns
ns
ns
Lung NAT
Lung tumor
Colon NAT
Colon tumor
Breast normal
Breast NAT
Breast tumor
C
WIS
Tissue Extraction control Paraffin control Melanoma Pancreas Lung GBM Bone Ovary Breast
R=0.72, p=0.028
rDNA copies/40ng DNA
bacteria/40ng DNA
D
WIS
Total reads
Extraction controls
Paraffin controls
Melanoma
GBM
Lung
PDAC
Ovary
Bone
Breast
Colon
E
TCGA: % reads in primary tumor samples mapped to fungal genomes in rep200
Fungal reads (%)
Unmapped reads Total reads
Percentage of reads (%)
72 103 150 140 70 33 92 166 375 50 313 1146 534 594 113 57 612 539 677 363 648 252 899 116 984 410 856 333 154 243 40 167
MESO THYM PCPG TGCT ACC CHOL UVM PAAD LIHC DLBC KIRP BRCA LUSC LGG SKCM UCS PRAD BLCA LUAD CESC THCA ESCA OV KICH KIRC COAD STAD UCEC READ GBM SARC HNSC
F
TCGA: % of reads in TCGA across all sample types mapped to fungal genomes in rep200
Percentage of reads (%)
72 105 158 70 41 146 171 1382 480 57 384 632 57 718 800 1094 440 873 913 988 768 711 191 339 585 1086 159 143 342 82 227 518 333
MESO THYM PCPG ACC CHOL TGCT PAAD BRCA LIHC DLBC KIRP LUSC UCS LGG PRAD KIRC CESC THCA LUAD OV SKCM BLCA KICH ESCA COAD STAD LAML UVM GBM SARC READ UCEC HNSC

G

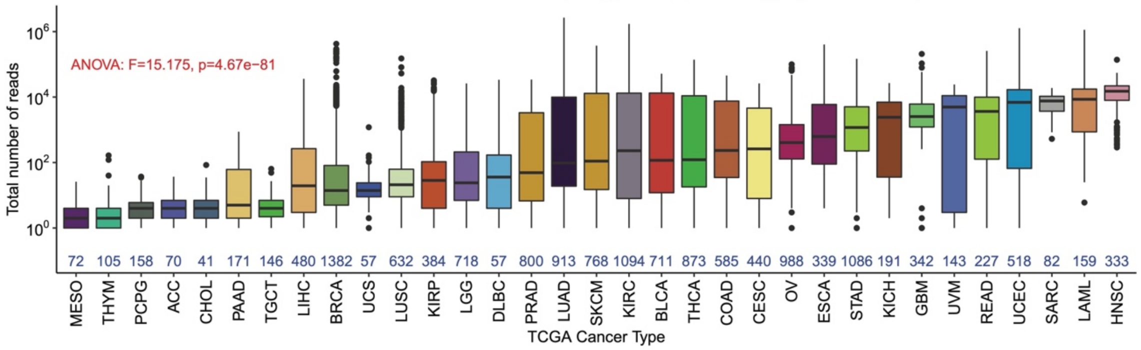

H

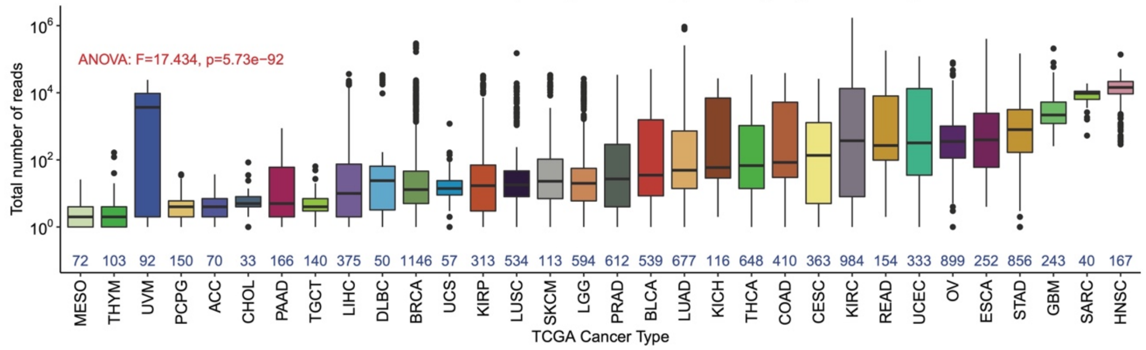

Data S1.1. Distribution of fungal nucleic acids across cancer types and their correlation with bacterial nucleic acids

(A) 5.8*S* qPCR sensitivity and specificity analysis. qPCR cycle threshold values of empty control containing only sterile water, 40ng of human DNA, and subsequent spike-in of gradually increasing amounts of fungal DNA. Human DNA from three different human cell lines (HS-5, AsPC1, G361) was used in Experiment 1 (red) and from only HS-5 in Experiment 2 (cyan). The lower bound of sensitivity is 0.012pg of fungal DNA, which reflects one cell of S. cerevisiae. Primers are specific for fungi as human DNA amplification is comparable to empty water control (p=0.83, t-test).

(B) Comparison of fungal DNA abundance in tumors and their NAT/normal counterparts in WIS cohort quantified by 5.8*S* qPCR. Blue bars show medians. Values clipped at 1000. Significant p-values of one-sided T-tests between tumor types and a random subset of their respective NAT/normal samples are displayed. ns, non-significant. Fungal load is significantly higher in both colon and lung tumors as compared to their adjacent tissue. While the load of fungi in breast tumors is also higher than breast NAT and normal tissues, this difference is not statistically significant. Lung NAT, n=24; Lung tumor n=59; Colon NAT, n=22; Colon tumor, n=20; Breast normal, n=60; Breast NAT, n=64; Breast tumor, n=57.

(C) Scatter plot demonstrating the Pearson correlation between fungal and bacterial load (median per tumor type) as measured by qPCR across seven tumor types and controls from the WIS cohort. Fungal load is represented as rDNA copies since fungi contain a wide range of rDNA copies per cell across different species. Regression lines and confidence intervals are shown. Pearson correlation coefficient (R) and p-value (p) are presented.

(**D**) Violin dot plot of the number of total ITS2 fungal reads (before flooring and normalization) per sample. Blue bars represent the median.
(**E**) Percentage of reads in TCGA primary tumors mapped to fungal genomes in the rep200 database versus unmapped (blue) and total (red) reads in the concomitant bam files. One-way ANOVA results showed significant variation between cancer types for fungal percentages of unmapped ($F=17.96$, $p=3.45\times10^{-95}$) and mapped ($F=18.81$, $p=2.50\times10^{-100}$).
(**F**) Percentage of reads in TCGA across all sample types mapped to fungal genomes in the rep200 database versus unmapped (blue) and total (red) reads in the concomitant bam files. One-way ANOVA results showed significant variation between cancer types for fungal percentages of unmapped ($F=22.35$, $p=1.21\times10^{-126}$) and mapped ($F=18.87$, $p=1.66\times10^{-104}$).
(**G**) Number of fungal reads in TCGA across all sample types mapped to fungal genomes in the rep200 database. (H) Number of fungal reads in TCGA primary tumors mapped to fungal genomes in the rep200 database.
(**E-G**) Y-axis is shown on a log scale. Samples sizes are inset in blue and reflect samples with non-zero fungal reads. One-way ANOVA statistics inset on plot.
(**C, D**) Box plots show median (line), 25th and 75th percentiles (box), and 1.5× the interquartile range (IQR, whiskers).

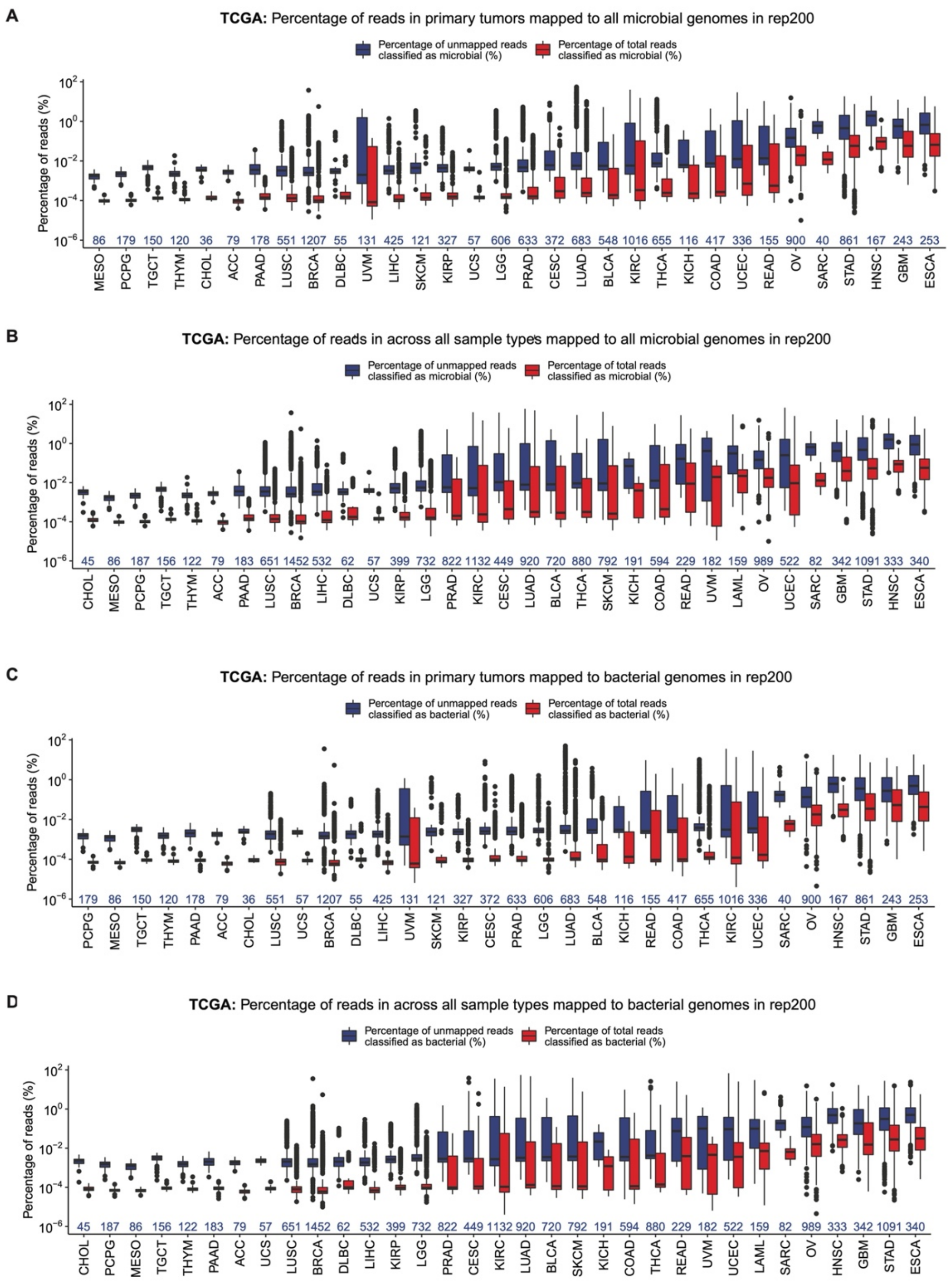
A
TCGA: Percentage of reads in primary tumors mapped to all microbial genomes in rep200
Percentage of unmapped reads classified as microbial (%)
Percentage of total reads classified as microbial (%)
Percentage of reads (%)
86 179 150 120 36 79 178 551 1207 55 131 425 121 327 57 606 633 372 683 548 1016 655 116 417 336 155 900 40 861 167 243 253
MESO PCPG TGCT THYM CHOL ACC PAAD LUSC BRCA DLBC UVM LIHC SKCM KIRP UCS LGG PRAD CESC LUAD BLCA KIRC THCA KICH COAD UCEC READ OV SARC STAD HNSC GBM ESCA
B
TCGA: Percentage of reads in across all sample types mapped to all microbial genomes in rep200
Percentage of unmapped reads classified as microbial (%)
Percentage of total reads classified as microbial (%)
Percentage of reads (%)
45 86 187 156 122 79 183 651 1452 532 62 57 399 732 822 1132 449 920 720 880 792 191 594 229 182 159 989 522 82 342 1091 333 340
CHOL MESO PCPG TGCT THYM ACC PAAD LUSC BRCA LIHC DLBC UCS KIRP LGG PRAD KIRC CESC LUAD BLCA THCA SKCM KICH COAD READ UVM LAML OV UCEC SARC GBM STAD HNSC ESCA
C
TCGA: Percentage of reads in primary tumors mapped to bacterial genomes in rep200
Percentage of unmapped reads classified as bacterial (%)
Percentage of total reads classified as bacterial (%)
Percentage of reads (%)
179 86 150 120 178 79 36 551 57 1207 55 425 131 121 327 372 633 606 683 548 116 155 417 655 1016 336 40 900 167 861 243 253
PCPG MESO TGCT THYM PAAD ACC CHOL LUSC UCS BRCA DLBC LIHC UVM SKCM KIRP CESC PRAD LGG LUAD BLCA KICH READ COAD THCA KIRC UCEC SARC OV HNSC STAD GBM ESCA
D
TCGA: Percentage of reads in across all sample types mapped to bacterial genomes in rep200
Percentage of unmapped reads classified as bacterial (%)
Percentage of total reads classified as bacterial (%)
Percentage of reads (%)
45 187 86 156 122 183 79 57 651 1452 62 532 399 732 822 449 1132 920 720 792 191 594 880 229 182 522 159 82 989 333 342 1091 340
CHOL PCPG MESO TGCT THYM PAAD ACC UCS LUSC BRCA DLBC LIHC KIRP LGG PRAD CESC KIRC LUAD BLCA SKCM KICH COAD THCA READ UVM UCEC LAML SARC OV HNSC GBM STAD ESCA

E

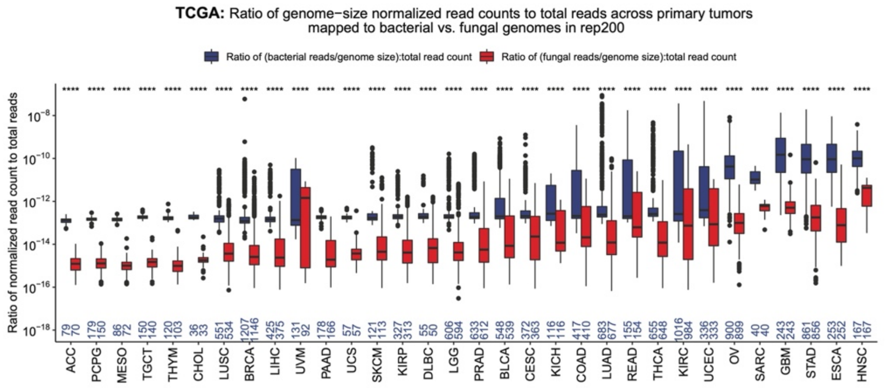

F

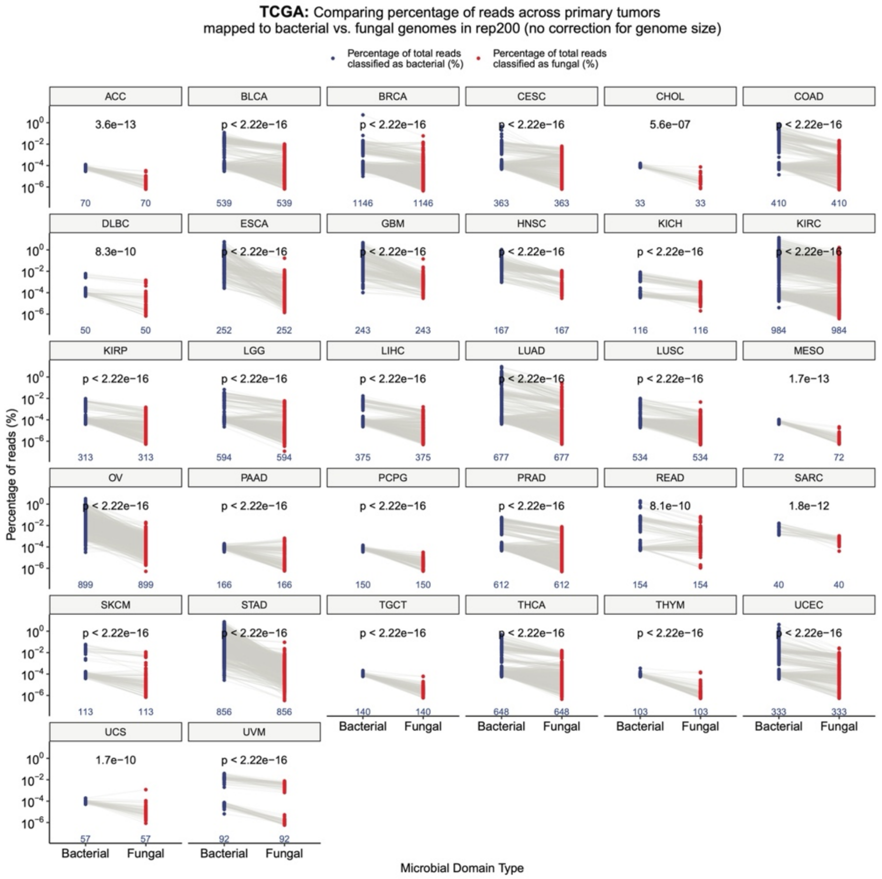

Data S1.2. Distribution of pan-microbial and bacterial nucleic acids across TCGA cancer types and the comparison of genome-normalized fungal versus bacterial proportions

(**A**) Percentage of reads in TCGA primary tumors mapped to all microbial genomes in the rep200 database versus unmapped (blue) and total (red) reads in the concomitant bam files. One-way ANOVA results showed significant variation between cancer types for microbial percentages of unmapped ($F=29.42$, $p=7.84\times10^{-165}$) and mapped ($F=25.10$, $p=1.17\times10^{-138}$).

(**B**) Percentage of reads in TCGA across all sample types mapped to all microbial genomes in the rep200 database versus unmapped (blue) and total (red) reads in the concomitant bam files. One-way ANOVA results showed significant variation between cancer types for microbial percentages of unmapped ($F=35.93$, $p=1.06\times10^{-212}$) and mapped ($F=15.42$, $p=1.27\times10^{-82}$).

(**C**) Percentage of reads in TCGA primary tumors mapped to bacterial genomes in the rep200 database versus unmapped (blue) and total (red) reads in the concomitant bam files. One-way ANOVA results showed significant variation between cancer types for bacterial percentages of unmapped ($F=26.74$, $p=1.31\times10^{-148}$) and mapped ($F=25.56$, $p=1.84\times10^{-141}$).

(**D**) Percentage of reads in TCGA across all sample types mapped to bacterial genomes in the rep200 database versus unmapped (blue) and total (red) reads in the concomitant bam files. One-way ANOVA results showed significant variation between cancer types for bacterial percentages of unmapped ($F=31.32$, $p=1.29\times10^{-183}$) and mapped ($F=15.25$, $p=1.50\times10^{-81}$).

(**E**) Genome size-normalized ratios of bacterial (blue) or fungal (red) read proportions between cancer types in TCGA primary tumors. Reads were mapped to fungal and bacterial genomes in the rep200 database, and total read count was calculated using the samtools idxstats function of the concomitant bam files. Two-sided Wilcoxon tests are used for each cancer type fungal versus bacterial read percentage comparison; ****, $p<0.001$. This analysis is the counterpart of Figure 1B, which does not account for differences in genome sizes for fungi versus bacteria but compares using direct percentages of total reads.

(**F**) Comparison of the paired percentage of fungal and bacterial reads out of total reads in concomitant bam files in TCGA primary tumors. P-value inset on plots from paired Wilcoxon signed-rank test. Sample size inset in blue text. This is the paired version of the analysis shown in Figure 1B.

(**A-F**) Y-axis is shown on a log scale. Samples sizes are inset in blue and reflect samples with non-zero microbial reads for that particular domain (fungi or bacteria). Samples sizes may vary slightly between bacterial and fungal data when samples had bacteria but no fungi.

(**A, B, C, D, E**) Box plots show median (line), 25th and 75th percentiles (box), and 1.5× the interquartile range (IQR, whiskers).

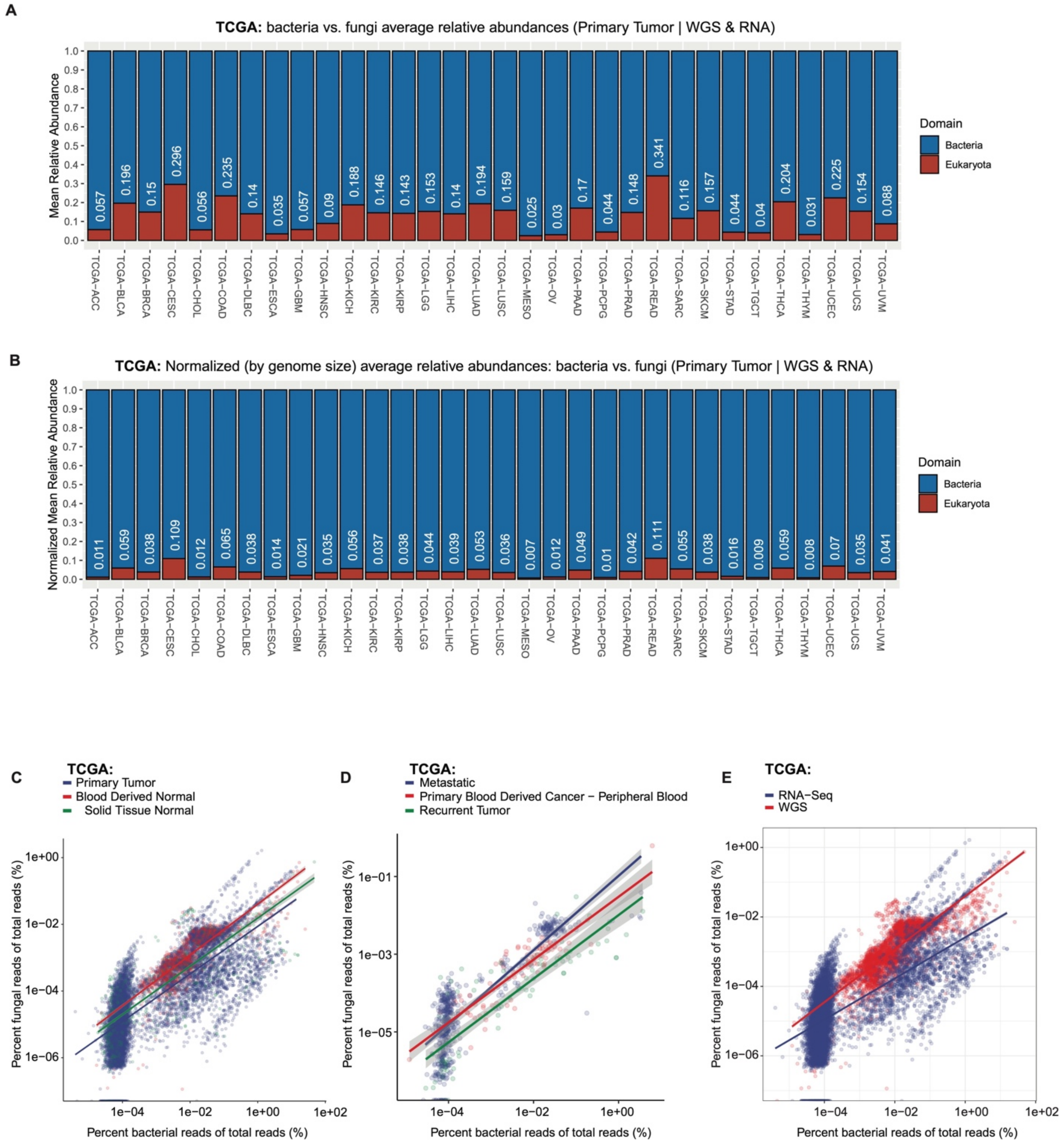
A
TCGA: bacteria vs. fungi average relative abundances (Primary Tumor | WGS & RNA)
Mean Relative Abundance
1.0
0.9
0.8
0.7
0.6
0.5
0.4
0.3
0.2
0.1
0.0
0.057
0.196
0.15
0.296
0.056
0.235
0.14
0.035
0.057
0.09
0.188
0.146
0.143
0.153
0.14
0.194
0.159
0.025
0.03
0.17
0.044
0.148
0.341
0.116
0.157
0.044
0.04
0.204
0.031
0.225
0.154
0.088
TCGA−ACC
TCGA−BLCA
TCGA−BRCA
TCGA−CESC
TCGA−CHOL
TCGA−COAD
TCGA−DLBC
TCGA−ESCA
TCGA−GBM
TCGA−HNSC
TCGA−KICH
TCGA−KIRC
TCGA−KIRP
TCGA−LGG
TCGA−LIHC
TCGA−LUAD
TCGA−LUSC
TCGA−MESO
TCGA−OV
TCGA−PAAD
TCGA−PCPG
TCGA−PRAD
TCGA−READ
TCGA−SARC
TCGA−SKCM
TCGA−STAD
TCGA−TGCT
TCGA−THCA
TCGA−THYM
TCGA−UCEC
TCGA−UCS
TCGA−UVM
Domain
Bacteria
Eukaryota
B
TCGA: Normalized (by genome size) average relative abundances: bacteria vs. fungi (Primary Tumor | WGS & RNA)
Normalized Mean Relative Abundance
1.0
0.9
0.8
0.7
0.6
0.5
0.4
0.3
0.2
0.1
0.0
0.011
0.059
0.038
0.109
0.012
0.065
0.038
0.014
0.021
0.035
0.056
0.037
0.038
0.044
0.039
0.053
0.036
0.007
0.012
0.049
0.01
0.042
0.111
0.055
0.038
0.016
0.009
0.059
0.008
0.07
0.035
0.041
TCGA−ACC
TCGA−BLCA
TCGA−BRCA
TCGA−CESC
TCGA−CHOL
TCGA−COAD
TCGA−DLBC
TCGA−ESCA
TCGA−GBM
TCGA−HNSC
TCGA−KICH
TCGA−KIRC
TCGA−KIRP
TCGA−LGG
TCGA−LIHC
TCGA−LUAD
TCGA−LUSC
TCGA−MESO
TCGA−OV
TCGA−PAAD
TCGA−PCPG
TCGA−PRAD
TCGA−READ
TCGA−SARC
TCGA−SKCM
TCGA−STAD
TCGA−TGCT
TCGA−THCA
TCGA−THYM
TCGA−UCEC
TCGA−UCS
TCGA−UVM
Domain
Bacteria
Eukaryota
C
TCGA:
Primary Tumor
Blood Derived Normal
Solid Tissue Normal
Percent fungal reads of total reads (%)
1e+00
1e−02
1e−04
1e−06
1e−04
1e−02
1e+00
1e+02
Percent bacterial reads of total reads (%)
D
TCGA:
Metastatic
Primary Blood Derived Cancer − Peripheral Blood
Recurrent Tumor
Percent fungal reads of total reads (%)
1e−01
1e−03
1e−05
1e−04
1e−02
1e+00
Percent bacterial reads of total reads (%)
E
TCGA:
RNA−Seq
WGS
Percent fungal reads of total reads (%)
1e+00
1e−02
1e−04
1e−06
1e−04
1e−02
1e+00
1e+02
Percent bacterial reads of total reads (%)

F

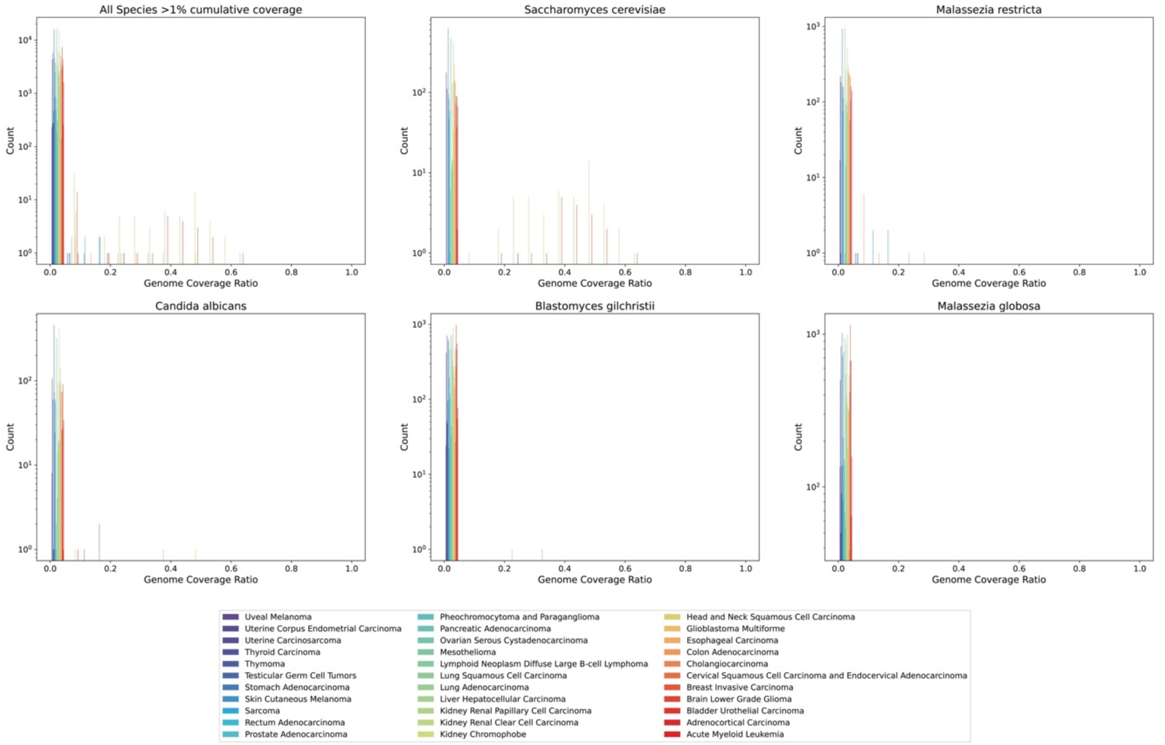

G

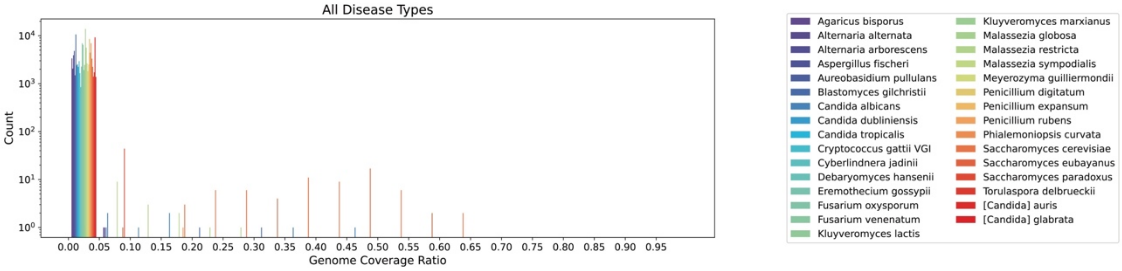

H

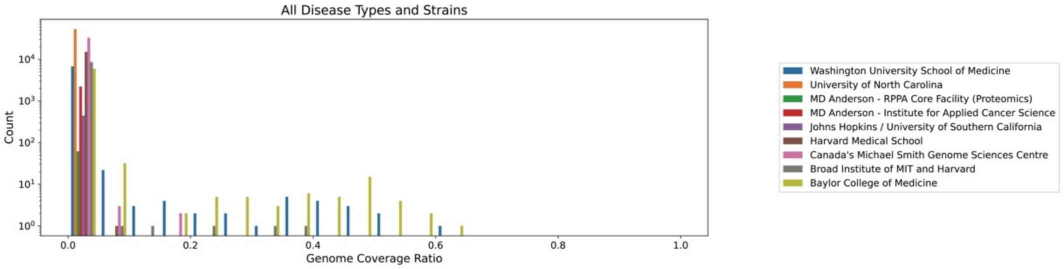

Data S1.3. Comparison of pan-cancer fungal and bacterial read proportions in TCGA

(**A**) Comparison of averaged relative abundances across TCGA cancer types within primary tumors for both WGS and RNA-Seq samples. Raw data of all hits against the rep200 database were subset to bacteria and fungi, and then relative abundances were calculated and averaged within groups, wherein each group comprised all primary tumor samples within a given cancer type. Thus, “Eukaryota” only reflects fungi in this plot. The white numbers reflect average relative

abundances of fungi per cancer type, and the bacterial average relative abundances per cancer type can be calculated by subtracting these values from 1. The means of the average fungal and bacterial relative abundances across all cancer types were 13.3% and 86.7%, respectively.

(**B**) Comparison of genome size-normalized, averaged relative abundances across TCGA cancer types within primary tumors for both WGS and RNA-Seq samples. Raw data of all hits against the rep200 database were subset to bacteria and fungi, followed by dividing all counts across all samples by their respective genome sizes. Normalized relative abundances were then calculated and averaged within groups, wherein each group comprised all primary tumor samples within a given cancer type. "Eukaryota" only reflects fungi in this plot. The white numbers reflect average relative abundances of fungi per cancer type, and the bacterial average relative abundances per cancer type can be calculated by subtracting these values from 1. The means of the genome size-normalized, average fungal and bacterial relative abundances across all cancer types were 4% and 96%, respectively.

(**C**) Log_{10}-scaled scatter plot of the percent of fungal versus bacterial reads with respect to total reads in the concomitant bam files in primary tumor, blood derived normal, and solid tissue normal (NAT) samples in TCGA. Linear regression lines are overlaid on the scatter plot, colored by the respective TCGA sample type. Non-parametric Spearman correlation testing revealed significant associations between proportions of fungal and bacterial reads: Primary tumor, $\rho=0.76$, $t=6.4\times10^{10}$, $p<2.2\times10^{-308}$; blood derived normal, $\rho=0.85$, $t=1.6\times10^{8}$, $p\leq2.2\times10^{-308}$; solid tissue normal, $\rho=0.83$, $t=5.1\times10^{7}$, $p<2.2\times10^{-308}$. Note that "normal" in blood derived normal and solid tissue normal refers to germline tissue samples from cancer patients; they are not samples from truly healthy individuals.

(**D**) Log_{10}-scaled scatter plot of the percent of fungal versus bacterial reads with respect to total reads in the concomitant bam files in metastatic, primary blood cancer (leukemic), and recurrent tumor samples in TCGA. Linear regression lines are overlaid on the scatter plot, colored by the respective TCGA sample type. Non-parametric Spearman correlation testing revealed significant associations between proportions of fungal and bacterial reads: Metastatic (as defined by TCGA), $\rho=0.76$, $t=6.3\times10^{6}$, $p=1.7\times10^{-100}$; primary blood derived cancer peripheral blood (as defined by TCGA), $\rho=0.81$, $t=1.7\times10^{4}$, $p<2.2\times10^{-308}$; recurrent tumor (as defined by TCGA), $\rho=0.88$, $t=1.4\times10^{4}$, $p=1.58\times10^{-29}$.

(**E**) Log_{10}-scaled scatter plot of the percent of fungal versus bacterial reads with respect to total reads in the concomitant bam files. Linear regression lines are overlaid on the scatter plot, colored by the respective experimental strategy. Non-parametric Spearman correlation testing revealed significant associations between proportions of fungal and bacterial reads regardless of experimental strategy: WGS, $\rho=0.82$, $t=3.3\times10^{9}$, $p<2.2\times10^{-308}$; RNA-Seq, $\rho=0.63$, $t=7.7\times10^{10}$, $p<2.2\times10^{-308}$.

(**F-G**) Histograms of per-sample fungal genome coverages for the 31 high coverage fungi or top five covered fungi, colored by (**F**) TCGA cancer type or (**G**) fungal species.

(**H**) Histograms of per-sample fungal genome coverages for the 31 high coverage fungi, colored by TCGA sequencing centers.

(**F-H**) Data for these plots derive from Table S4.2.

(**C, D, E**) Gray ribbons around regression lines denote 95% confidence intervals.

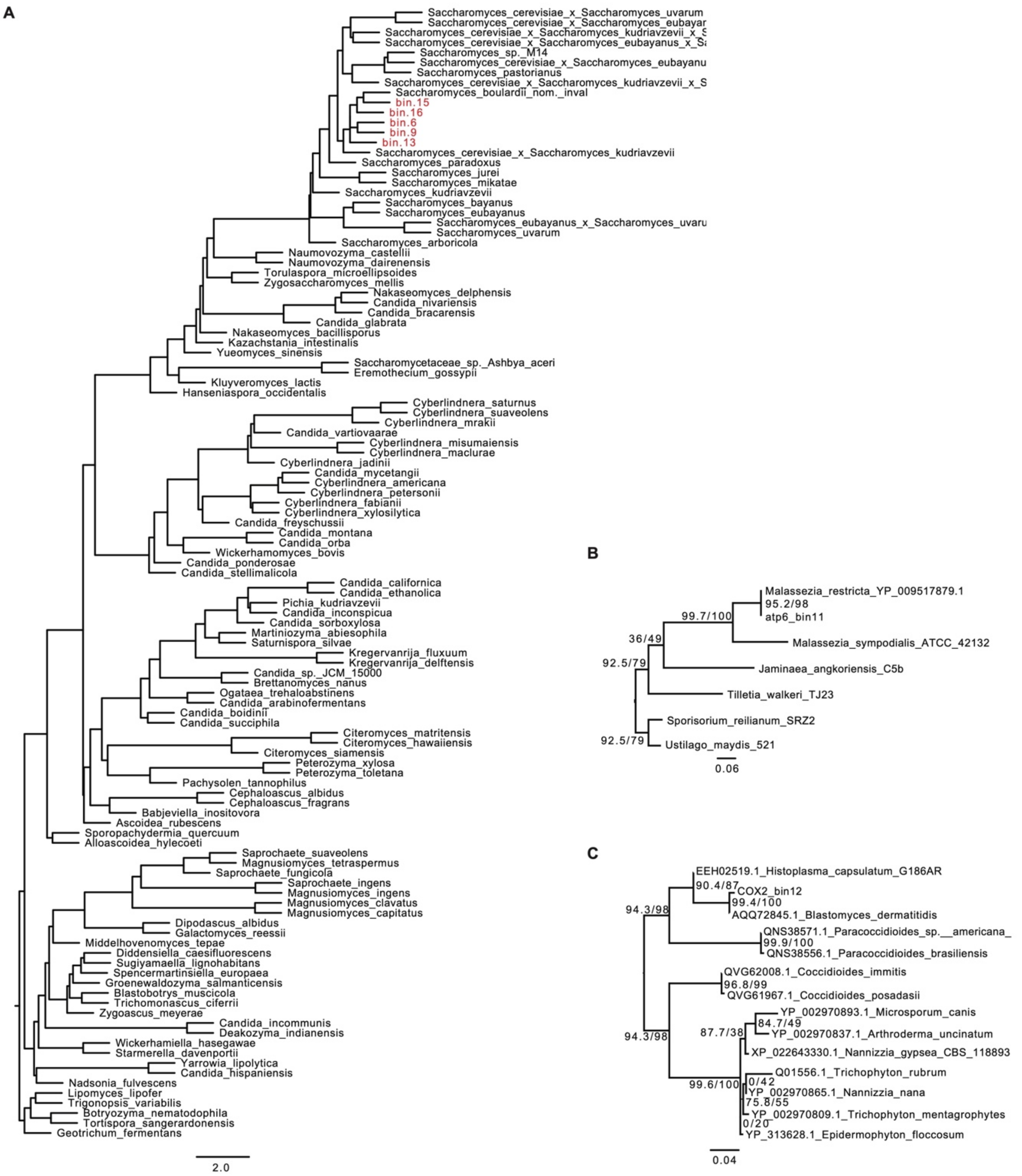

Data S1.4. Phylogenomics of several metagenome-assembled fungal bins from TCGA non-human data

(**A**) Phylogenomics of fungal bins larger than 85 kilobases shown using BUSCO across an orthogonal set of 2,623 fungal genomes from NCBI (see STAR Methods for more details). This panel complements the smaller set of comparative fungi shown in Figure 1D. All bins were placed within the *Saccharomyces* genus and are colored red.

(**B**) Phylogenomic placement of a fungal bin less than 85 kilobases found to be closest the fungal mitochondrially-encoded ATP synthase membrane subunit 6 (ATP6) locus of *Malassezia restricta* (see STAR Methods for more details).

(**C**) Phylogenomic placement of a fungal bin less than 85 kilobases found to be closest the fungal mitochondrially-encoded cyclooxygenase-2 (COX2) locus of *Blastomyces dermatitidis* (see STAR Methods for more details).

A

WIS

Taxonomic rank	# Reads	Percent (%)
Kingdom	59,919,467	100
Phylum	59,412,527	99.15
Class	59,200,179	98.8
Order	59,035,172	98.52
Family	54,747,190	91.37
Genus	53,154,728	88.71
Species	42,155,685	70.35

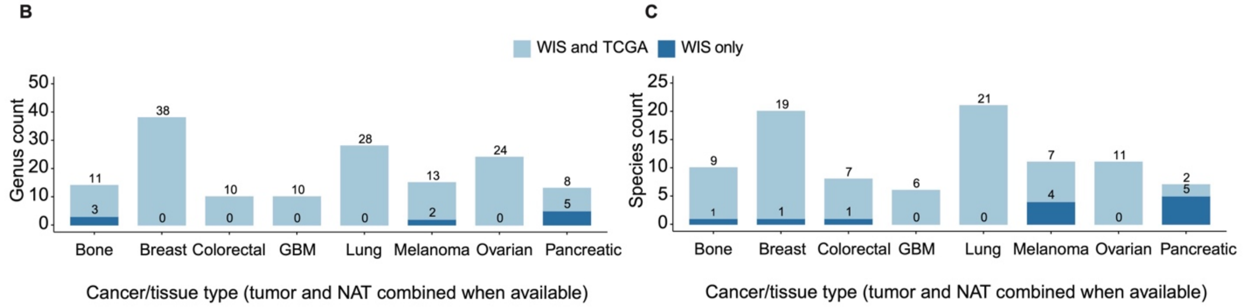

Data S1.5. ITS2 classification and taxa overlap between WIS and TCGA cohorts

(**A**) Number and percent of the fungal reads that were classified to each taxonomic level in the WIS cohort. All fungal reads were included in this analysis before flooring and normalization.

(**B-C**) Overlap at (**B**) genus-level and (**C**) species level per shared cancer type of fungi found in the WIS cohort and the TCGA cohort shown as a stacked bar plot. This analysis was restricted by the overlap of the full rep200 database (i.e., not the measured fungal hits by genome alignment) and the WIS cohort data, which represented the maximum possible overlap between the two datasets. Thus, the "Weizmann only" (dark blue) data represents fungal genera contained in the rep200 database and found in the WIS cohort but not identified in the TCGA for that particular cancer type. Conversely, the "Found in Weizmann and TCGA" (light blue) denotes fungal genera contained in the rep200 database that was found in both the WIS and TCGA cohorts for a given cancer type. Only shared cancer types between the two cohorts are shown. Tumor and NAT samples were combined when available. Fungal genera exclusively found in TCGA are not shown here since TCGA had no contamination controls and generally-speaking their numbers were higher than the total numbers reflected here. Abbreviations: "Colorectal" combined TCGA COAD and READ studies; "lung" combined TCGA "LUAD" and "LUSC" studies; "bone" denotes the TCGA "SARC" study.

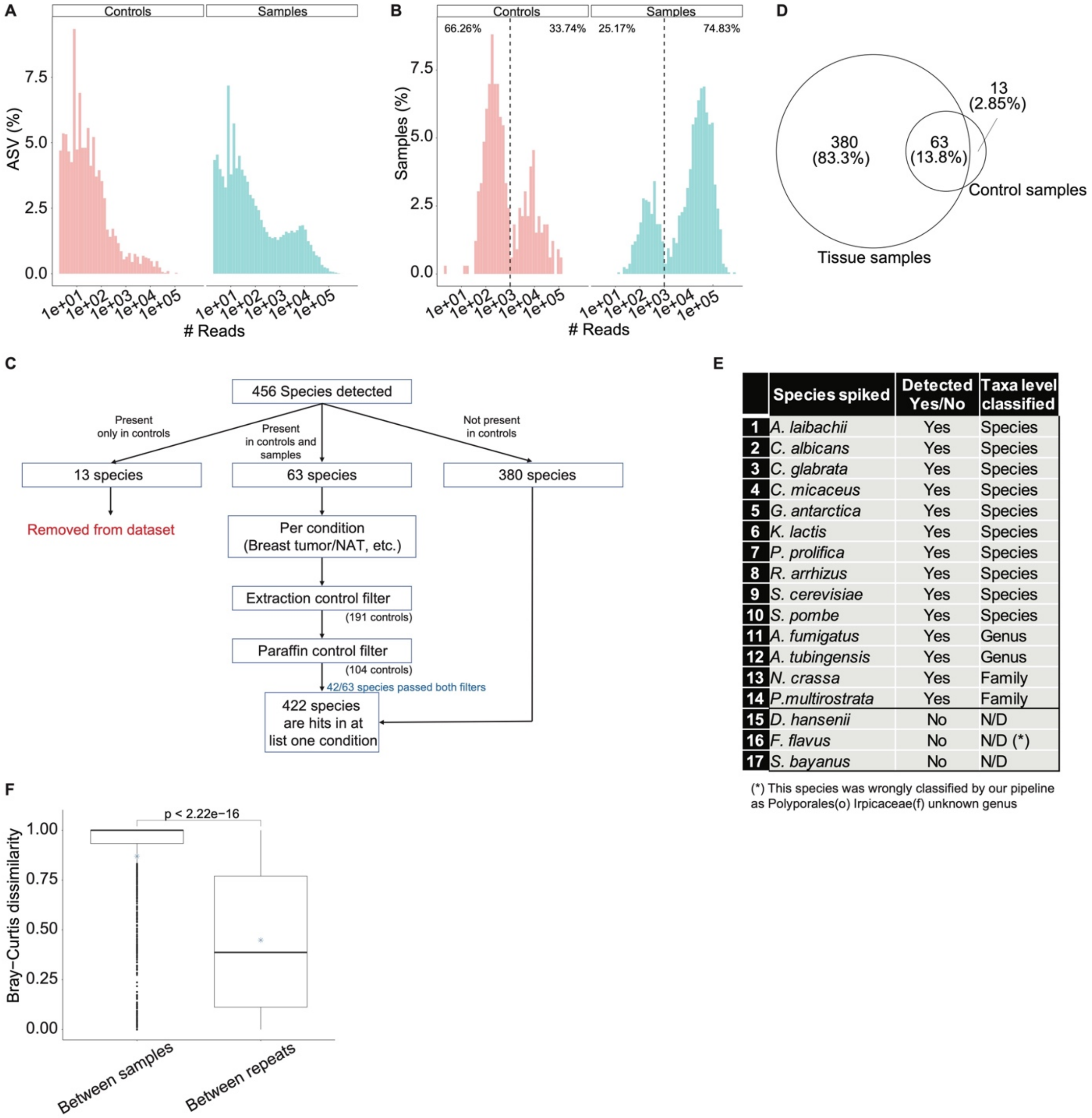

	Species spiked	Detected Yes/No	Taxa level classified
1	*A. laibachii*	Yes	Species
2	*C. albicans*	Yes	Species
3	*C. glabrata*	Yes	Species
4	*C. micaceus*	Yes	Species
5	*G. antarctica*	Yes	Species
6	*K. lactis*	Yes	Species
7	*P. prolifica*	Yes	Species
8	*R. arrhizus*	Yes	Species
9	*S. cerevisiae*	Yes	Species
10	*S. pombe*	Yes	Species
11	*A. fumigatus*	Yes	Genus
12	*A. tubingensis*	Yes	Genus
13	*N. crassa*	Yes	Family
14	*P.multirostrata*	Yes	Family
15	*D. hansenii*	No	N/D
16	*F. flavus*	No	N/D (*)
17	*S. bayanus*	No	N/D

Data S1.6. ITS2 sequencing pipeline development

(A) Normalized histograms of the number of reads per ASV per sample in control samples (extraction controls and paraffin controls) versus all other samples.

(B) Normalized histograms of the total number of fungal reads before flooring and normalization per sample in control samples (extraction controls and paraffin controls) versus all other samples.

(C) Schematic illustration of the decontamination workflow applied to WIS ITS2 to flag and remove contaminant species.

(D) Venn diagram of the overlap between all species before hit calling (after flooring and normalization), as detected in control samples and tissue samples.

(E) Table showing fungal species in mock samples that were detected by the ITS2 sequencing pipeline. 1.9×10^{-5} ng of DNA from each of 17 fungal species were pooled together and spiked into

100 ng of human DNA. Detection status and taxonomy level of classification are depicted in the table.

(**F**) Box plot of Bray-Curtis dissimilarity scores comparing the dissimilarity between the fungal composition in pairs of samples (both tumor and NAT) from the same tissue type versus the dissimilarity between the fungal composition in sequencing technical repeats of the same samples. Samples represent breast (tumors, n=17; NAT, n=16), colon (tumors, n=4; NAT, n=5) and lung (tumors, n=20; NAT, n=20) tissues. DNA from each sample was amplified and sequenced in triplicates. Two-sided T-test was performed; p-value is depicted in the plot. The boxes cover the first and third quartiles. The whiskers are drawn to the extreme values, but no more than 1.5×IQR (interquartile range). The lines depict the median.
