# Supplementary material for: Pan-cancer analyses reveal cancer-type-specific fungal ecologies and bacteriome interactions
Source: Cell. 2022 Sep 29;185(20):3789–3806.e17. doi: 10.1016/j.cell.2022.09.005 (PMC9567272; doi:10.1016/j.cell.2022.09.005)
Supplement: Data S2. Fungal imaging in human tumors, related to Figure 2 and STAR Methods [file mmc10.pdf]

Pan-cancer analyses reveal cancer type-specific fungal ecologies and bacteriome interactions

DATA S2

Fungal imaging in human tumors, related to **Figure 2** and **STAR Methods**.

Table of Contents

Data S2.1. Validation of fungal staining methods on positive control slides 2

Data S2.2. Detection of canonical patterns of fungi in tumors 3

Data S2.3. Visualization of fungi in human tumors 5

Data S2.4. Visualization of fungi in human tumors 6

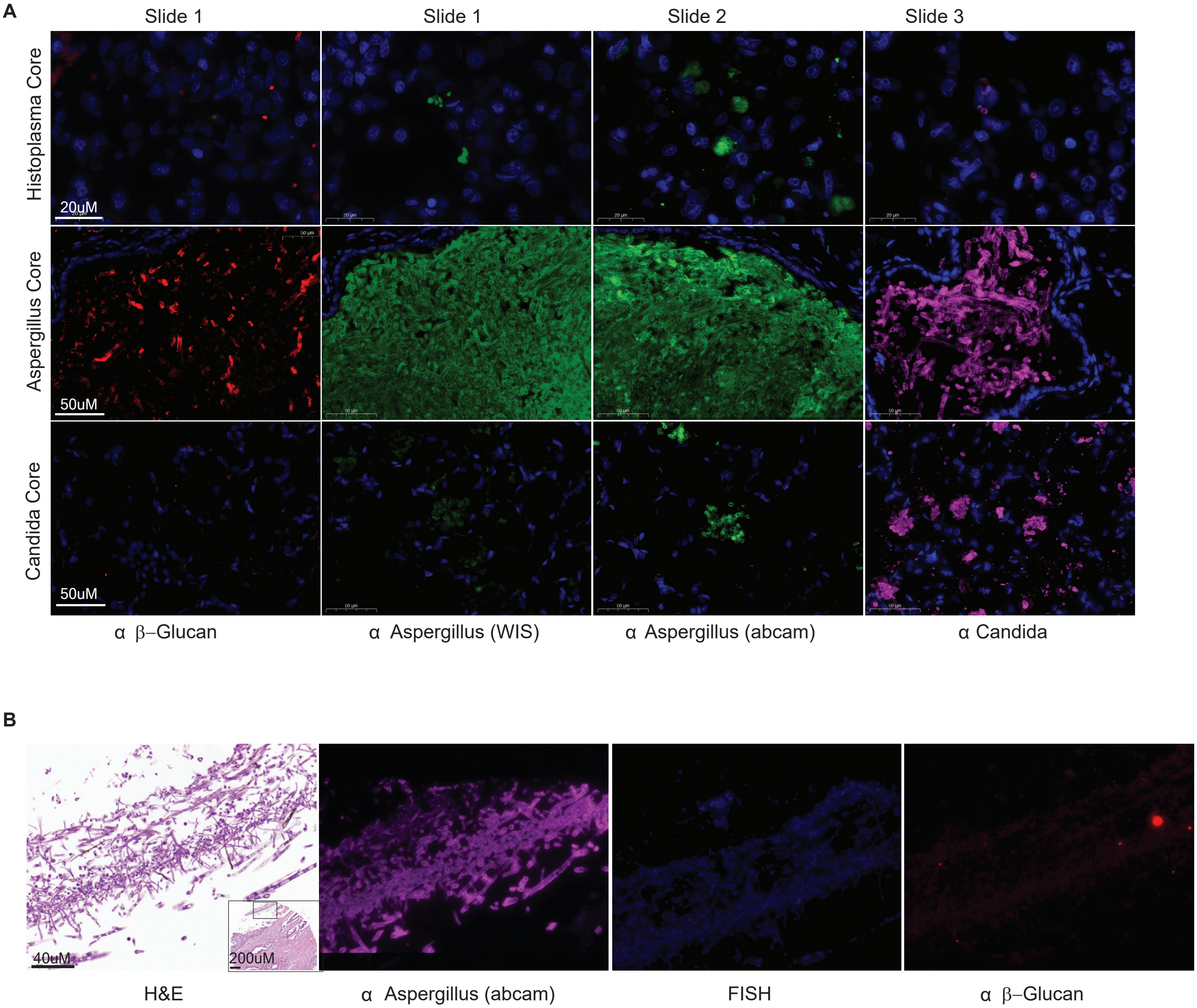

Data S2.1. Validation of fungal staining methods on positive control slides

(**A**) FFPE slides from tissues infected with *Histoplasma* (upper panel), *Aspergillus* (middle panel) and *Candida* (lower panel), were stained with antibodies against β-glucan, *Aspergillus* (two antibodies), and *Candida*. Slide 1 was stained with antibodies against both β-glucan (red) and *Aspergillus* (green, abcam); slides 2 and 3 were stained with anti-*Aspergillus* and anti-*Candida*, respectively. Upper panel scale bar 20 µm, middle and lower panels scale bar 50 µm.

(**B**) Consecutive slides from a FFPE tumor block that was found to be contaminated with fungi in the paraffin (and not the tissue) were stained with hematoxylin and eosin (H&E), antibodies against *Aspergillus (abcam)* and β-glucan, or with fluorescence in situ hybridization (FISH) using probes against fungal 28*S* rRNA. The hyphae were only detected by the anti-*Aspergillus* antibody.

A

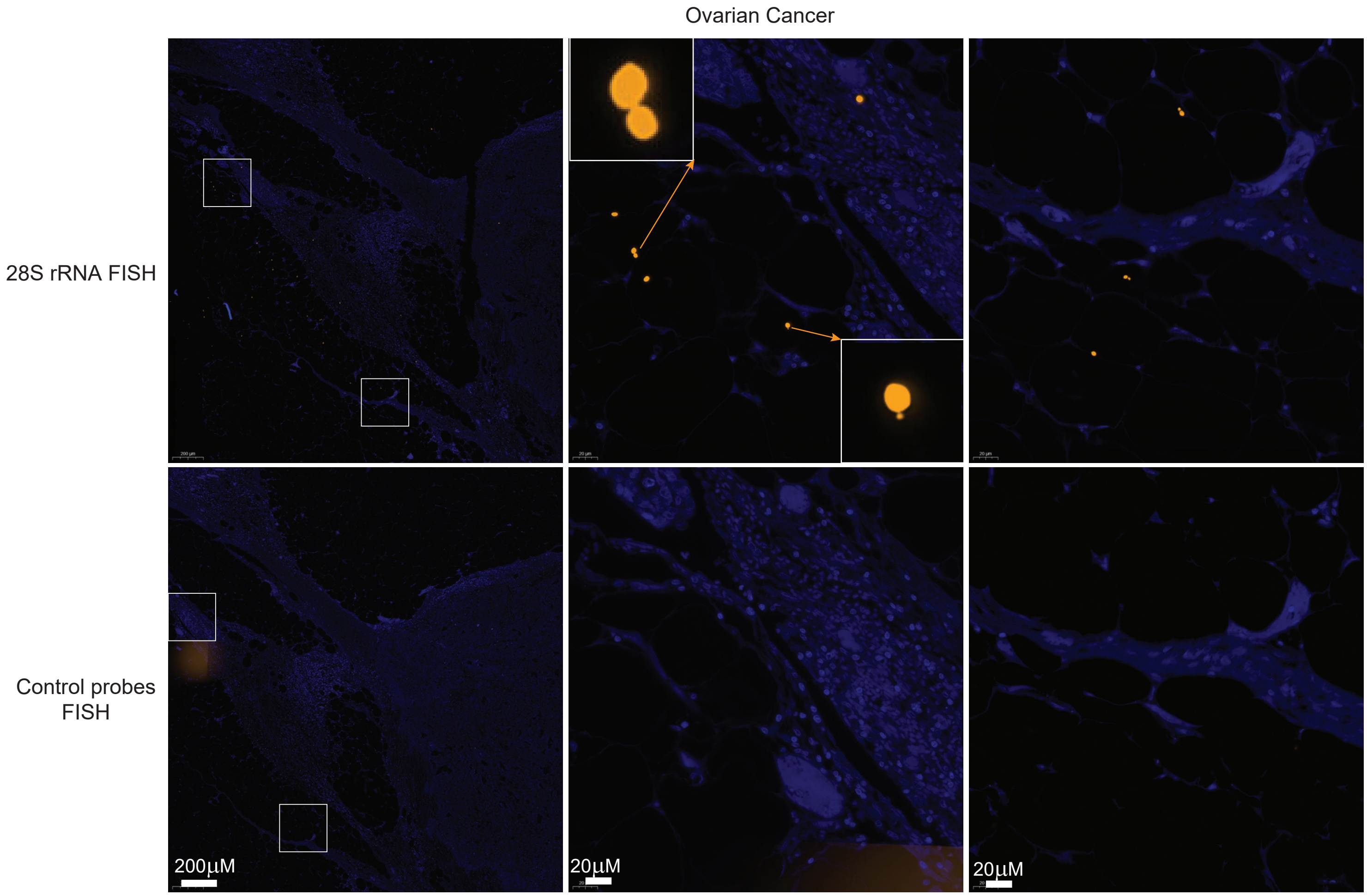
Ovarian Cancer
28S rRNA FISH
Control probes FISH
200µM
20µM
20µM

B

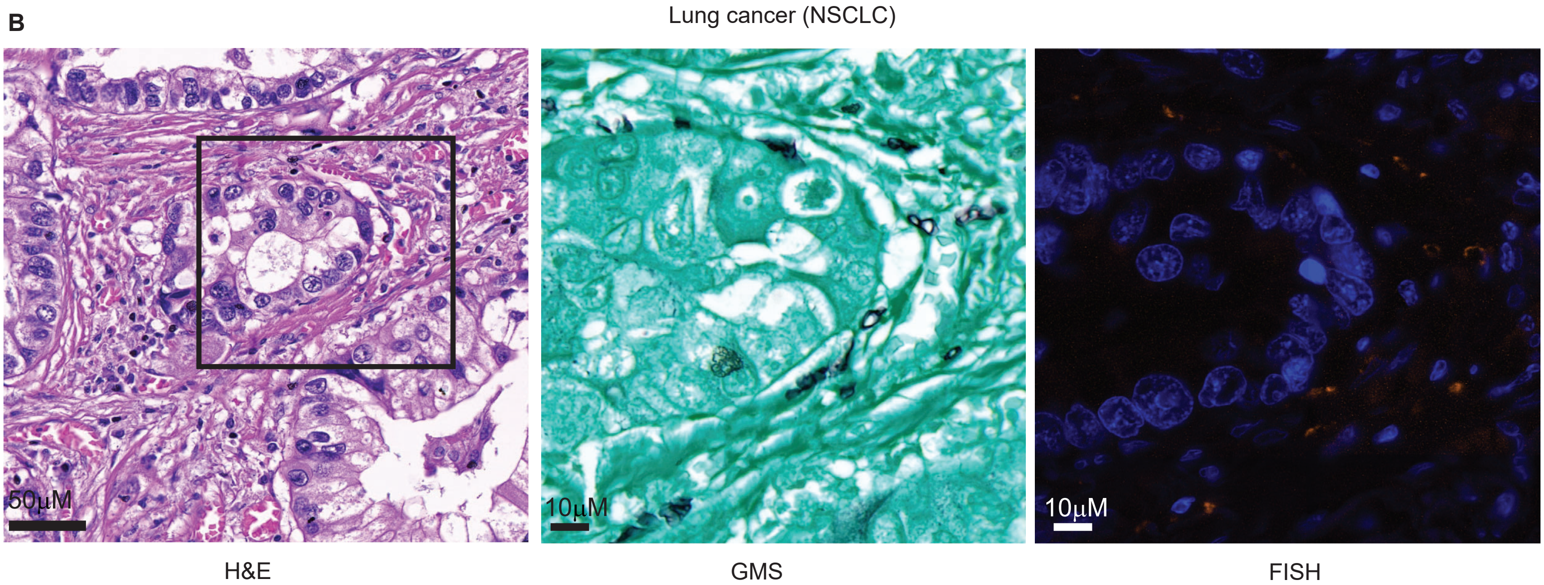
Lung cancer (NSCLC)
50µM
10µM
10µM
H&E
GMS
FISH

Data S2.2. Detection of canonical patterns of fungi in tumors

(A) Fluorescence in situ hybridization (FISH) of a human ovarian tumor in FFPE block using 3 probes against fungal 28*S* rRNA (upper panel) or 3 scrambled control probes (lower panel) (see STAR Methods for probe details). ITS2 sequencing identified *Vishniacozyma victoriae* in this tissue sample. Scale bar in the first column is 200 μm. Squares demarcates the areas presented at higher magnification in the next two columns in which the scale bar is 20 μm.

(B) A human lung tumor in FFPE block was stained with hematoxylin and eosin (H&E), Gomori methenamine silver stain (GMS), or fluorescence in situ hybridization (FISH) using the same probes as in (A). ITS2 sequencing identified *Fusarium keratoplasticum* and *Aspergillus tardicrescens* in this tissue sample. Scale bar in the first column is 50 μm. Square demarcates the area presented at higher magnification in next two columns in which scale bar is 10 μm.

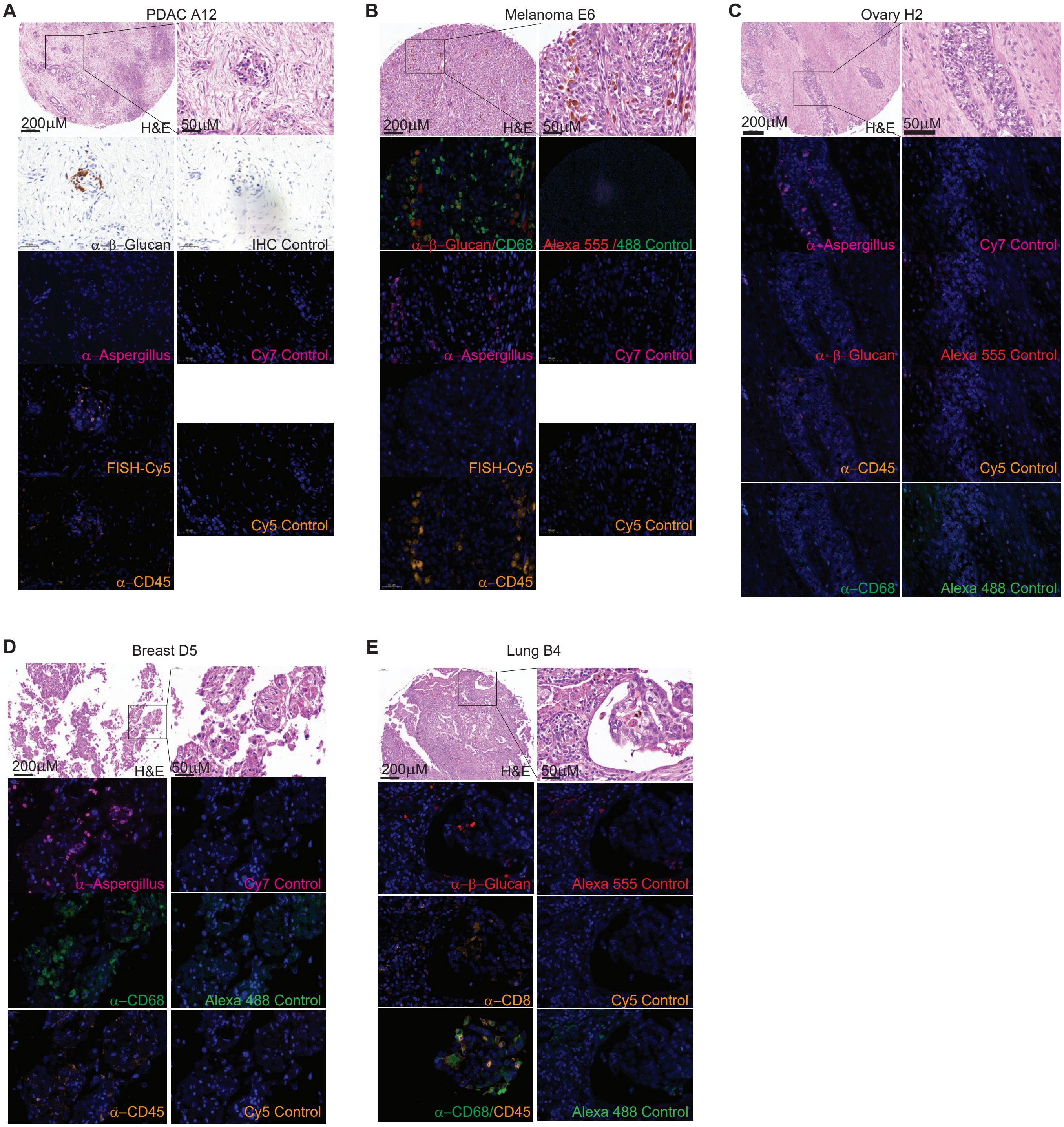

Data S2.3. Visualization of fungi in human tumors

(**A-E**) Consecutive slides from a tumor microarray of **(A)** human pancreatic adenocarcinoma, **(B)** melanoma, **(C)** ovary, **(D)** breast, and **(E)** lung cancer, that appear in Figure 2. Slides were stained with hematoxylin and eosin (H&E), or antibodies against β-glucan, *Aspergillus (abcam)*, CD45, CD68, CD8, or by fluorescence in situ hybridization (FISH) probes against fungal 28*S* rRNA. Slides were also stained with only secondary antibodies as a negative control, which appear here. (**A-E**) Scale bar for lower magnification of cores is located in the upper left corners of H&E staining panel: 200 μm. Scale bar for higher magnifications (all other panels) is 50 μm. Related to Figure 2.

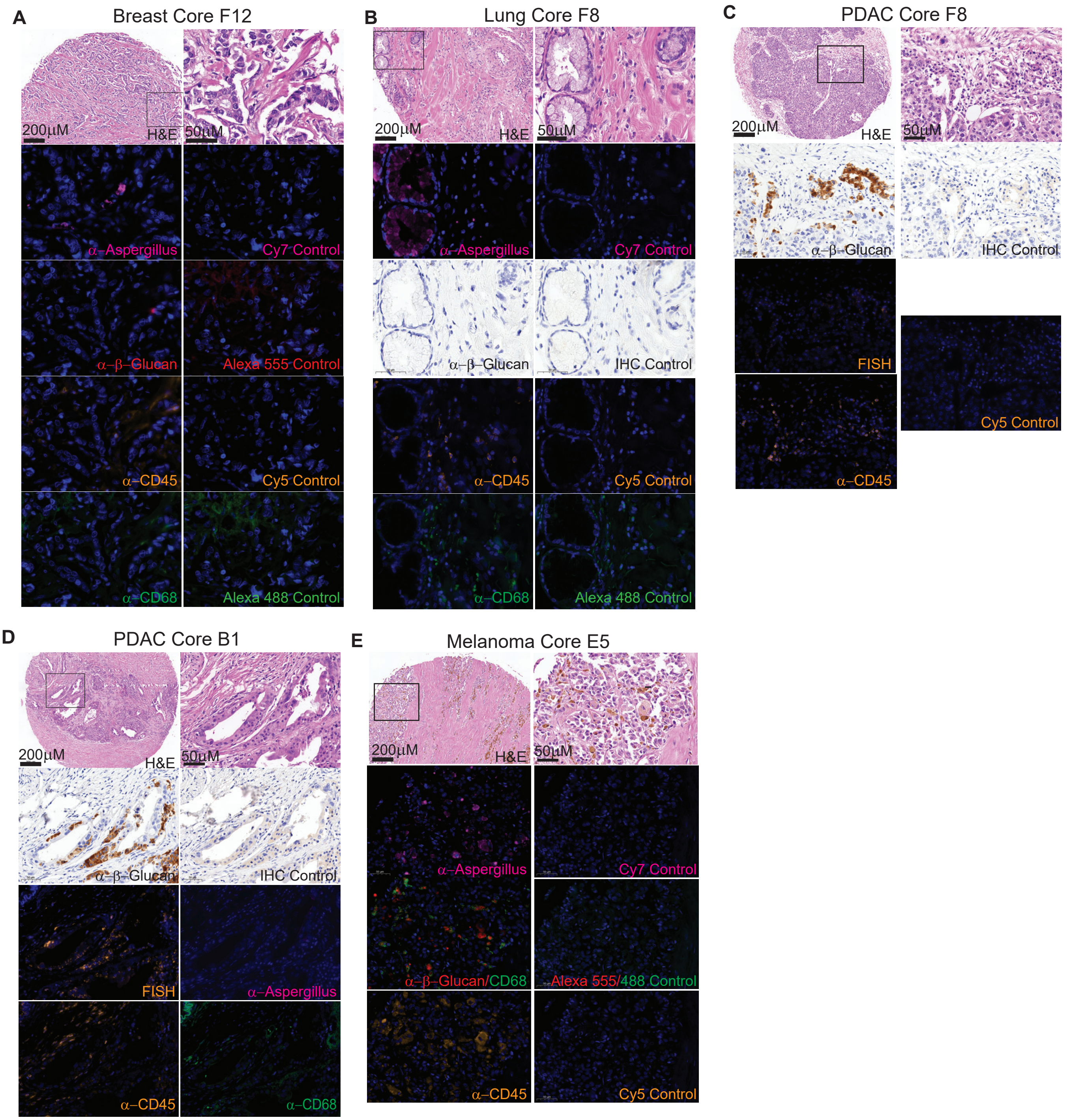

Data S2.4. Visualization of fungi in human tumors

(**A-E**) Consecutive slides from representative cores from tumor microarrays of (**A**) human breast cancer, (**B**) lung cancer, (**C-D**) pancreatic adenocarcinoma, and (**E**) melanoma, were stained with hematoxylin and eosin (H&E), antibodies against β-glucan, CD45, CD68, *Aspergillus* (abcam), CD8, or by fluorescence in situ hybridization (FISH) probes against fungal 28*S* rRNA. Slides were also stained with only secondary antibodies as a negative control. Note that in (**D**) the core used to evaluate fluorescence negative control is missing from this slide.

(**A-E**) Scale bar for low magnification: 200 µm. Square demarcates the area presented at higher magnification. Scale bar for higher magnification (all other panels): 50 µm.
