# Supplementary material for: Pan-cancer analyses reveal cancer-type-specific fungal ecologies and bacteriome interactions
Source: Cell. 2022 Sep 29;185(20):3789–3806.e17. doi: 10.1016/j.cell.2022.09.005 (PMC9567272; doi:10.1016/j.cell.2022.09.005)
Supplement: Data S3. Cancer type-specific mycobiome characteristics and NAT comparisons, related to Figure 3 [file mmc11.pdf]

Pan-cancer analyses reveal cancer type-specific fungal ecologies and bacteriome interactions

DATA S3

Cancer type-specific mycobiome characteristics and NAT comparisons, related to **Figure 3**.

Table of Contents

Data S3.1. Mycobiome richness varies across cancer types 2

Data S3.2. Classical metagenomic and clustering analyses show cancer type-specific mycobiomes and similarities between tumor versus NAT samples 6

Data S3.3. Classical metagenomic analyses show cancer type-specific mycobiomes in TCGA even after subsetting to 34 WIS-overlapping fungi or the 20-fungi Hopkins signature 9

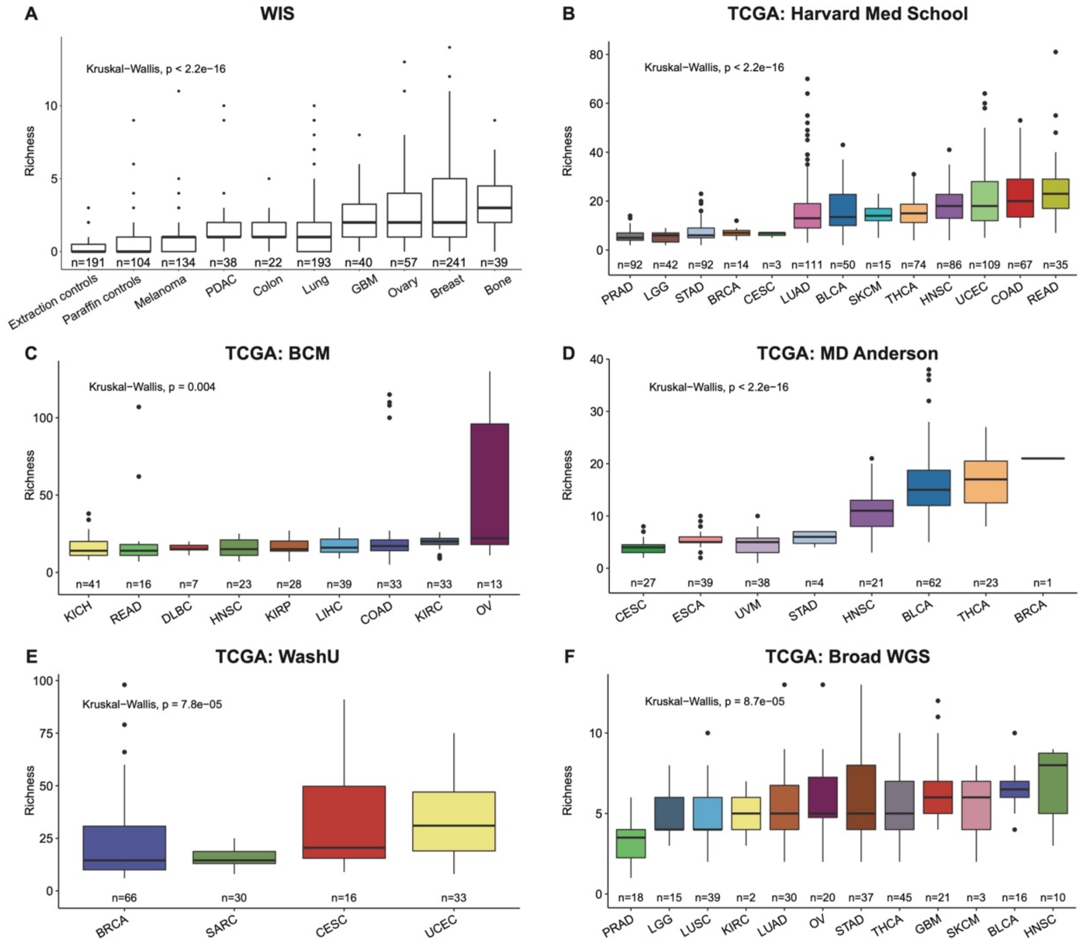

Data S3.1. Mycobiome richness varies across cancer types

(**A**) The number of species per WIS sample in different tumor types after data flooring and normalization (STAR Methods). Only fungi that passed the filtering step in any of the tumor types were included in the analysis. The boxes cover the first and third quartiles. The whiskers are drawn to the extreme values, but no more than 1.5×IQR (interquartile range). The bold horizontal lines depict the median. Kruskal-Wallis test reflects significant richness variation across cancer types.

(**B**) Alpha diversity of raw fungal data from primary tumor samples at Harvard Medical School. Data were rarefied at 5000 reads/sample (approximately the first quartile of the read/sample distribution) prior to calculating richness. Sample counts are inset below the boxplots, and samples are ordered by their median values. Kruskal-Wallis test reflects significant richness variation across cancer types.

(**C**) Alpha diversity of raw fungal data from primary tumor samples at Baylor College of Medicine (BCM). Data were rarefied at 5000 reads/sample prior to calculating richness. Sample counts are inset below the boxplots, and samples are ordered by their median values. Kruskal-Wallis test reflects significant richness variation across cancer types.

(**D**) Alpha diversity of raw fungal data from primary tumor samples at MD Anderson. Data were rarefied at 5000 reads/sample (approximately the first quartile of the read/sample distribution) prior to calculating richness. Sample counts are inset below the boxplots, and samples are ordered by their median values. Kruskal-Wallis test reflects significant richness variation across cancer types.

(**E**) Alpha diversity of raw fungal data from primary tumor samples at Washington University (WashU). Data were rarefied at 5000 reads/sample (approximately the first quartile of the read/sample distribution) prior to calculating richness. Sample counts are inset below the boxplots, and samples are ordered by their median values. Kruskal-Wallis test reflects significant richness variation across cancer types.
(**F**) Alpha diversity of raw fungal data from WGS primary tumor samples at the Broad Institute. The Broad also performed RNA-Seq on 156 glioblastoma (GBM) primary tumors and 13 recurrent tumors that are not shown here. Data were rarefied at 2000 reads/sample (approximately the first quartile of the read/sample distribution) prior to calculating richness. Sample counts are inset below the boxplots, and samples are ordered by their median values. Kruskal-Wallis test reflects significant richness variation across cancer types.
(**B-F**) TCGA RNA-Seq richness data not calculated due to ~100-fold lower read counts per sample than WGS samples. Raw data was decontaminated prior to rarefaction. Box plots show median (line), 25th and 75th percentiles (box), and 1.5× the interquartile range (IQR, whiskers).

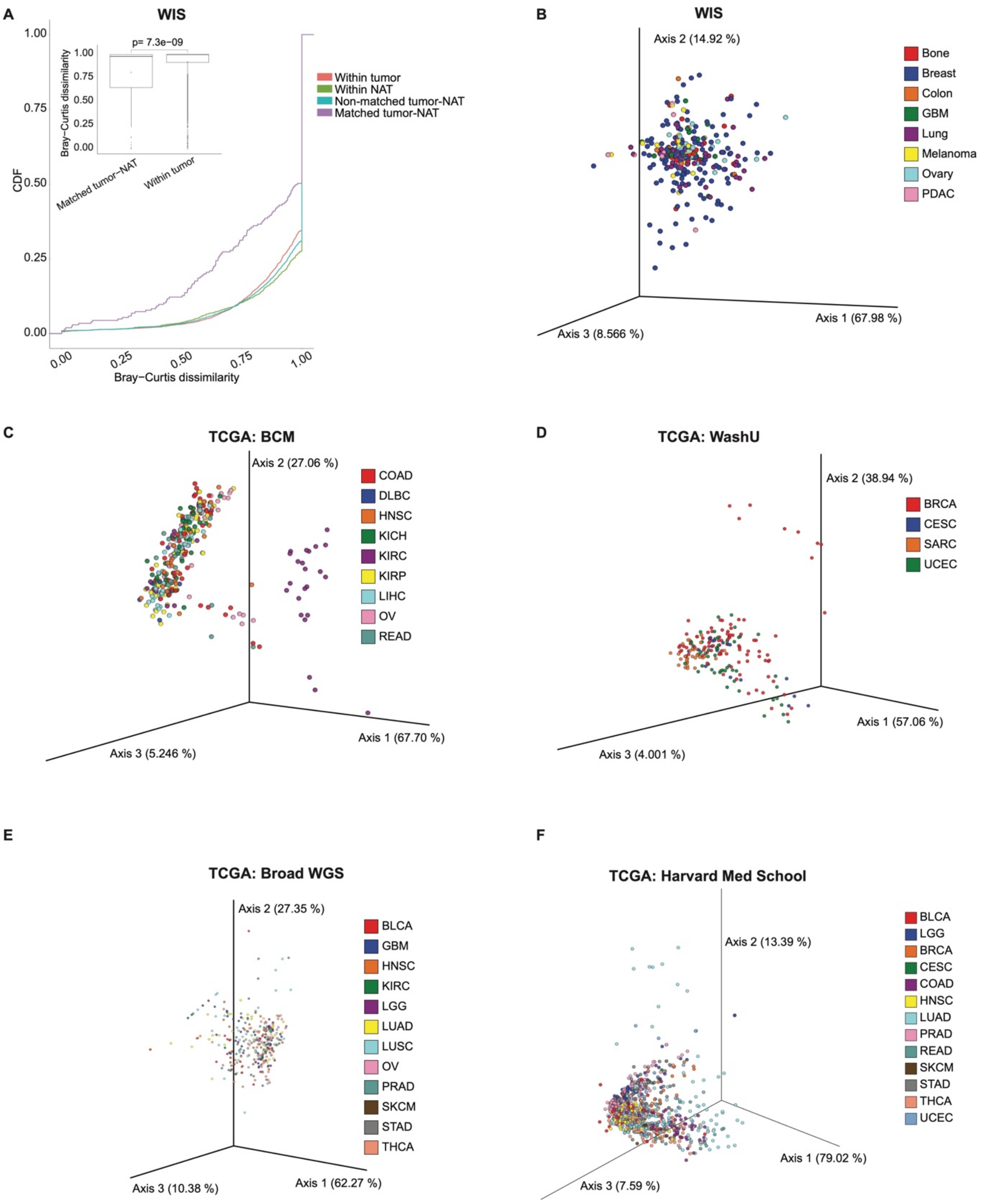
A
WIS
1.00
0.75
0.50
0.25
0.00
CDF
p= 7.3e−09
Bray−Curtis dissimilarity
Matched tumor−NAT
Within tumor
Within tumor
Within NAT
Non-matched tumor-NAT
Matched tumor-NAT
Bray−Curtis dissimilarity
B
WIS
Axis 2 (14.92 %)
Axis 1 (67.98 %)
Axis 3 (8.566 %)
Bone
Breast
Colon
GBM
Lung
Melanoma
Ovary
PDAC
C
TCGA: BCM
Axis 2 (27.06 %)
Axis 1 (67.70 %)
Axis 3 (5.246 %)
COAD
DLBC
HNSC
KICH
KIRC
KIRP
LIHC
OV
READ
D
TCGA: WashU
Axis 2 (38.94 %)
Axis 1 (57.06 %)
Axis 3 (4.001 %)
BRCA
CESC
SARC
UCEC
E
TCGA: Broad WGS
Axis 2 (27.35 %)
Axis 1 (62.27 %)
Axis 3 (10.38 %)
BLCA
GBM
HNSC
KIRC
LGG
LUAD
LUSC
OV
PRAD
SKCM
STAD
THCA
F
TCGA: Harvard Med School
Axis 2 (13.39 %)
Axis 1 (79.02 %)
Axis 3 (7.59 %)
BLCA
LGG
BRCA
CESC
COAD
HNSC
LUAD
PRAD
READ
SKCM
STAD
THCA
UCEC

G

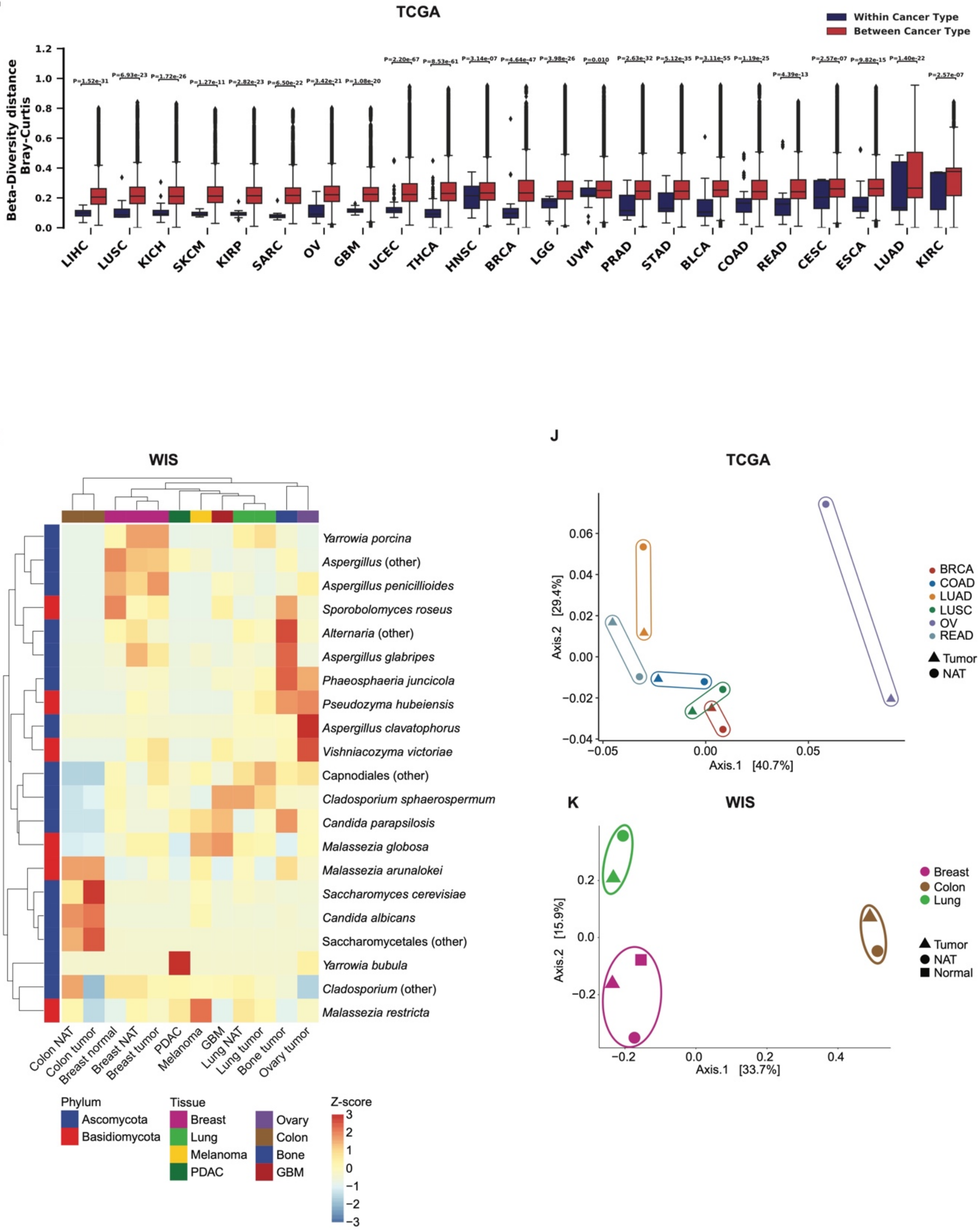
TCGA
Within Cancer Type
Between Cancer Type
Beta-Diversity distance
Bray-Curtis
P=1.52e-31
P=6.93e-23
P=1.72e-26
P=1.27e-11
P=2.82e-23
P=6.50e-22
P=3.42e-21
P=1.08e-20
P=2.20e-67
P=8.53e-61
P=3.14e-07
P=4.64e-47
P=3.98e-26
P=0.010
P=2.63e-32
P=5.12e-35
P=3.11e-55
P=1.19e-25
P=4.39e-13
P=2.57e-07
P=9.82e-15
P=1.40e-22
P=2.57e-07
LIHC
LUSC
KICH
SKCM
KIRP
SARC
OV
GBM
UCEC
THCA
HNSC
BRCA
LGG
UVM
PRAD
STAD
BLCA
COAD
READ
CESC
ESCA
LUAD
KIRC
H
WIS
Yarrowia porcina
Aspergillus (other)
Aspergillus penicillioides
Sporobolomyces roseus
Alternaria (other)
Aspergillus glabripes
Phaeosphaeria juncicola
Pseudozyma hubeiensis
Aspergillus clavatophorus
Vishniacozyma victoriae
Capnodiales (other)
Cladosporium sphaerospermum
Candida parapsilosis
Malassezia globosa
Malassezia arunalokei
Saccharomyces cerevisiae
Candida albicans
Saccharomycetales (other)
Yarrowia bubula
Cladosporium (other)
Malassezia restricta
Colon NAT
Colon tumor
Breast normal
Breast NAT
Breast tumor
PDAC
Melanoma
GBM
Lung NAT
Lung tumor
Bone tumor
Ovary tumor
Phylum
Ascomycota
Basidiomycota
Tissue
Breast
Lung
Melanoma
PDAC
Ovary
Colon
Bone
GBM
Z-score
J
TCGA
Axis.1 [40.7%]
Axis.2 [29.4%]
BRCA
COAD
LUAD
LUSC
OV
READ
Tumor
NAT
K
WIS
Axis.1 [33.7%]
Axis.2 [15.9%]
Breast
Colon
Lung
Tumor
NAT
Normal

I

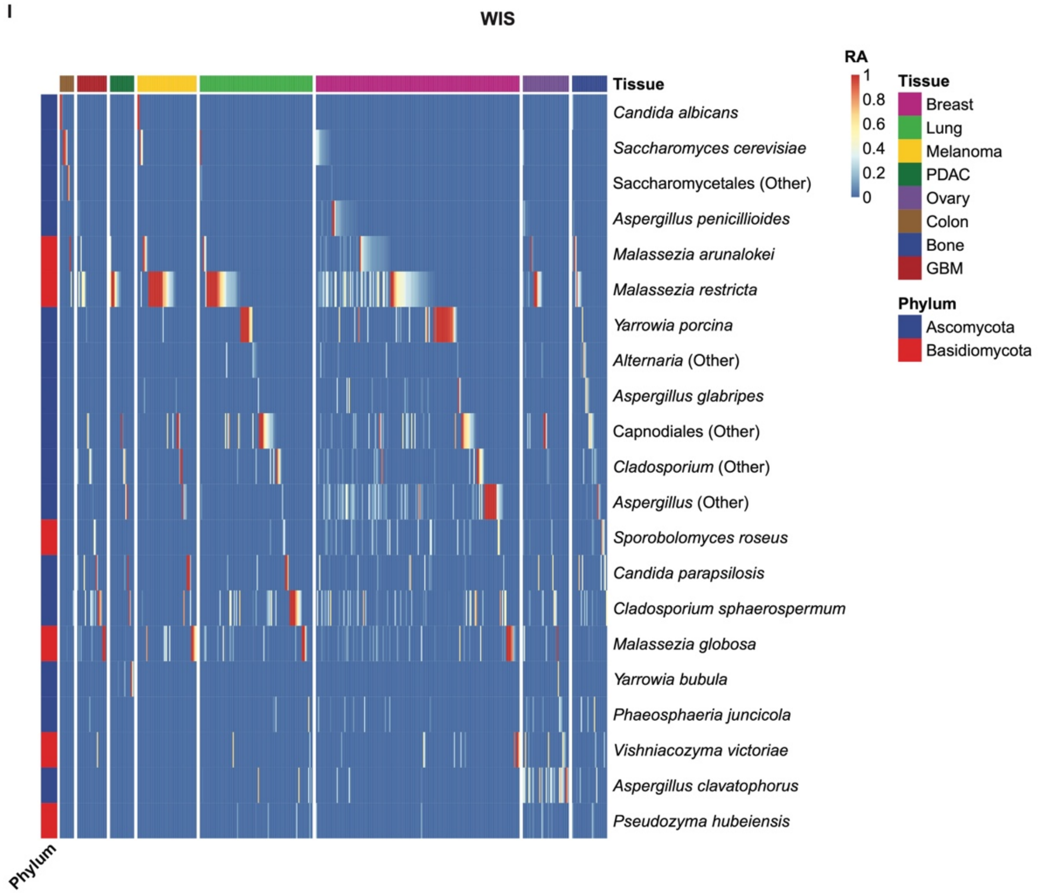

Data S3.2. Classical metagenomic and clustering analyses show cancer type-specific mycobiomes and similarities between tumor versus NAT samples

(**A**) Cumulative Distribution Function (CDF) plot of Bray-Curtis dissimilarity scores within tumor samples, within NAT samples, between tumor and NAT samples from different patients ("Non-matched tumor-NAT"), and between paired tumor and NAT samples from the same patient ("Matched tumor-NAT"). All scores of pairs were calculated within a tumor type and included only tissue types for which NAT samples were available: breast, lung and colon. Only fungi that passed the filtering step in any of the tumor types were included in the analysis. Inset: Boxplot of dissimilarity measurements between the paired samples group and each of the other three groups. Paired samples from the same patient demonstrated a reproducible, significantly more similar microbial profiles ($p=7.3\times10^{-9}$, Student's t-test). Boxplots show median, 25th and 75th percentiles, and 1.5 × IQR.

(**B-F**) Robust Aitchison distance principal coordinates analyses (RPCA PCoAs) of decontaminated fungal data on (**B**) the WIS cohort, with PERMANOVA F=1.65, R^2=0.02, p=0.029; (**C**) the Baylor College of Medicine (BCM) TCGA cohort, with PERMANOVA F=15.19, R^2=0.28, p=0.001; (**D**) the Washington University (WashU) TCGA cohort, with PERMANOVA F=5.34, R^2=0.08, p=0.001; (**E**) the Broad Institute WGS (Broad WGS) TCGA cohort, with PERMANOVA F=1.99, R^2=0.06, p=0.004; (**F**) the Harvard Medical School TCGA cohort, with PERMANOVA F=26.41, R^2=0.27, p=0.001.

(**G**) Comparisons of primary tumor beta diversity within and between all TCGA cancer types having WGS data and ≥10 samples. Inset p-values are based on Mann-Whitney U tests with FDR correction. (**H**) Unsupervised hierarchical clustering of the mean relative abundance of species that appear in over 10% of samples in at least one condition. Values represent Z-scores per row.

Only fungi that passed the filtering step in at least one of the tumor types were included in the analysis (see STAR Methods). ASVs that did not reach species level classification were aggregated by the lowest classification they received.

(**I**) Heatmap of the fungal relative abundance in the WIS cohort using species that appear in ≥10% of tumor samples. Only samples with >0 relative abundance of at least one of the species in the heatmap were included. ASVs without species level classification were aggregated by the lowest classification they received.

(**J**) Bray-Curtis PCoA of averaged relative abundances on rescaled, Voom-SNM corrected TCGA WGS and RNA-Seq data (see STAR Methods) on cancer types also found in the Weizmann cohort and with ≥10 tumors and NATs available in TCGA. Sample counts: breast NAT, n=119; breast tumor, n=1132; colon adenocarcinoma NAT, n=64; colon adenocarcinoma tumor, n=401; rectal adenocarcinoma NAT, n=14; rectal adenocarcinoma tumor, n=148; lung adenocarcinoma NAT, n=131; lung adenocarcinoma tumor, n=670; lung squamous cell NAT, n=80; lung squamous cell tumor, n=529; ovarian NAT, n=10; ovarian tumor, n=876.

(**K**) WIS cohort principal coordinate analysis (PCoA) on the Jaccard dissimilarity indices between species profiles of the different tissue types after discarding paired tumor-NAT patient samples. This analysis was done to demonstrate that the tumor-NAT clustering that was observed is not the result of high similarity between mycobiomes of samples that originate from the same patients. We have thus removed from the analysis either the tumor or the NAT samples (by random) from patients that had both sample types.

(**B-F**) All PERMANOVA analyses used 999 permutations. Note, a RPCA plot for MD Anderson primary tumor samples is shown in Figure 3D. As with richness data, TCGA RNA-Seq beta diversity was not calculated due to ~100-fold lower read counts per sample than WGS samples.

(**C-F**) TCGA cancer type abbreviations are shown.

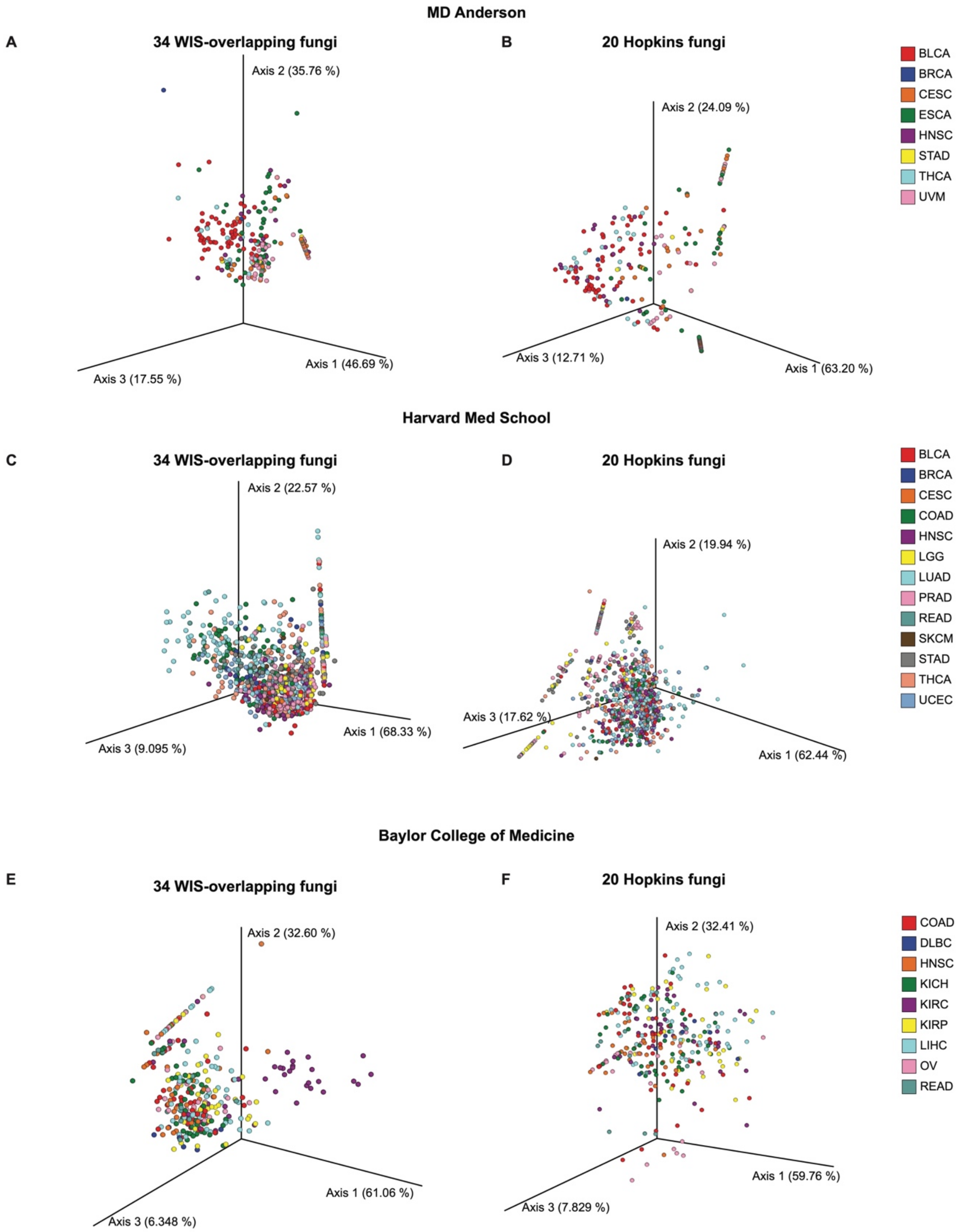
MD Anderson
A
34 WIS-overlapping fungi
Axis 2 (35.76 %)
Axis 1 (46.69 %)
Axis 3 (17.55 %)
B
20 Hopkins fungi
Axis 2 (24.09 %)
Axis 1 (63.20 %)
Axis 3 (12.71 %)
BLCA
BRCA
CESC
ESCA
HNSC
STAD
THCA
UVM
Harvard Med School
C
34 WIS-overlapping fungi
Axis 2 (22.57 %)
Axis 1 (68.33 %)
Axis 3 (9.095 %)
D
20 Hopkins fungi
Axis 2 (19.94 %)
Axis 1 (62.44 %)
Axis 3 (17.62 %)
BLCA
BRCA
CESC
COAD
HNSC
LGG
LUAD
PRAD
READ
SKCM
STAD
THCA
UCEC
Baylor College of Medicine
E
34 WIS-overlapping fungi
Axis 2 (32.60 %)
Axis 1 (61.06 %)
Axis 3 (6.348 %)
F
20 Hopkins fungi
Axis 2 (32.41 %)
Axis 1 (59.76 %)
Axis 3 (7.829 %)
COAD
DLBC
HNSC
KICH
KIRC
KIRP
LIHC
OV
READ

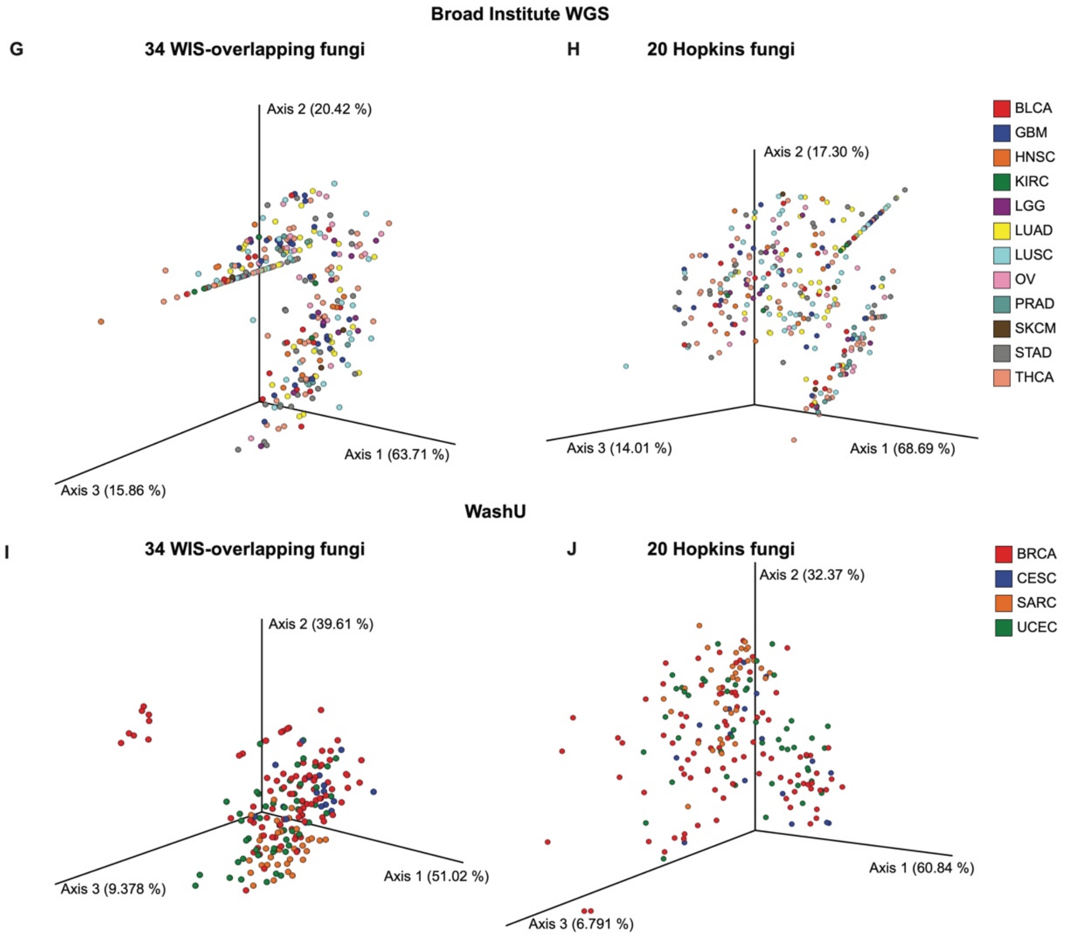

Data S3.3. Classical metagenomic analyses show cancer type-specific mycobiomes in TCGA even after subsetting to 34 WIS-overlapping fungi or the 20-fungi Hopkins signature

(**A-B**) Robust Aitchison distance principal coordinates analyses (RPCA PCoAs) on MD Anderson TCGA primary tumor samples using (**A**) 34 WIS-overlapping fungi, with PERMANOVA F=13.17, R^2=0.27, p=0.001; and (**B**) the 20-fungi Hopkins-signature, with PERMANOVA F=13.37, R^2=0.27, p=0.001.

(**C-D**) RPCA PCoAs on Harvard Medical School TCGA primary tumor samples using (**C**) 34 WIS-overlapping fungi, with PERMANOVA F=17.29, R^2=0.19, p=0.001; and (**D**) the 20-fungi Hopkins-signature, with PERMANOVA F=16.99, R^2=0.19, p=0.001.

(**E-F**) RPCA PCoAs on Baylor College of Medicine TCGA primary tumor samples using (**E**) 34 WIS-overlapping fungi, with PERMANOVA F=14.04, R^2=0.27, p=0.001; and (**F**) the 20-fungi Hopkins-signature, with PERMANOVA F=6.52, R^2=0.15, p=0.001.

(**G-H**) RPCA PCoAs on the Broad Institute WGS TCGA primary tumor samples using (**G**) 34 WIS-overlapping fungi, with PERMANOVA F=1.51, R^2=0.05, p=0.064; and (**H**) the 20-fungi Hopkins-signature, with PERMANOVA F=1.59, R^2=0.05, p=0.032.

(**I-J**) RPCA PCoAs on the Washington University (WashU) TCGA primary tumor samples using (**I**) 34 WIS-overlapping fungi, with PERMANOVA F=10.80, R^2=0.15, p=0.001; and (**J**) the 20-fungi Hopkins-signature, with PERMANOVA F=8.23, R^2=0.12, p=0.001.

(**A-J**) All PERMANOVAs used 999 permutations.
