# Supplementary material for: Pan-cancer analyses reveal cancer-type-specific fungal ecologies and bacteriome interactions
Source: Cell. 2022 Sep 29;185(20):3789–3806.e17. doi: 10.1016/j.cell.2022.09.005 (PMC9567272; doi:10.1016/j.cell.2022.09.005)
Supplement: Data S4. Fungal-bacterial-immune analyses, related to Figure 4 [file mmc12.pdf]

Pan-cancer analyses reveal cancer type-specific fungal ecologies and bacteriome interactions

DATA S4

Fungal-bacterial-immune analyses, related to **Figure 4**.

Table of Contents

Data S4.1. Analyses of fungi-bacteria co-occurrences 3

Data S4.2. Associations of multi-domain log-ratios with survival and immune response subtypes 4

Data S4.3. Associations of mycotype log-ratios with individual cancer survival and cancer stage estimated by Cox proportional hazards (CoxPH) modeling 6

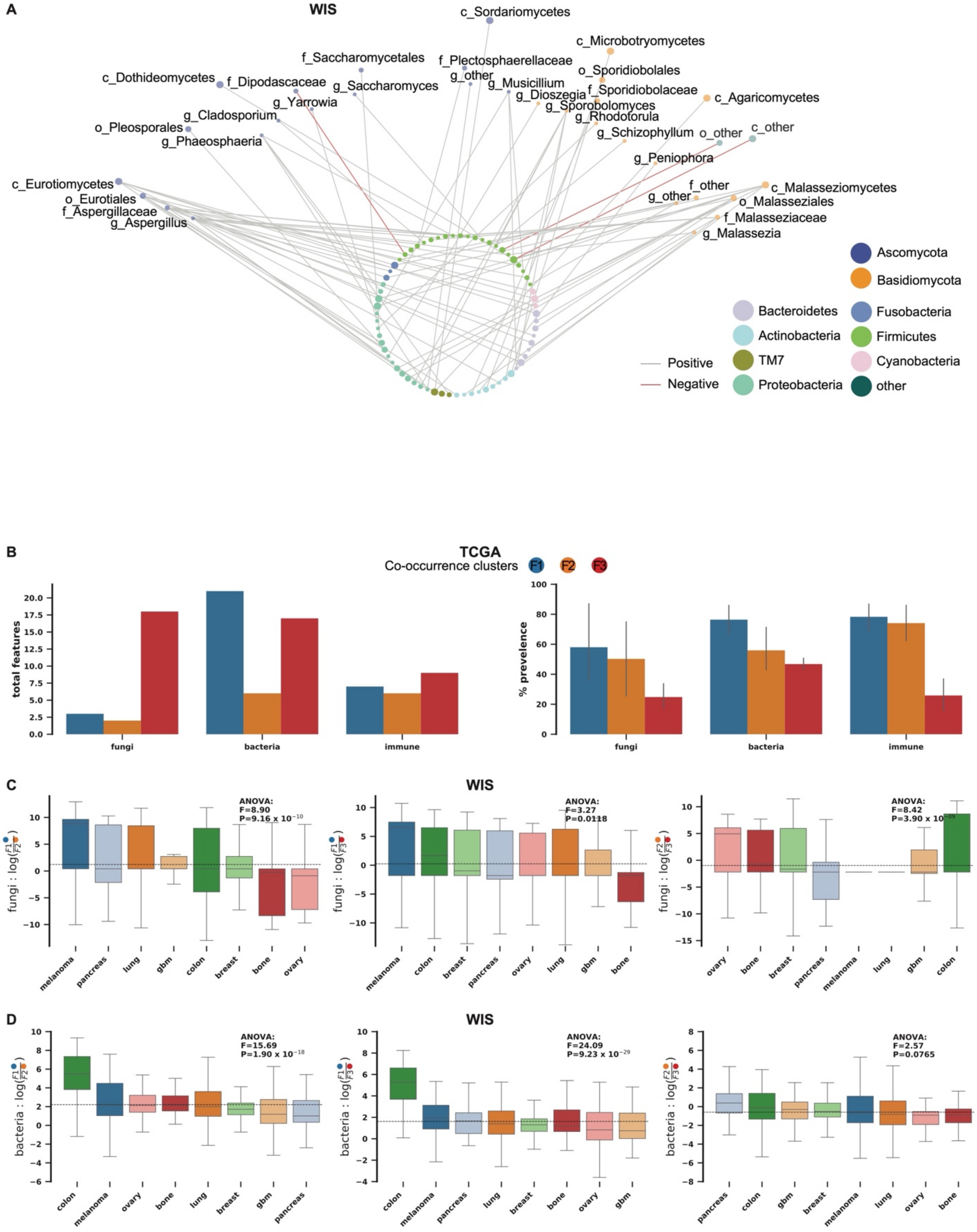
A
WIS
c_Sordariomycetes
c_Microbotryomycetes
f_Saccharomycetales
f_Plectosphaerellaceae
o_Sporidiobolales
c_Dothideomycetes
f_Dipodascaceae
g_Saccharomyces
g_other
g_Musicillium
f_Sporidiobolaceae
g_Yarrowia
g_Dioszegia
g_Sporobolomyces
c_Agaricomycetes
g_Cladosporium
g_Rhodotorula
o_Pleosporales
g_Phaeosphaeria
g_Schizophyllum
o_other
c_other
g_Peniophora
c_Eurotiomycetes
o_Eurotiales
f_Aspergillaceae
g_Aspergillus
f_other
g_other
c_Malasseziomycetes
o_Malasseziales
f_Malasseziaceae
g_Malassezia
Ascomycota
Basidiomycota
Bacteroidetes
Fusobacteria
Actinobacteria
Firmicutes
TM7
Cyanobacteria
Proteobacteria
other
Positive
Negative
B
TCGA
Co-occurrence clusters F1 F2 F3
total features
% prevelence
fungi
bacteria
immune
C
WIS
ANOVA:
F=8.90
P=9.16 x 10^-10
ANOVA:
F=3.27
P=0.0118
ANOVA:
F=8.42
P=3.90 x 10^-09
fungi : log(F1/F2)
fungi : log(F1/F3)
fungi : log(F2/F3)
melanoma
pancreas
lung
gbm
colon
breast
bone
ovary
D
WIS
ANOVA:
F=15.69
P=1.90 x 10^-18
ANOVA:
F=24.09
P=9.23 x 10^-29
ANOVA:
F=2.57
P=0.0765
bacteria : log(F1/F2)
bacteria : log(F1/F3)
bacteria : log(F2/F3)

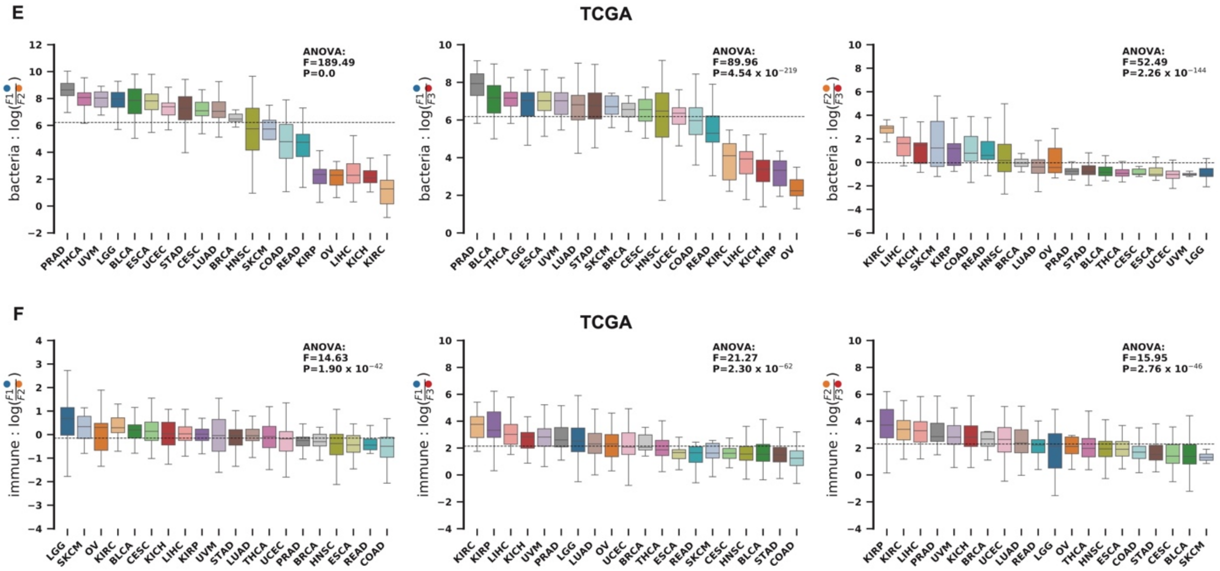

Data S4.1. Analyses of fungi-bacteria co-occurrences

(**A**) Network of fungi-bacteria connections at different taxonomic levels found in breast tumors drawn with Cytoscape (3.8.1). Nodes represent taxa color-coded according to phyla. Bacterial nodes are organized by phyla in a circle at the bottom. Fungal nodes are labeled with taxonomy preceded by a letter representing the taxonomic level (g: genus, f: family, o: order, c: class) and are organized in rainbow fashion according to taxonomic level from class (outer layer) to genus (inner layer). Nodes are grouped on a gradient from outer to inner layer based on taxonomic hierarchy. Size of the node corresponds to its taxonomic level from class (large) to genus (small). Edges represent connections between nodes with significant Normalized Mutual Information scores (permutation test n=1000, BH-FDR≤0.2) (STAR Methods). Both positive (gray) and negative (red) interactions are shown. In breast tumors, interdomain interactions are mainly positive with Malassezia and Aspergillus representing two interaction hubs.

(**B**) Enumeration of features and their prevalences in the MMvec-derived mycotypes clusters. Left: total number of fungal and bacterial genera and immune cell types within F1 (blue), F2 (orange), and F3 (red). Right: Prevalences of fungal and bacterial genera and immune cell types within F1 (blue), F2 (orange), and F3 (red). Error bars denote standard deviation of genera prevalences included in the mycotypes. These same colors correspond to the mycotypes clusters in Figures 4A.

(**C**) Log-ratios of fungi in the WIS cohort across WIS cancer types using the F1, F2, or F3 mycotype-associated fungi from the TCGA analysis.

(**D**) Log-ratios of bacteria in the WIS cohort across WIS cancer types using the F1, F2, or F3 mycotype-associated bacteria from the TCGA analysis. Two out of the three bacterial log-ratios (F1/F2 and F2/F3) show significant variation across cancer types in both cohorts.

(**E-F**) Log-ratios of bacterial (**E**) and immune (**F**) abundances associated with each mycotype abundances across TCGA cancer types, revealing significantly differing values (one-way ANOVA statistics inset on plots).

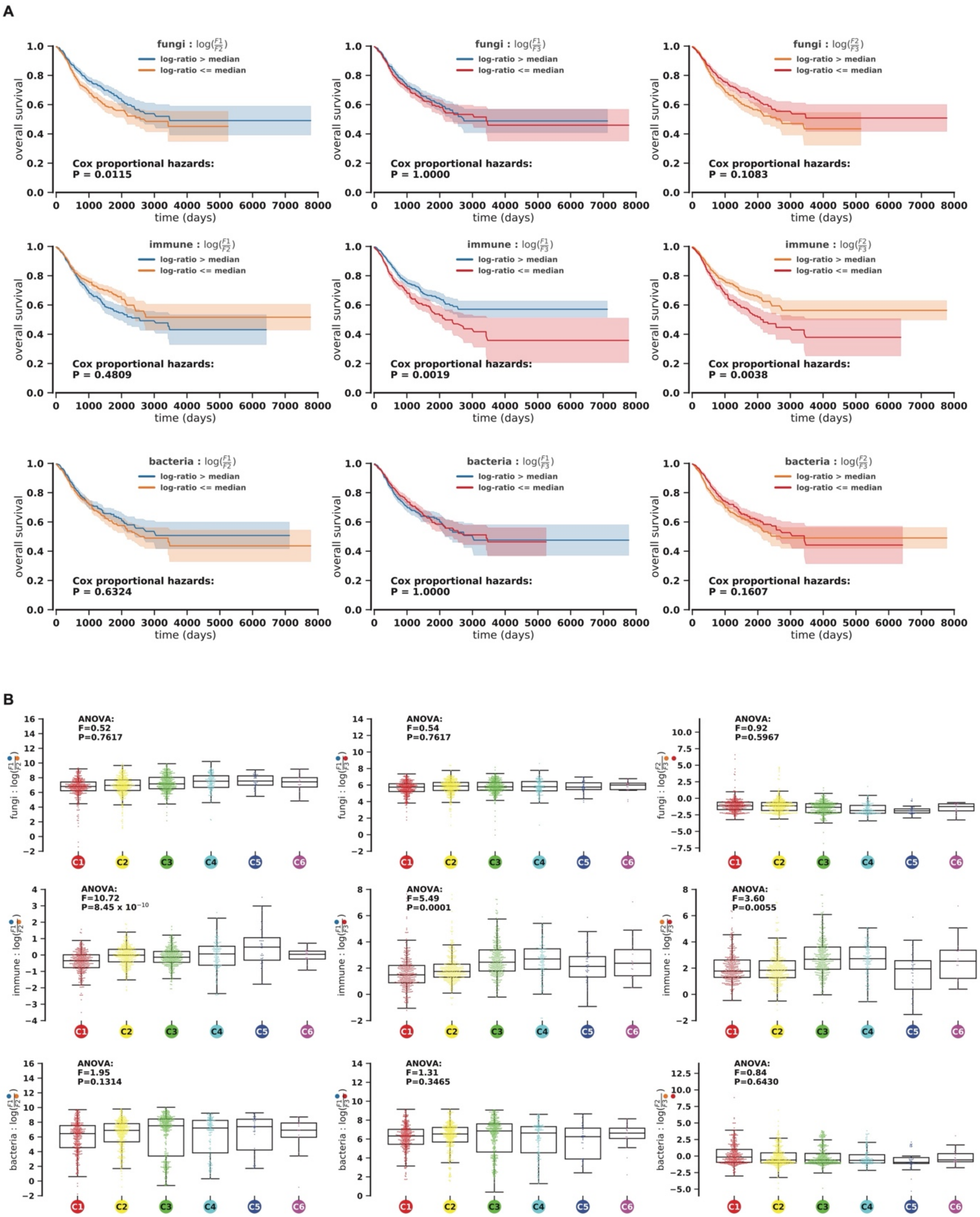

Data S4.2. Associations of multi-domain log-ratios with survival and immune response subtypes

(**A**) Log-ratios of fungi, immune cell types, or bacteria associated with the three mycotypes identified in TCGA (STAR Methods) and their relationship to pan-cancer survival (n=20 cancer types). Three out of the nine comparisons separated survival in a statistically significant way:

fungal F1/F2; immune F1/F3; immune F2/F3. The specific fungi, bacteria, and immune cell types included in these mycotypes are shown in Figure 4A. Table S7.6 shows the sample sizes above and below the medians.

(**B**) Log-ratios of fungi, immune cell types, or bacteria associated with the three mycotypes identified in TCGA (STAR Methods) and their relationship to immune response subtypes. C1: wound healing; C2: IFN-γ dominant; C3: inflammatory; C4: lymphocyte depleted (but with second highest macrophages); C5: immunologically quiet (but with highest macrophages); C6: TGF-β dominant. The log-ratios that were associated with significantly varied immune responses were immune cell compositions associated with the mycotypes (middle row).

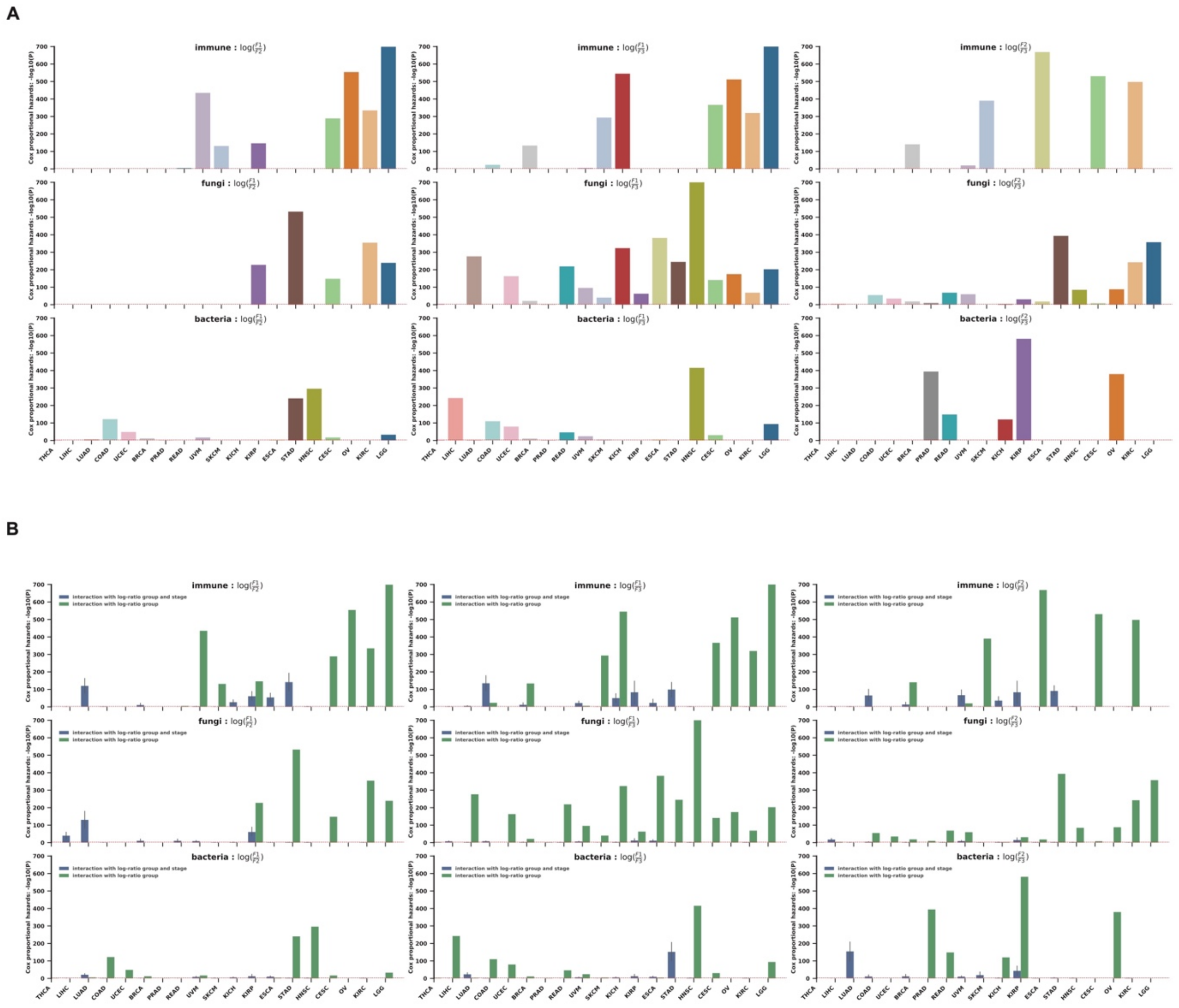

Data S4.3. Associations of mycotype log-ratios with individual cancer survival and cancer stage estimated by Cox proportional hazards (CoxPH) modeling

(**A**) CoxPH modeling survival based on log-ratio median cutoffs using an interaction term for cancer type. The y-axis denotes the significance (-$\log_{10}$ p-value) of the interaction for each cancer type. In the original survival analysis, the mycotypes log-ratios that distinguished pan-cancer survival included immune F1/F3 (top middle), immune F2/F3 (top right), and fungal F1/F2 (middle left).

(**B**) CoxPH modeling survival based on log-ratio median cutoffs using interaction terms for cancer type (green) and cancer stage (blue). The y-axis denotes the significance (-$\log_{10}$ p-value) of the interaction for each cancer type and stage. In the original survival analysis, the mycotypes log-ratios that distinguished pan-cancer survival included immune F1/F3 (top middle), immune F2/F3 (top right), and fungal F1/F2 (middle left).

(**A-B**) Determination of CoxPH significance was directional, as shown in Figure 4D, such that significance implied larger log-ratio values above the median were associated with better survival.
