# Supplementary material for: Pan-cancer analyses reveal cancer-type-specific fungal ecologies and bacteriome interactions
Source: Cell. 2022 Sep 29;185(20):3789–3806.e17. doi: 10.1016/j.cell.2022.09.005 (PMC9567272; doi:10.1016/j.cell.2022.09.005)
Supplement: Data S5. Mycobiome machine learning and control analyses, related to Figure 5 and STAR Methods [file mmc13.pdf]

Pan-cancer analyses reveal cancer type-specific fungal ecologies and bacteriome interactions

DATA S5

Mycobiome machine learning and control analyses, related to **Figure 5** and **STAR Methods**.

Table of Contents

DATA S5 NOTE 2

TCGA ML: Positive and negative control analyses 2

DATA S5 FIGURES 5

Data S5.1. Machine learning on TCGA subsets of fungal data to distinguish one cancer type versus all others 7

Data S5.2. Machine learning on TCGA subsets of raw fungal count data summarized to various taxa levels to distinguish one cancer type versus all others 11

Data S5.3. Evaluating negative and positive controls for machine learning on TCGA raw data . 14

Data S5.4. Representative differential abundance volcano plots of one cancer type versus all others using intratumoral decontaminated fungi in TCGA 17

Data S5.5. Evaluating WIS-associated features in TCGA and in the WIS-cohort for machine learning 18

Data S5.6. TCGA batch correction and negative and positive controls on pan-cancer tumor sample machine learning 20

Data S5.7. Differential abundance volcano plots of stage I versus stage IV tumors using intratumoral decontaminated fungi in TCGA 22

Data S5.8. TCGA machine learning between stage I and stage IV tumors, as well as tumor versus NAT samples 26

Data S5.9. Representative differential abundance volcano plots of one cancer type versus all others using blood-derived decontaminated fungi in TCGA 28

Data S5.10. Machine learning on TCGA subsets of raw fungal count data to distinguish blood samples from one cancer type versus all others 31

Data S5.12. Evaluating negative and positive controls for machine learning on TCGA blood raw data 34

Data S5.13. TCGA batch correction and negative and positive controls on pan-cancer blood sample machine learning 36

Data S5.14. Testing other machine learning model types for cancer type discrimination in TCGA using batch-corrected and raw decontaminated data 39

Data S5.15. Testing other sampling strategies during machine learning for cancer type discrimination in TCGA using batch-corrected and raw decontaminated data 41

DATA S5 NOTE

TCGA ML: Positive and negative control analyses

Our previous machine learning (ML) analyses of the TCGA bacteriome used batch-corrected data to demonstrate cancer type-specific microbiomes (Poore et al., 2020). However, to build greater confidence in these models when using fungal information, and to demonstrate that the batch correction was not biasing the ML performance, we applied our methods to raw count data with extensive control analyses and orthogonal (differential abundance) methods. These are detailed below for pan-cancer discrimination using primary tumor tissues and blood samples.

To use raw count data for primary tumor discrimination, we subset all TCGA samples to (i) a single sequencing platform (Illumina HiSeq) that accounted for 97% (15,088/15,512) of samples; (ii) individual sequencing centers (n=7); and, as necessary, (iii) single experimental strategies (WGS vs. RNA-Seq), although most centers (6 of 7) only performed one. Gradient boosting machine learning models with 10-fold cross-validation were then built within each primary tumor sample subset to iteratively predict one-cancer-type-versus-all-others using decontaminated raw mycobiome counts (STAR Methods) (Figure 5A, Data S5.1A-F). Areas under the receiver operating characteristic (AUROC) and precision-recall (AUPR) curves were calculated on each independent holdout k^{th}-fold and aggregated to estimate the average performance and 95% confidence intervals thereof per cancer type per center, which were then compared to their null values—50% area for AUROC and the dynamic prevalence of the positive class for AUPR. Across all primary tumor comparisons, the average fungal AUROC was 81.04% (95% CI: [78.37, 83.72]%), with models on WGS data significantly outperforming RNA-Seq-derived models (Data S5.1G), likely from ~100-fold more decontaminated fungal reads in WGS samples (Data S5.1H). Average AUROC and AUPR also significantly correlated with the ratio of the minority class to the majority class (Data S5.1I-J), suggesting sample count performance limitations.

We then performed numerous positive and negative control analyses. First, we re-evaluated models using summarized taxonomic counts, based on aggregating the decontaminated species data, revealing generalizable performance that frequently plateaued at the order level (Data S5.2A-G). We also re-evaluated models subsetted to 34 WIS-overlapping species or 31 species with ≥1% aggregate genome coverage (Figure 5A, Data S5.1A-F, Table S4.1), demonstrating similar performance to the full set of 224 decontaminated species. As negative controls, we evaluated models with scrambled metadata labels or shuffled count data (STAR Methods), verifying that decontaminated, WIS-intersecting, or high coverage fungi always outperformed negative controls (Figure 5B, Data S5.3A-F). We also split raw count data into two pan-cancer stratified halves (STAR Methods), trained one-cancer-type-versus-all-other models on each half, and then cross-tested the models on the other data halves, demonstrating highly similar performance (Data S5.3G-H). We then performed differential abundance testing using ANCOM-BC (Lin and Peddada, 2020) on every sequencing center subset, again showing cancer type-specific differences (Data S5.4). We then built a website for users to interactively access the resultant volcano plots (https://cancermycobiome.shinyapps.io/website/, user="reviewer", password="fungi"). Overall, these positive and negative control analyses evince cancer type-specific primary tumor mycobiomes.

Motivated by equivalent ML performance with 34 WIS-intersecting fungal species (15% of the decontaminated features), we simulated 500 independent folds (50 iterations of ten-fold cross-validation) of multi-class cancer type classification among all WGS primary tumors (n=24 cancer types) between WIS-overlapping species and equivalent numbers of randomly sampled non-WIS-intersecting, decontaminated species (STAR Methods). The 34 WIS-overlapping species provided better pan-cancer performance (Data S5.5A), demonstrating their utility and generalizability between two international cohorts. We repeated these simulations using WIS-overlapping fungi, bacteria, or both and found stepwise, significant, synergistic species-level ML performance enhancements in TCGA tumors with both domains (Figures 5C). We then applied our one-cancer-type-versus-all-others machine learning framework to the WIS cohort with matched fungal and bacterial count data (Data S5.5B), demonstrating cancer type-specific discrimination, albeit with lower performance than TCGA models (Average fungal AUROC: 72.09%, 95% CI: [63.52, 80.66]%). Like TCGA, the ratio of the minority to majority class significantly correlated with the average AUPR, but average AUROC did not (Data S5.5C-D), suggesting that some ML performance was sample size limited. Using relative abundances or binary presence-absences instead of counts did not improve WIS ML performance (Data S5.5E). Importantly, though, the addition of fungal information to bacterial data synergistically and significantly increased the average AUROC (Figure 5D) and raised AUPR, albeit not significantly (Figure 5D). Thus, despite numerous sample, methodological, and bioinformatic differences between the TCGA and WIS cohorts, they both provide concordant cancer type-specific mycobiome conclusions on raw data and shared features, and depict significant performance benefits when combining fungal and bacterial information.

Our previous analysis of the TCGA bacteriome also indicated the presence of cancer type-specific microbial DNA in patient blood (Poore et al., 2020), motivating a similar analysis for fungal DNA. To use decontaminated raw count data, we again subset all TCGA blood samples to (i) those run on Illumina HiSeqs and (ii) individual sequencing centers (n=5), resulting in 1771 samples across 24 cancer types. We note that TCGA blood samples only underwent WGS, so no RNA-Seq samples were available, and that TCGA protocols permitted either whole blood or buffy coat collection collectively under a single "blood derived normal" sample type (i.e., for patient germline sequencing). We then repeated the one-cancer-type-versus-all-others ML approach using ten-fold cross validation and differential abundance using ANCOM-BC (Lin and Peddada, 2020) on the raw, decontaminated, blood-derived, fungal count data within each sequencing center, which revealed cancer type-specific blood mycobiomes (AUROC 95% CI on all cancer-center combinations and folds: [84.80, 87.58]%; Figure 5F, Data S5.9-S5.10). Aggregating counts to higher taxonomic levels using the decontaminated species-level data still revealed strong discriminatory performance that frequently plateaued at the order level (Data S5.11), suggesting taxonomic generalizability. We then performed the same negative control analyses as before, scrambling metadata disease type labels and shuffling the count data, revealing null, significantly worse performance (Data S5.12A-E). Validation by independently training models on stratified halves of raw data, followed by cross-testing, also demonstrated significantly correlated performance (Data S5.3G-H). As with primary tumor data, we noted better performances using the 34 WIS-overlapping species than any other equivalent number of randomly drawn decontaminated species across 500 independent folds (50 iterations of ten-fold cross-validation) of multi-class cancer type classification in TCGA blood samples (n=24 cancer types; Data S5.12F-

G), and synergistic performance enhancements when combining WIS-overlapping fungi and bacteria (Figure 5G).

DATA S5 FIGURES

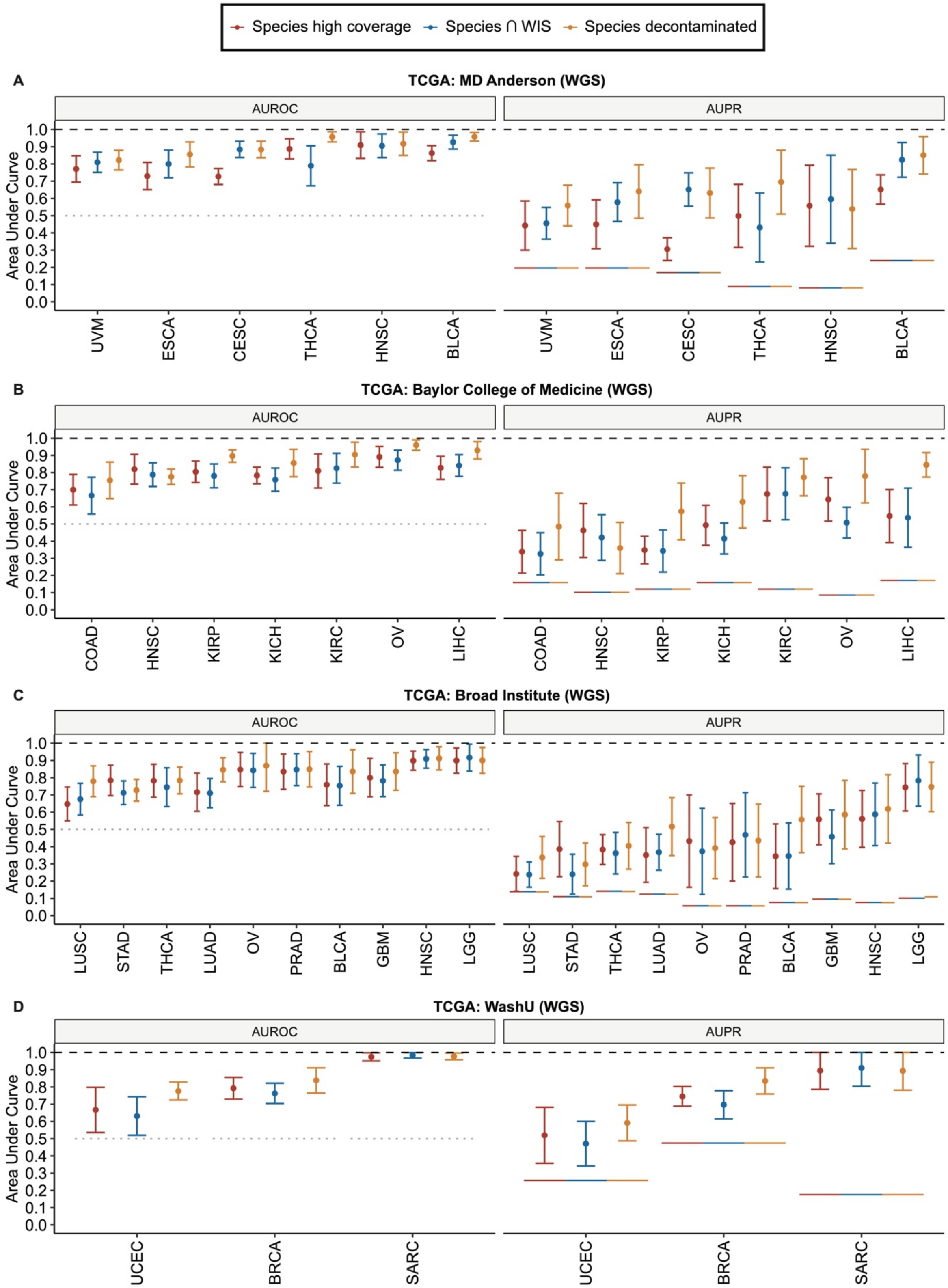

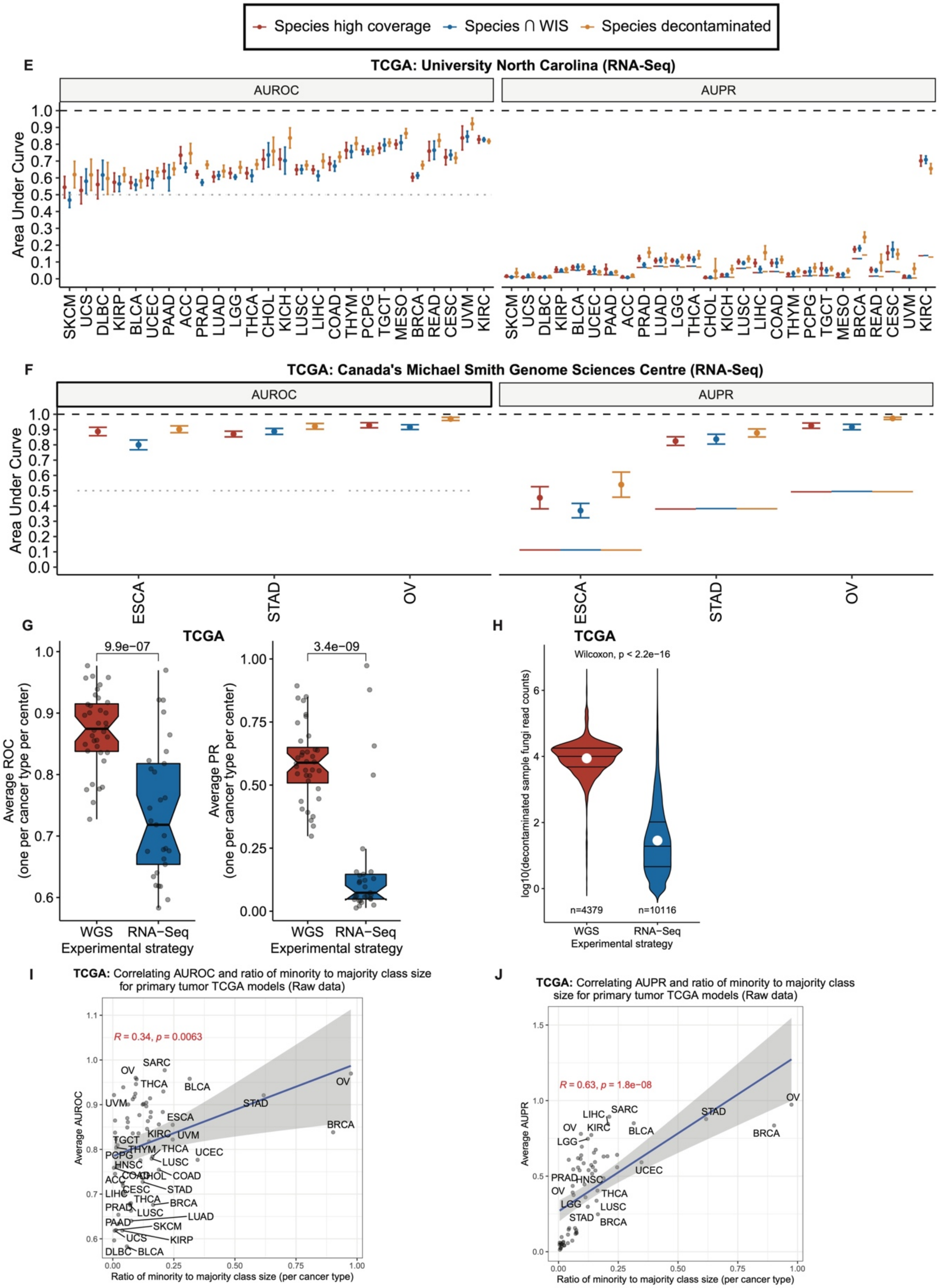
Species high coverage
Species ∩ WIS
Species decontaminated
E
TCGA: University North Carolina (RNA-Seq)
AUROC
AUPR
Area Under Curve
SKCM UCS DLBC KIRP BLCA UCEC PAAD ACC PRAD LUAD LGG THCA CHOL KICH LUSC LIHC COAD THYM PCPG TGCT MESO BRCA READ CESC UVM KIRC
SKCM UCS DLBC KIRP BLCA UCEC PAAD ACC PRAD LUAD LGG THCA CHOL KICH LUSC LIHC COAD THYM PCPG TGCT MESO BRCA READ CESC UVM KIRC
F
TCGA: Canada's Michael Smith Genome Sciences Centre (RNA-Seq)
AUROC
AUPR
Area Under Curve
ESCA
STAD
OV
ESCA
STAD
OV
G
TCGA
9.9e−07
3.4e−09
Average ROC (one per cancer type per center)
Average PR (one per cancer type per center)
WGS
RNA−Seq
Experimental strategy
WGS
RNA−Seq
Experimental strategy
H
TCGA
Wilcoxon, p < 2.2e−16
log10(decontaminated sample fungi read counts)
n=4379
n=10116
WGS
RNA−Seq
Experimental strategy
I
TCGA: Correlating AUROC and ratio of minority to majority class size for primary tumor TCGA models (Raw data)
R = 0.34, p = 0.0063
Average AUROC
Ratio of minority to majority class size (per cancer type)
J
TCGA: Correlating AUPR and ratio of minority to majority class size for primary tumor TCGA models (Raw data)
R = 0.63, p = 1.8e−08
Average AUPR
Ratio of minority to majority class size (per cancer type)

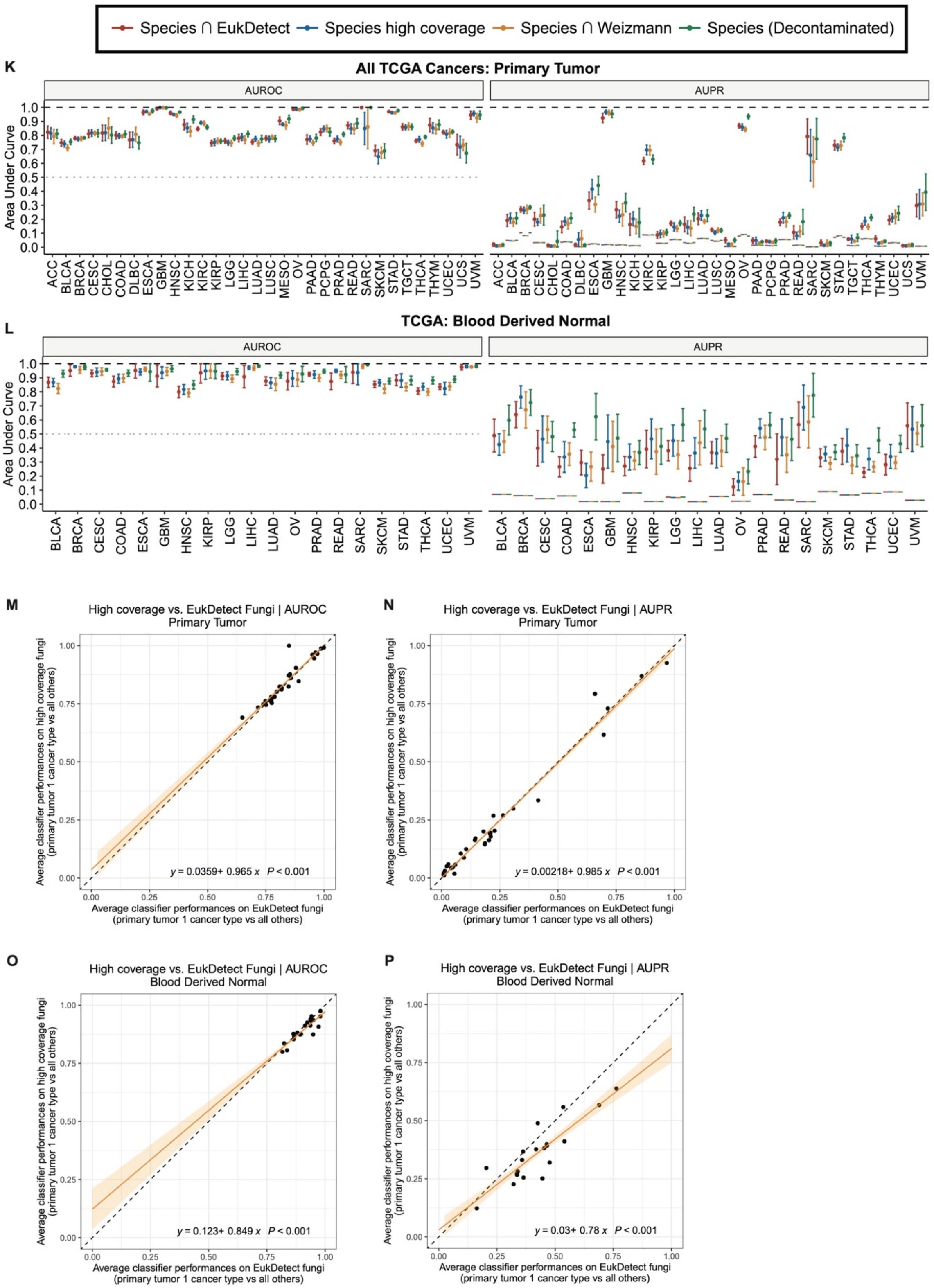

Data S5.1. Machine learning on TCGA subsets of fungal data to distinguish one cancer type versus all others

(**A-G**) Raw count data were subset to a single sequencing center, experimental strategy (WGS or RNA-Seq), and sequencing platform (Illumina HiSeq) prior to evaluating ten-fold cross-validation gradient boosting machine learning models. Notably, 6 of 7 sequencing centers only used one type of experimental strategy (WGS or RNA-Seq); the exception was the Broad Institute, which performed RNA-Seq on glioblastoma tumors, but since it was the only cancer type to have RNA from Broad, it could not be compared in one-cancer-type-versus-all-other predictions here. Predictions were made on each of the ten holdout folds to generate average and 95% confidence intervals of discriminatory performance, as measured by AUROC and AUPR. Multiple feature sets were run, including 31 fungal species with aggregate coverage ≥1% ("high coverage") (red), 34 fungal species that overlapped with the WIS cohort (blue), and 224 fungal species that passed TCGA decontamination (orange). A minimum of 20 samples were required in any comparison to be tested. Cancer type discrimination is shown among (**A**) MD Anderson (WGS) samples, (**B**) Baylor College of Medicine (WGS) samples, (**C**) Broad Institute (WGS) samples, (**D**) Washington University (WashU; WGS) samples, (**E**) University of North Carolina (RNA-Seq) samples, and (**F**) Canada's Michael Smith Genome Sciences Centre (RNA-Seq) samples. (**G**) The average AUROC (left) and AUPR (right), using the mean performance value from each cancer type within each sequencing center, was significantly higher for WGS-based models compared to RNA-Seq-based models. Two-sided Wilcoxon test shown.
(**H**) Distributions of reads/sample in TCGA between WGS and RNA-Seq samples reveals approximately 100-fold more reads/sample in WGS samples than RNA-Seq samples. Y-axis plotted on log scale. Two-sided Wilcoxon test shown.
(**I**) Scatter plot and regression analysis comparing average AUROC values (one per cancer type per sequencing center) to the ratio of the minority class (primary tumors of a cancer type of interest) to the majority class (the remaining primary tumors of other cancer types). Note: Since some cancer types were shared across sequencing centers, they may appear more than once in this plot.
(**J**) Scatter plot and regression analysis comparing average AUPR values (one per cancer type per sequencing center) to the ratio of the minority class to the majority class. Note: Since some cancer types were shared across sequencing centers, they may appear more than once in this plot.
(**K-L**) Batch-corrected TCGA data were subset to fungal species detected by EukDetect prior to running one-cancer-type-versus-all-others machine learning among (**K**) primary tumors or (**L**) blood samples. Performances were then plotted adjacent to those from other fungal species subsets, as originally shown in Figure 5E and Figure 5H, for comparison. Predictions were made on each of the ten holdout folds to generate average and 95% confidence intervals of discriminatory performance, as measured by AUROC and AUPR.
(**M-N**) Machine learning performance for one-cancer-type-versus-all-others among TCGA primary tumors using EukDetect fungi versus high coverage fungi were regressed using (**M**) AUROC or (**N**) AUPR values. Linear regression fit and associated p-value are inset on the plots. Performances derived from machine learning models on batch-corrected data.
(**O-P**) Machine learning performance for one-cancer-type-versus-all-others among TCGA blood samples using EukDetect fungi versus high coverage fungi were regressed using (**O**) AUROC or (**P**) AUPR values. Linear regression fit and associated p-value are inset on the plots. Performances derived from machine learning models on batch-corrected data.
(**A-F**, **K-L**) Dots in each plot denote average values and error bars denote 95% confidence intervals. Gray horizontal dotted lines under AUROC denote null values. Color horizontal lines under AUPR denote null values, which equates the prevalence of the positive class (each cancer

type) among the full set of all cancer types within a sequencing center subset. Null values for AUPR may vary slightly since subsetting features to only those with aggregate high coverage or overlapping with the WIS cohort sometimes left samples with zero remaining fungal reads, which had to be removed, thereby slightly modifying the prevalence of the positive class.

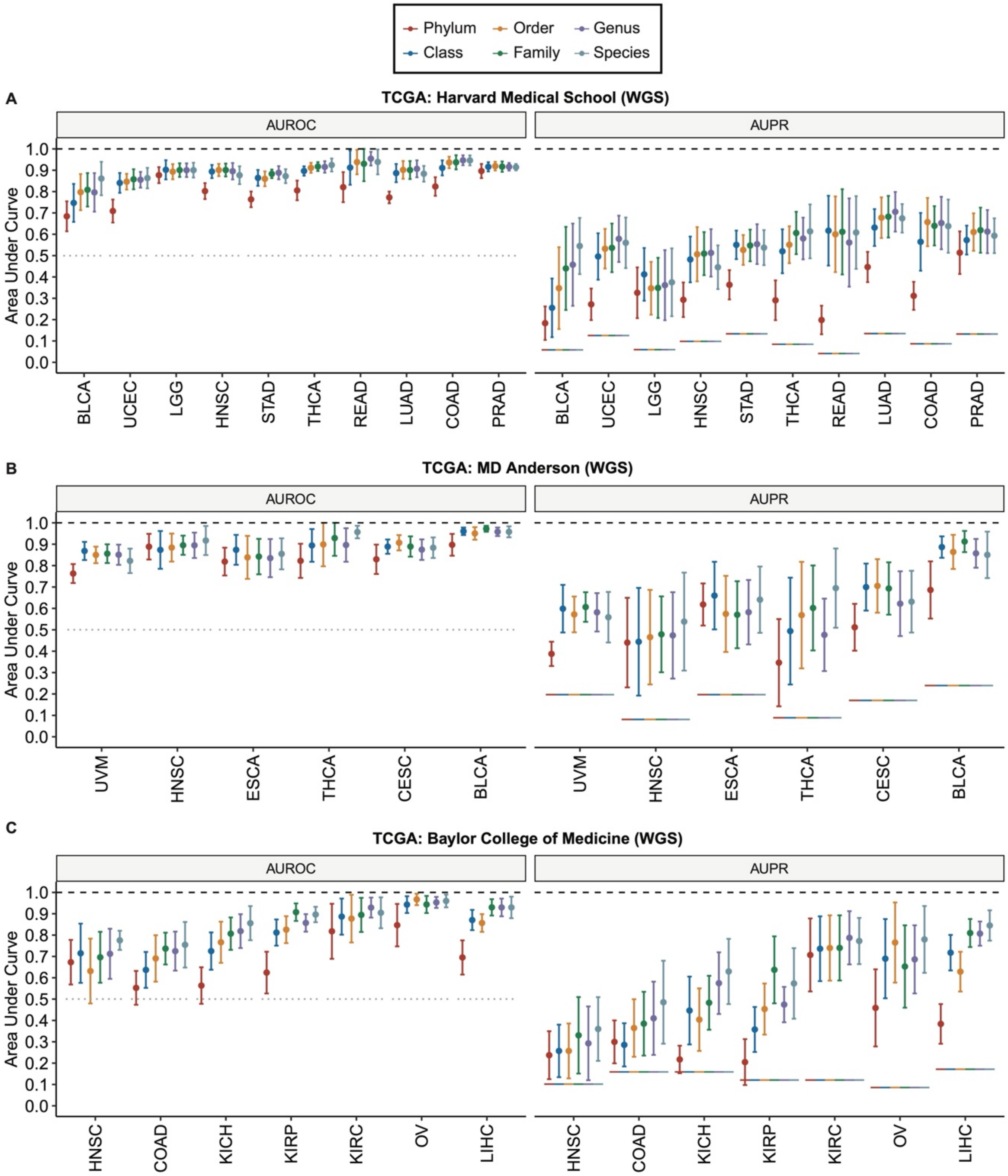

Phylum
Class
Order
Family
Genus
Species
A
TCGA: Harvard Medical School (WGS)
AUROC
AUPR
Area Under Curve
BLCA UCEC LGG HNSC STAD THCA READ LUAD COAD PRAD
B
TCGA: MD Anderson (WGS)
UVM HNSC ESCA THCA CESC BLCA
C
TCGA: Baylor College of Medicine (WGS)
HNSC COAD KICH KIRP KIRC OV LIHC

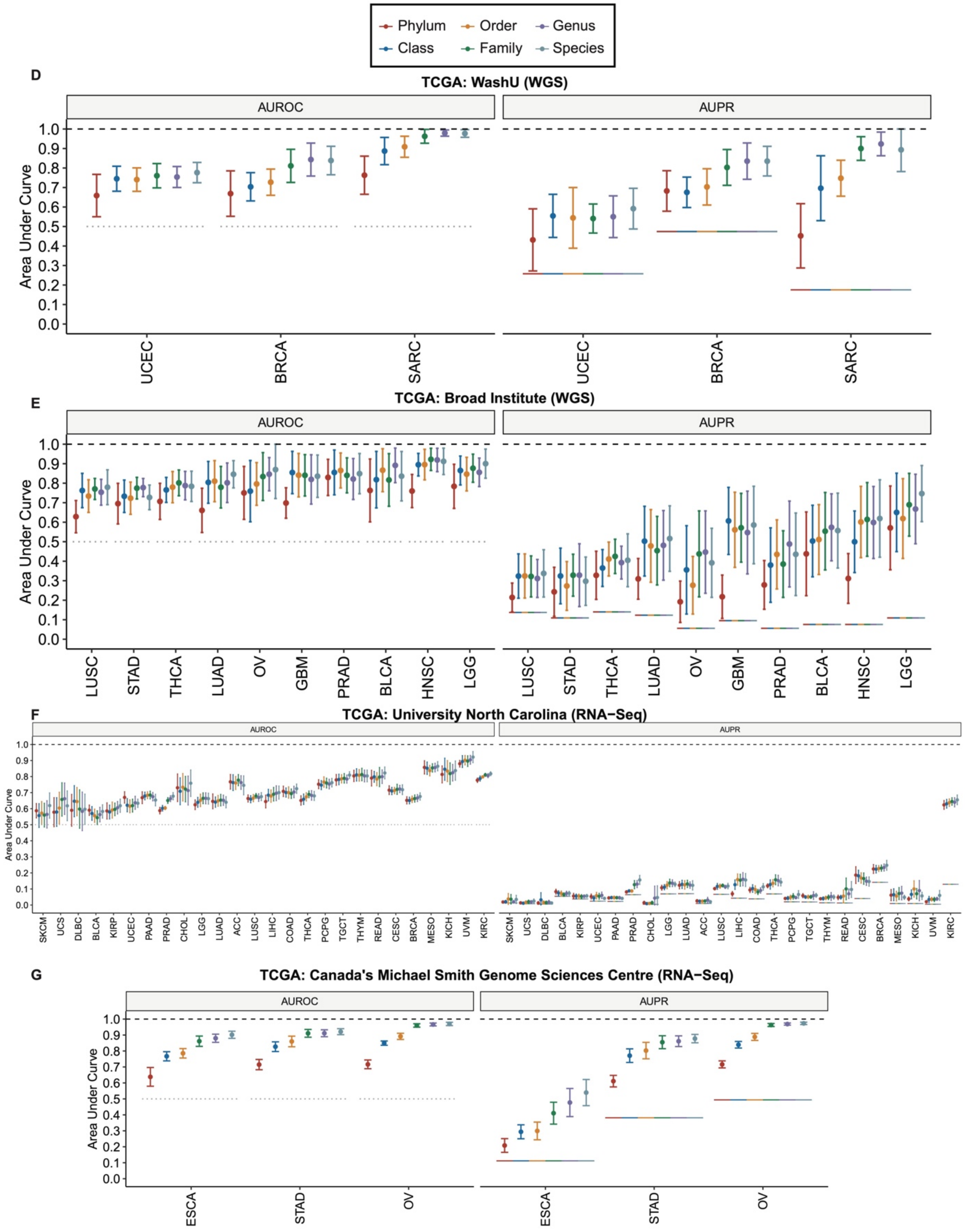

Data S5.2. Machine learning on TCGA subsets of raw fungal count data summarized to various taxa levels to distinguish one cancer type versus all others

(**A-G**) Decontaminated fungal count data were subset to a single sequencing center, experimental strategy (WGS or RNA-Seq), and sequencing platform (Illumina HiSeq) prior to summarizing to various taxa levels. Predictions were made on each of the ten holdout folds to generate average and 95% confidence intervals of discriminatory performance, as measured by AUROC and AUPR. A minimum of 20 samples were required in any comparison to be tested. Cancer type discrimination is shown among (**A**) Harvard Medical School (WGS) samples, (**B**) MD Anderson (WGS) samples, (**C**) Baylor College of Medicine (WGS) samples (**D**) Washington University (WashU; WGS) samples, (**E**) Broad Institute (WGS) samples, (**F**) University of North Carolina (RNA-Seq) samples, and (**G**) Canada's Michael Smith Genome Sciences Centre (RNA-Seq) samples. Dots in each plot denote average values and error bars denote 95% confidence intervals. Gray horizontal dotted lines under AUROC denote null values. Color horizontal lines under AUPR denote null values, which equates the prevalence of the positive class (each cancer type) among the full set of all cancer types within a sequencing center subset.

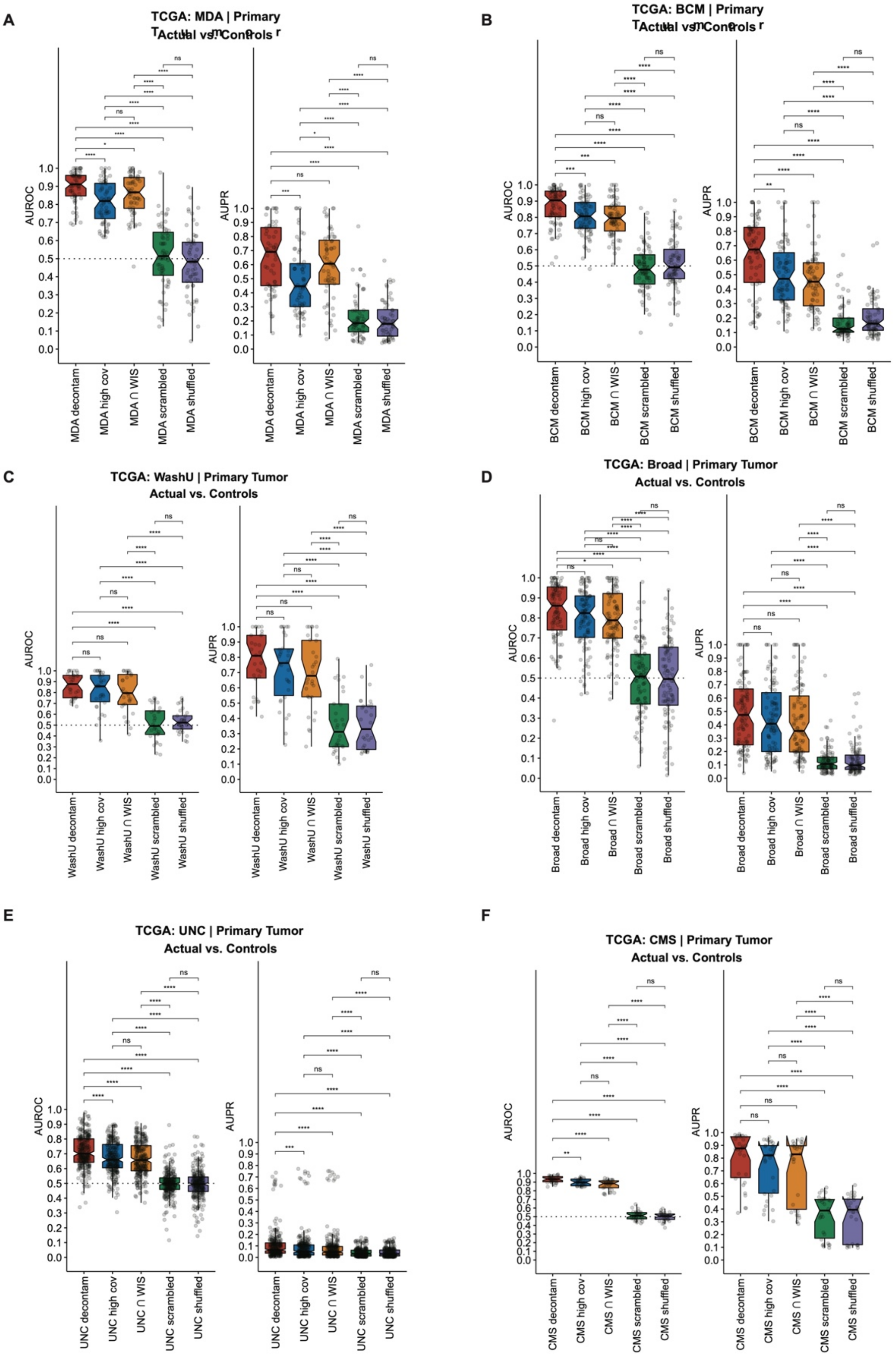

A
TCGA: MDA | Primary
Actual vs. Controls
AUROC
AUPR
MDA decontam
MDA high cov
MDA ∩ WIS
MDA scrambled
MDA shuffled
B
TCGA: BCM | Primary
Actual vs. Controls
AUROC
AUPR
BCM decontam
BCM high cov
BCM ∩ WIS
BCM scrambled
BCM shuffled
C
TCGA: WashU | Primary Tumor
Actual vs. Controls
AUROC
AUPR
WashU decontam
WashU high cov
WashU ∩ WIS
WashU scrambled
WashU shuffled
D
TCGA: Broad | Primary Tumor
Actual vs. Controls
AUROC
AUPR
Broad decontam
Broad high cov
Broad ∩ WIS
Broad scrambled
Broad shuffled
E
TCGA: UNC | Primary Tumor
Actual vs. Controls
AUROC
AUPR
UNC decontam
UNC high cov
UNC ∩ WIS
UNC scrambled
UNC shuffled
F
TCGA: CMS | Primary Tumor
Actual vs. Controls
AUROC
AUPR
CMS decontam
CMS high cov
CMS ∩ WIS
CMS scrambled
CMS shuffled

G

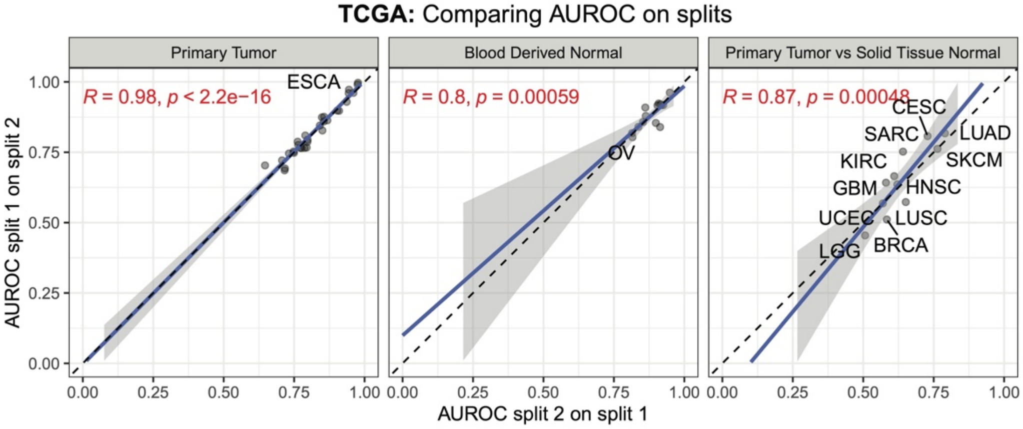

H

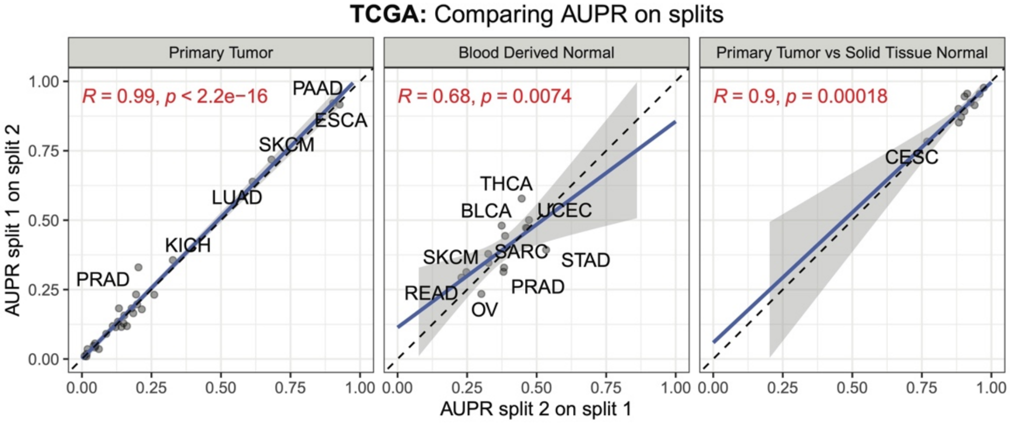

Data S5.3. Evaluating negative and positive controls for machine learning on TCGA raw data

(**A-F**) Machine learning models predicting one cancer type versus all others with intratumoral fungi were re-evaluated using scrambled metadata or shuffled count data within each cancer type and sequencing center subset and compared to performance on actual biological samples. Biological comparisons used 224 decontaminated fungal species (x-axis "decontam"), 31 fungal species with ≥1% aggregate genome coverage (x-axis "high cov"), or 34 fungal species overlapping with the WIS cohort (x-axis "∩ WIS"). Since each fold of the biological samples should show better than random performance, all folds from each cancer type comparison (i.e., ten from each cancer type) are included in the biological sample boxplots, and each fold from the scrambled or shuffled controls are also shown. Comparison of biological versus negative controls for (**A**) MD Anderson ("MDA") WGS samples, (**B**) Baylor College of Medicine ("BCM") WGS samples, (**C**) Washington University ("WashU") WGS samples, (**D**) Broad Institute ("Broad") WGS samples, (**E**) University of North Carolina ("UNC") RNA-Seq samples, and (**F**) Canada's Michael Smith ("CMS") Genome Sciences Centre (RNA-Seq) samples. Pairwise two-sided Wilcox tests, corrected for multiple hypothesis testing using the Benjamini-Hochberg method, are shown. ns: not significant (q>0.05); *: q≤0.05; **: q≤0.01; ***: q≤0.001; ****: q≤0.0001.

(**G-H**) As a positive control for machine learning, two stratified halves of TCGA raw count data were created and machine learning models were independently built on each stratified half,

followed by testing each model on the other half's data distinguishing one cancer type versus all others (STAR Methods). The performances were then regressed to test for concordance, as shown by (**G**) AUROC and (**H**) AUPR plots. Pearson correlation is shown with concomitant *R* and p-values. The dotted line denotes what would be perfect concordance between performance on the two splits.

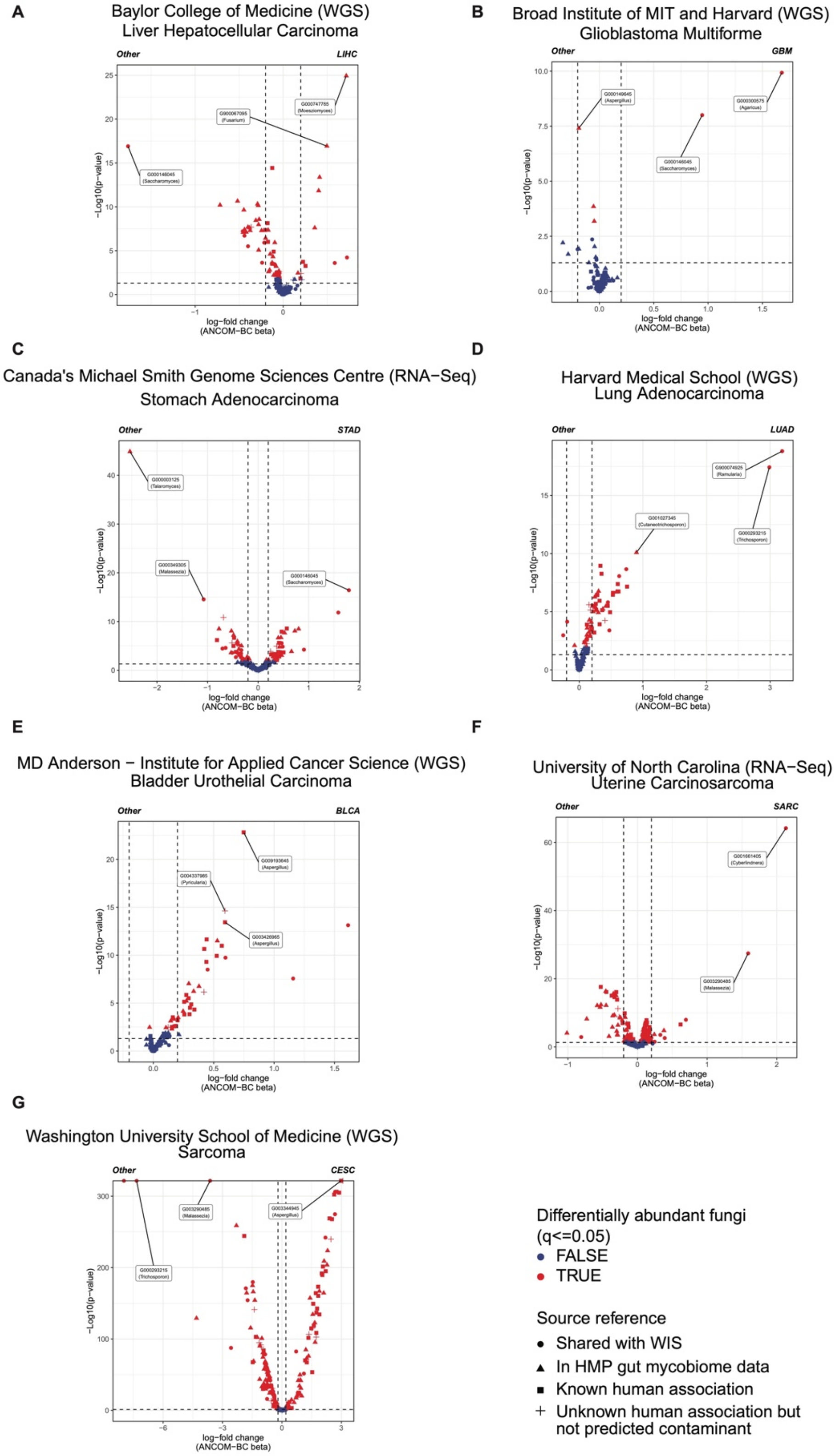

A
Baylor College of Medicine (WGS)
Liver Hepatocellular Carcinoma
Other
LIHC
G000747765 (Mrakiomyces)
G000067095 (Fusarium)
G000146045 (Saccharomyces)
B
Broad Institute of MIT and Harvard (WGS)
Glioblastoma Multiforme
Other
GBM
G000149645 (Aspergillus)
G000300575 (Agaricus)
G000146045 (Saccharomyces)
C
Canada's Michael Smith Genome Sciences Centre (RNA−Seq)
Stomach Adenocarcinoma
Other
STAD
G000003125 (Talaromyces)
G000349305 (Malassezia)
G000146045 (Saccharomyces)
D
Harvard Medical School (WGS)
Lung Adenocarcinoma
Other
LUAD
G900074025 (Ramularia)
G001027345 (Cutaneotrichosporon)
G000293215 (Trichosporon)
E
MD Anderson − Institute for Applied Cancer Science (WGS)
Bladder Urothelial Carcinoma
Other
BLCA
G009193645 (Aspergillus)
G004337985 (Pyricularia)
G003426965 (Aspergillus)
F
University of North Carolina (RNA−Seq)
Uterine Carcinosarcoma
Other
SARC
G001661405 (Cyberlindnera)
G003290485 (Malassezia)
G
Washington University School of Medicine (WGS)
Sarcoma
Other
CESC
G003290485 (Malassezia)
G003344945 (Aspergillus)
G000293215 (Trichosporon)
−Log10(p−value)
log−fold change
(ANCOM−BC beta)
Differentially abundant fungi
(q<=0.05)
FALSE
TRUE
Source reference
Shared with WIS
In HMP gut mycobiome data
Known human association
Unknown human association but
not predicted contaminant

Data S5.4. Representative differential abundance volcano plots of one cancer type versus all others using intratumoral decontaminated fungi in TCGA

(**A-G**) To calculate differential fungal abundances on TCGA primary tumor raw count data, ANCOM-BC was applied to subsets of TCGA in a single sequencing center, experimental strategy, and sequencing platform (see STAR Methods). Species-level fungal data were used. Volcano plots were then constructed using the log-fold change betas calculated by ANCOM-BC, colored red if fungi achieved a q-value≤0.05, and labeled by their reason for passing decontamination. The top 2-3 fungi in each plot by p-value smallness, if significant, were additionally labeled by its genome ID in the rep200 database and its genus name. A minimum of 10 samples per stage were required to be included in the testing. Results show differentially abundant fungi in (**A**) Baylor College of Medicine liver cancer tumors versus other cancer types, (**B**) Broad Institute glioblastoma tumors versus other cancer types, (**C**) Canada's Michael Smith Genome Science Centre stomach cancer tumors versus other cancer types, (**D**) Harvard Medical School lung cancer tumors versus other cancer types, (**E**) MD Anderson bladder cancer tumors versus other cancer types, (**F**) University of North Carolina uterine cancer tumors versus other cancer types, and (**G**) Washington University sarcoma tumors versus other cancer types.

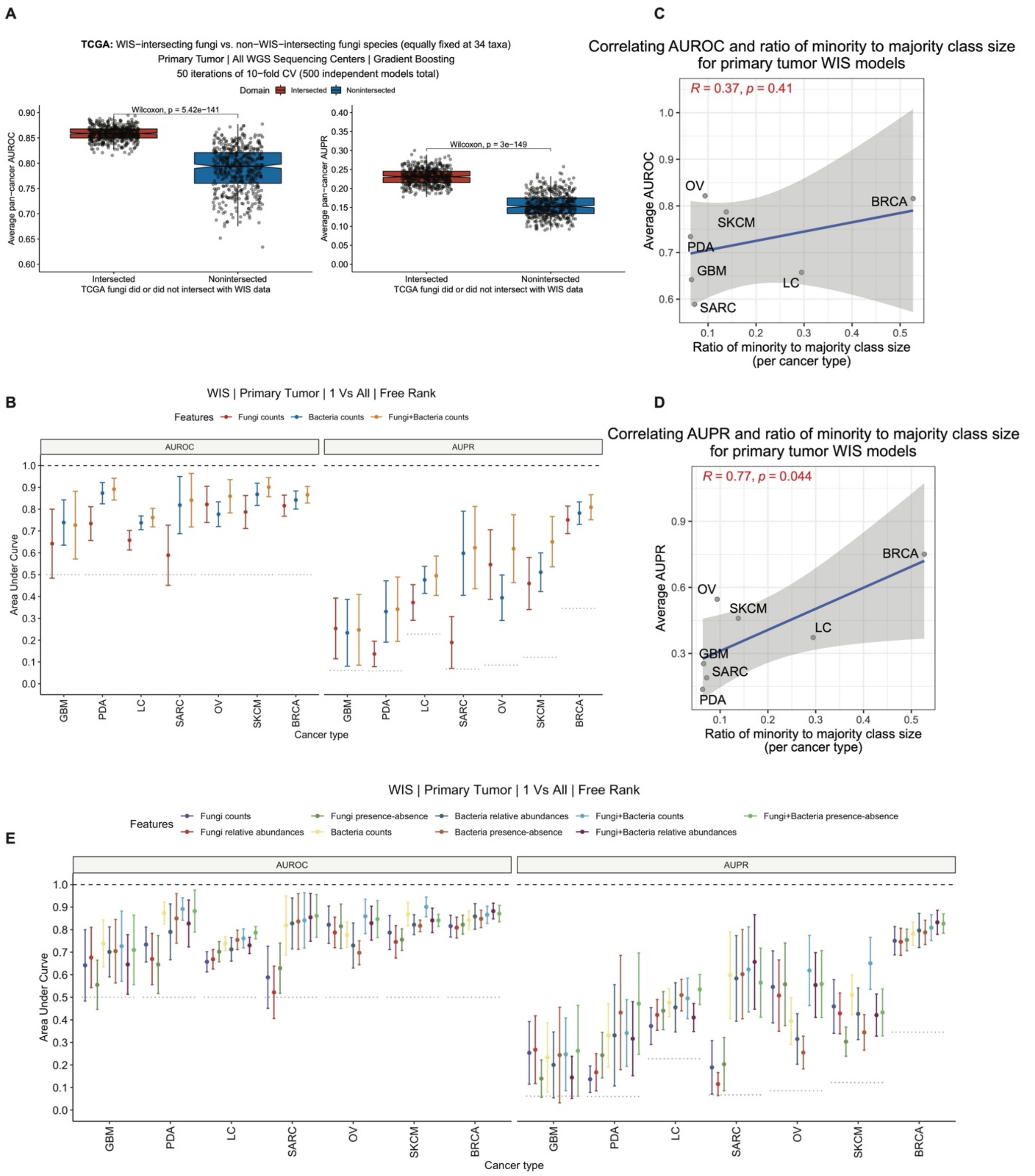

Data S5.5. Evaluating WIS-associated features in TCGA and in the WIS-cohort for machine learning

(**A**) To test whether the 34 WIS-overlapping species provided greater discriminatory performance in TCGA than other detected fungi, pan-cancer, multi-class machine learning models were built on WGS data using 10-fold cross-validation using only the 34 WIS-overlapping species or 34 non-WIS-overlapping randomly selected fungi. This process was repeated for 50 iterations (500 total

folds), and AUROC (left) and AUPR (right) performance was calculated on each holdout fold. Two-sided Wilcox tests were used to test for significant differences.

(**B**) The same ten-fold cross-validation machine learning approach was applied to the WIS cohort data, using fungal, bacterial, or fungal and bacterial raw counts to discriminate one cancer type versus all others. All filtered fungal hits across all taxa levels ("free rank") were included. Dots denote average performance and error bars denote 95% confidence intervals. Gray horizontal dots denote the null AUROC and AUPR values, the latter of which is the prevalence of the positive class (here, each cancer type).

(**C-D**) Scatter plot and regression analysis of (**C**) AUROC and (**D**) AUPR against the ratio of the minority class to the majority class. Pearson correlation coefficient (*R*) and concomitant p-value shown.

(**E**) Multiple types of feature sets were applied to WIS cohort machine learning, including raw counts, relative abundances, and binary presence-absence data across fungi, bacteria, or fungi and bacteria. All filtered fungal and/or bacterial hits across all taxa levels ("free rank") were included.

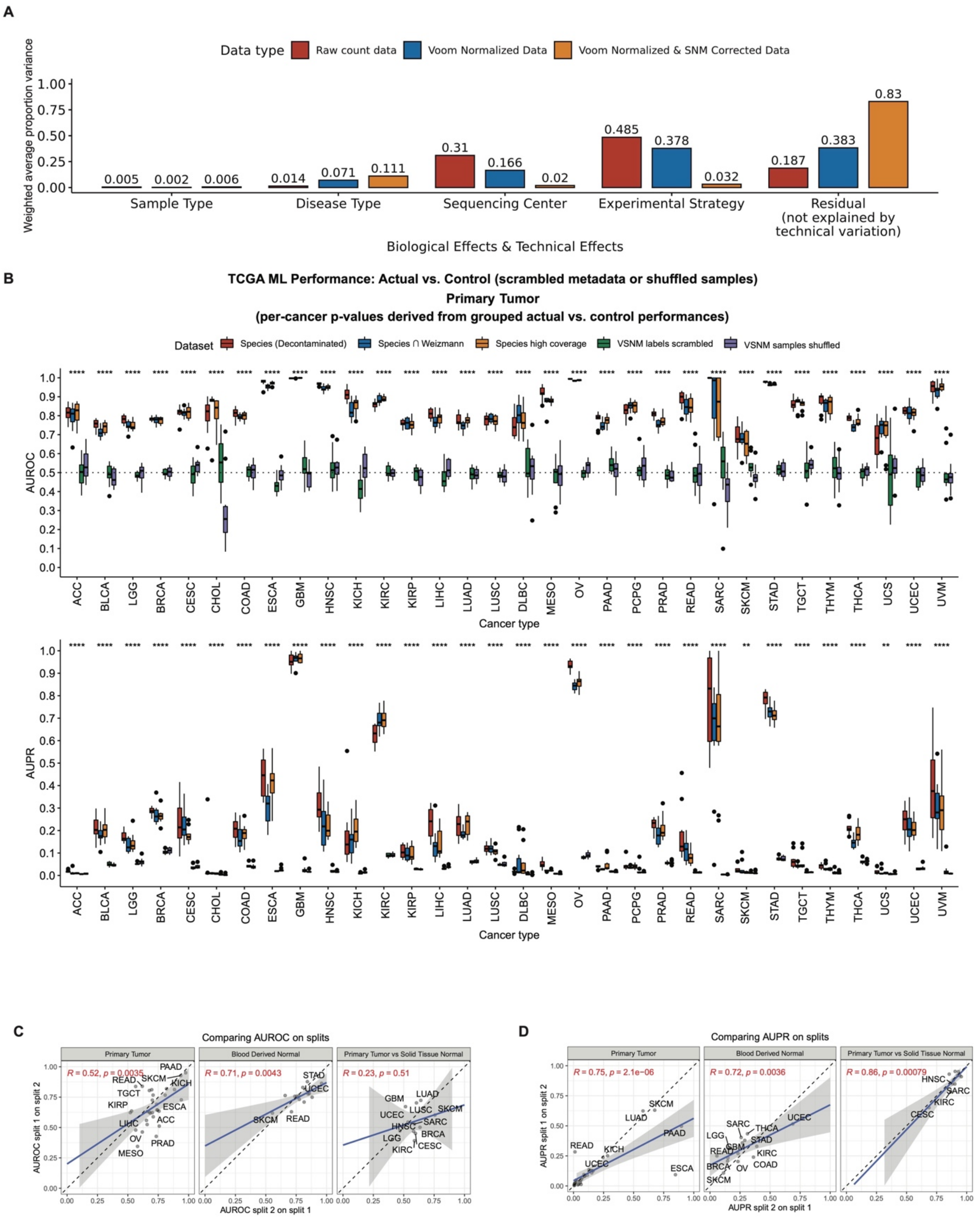

Data S5.6. TCGA batch correction and negative and positive controls on pan-cancer tumor sample machine learning

(**A**) Principal variance components analysis (PVCA) was applied to pre- and post-batch corrected TCGA decontaminated fungal data across all sample types to estimate the quality of Voom-SNM normalization (see STAR Methods). The results show up to 20.4-fold reduction in technical variance (experimental strategy) with up to 7.7-fold increase in biological signal. During the supervised batch correction, only TCGA "sample type" was included as the biological variable, so the relative increase in signal attributed to disease type can be thought of as being due to increased signal-to-noise ratio caused by the batch correction.
(**B**) Scrambled and shuffled machine learning negative controls were repeated on pan-cancer, batch-corrected primary tumor data and compared to performance using biological samples, which included 224 decontaminated fungal species, 34 WIS-overlapping fungal species, or 31 fungal species with ≥1% aggregate coverage. For hypothesis testing, biological data and scrambled/shuffled controls were aggregated into two separate groups, and two-sided Wilcox tests were applied per cancer type per performance metric (AUROC or AUPR). *: $p \leq 0.05$; **: $p \leq 0.01$; ***: $p \leq 0.001$; ****: $p \leq 0.0001$.
(**C-D**) As a positive control, two stratified halves of TCGA were split on the basis of sequencing center, sample type, and disease type, followed by Voom-SNM batch correction on each half, independently training one-cancer-type-versus-all-others or tumor versus NAT models on each half, and cross-testing the models on the other halves of batch corrected data. The (**C**) AUROC and (**D**) AUPR performances were then correlated. Pearson correlation coefficients (*R*) and concomitant p-values are shown. The goal is to obtain 1:1 (denoted by the dotted gray lines) or better performance between the comparisons on either stratified half.

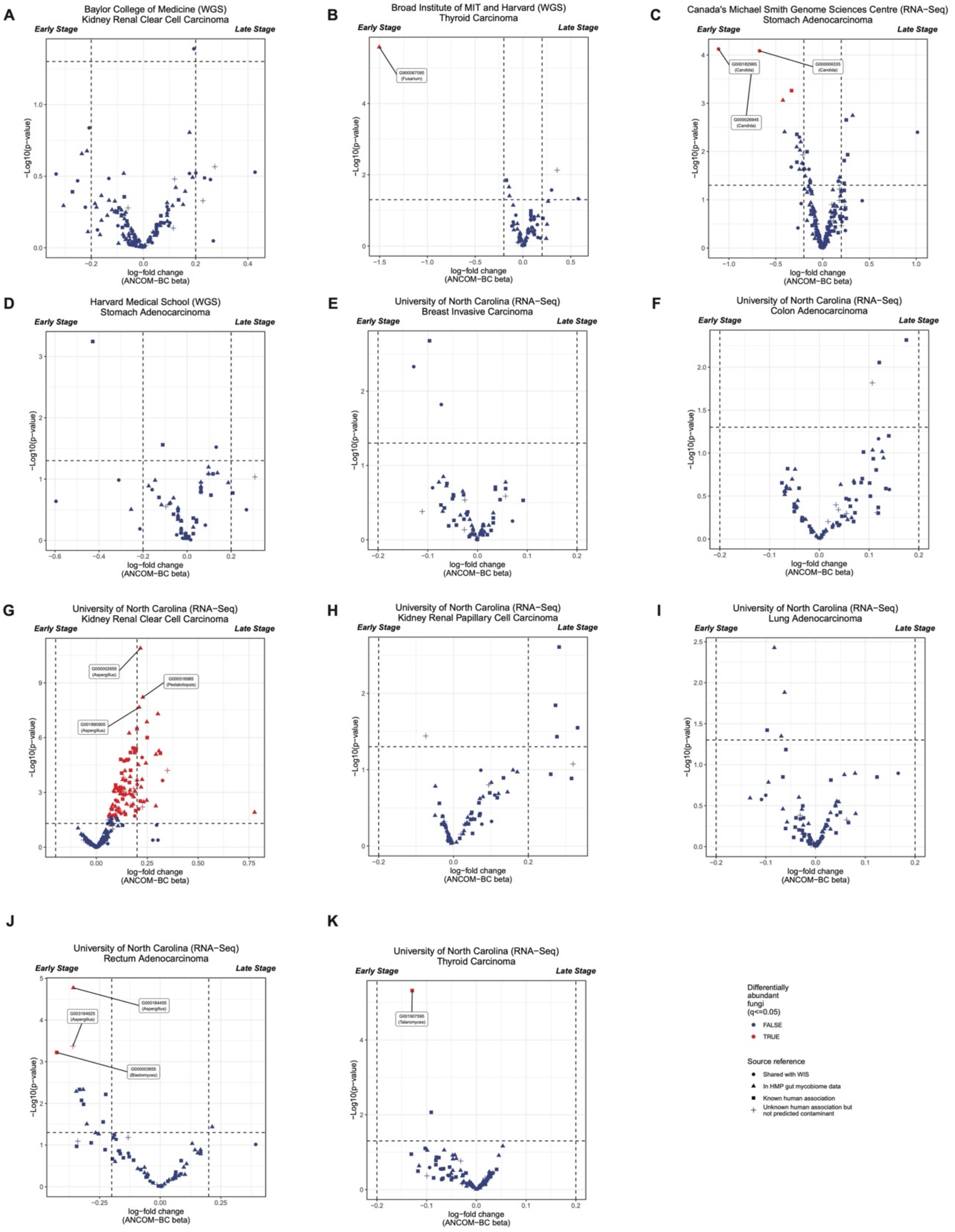

Data S5.7. Differential abundance volcano plots of stage I versus stage IV tumors using intratumoral decontaminated fungi in TCGA

(**A-K**) To calculate differential fungal abundances of stage I ("early stage") versus stage IV ("late stage") TCGA tumors, ANCOM-BC was applied to subsets of TCGA samples by a single

sequencing center, experimental strategy, and sequencing platform (see STAR Methods). Species-level fungal data were used. Volcano plots were then constructed using the log-fold change betas calculated by ANCOM-BC, colored red if fungi achieved a q-value≤0.05, and labeled by their reason for passing decontamination. The top 2-3 fungi in each plot by p-value smallness, if significant, were additionally labeled by its genome ID in the rep200 database and its genus name. A minimum of 10 samples per stage were required to be included in the testing. (**A**) Stage I versus IV comparisons in Baylor College of Medicine kidney renal clear cell carcinoma. (**B**) Stage I versus IV comparisons in Broad Institute thyroid carcinoma. (**C**) Stage I versus IV comparisons in Canada's Michael Smith Genome Science Centre stomach adenocarcinoma. (**D**) Stage I versus IV comparisons in Harvard Medical School stomach adenocarcinoma. (**E**) Stage I versus IV comparisons in University of North Carolina breast invasive carcinoma. (**F**) Stage I versus IV comparisons in University of North Carolina colon adenocarcinoma. (**G**) Stage I versus IV comparisons in University of North Carolina renal clear cell carcinoma. (**H**) Stage I versus IV comparisons in University of North Carolina renal papillary cell carcinoma. (**I**) Stage I versus IV comparisons in University of North Carolina lung adenocarcinoma. (**J**) Stage I versus IV comparisons in University of North Carolina rectum adenocarcinoma. (**K**) Stage I versus IV comparisons in University of North Carolina thyroid carcinoma.

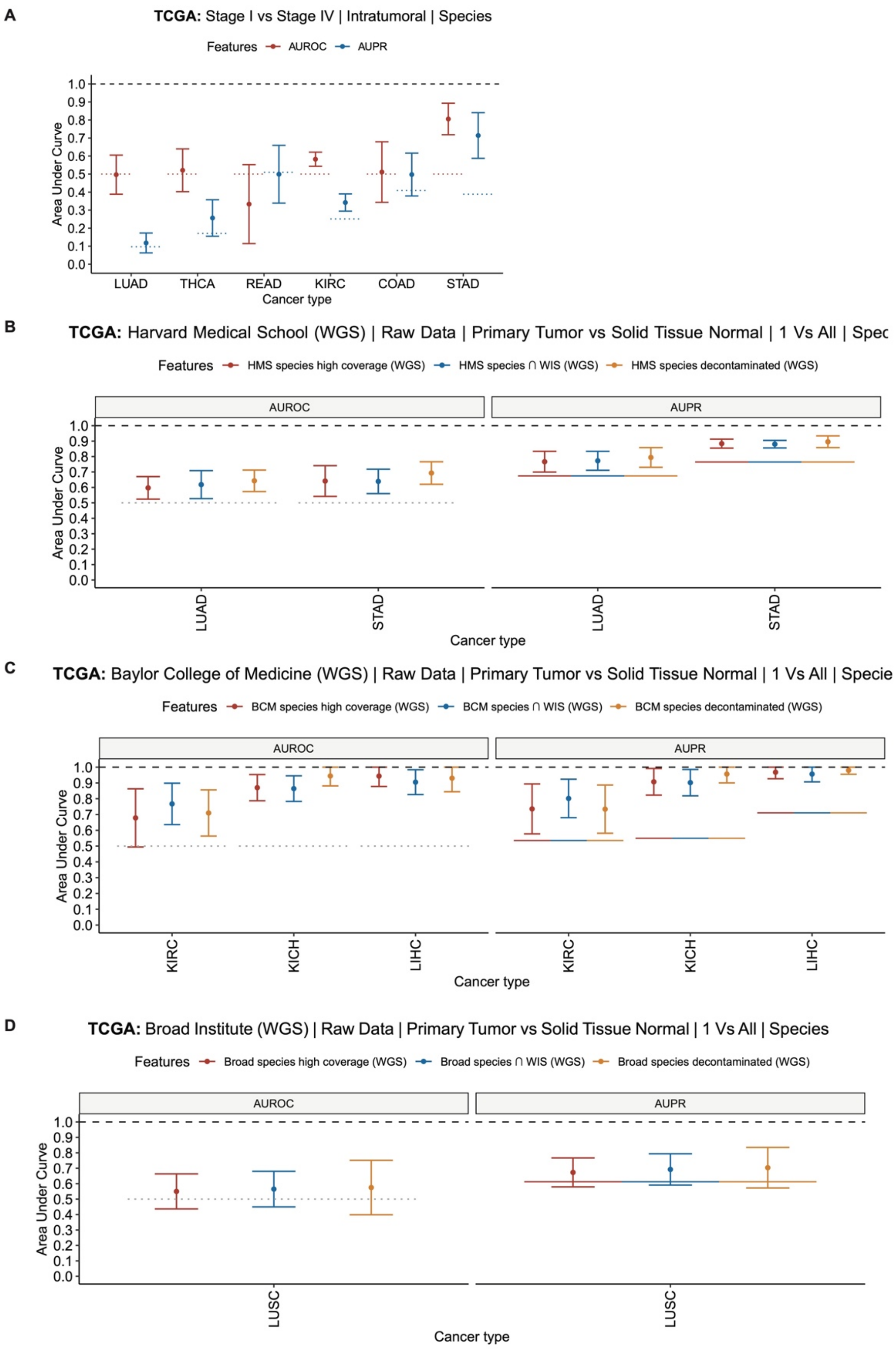
A
TCGA: Stage I vs Stage IV | Intratumoral | Species
Features AUROC AUPR
Area Under Curve
1.0 0.9 0.8 0.7 0.6 0.5 0.4 0.3 0.2 0.1 0.0
LUAD THCA READ KIRC COAD STAD
Cancer type
B
TCGA: Harvard Medical School (WGS) | Raw Data | Primary Tumor vs Solid Tissue Normal | 1 Vs All | Spec
Features HMS species high coverage (WGS) HMS species ∩ WIS (WGS) HMS species decontaminated (WGS)
AUROC
AUPR
Area Under Curve
1.0 0.9 0.8 0.7 0.6 0.5 0.4 0.3 0.2 0.1 0.0
LUAD STAD LUAD STAD
Cancer type
C
TCGA: Baylor College of Medicine (WGS) | Raw Data | Primary Tumor vs Solid Tissue Normal | 1 Vs All | Specie
Features BCM species high coverage (WGS) BCM species ∩ WIS (WGS) BCM species decontaminated (WGS)
AUROC
AUPR
Area Under Curve
1.0 0.9 0.8 0.7 0.6 0.5 0.4 0.3 0.2 0.1 0.0
KIRC KICH LIHC KIRC KICH LIHC
Cancer type
D
TCGA: Broad Institute (WGS) | Raw Data | Primary Tumor vs Solid Tissue Normal | 1 Vs All | Species
Features Broad species high coverage (WGS) Broad species ∩ WIS (WGS) Broad species decontaminated (WGS)
AUROC
AUPR
Area Under Curve
1.0 0.9 0.8 0.7 0.6 0.5 0.4 0.3 0.2 0.1 0.0
LUSC LUSC
Cancer type

E

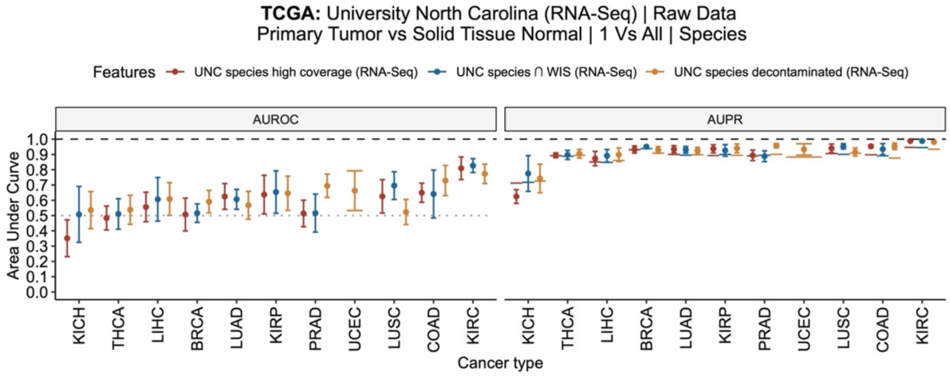
TCGA: University North Carolina (RNA-Seq) | Raw Data
Primary Tumor vs Solid Tissue Normal | 1 Vs All | Species
Features
UNC species high coverage (RNA-Seq)
UNC species ∩ WIS (RNA-Seq)
UNC species decontaminated (RNA-Seq)
AUROC
AUPR
Area Under Curve
1.0
0.9
0.8
0.7
0.6
0.5
0.4
0.3
0.2
0.1
0.0
KICH
THCA
LIHC
BRCA
LUAD
KIRP
PRAD
UCEC
LUSC
COAD
KIRC
Cancer type

F

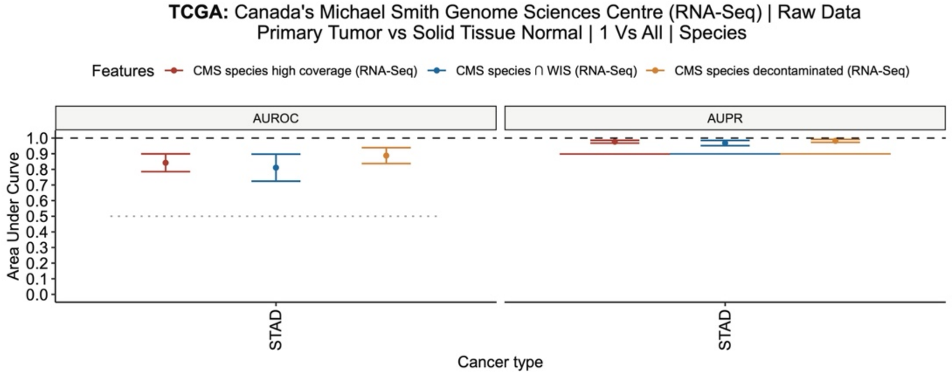
TCGA: Canada's Michael Smith Genome Sciences Centre (RNA-Seq) | Raw Data
Primary Tumor vs Solid Tissue Normal | 1 Vs All | Species
Features
CMS species high coverage (RNA-Seq)
CMS species ∩ WIS (RNA-Seq)
CMS species decontaminated (RNA-Seq)
AUROC
AUPR
Area Under Curve
1.0
0.9
0.8
0.7
0.6
0.5
0.4
0.3
0.2
0.1
0.0
STAD
STAD
Cancer type

G

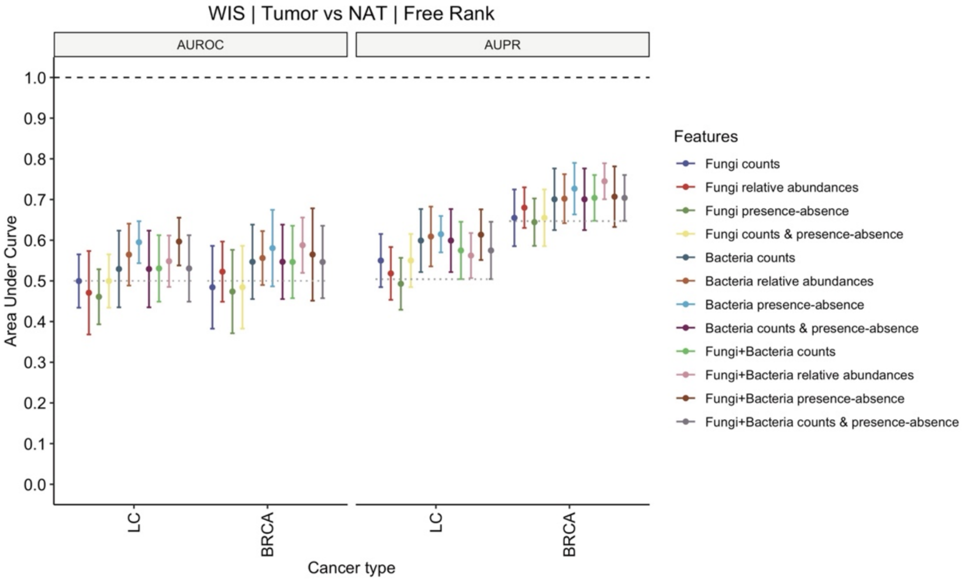
WIS | Tumor vs NAT | Free Rank
AUROC
AUPR
Area Under Curve
1.0
0.9
0.8
0.7
0.6
0.5
0.4
0.3
0.2
0.1
0.0
LC
BRCA
LC
BRCA
Cancer type
Features
Fungi counts
Fungi relative abundances
Fungi presence-absence
Fungi counts & presence-absence
Bacteria counts
Bacteria relative abundances
Bacteria presence-absence
Bacteria counts & presence-absence
Fungi+Bacteria counts
Fungi+Bacteria relative abundances
Fungi+Bacteria presence-absence
Fungi+Bacteria counts & presence-absence

H

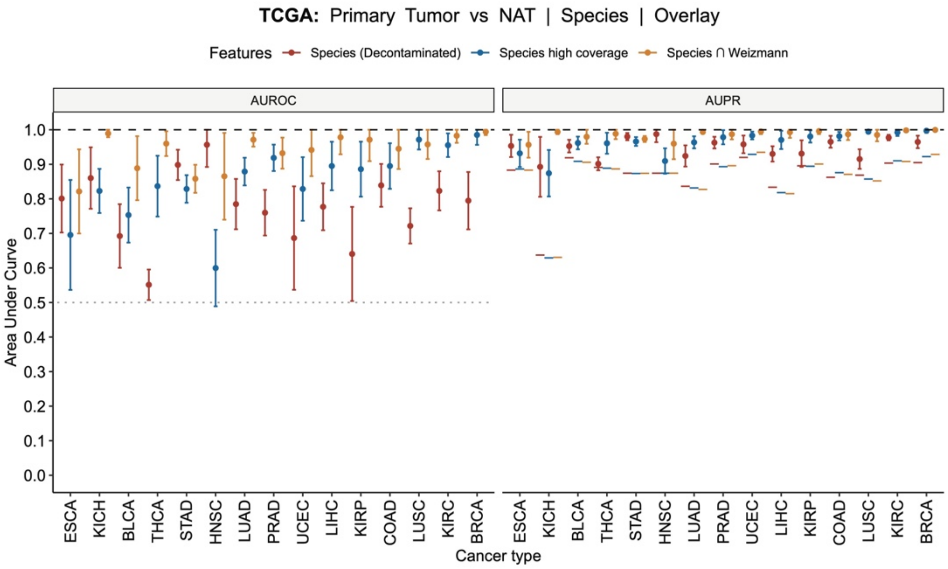

I

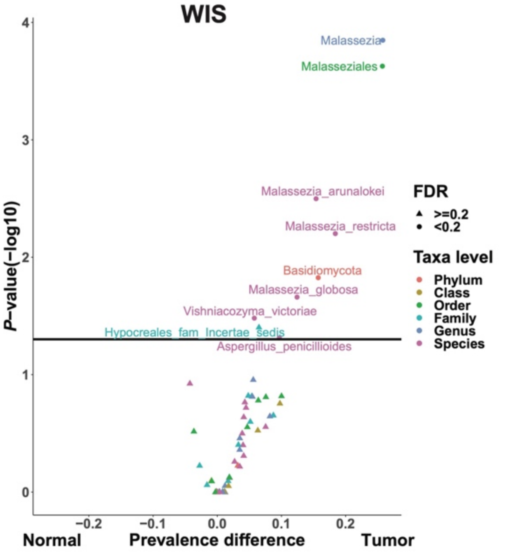

J

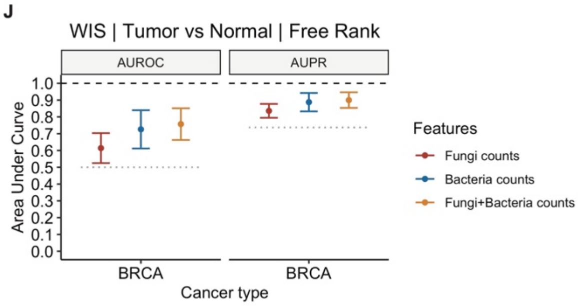

Data S5.8. TCGA machine learning between stage I and stage IV tumors, as well as tumor versus NAT samples

(**A**) Ten-fold cross-validation machine learning models tested to discriminate between stage I and stage IV tumors using batch corrected TCGA data. At least 20 samples in each class were required to be tested. AUROC (red) and AUPR (blue) performance shown, with dots denoting average values and error bars denoting 95% confidence intervals. Horizontal colored dots denote null values for AUROC (red dots) and AUPR (blue dots).

(**B-F**) Ten-fold cross-validation machine learning models tested to discriminate TCGA tumor versus NAT samples using raw count data subset to a single sequencing center, experimental strategy, and sequencing platform. Performances shown per cancer type for (**B**) Harvard Medical School (WGS) samples, (**C**) Baylor College of Medicine (WGS) samples, (**D**) Broad Institute (WGS) samples, (**E**) University of North Carolina (RNA-Seq) samples, (**F**) and Canada's Michael

Smith Genome Science Centre (RNA-Seq) samples. Dots denote average values and error bars denote 95% confidence intervals. Horizontal gray dots or colored bars denote the null AUROC and AUPR values, respectively. At least 20 samples in each class were required to be tested.
(**G**) Ten-fold cross-validation machine learning models tested to discriminate tumor versus NAT samples using the WIS cohort fungal, bacterial, or fungal and bacterial data comprising raw counts, relative abundances, binary presence-absence, or combined counts and presence-absence information. Microbial hits at all taxa levels that passed filtering were included ("free rank"). Dots denote average values and error bars denote 95% confidence intervals. Horizontal gray dots denote the null AUROC and AUPR values. At least 20 samples in each class were required to be tested.
(**H**) Ten-fold cross-validation machine learning models tested to discriminate TCGA tumor versus NAT samples using pan-cancer batch corrected data. Feature subsets included 224 decontaminated fungal species (red), 31 fungal species with ≥1% aggregate coverage ("high coverage"), and 34 fungal species that overlapped with the WIS cohort ("∩ Weizmann). Dots denote average values and error bars denote 95% confidence intervals. Horizontal gray dots or colored bars denote the null AUROC and AUPR values, respectively. Null AUPR values vary slightly when subsetting feature sets resulted in zero sum samples that had to be removed prior to batch correction and machine learning.
(**I**) Differential prevalence testing in the WIS cohort between breast cancer tumor samples and true normal breast tissue samples across all taxa levels. Taxa are colored by corresponding taxa level and shaped based on whether it met the FDR cutoff or not.
(**J**) Ten-fold cross-validation machine learning models built to discriminate WIS breast cancer tumor samples versus true normal breast tissue based on fungi, bacteria, or fungi and bacteria raw counts. Microbial hits at all taxa levels that passed filtering were included ("free rank"). Dots denote average performance and error bars denote 95% confidence intervals. Gray horizontal dots denote the null AUROC and AUPR values.

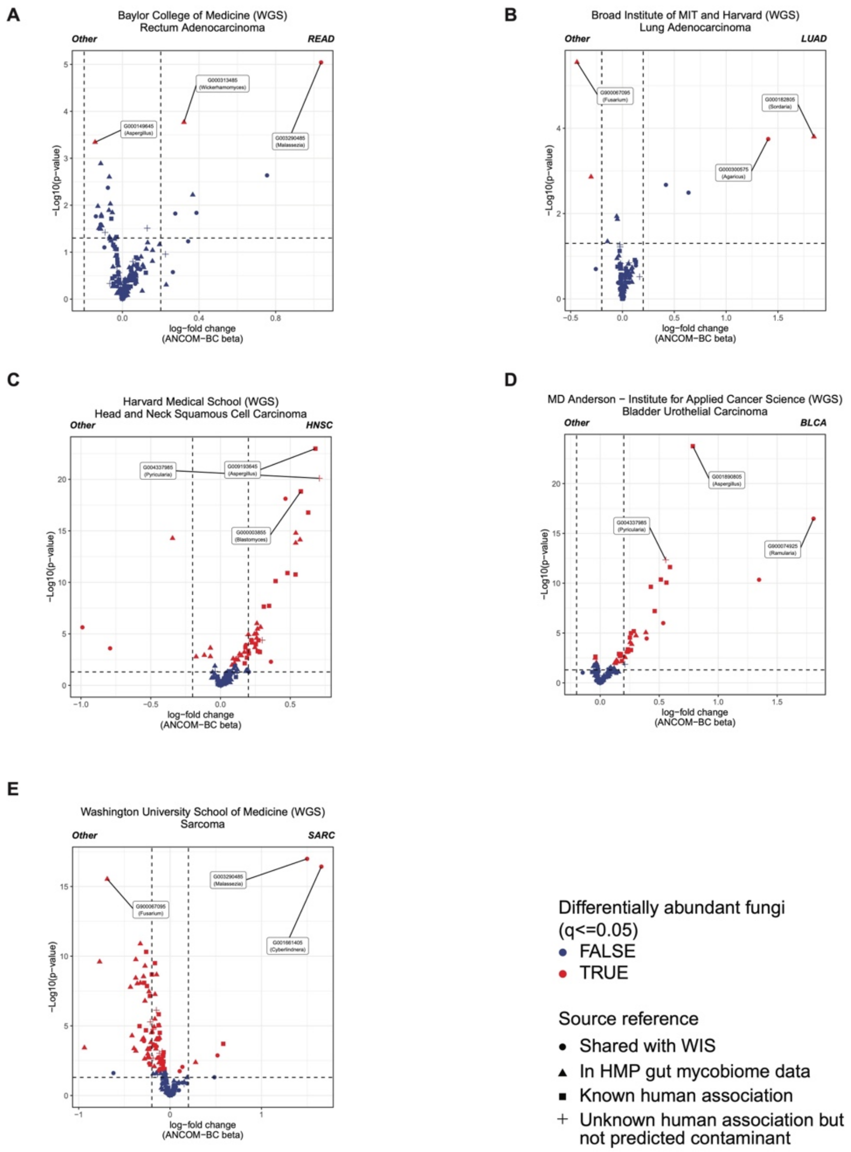

Data S5.9. Representative differential abundance volcano plots of one cancer type versus all others using blood-derived decontaminated fungi in TCGA

(**A-E**) To calculate differential fungal abundances on TCGA blood sample raw count data, ANCOM-BC was applied to subsets of TCGA in a single sequencing center and sequencing platform (see STAR Methods). TCGA blood samples only had WGS performed on them. Species-level fungal data were used. Volcano plots were then constructed using the log-fold change betas calculated by ANCOM-BC, colored red if fungi achieved a q-value≤0.05, and labeled by their reason for passing decontamination. The top 2-3 fungi in each plot by p-value smallness, if significant, were additionally labeled by its genome ID in the rep200 database and its genus name. A minimum of 10 samples per stage were required to be included in the testing. Results show differentially abundant fungi in (**A**) Baylor College of Medicine rectum cancer blood samples versus other cancer types, (**B**) Broad Institute lung cancer blood samples versus other cancer types,

(**C**) Harvard Medical School head and neck cancer blood samples versus other cancer types, (**D**) MD Anderson bladder cancer blood samples versus other cancer types, and (**E**) Washington University sarcoma cancer blood samples versus other cancer types.

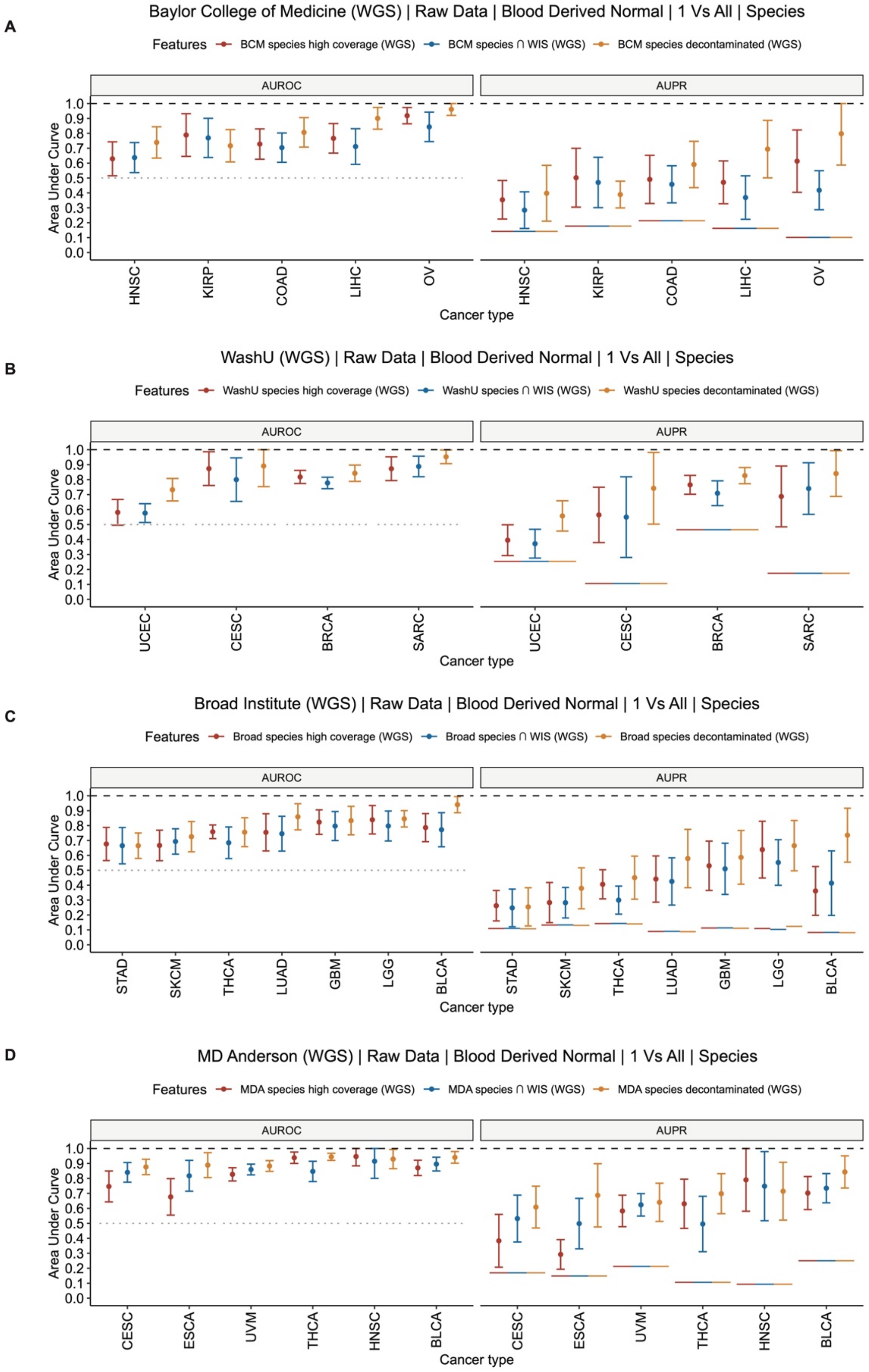
A
Baylor College of Medicine (WGS) | Raw Data | Blood Derived Normal | 1 Vs All | Species
Features
BCM species high coverage (WGS)
BCM species ∩ WIS (WGS)
BCM species decontaminated (WGS)
AUROC
AUPR
Area Under Curve
HNSC
KIRP
COAD
LIHC
OV
Cancer type
B
WashU (WGS) | Raw Data | Blood Derived Normal | 1 Vs All | Species
Features
WashU species high coverage (WGS)
WashU species ∩ WIS (WGS)
WashU species decontaminated (WGS)
AUROC
AUPR
Area Under Curve
UCEC
CESC
BRCA
SARC
Cancer type
C
Broad Institute (WGS) | Raw Data | Blood Derived Normal | 1 Vs All | Species
Features
Broad species high coverage (WGS)
Broad species ∩ WIS (WGS)
Broad species decontaminated (WGS)
AUROC
AUPR
Area Under Curve
STAD
SKCM
THCA
LUAD
GBM
LGG
BLCA
Cancer type
D
MD Anderson (WGS) | Raw Data | Blood Derived Normal | 1 Vs All | Species
Features
MDA species high coverage (WGS)
MDA species ∩ WIS (WGS)
MDA species decontaminated (WGS)
AUROC
AUPR
Area Under Curve
CESC
ESCA
UVM
THCA
HNSC
BLCA
Cancer type

Data S5.10. Machine learning on TCGA subsets of raw fungal count data to distinguish blood samples from one cancer type versus all others

(**A-D**) Decontaminated fungal count data were subset to a single sequencing center and sequencing platform (Illumina HiSeq) prior to ten-fold cross-validation machine learning. TCGA blood samples only had WGS performed on them. Species-level fungal data were used. Predictions were made on each of the ten holdout folds to generate average and 95% confidence intervals of discriminatory performance, as measured by AUROC and AUPR. A minimum of 20 samples were required in any comparison to be tested. Cancer type discrimination is shown among (**A**) Baylor College of Medicine samples, (**B**) Washington University ("WashU") samples, (**C**) Broad Institute samples, and (**D**) MD Anderson samples. Dots denote average values and error bars denote 95% confidence intervals. Horizontal gray dots or colored bars denote the null AUROC and AUPR values, respectively. Various feature sets for the machine learning included 224 decontaminated fungal species ("decontaminated"), 34 WIS-overlapping fungal species ("∩ WIS"), or 31 fungal species with ≥1% aggregate coverage ("high coverage").

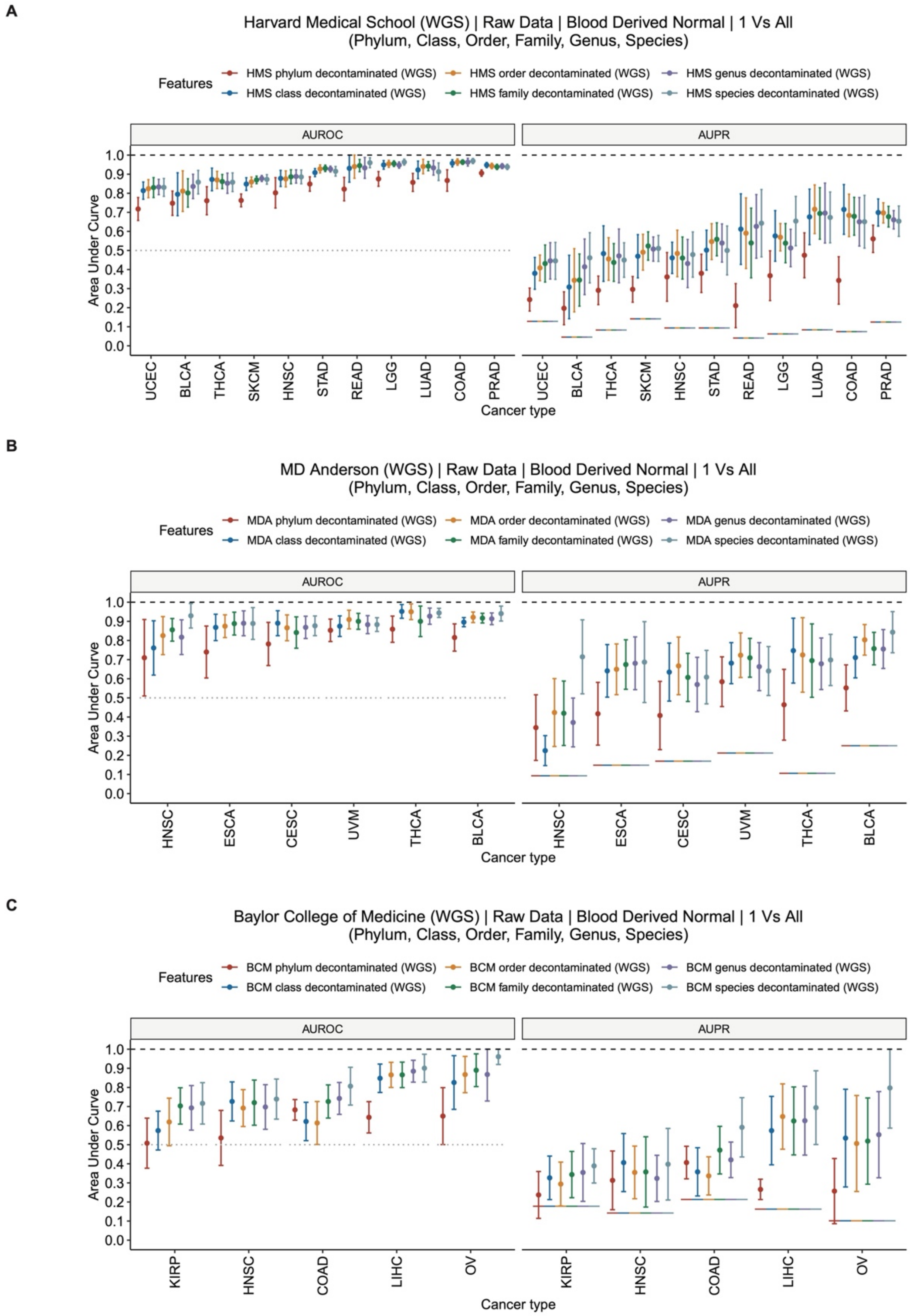
A
Harvard Medical School (WGS) | Raw Data | Blood Derived Normal | 1 Vs All
(Phylum, Class, Order, Family, Genus, Species)
Features
HMS phylum decontaminated (WGS)
HMS class decontaminated (WGS)
HMS order decontaminated (WGS)
HMS family decontaminated (WGS)
HMS genus decontaminated (WGS)
HMS species decontaminated (WGS)
AUROC
AUPR
Area Under Curve
UCEC BLCA THCA SKCM HNSC STAD READ LGG LUAD COAD PRAD
Cancer type
B
MD Anderson (WGS) | Raw Data | Blood Derived Normal | 1 Vs All
(Phylum, Class, Order, Family, Genus, Species)
Features
MDA phylum decontaminated (WGS)
MDA class decontaminated (WGS)
MDA order decontaminated (WGS)
MDA family decontaminated (WGS)
MDA genus decontaminated (WGS)
MDA species decontaminated (WGS)
AUROC
AUPR
Area Under Curve
HNSC ESCA CESC UVM THCA BLCA
Cancer type
C
Baylor College of Medicine (WGS) | Raw Data | Blood Derived Normal | 1 Vs All
(Phylum, Class, Order, Family, Genus, Species)
Features
BCM phylum decontaminated (WGS)
BCM class decontaminated (WGS)
BCM order decontaminated (WGS)
BCM family decontaminated (WGS)
BCM genus decontaminated (WGS)
BCM species decontaminated (WGS)
AUROC
AUPR
Area Under Curve
KIRP HNSC COAD LIHC OV
Cancer type

D

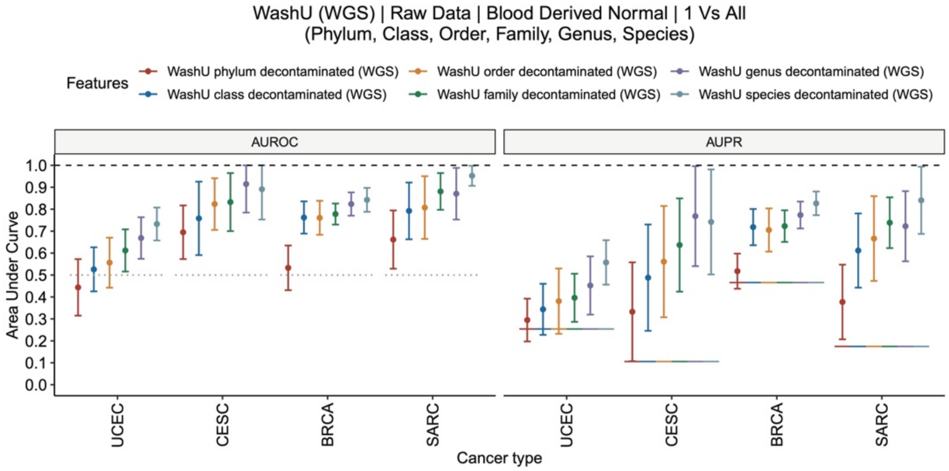

E

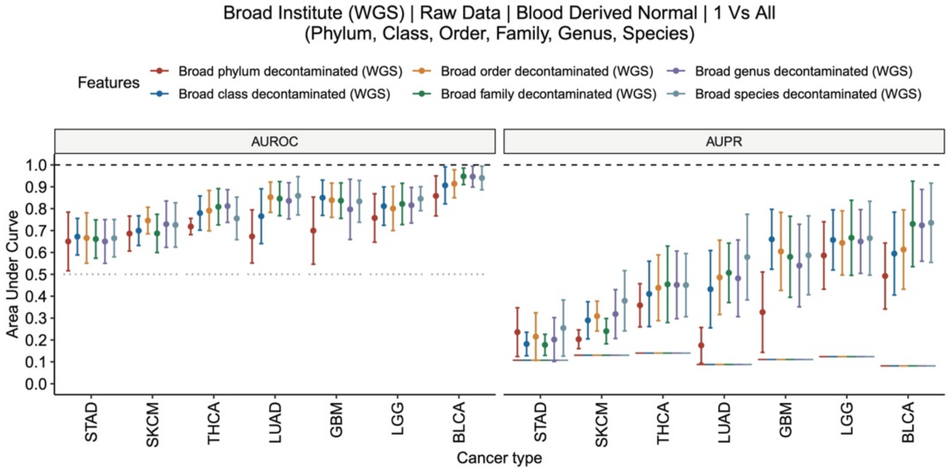

Data S5.11. Machine learning on TCGA subsets of raw fungal count data summarized to various taxa levels to distinguish blood samples from one cancer type versus all others
(**A-E**) Decontaminated fungal count data were subset to a single sequencing center and sequencing platform (Illumina HiSeq) prior to summarizing to various taxa levels and performing machine learning. TCGA blood samples only had WGS performed on them. Predictions were made on each of the ten holdout folds to generate average and 95% confidence intervals of discriminatory performance, as measured by AUROC and AUPR. A minimum of 20 samples were required in any comparison to be tested. Cancer type discrimination is shown among (**A**) Harvard Medical School samples, (**B**) MD Anderson samples, (**C**) Baylor College of Medicine samples (**D**) Washington University ("WashU") samples, and (**E**) Broad Institute samples. Dots in each plot denote average values and error bars denote 95% confidence intervals. Gray horizontal dotted lines under AUROC denote null values. Colored horizontal lines under AUPR denote null values, which equates the prevalence of the positive class (each cancer type) among the full set of all cancer types within a sequencing center subset.

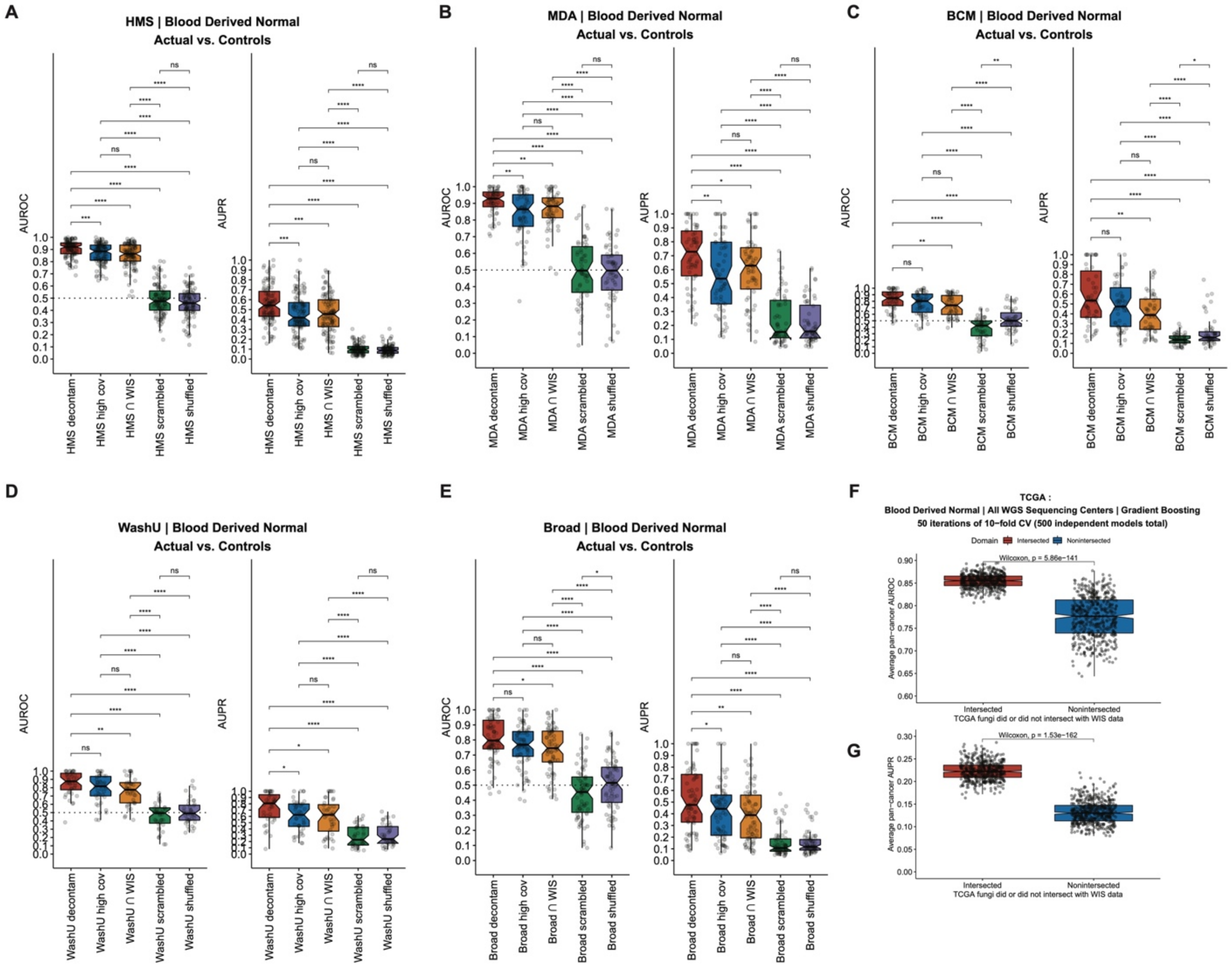

Data S5.12. Evaluating negative and positive controls for machine learning on TCGA blood raw data

(**A-E**) Machine learning models predicting one cancer type versus all others with blood-derived fungi were re-evaluated using scrambled metadata or shuffled count data within each cancer type and sequencing center subset and compared to performance on actual biological samples. Biological comparisons used 224 decontaminated fungal species (x-axis "decontam"), 31 fungal species with ≥1% aggregate genome coverage (x-axis "high cov"), or 34 fungal species overlapping with the WIS cohort (x-axis "∩ WIS"). Since each fold of the biological samples should show better than random performance, all folds from each cancer type comparison (i.e., ten from each cancer type) are included in the biological sample boxplots, and each fold from the scrambled or shuffled controls are also shown. Comparison of biological versus negative controls for (**A**) Harvard Medical School ("HMS") blood samples, (**B**) MD Anderson ("MDA") blood samples, (**C**) Baylor College of Medicine ("BCM") blood samples, (**D**) Washington University ("WashU") WGS blood samples, and (**E**) Broad Institute ("Broad") blood samples. Note that TCGA blood samples only had WGS performed on them. Pairwise two-sided Wilcox tests, corrected for multiple hypothesis testing using the Benjamini-Hochberg method, are shown. ns: not significant (q>0.05); *: q≤0.05; **: q≤0.01; ***: q≤0.001; ****: q≤0.0001.

(**F-G**) To test whether the 34 WIS-overlapping species provided greater discriminatory performance among TCGA blood samples than other detected fungi, pan-cancer, multi-class machine learning models were built using 10-fold cross-validation using only the 34 WIS-overlapping species or 34 non-WIS-overlapping randomly selected fungi. This process was repeated for 50 iterations (500 total folds), and AUROC (left) and AUPR (right) performance was calculated on each holdout fold. Two-sided Wilcox tests were used to test for significant differences. Note: All TCGA blood samples comprised WGS data.

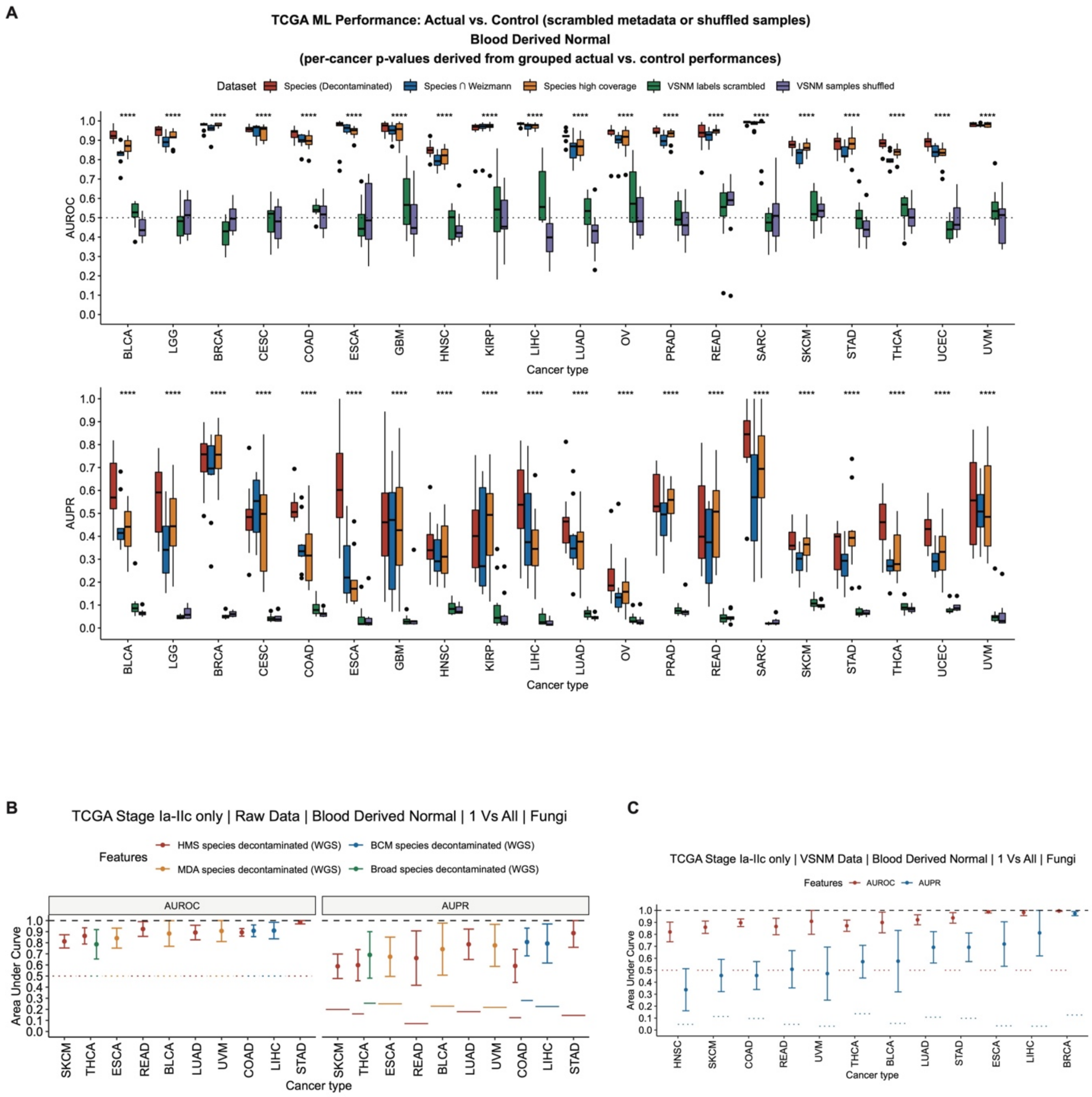

Data S5.13. TCGA batch correction and negative and positive controls on pan-cancer blood sample machine learning

(**A**) Scrambled and shuffled machine learning negative controls were repeated on pan-cancer, batch-corrected blood samples and compared to performance using biological samples, which included 224 decontaminated fungal species, 34 WIS-overlapping fungal species, or 31 fungal species with ≥1% aggregate coverage. For hypothesis testing, biological data and scrambled/shuffled controls were aggregated into two separate groups, and two-sided Wilcox tests were applied per cancer type per performance metric (AUROC or AUPR). *: p≤0.05; **: p≤0.01; ***: p≤0.001; ****: p≤0.0001.

(**B**) Ten-fold cross-validation machine learning models tested on raw, decontaminated fungal counts to discern one cancer type versus all others using stage Ia-IIc TCGA blood samples subset

to individual sequencing centers to preclude batch correction. At least 20 blood samples were required per cancer type per center to test; however, if a cancer type did not have ≥20 samples in an individual center, it was still included in the "Other" class during one-cancer-type-versus-all-others machine learning. Colors denote which sequencing center the comparison was made within, dots denote the average performance, and error bars indicate the 95% confidence intervals of performance. Horizontal, colored, dotted lines denote the null AUROC and AUPR values, with each color corresponding to the sequencing center.
(**C**) Ten-fold cross-validation machine learning models tested on pan-cancer, batch-corrected TCGA data to discern one cancer type versus all others using stage Ia-IIc TCGA blood samples. At least 20 blood samples were required per cancer type to test. Colors denote the performance metric (AUROC, red; AUPR, blue), dots denote the average performance, and error bars indicate the 95% confidence intervals of performance. Horizontal, colored, dotted lines denote the null AUROC (red) and AUPR (blue) values.

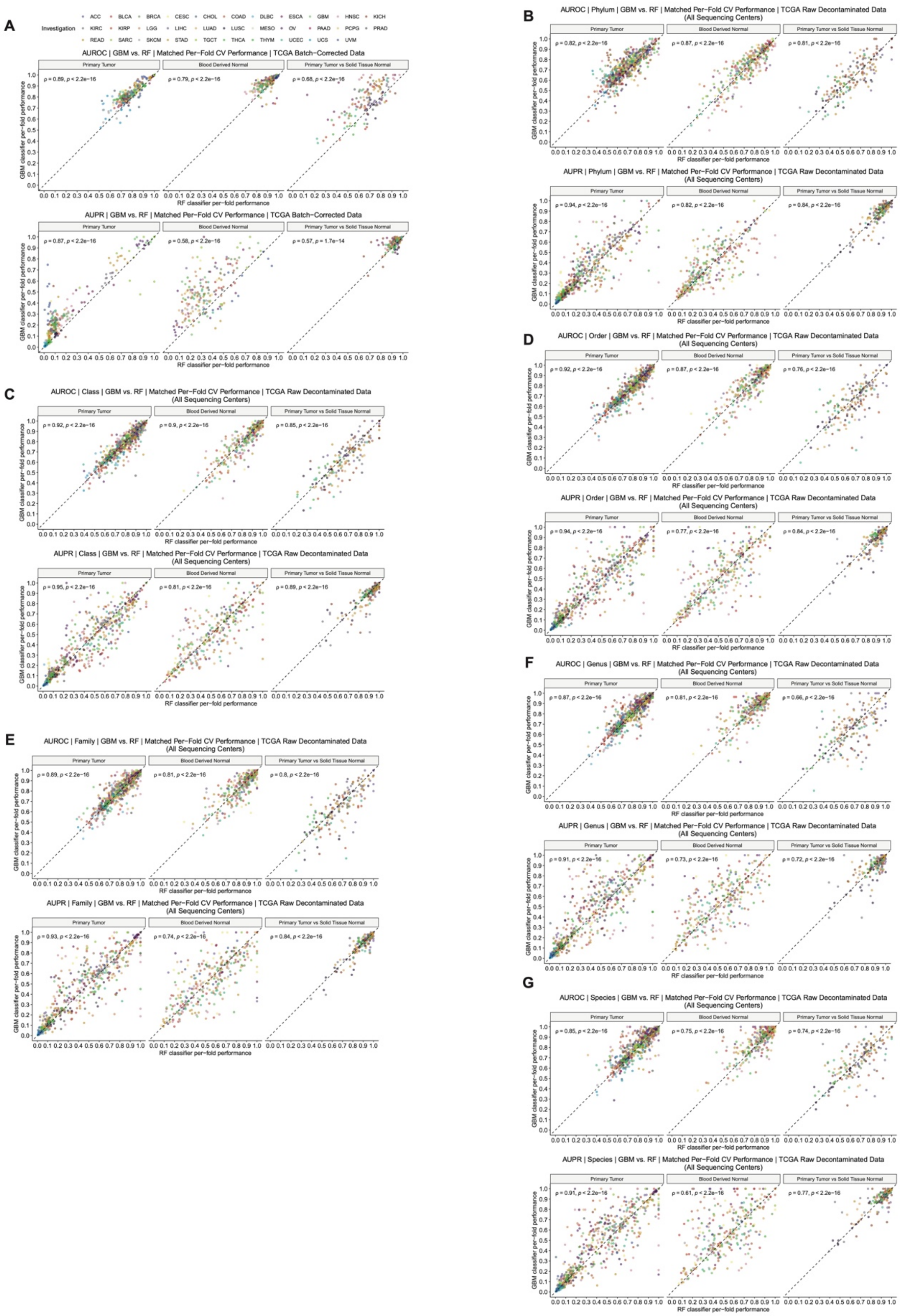
A
Investigation
ACC BLCA BRCA CESC CHOL COAD DLBC ESCA GBM HNSC KICH
KIRC KIRP LGG LIHC LUAD LUSC MESO OV PAAD PCPG PRAD
READ SARC SKCM STAD TGCT THCA THYM UCEC UCS UVM
AUROC | GBM vs. RF | Matched Per-Fold CV Performance | TCGA Batch-Corrected Data
Primary Tumor
Blood Derived Normal
Primary Tumor vs Solid Tissue Normal
ρ = 0.89, p < 2.2e−16
ρ = 0.79, p < 2.2e−16
ρ = 0.68, p < 2.2e−16
GBM classifier per−fold performance
RF classifier per−fold performance
AUPR | GBM vs. RF | Matched Per-Fold CV Performance | TCGA Batch-Corrected Data
ρ = 0.87, p < 2.2e−16
ρ = 0.58, p < 2.2e−16
ρ = 0.57, p = 1.7e−14
B
AUROC | Phylum | GBM vs. RF | Matched Per-Fold CV Performance | TCGA Raw Decontaminated Data
(All Sequencing Centers)
ρ = 0.82, p < 2.2e−16
ρ = 0.87, p < 2.2e−16
ρ = 0.81, p < 2.2e−16
AUPR | Phylum | GBM vs. RF | Matched Per-Fold CV Performance | TCGA Raw Decontaminated Data
(All Sequencing Centers)
ρ = 0.94, p < 2.2e−16
ρ = 0.82, p < 2.2e−16
ρ = 0.84, p < 2.2e−16
C
AUROC | Class | GBM vs. RF | Matched Per-Fold CV Performance | TCGA Raw Decontaminated Data
(All Sequencing Centers)
ρ = 0.92, p < 2.2e−16
ρ = 0.9, p < 2.2e−16
ρ = 0.85, p < 2.2e−16
AUPR | Class | GBM vs. RF | Matched Per-Fold CV Performance | TCGA Raw Decontaminated Data
(All Sequencing Centers)
ρ = 0.95, p < 2.2e−16
ρ = 0.81, p < 2.2e−16
ρ = 0.89, p < 2.2e−16
D
AUROC | Order | GBM vs. RF | Matched Per-Fold CV Performance | TCGA Raw Decontaminated Data
(All Sequencing Centers)
ρ = 0.92, p < 2.2e−16
ρ = 0.87, p < 2.2e−16
ρ = 0.76, p < 2.2e−16
AUPR | Order | GBM vs. RF | Matched Per-Fold CV Performance | TCGA Raw Decontaminated Data
(All Sequencing Centers)
ρ = 0.94, p < 2.2e−16
ρ = 0.77, p < 2.2e−16
ρ = 0.84, p < 2.2e−16
E
AUROC | Family | GBM vs. RF | Matched Per-Fold CV Performance | TCGA Raw Decontaminated Data
(All Sequencing Centers)
ρ = 0.89, p < 2.2e−16
ρ = 0.81, p < 2.2e−16
ρ = 0.8, p < 2.2e−16
AUPR | Family | GBM vs. RF | Matched Per-Fold CV Performance | TCGA Raw Decontaminated Data
(All Sequencing Centers)
ρ = 0.93, p < 2.2e−16
ρ = 0.74, p < 2.2e−16
ρ = 0.84, p < 2.2e−16
F
AUROC | Genus | GBM vs. RF | Matched Per-Fold CV Performance | TCGA Raw Decontaminated Data
(All Sequencing Centers)
ρ = 0.87, p < 2.2e−16
ρ = 0.81, p < 2.2e−16
ρ = 0.66, p < 2.2e−16
AUPR | Genus | GBM vs. RF | Matched Per-Fold CV Performance | TCGA Raw Decontaminated Data
(All Sequencing Centers)
ρ = 0.91, p < 2.2e−16
ρ = 0.73, p < 2.2e−16
ρ = 0.72, p < 2.2e−16
G
AUROC | Species | GBM vs. RF | Matched Per-Fold CV Performance | TCGA Raw Decontaminated Data
(All Sequencing Centers)
ρ = 0.85, p < 2.2e−16
ρ = 0.75, p < 2.2e−16
ρ = 0.74, p < 2.2e−16
AUPR | Species | GBM vs. RF | Matched Per-Fold CV Performance | TCGA Raw Decontaminated Data
(All Sequencing Centers)
ρ = 0.91, p < 2.2e−16
ρ = 0.81, p < 2.2e−16
ρ = 0.77, p < 2.2e−16

Data S5.14. Testing other machine learning model types for cancer type discrimination in TCGA using batch-corrected and raw decontaminated data

(**A-G**) Random forest (RF) machine learning models using fixed hyperparameters (n_trees=500, mtry=4) were applied to the same independent kth-folds as the gradient boosting machine (GBM) learning analyses across all TCGA disease types, sample types, and sequencing center subsets (as needed). Keeping the rest of the modeling procedure equivalent enabled direct comparison between RF and GBM performances. As before, AUROC and AUPR were calculated on each independent kth-fold. Machine learning performance between RFs and GBMs were compared for all sample types across (**A**) batch-corrected species-level data, (**B**) phylum-level raw decontaminated data, (**C**) class-level raw decontaminated data, (**D**) order-level raw decontaminated data, (**E**) family-level raw decontaminated data, (**F**) genus-level raw decontaminated data, and (**G**) species-level raw decontaminated data. Raw decontaminated count data at various taxa levels were generated by taking the decontaminated species and aggregating at each taxonomy level. Raw data machine learning comparisons were calculated within each TCGA sequencing center separately but are plotted here in aggregate for brevity. Spearman correlation statistics and concomitant p-values are inset on every plot. AUROCs shown on the first row of each subfigure and AUPRs shown on the second row of each subfigure. Colors denote TCGA cancer types.

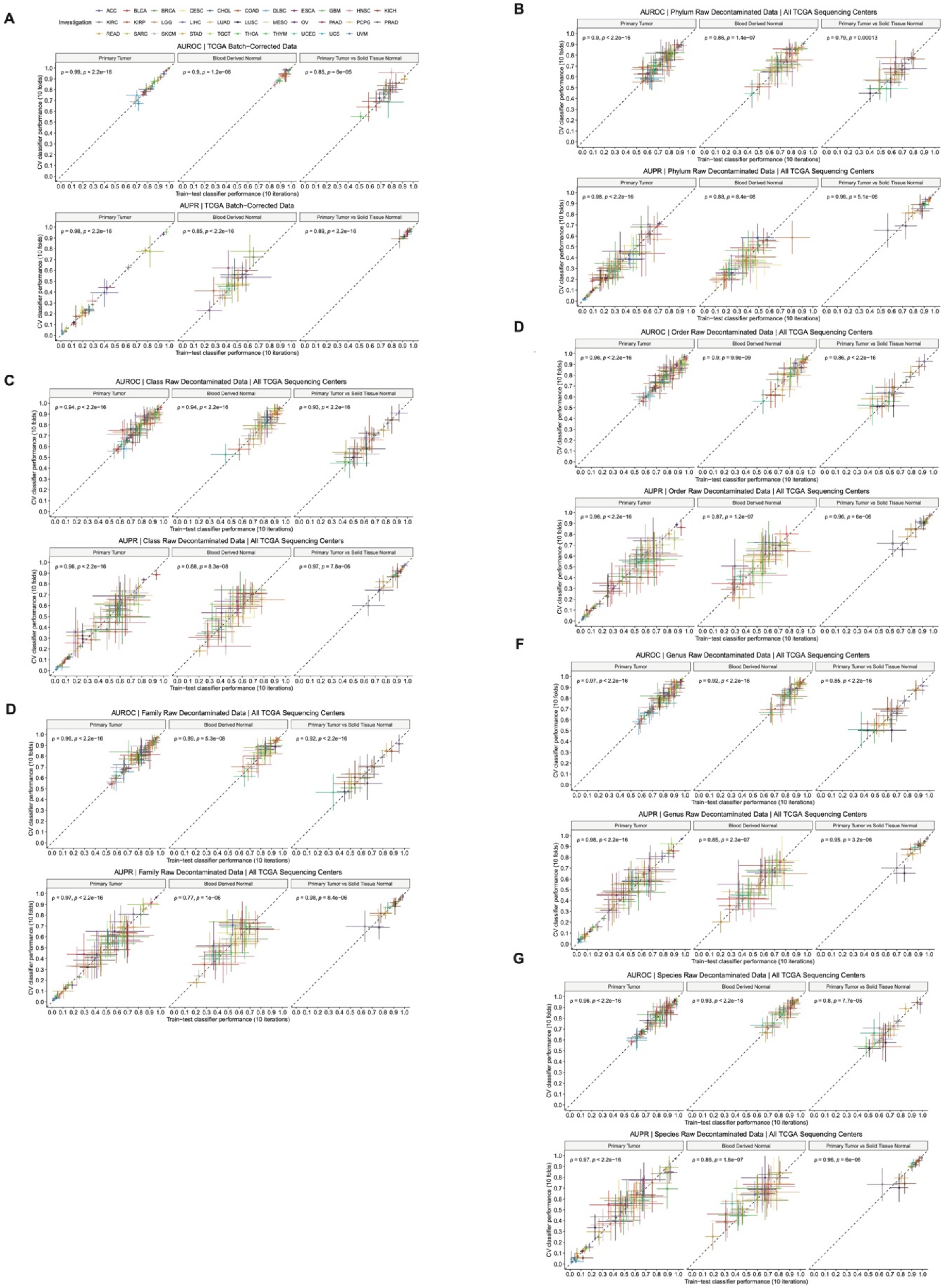
A
Investigation
ACC BLCA BRCA CESC CHOL COAD DLBC ESCA GBM HNSC KICH
KIRC KIRP LGG LIHC LUAD LUSC MESO OV PAAD PCPG PRAD
READ SARC SKCM STAD TGCT THCA THYM UCEC UCS UVM
AUROC | TCGA Batch-Corrected Data
Primary Tumor
Blood Derived Normal
Primary Tumor vs Solid Tissue Normal
AUPR | TCGA Batch-Corrected Data
B
AUROC | Phylum Raw Decontaminated Data | All TCGA Sequencing Centers
AUPR | Phylum Raw Decontaminated Data | All TCGA Sequencing Centers
C
AUROC | Class Raw Decontaminated Data | All TCGA Sequencing Centers
AUPR | Class Raw Decontaminated Data | All TCGA Sequencing Centers
D
AUROC | Order Raw Decontaminated Data | All TCGA Sequencing Centers
AUPR | Order Raw Decontaminated Data | All TCGA Sequencing Centers
D
AUROC | Family Raw Decontaminated Data | All TCGA Sequencing Centers
AUPR | Family Raw Decontaminated Data | All TCGA Sequencing Centers
F
AUROC | Genus Raw Decontaminated Data | All TCGA Sequencing Centers
AUPR | Genus Raw Decontaminated Data | All TCGA Sequencing Centers
G
AUROC | Species Raw Decontaminated Data | All TCGA Sequencing Centers
AUPR | Species Raw Decontaminated Data | All TCGA Sequencing Centers
CV classifier performance (10 folds)
Train−test classifier performance (10 iterations)

Data S5.15. Testing other sampling strategies during machine learning for cancer type discrimination in TCGA using batch-corrected and raw decontaminated data

(**A-G**) To ensure that the ten-fold cross-validation (CV) procedure was not artificially inflating the machine learning performances, an alternative sampling strategy of iterative 90% training-10% holdout testing splits, repeated ten times per comparison, was implemented. In this process, 90% of the data are set aside for training the gradient boosting machine learning model using internal four-fold cross-validation, followed by testing on the held out 10% test set, on which AUROC and AUPR are calculated. These steps are repeated ten times for each machine learning comparison to calculate a 95% confidence interval of AUROC and AUPR performance. The rest of the machine learning processes were kept the same across all TCGA disease types, sample types, and sequencing center subsets (as needed), enabling direct comparison between the 95% confidence intervals for the 10-fold CV and repeated train-test sampling procedures. Machine learning performances were then compared for all sample types across (**A**) batch-corrected species-level data, (**B**) phylum-level raw decontaminated data, (**C**) class-level raw decontaminated data, (**D**) order-level raw decontaminated data, (**E**) family-level raw decontaminated data, (**F**) genus-level raw decontaminated data, and (**G**) species-level raw decontaminated data. Raw decontaminated count data at various taxa levels were generated by taking the decontaminated species and aggregating at each taxonomy level. Raw data machine learning comparisons were calculated within each TCGA sequencing center separately but are plotted here in aggregate for brevity. Spearman correlation statistics and concomitant p-values are inset on every plot. AUROCs shown on the first row of each subfigure and AUPRs shown on the second row of each subfigure. Colors denote TCGA cancer types.
