# Supplementary material for: Pan-cancer analyses reveal cancer-type-specific fungal ecologies and bacteriome interactions
Source: Cell. 2022 Sep 29;185(20):3789–3806.e17. doi: 10.1016/j.cell.2022.09.005 (PMC9567272; doi:10.1016/j.cell.2022.09.005)
Supplement: Data S6. Clinical associations and utility of fungal DNA in tumors or plasma, related to Figure 6 [file mmc14.pdf]

Pan-cancer analyses reveal cancer type-specific fungal ecologies and bacteriome interactions

DATA S6

Clinical associations and utility of fungal DNA in tumors or plasma, related to **Figure 6**.

Table of Contents

Data S6.1. Clinical associations of fungi in intratumor and plasma samples 2

Data S6.2. Evaluating the clinical utility of fungal DNA in plasma samples 4

Data S6.3. Additional clinical and control analyses of fungal DNA in UCSD cohort plasma samples 6

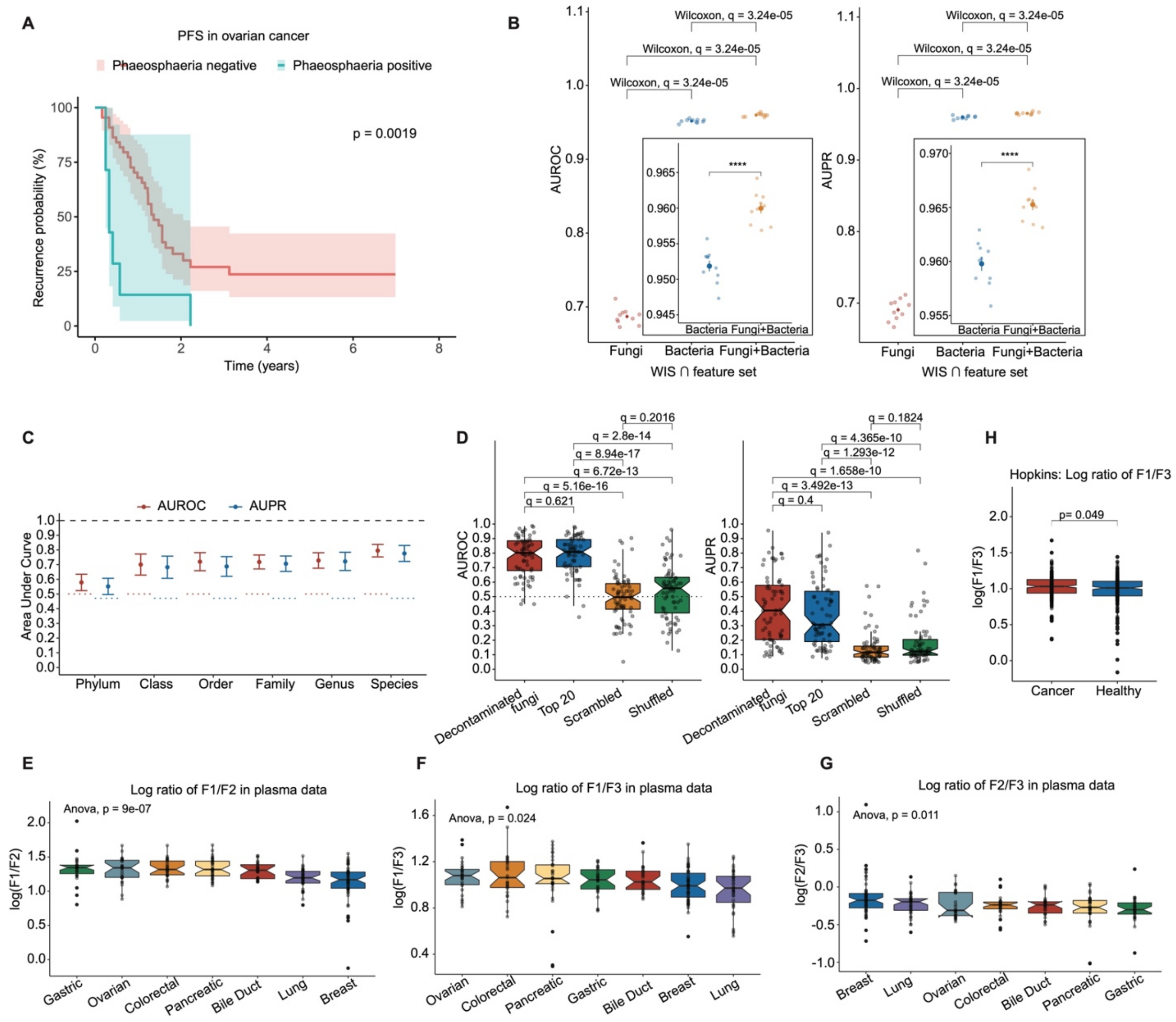

Data S6.1. Clinical associations of fungi in intratumor and plasma samples

(**A**) Kaplan-Meier plot demonstrating the progression free survival probability in ovarian cancer patients from the WIS cohort that were found to be positive (n=8) or negative (n=46) for the genus *Phaeosphaeria*. P-value was calculated by the log-rank test.

(**B**) Ten-fold cross-validation repeated ten times was performed on the Hopkins cohort using WIS-overlapping fungi, bacteria, or fungi and bacteria to discriminate between pan-cancer patients (8 cancer types) and healthy individuals. AUROC (left) and AUPR (right) performance values were measured across each repetition and are shown on the plot. Centered dots denote average performance and error bars denote 99% confidence intervals. Two-sided Wilcox tests, corrected for multiple comparisons using the Benjamini-Hochberg method, are shown. The inset plots show the performance comparison between bacteria versus fungi and bacteria, revealing synergistic performance gains.

(**C**) Prior to performance machine learning discriminating pan-cancer versus healthy individuals in the Hopkins cohort, decontaminated fungal counts were summarized at various taxonomic levels. Ten-fold cross-validation machine learning then tested the discriminatory performance at each taxonomic level, as shown by AUROC (red) and AUPR (blue). Centered dots denote average

performances, and error bars denote 95% confidence intervals. Horizontal, colored, dotted lines represent null performance values for AUROC (red) and AUPR (blue).

(**D**) As negative controls in the Hopkins cohort, machine learning models predicting each cancer type versus healthy individuals with plasma-derived fungi were re-evaluated using scrambled metadata or shuffled count data and compared to performance on actual biological samples. Biological comparisons used 209 decontaminated fungal species (x-axis "decontaminated") or the top 20 ranked fungi found to be most important in the pan-cancer versus healthy modeling (ten-fold cross-validation repeated ten-times). Since each fold of the biological samples comparing cancer versus healthy should show better than random performance, all folds from each cancer type (i.e., ten from each cancer type) are included in the biological sample boxplots, and each fold from the scrambled or shuffled controls are also shown. Pairwise two-sided Wilcox tests, corrected for multiple hypothesis testing using the Benjamini-Hochberg method, are shown on AUROC (left) and AUPR (right) data.

(**E-G**) Fungi identified in the TCGA mycotypes were compared in the Hopkins cohort using log-ratios to test if significant cancer type variation existed. Analogous to TCGA, this involved summarizing count data to the genus level, intersecting the taxa with those found in the mycotypes, and calculating the log-ratios with a pseudocount of 1 to minimize sample dropout. One-way ANOVAs were then calculated to infer cancer type variation of the log-ratios for the (**E**) F1/F2 fungal comparisons, (**F**) F1/F3 fungal comparisons, and (**G**) F2/F3 fungal comparisons.

(**H**) Log-ratios of Hopkins-associated fungi identified in the TCGA mycotypes were also calculated between grouped cancer plasma samples versus healthy plasma samples, and the F1/F3 comparison was found to be significantly different by a two-sided Wilcox test.

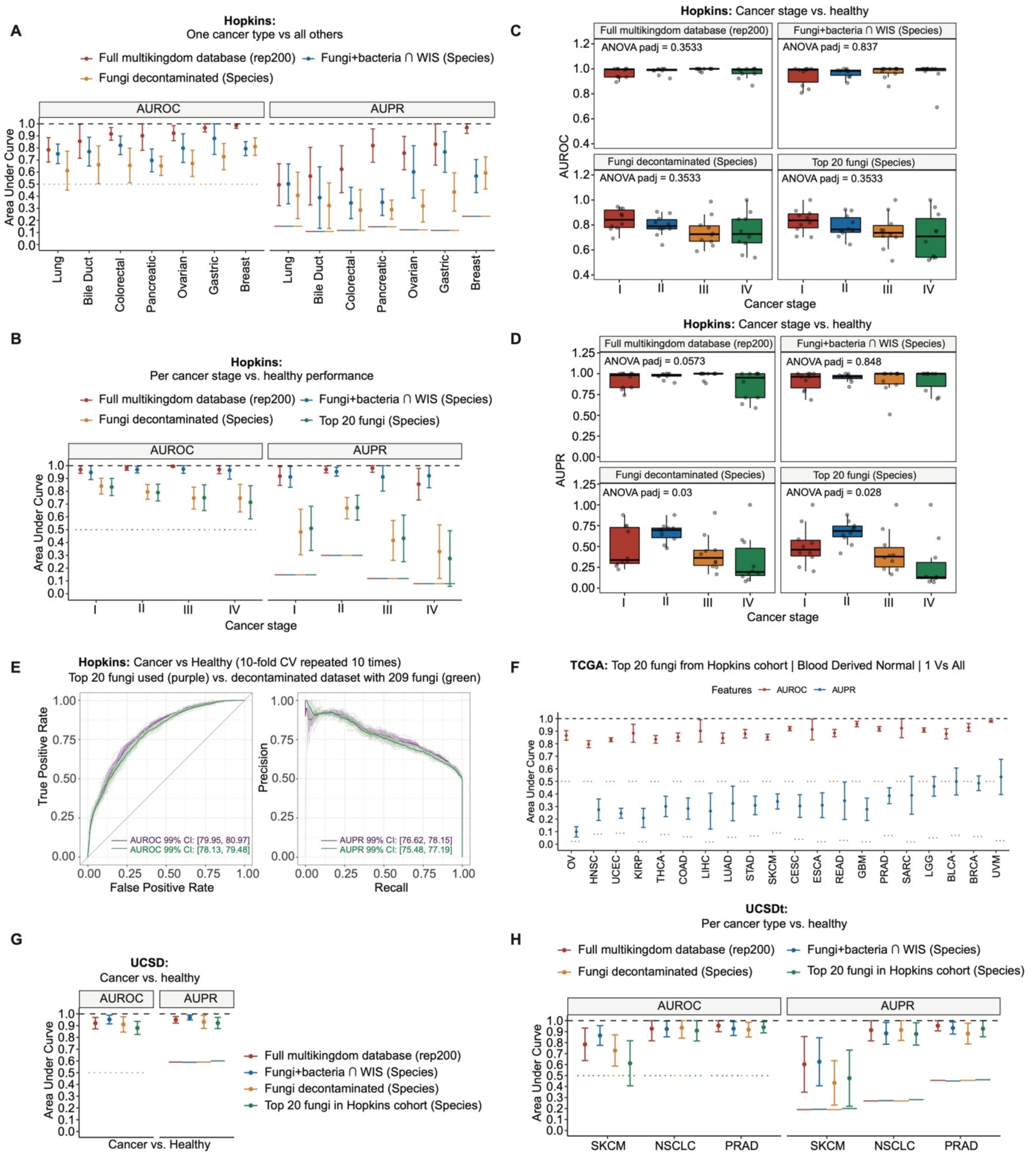

Data S6.2. Evaluating the clinical utility of fungal DNA in plasma samples

(**A**) Each cancer type versus all others machine learning performance in the Hopkins plasma cohort across three feature sets: all microbial hits against the rep200 database (red; 7418 features), only fungal and bacterial species overlapping with the WIS cohort (blue; 287 species), and decontaminated fungi (209 species).

(**B**) Per cancer stage versus healthy machine learning performance in the Hopkins plasma cohort across three feature sets: all microbial hits against the rep200 database (red; 7418 features), only fungal and bacterial species overlapping with the WIS cohort (blue; 287 species), decontaminated

fungi (209 species), and the top 20 ranked fungal species identified during pan-cancer versus healthy machine learning (ten-fold cross-validation repeated ten times).

(**C-D**) Stage-invariant discriminatory performance between pan-cancer versus healthy plasma samples in the Hopkins cohort. Each dataset shown in (**B**) is shown in a separate panel and a one-way ANOVA, corrected for multiple testing using the Benjamini-Hochberg method, is calculated across per-stage (**C**) AUROC or (**D**) AUPR performance values. Ten performance values from each ten folds comprise the box plot data.

(**E**) The top 20 fungal species identified in the Hopkins cohort pan-cancer versus healthy machine learning perform slightly but significantly better than the set of 209 decontaminated fungal species. Shown here is ten-fold cross-validation repeated ten times to discriminate pan-cancer versus healthy plasma samples for both the top 20 fungal species and the 209 decontaminated fungal species. AUROC and AUPR were calculated on each of the 10 repeats, and the corresponding ROC and PR curves for each repeat are shown as gray lines. Colored average ROC and PR curves are overlaid on the plot with colored ribbons representing the 99% confidence intervals along each of the average ROC or PR curves. Summary area performances are inset on the plot and colored with respect to the feature set.

(**F**) The top 20 fungal species identified in the Hopkins plasma cohort, discerning pan-cancer versus healthy, were used to test pan-cancer discrimination in the pan-cancer, batch corrected TCGA data using one-cancer-type-versus-all-others modeling with ten-fold cross-validation. Centered dots denote average AUROC (red) or AUPR (blue) performance per TCGA cancer type, and error bars represent the 95% confidence intervals. Horizontal, dotted, colored lines represent the null AUROC (red) and AUPR (blue) values.

(**G**) Pan-cancer versus healthy discriminatory performance in the UCSD cohort using various batch corrected feature sets (see STAR Methods): all microbial hits against the rep200 database (red; 7777 features), only fungal and bacterial species overlapping with the WIS cohort (blue; 281 species), decontaminated fungi (215 species), and the top 20 ranked fungal species identified in the Hopkins cohort during pan-cancer versus healthy machine learning (18 species overlapped with the UCSD cohort and were used).

(**H**) Per cancer versus healthy discriminatory performance in the UCSD cohort using the same batch corrected feature sets shown in (**G**).

(**A, B, G, H**) Performance estimated using ten-fold cross-validation, with predictions on each holdout fold to calculate AUROC and AUPR. Centered dots denote average performance and error bars represent 95% confidence intervals. Horizontal, dotted, gray or colored lines represent null AUROC and AUPR values, respectively. Null AUPR values may slightly vary between feature sets when subsetting resulted in zero-sum samples that had to be removed prior to batch correction and/or machine learning.

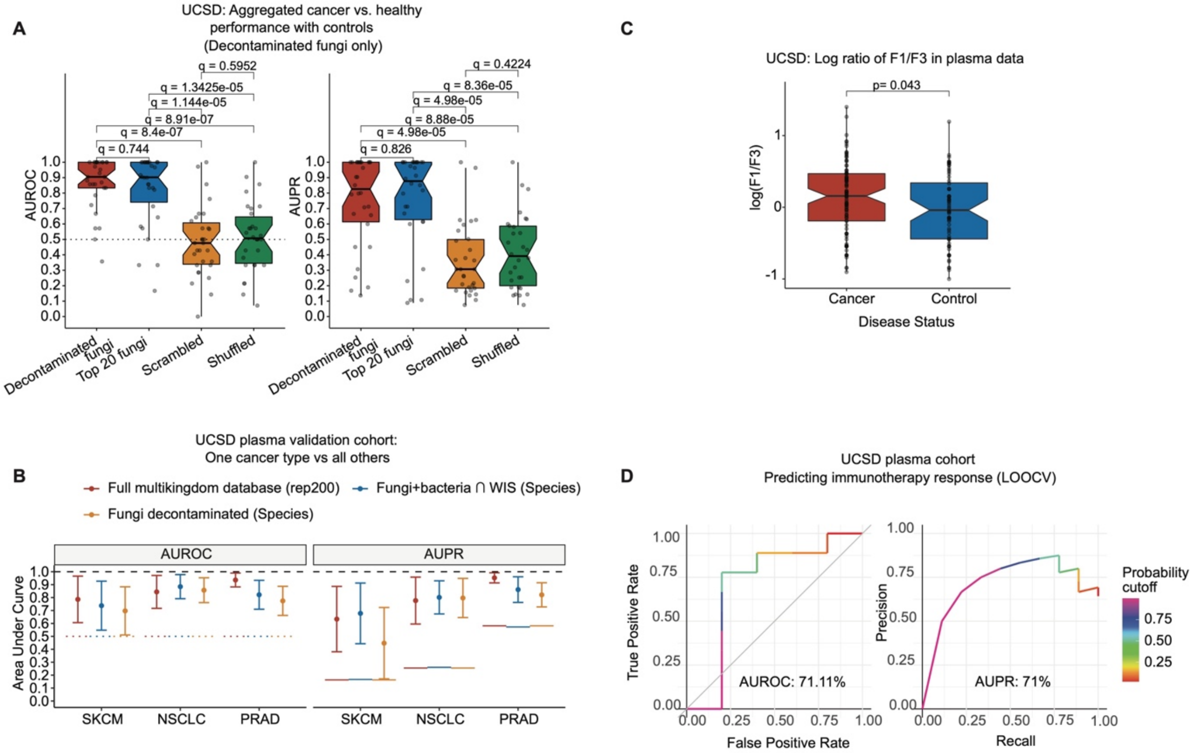

Data S6.3. Additional clinical and control analyses of fungal DNA in UCSD cohort plasma samples

(**A**) As negative controls in the UCSD cohort, machine learning models predicting each cancer type versus healthy individuals with plasma-derived fungi were re-evaluated using scrambled metadata or shuffled count data and compared to performance on actual biological samples. Biological comparisons used 215 decontaminated fungal species (x-axis "decontaminated") or the top 20 ranked fungi found to be most important in the Hopkins cohort pan-cancer versus healthy modeling (18 species overlapped with UCSD and were used). Since each fold of the biological samples comparing cancer versus healthy should show better than random performance, all folds from each cancer type (i.e., ten from each cancer type) are included in the biological sample boxplots, and each fold from the scrambled or shuffled controls are also shown. Pairwise two-sided Wilcox tests, corrected for multiple hypothesis testing using the Benjamini-Hochberg method, are shown on AUROC (left) and AUPR (right) data.

(**B**) Each cancer type versus all others machine learning performance in the UCSD plasma cohort across three feature sets: all microbial hits against the rep200 database (red; 7777 features), only fungal and bacterial species overlapping with the WIS cohort (blue; 281 species), and decontaminated fungi (215 species). Performance estimated using ten-fold cross-validation, with predictions on each holdout fold to calculate AUROC and AUPR. Centered dots denote average performance and error bars represent 95% confidence intervals. Horizontal, dotted, gray or colored lines represent null AUROC and AUPR values, respectively. Null AUPR values may slightly vary between feature sets when subsetting resulted in zero-sum samples that had to be removed prior to batch correction and/or machine learning.

(**C**) Log-ratios of UCSD-associated fungi identified in the TCGA mycotypes were also calculated between grouped cancer plasma samples versus healthy plasma samples, and the F1/F3 comparison was found to be significantly different by a two-sided Wilcox test.

(**D**) Fourteen melanoma patients in the UCSD plasma cohort had available immunotherapy response information. WIS-overlapping fungal species were batch corrected in the UCSD cohort (see STAR Methods) and tested using nested leave-one-out cross-validation, wherein models were iteratively trained using four-fold cross-validation on every *k-1* sample subset and used to predict the immunotherapy response status on the kth held out sample. The resultant list of predictions and observed responses were used to calculate the ROC (left) and PR (right) curves and their respective areas (inset on plots). The colored lines denote the probability cutoffs that result in a particular sensitivity and specificity on the ROC plot (left), or in a particular precision and recall on the PR plot (right).
